# Stereoselective synthesis of alkyl-, aryl-, vinyl- and alkynyl-substituted *Z*-enamides and enol ethers†
†Electronic supplementary information (ESI) available: Experimental and computational data. CCDC 1876011. For ESI and crystallographic data in CIF or other electronic format see DOI: 10.1039/c8sc05573d


**DOI:** 10.1039/c8sc05573d

**Published:** 2019-02-04

**Authors:** Paola Caramenti, Nina Declas, Romain Tessier, Matthew D. Wodrich, Jerome Waser

**Affiliations:** a Laboratory of Catalysis and Organic Synthesis , Institut des Sciences et Ingénierie Chimique , Ecole Polytechnique Fédérale de Lausanne , Lausanne , Ch-1015 , Switzerland . Email: jerome.waser@epfl.ch

## Abstract

Enamides and enol ethers are valuable building blocks in synthetic chemistry, yet their stereoselective synthesis can be challenging. Herein, we report a new stereoselective synthesis of vinyl, aryl, alkynyl, alkyl and thio-substituted *Z*-enamides and enol ethers based on the use of vinylbenziodoxolone (VBX) reagents. The stable VBX reagents were synthesized by stereoselective addition of N- or O-nucleophiles on the corresponding alkynyl reagents in the presence of a catalytic amount of cesium carbonate. The VBX reagents were used in palladium-catalyzed cross-couplings at room temperature to access *Z*-enamides and enol ethers.

## Abstract 2

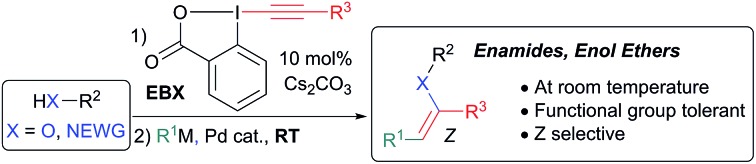
Highly stereoselective synthesis of *Z*-enamides and enol ethers at room temperature *via* an umpolung/cross coupling strategy applicable to drugs and natural products.

## 1. Introduction

**Scheme 1:**
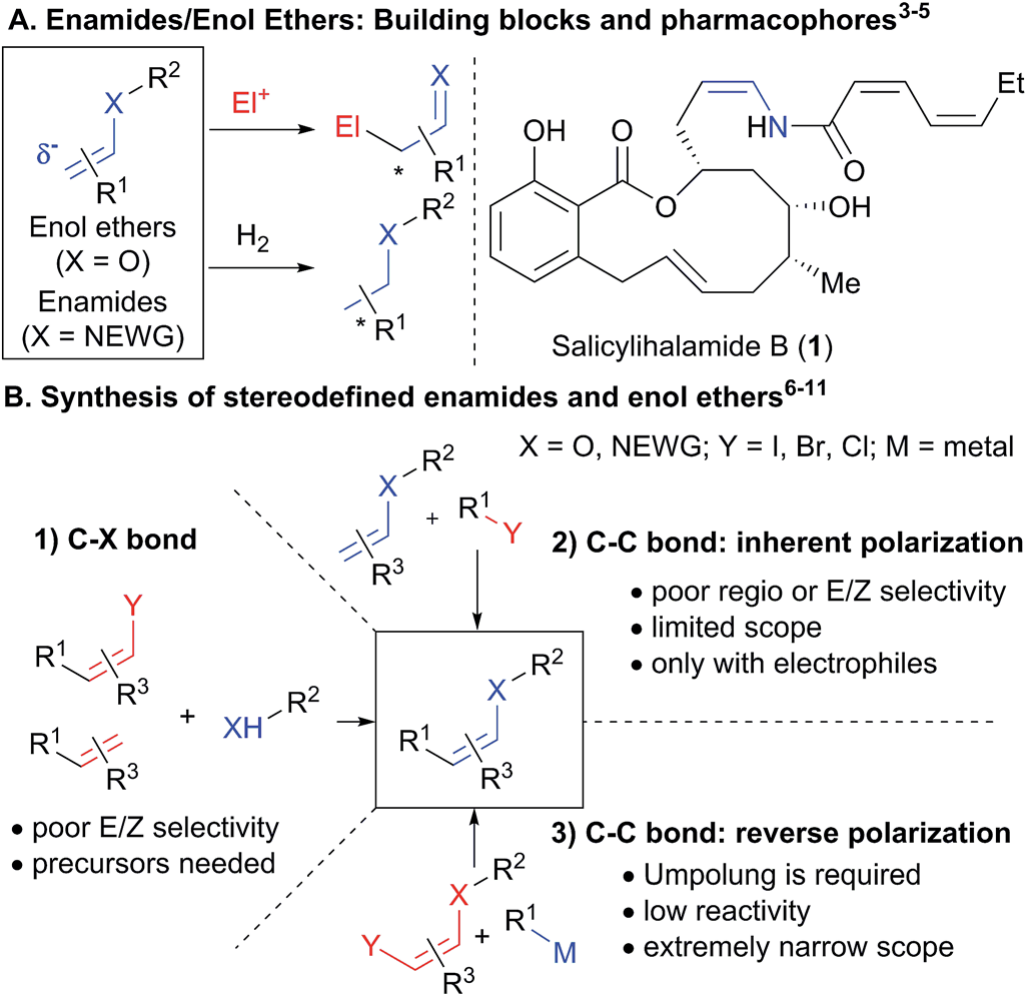
Enamides and enol ethers: importance and stereoselective synthesis.

The chemistry of carbonyl compounds, which can serve both as nucleophilic or electrophilic synthons, has been described as the backbone of organic synthesis.^1^ In particular, enolates, enamines and their derivatives have found broad applications as nucleophilic synthons.^2^ Enamides and enol ethers are especially attractive as nucleophiles due to their enhanced stability (Scheme 1(A)).^3^ In addition, they are valuable starting materials for the stereoselective synthesis of oxygen- and nitrogen-containing building blocks, especially *via* hydrogenation,^4^ as well as important pharmacophores in bioactive compounds, such as the natural product salicylihalamide B (**1**).^5^ Due to these numerous applications, the stereoselective synthesis of enamides and enol ethers is an important topic of research (Scheme 1(B)). Methods have been developed for the vinylation of amides, carbamates, alcohols and phenols,^6^ and the oxidative amidation of conjugated olefins (B1).^7^ However, these methods present issues of stereoselectivity or are based on the use of stereodefined starting materials, such as halogenated alkenes, which are often difficult to access. The selective synthesis of *Z*-enamides is particularly challenging.^8^ Alternatives to C–heteroatom bond formation have been developed,^9^ but lack convergence, as no new carbon–carbon or carbon–heteroatom bond is formed. Functionalization of enamides and enol ethers *via* C–C bond formation has recently been achieved when they are used as nucleophiles in C–H functionalization or Heck reactions (B2).^10^ In contrast, their use as electrophiles in C–C bond forming reactions has been much less exploited due to their low reactivity, limiting the range of available transformations (B3). Only the use of enol carboxylic and phosphonic esters and *trans*-iodo phthalimides has been reported.^11^ For these special substrates, the electron-density on oxygen/nitrogen is diminished by either one or two electron-withdrawing groups, making cross-coupling easier.

**Scheme 2:**
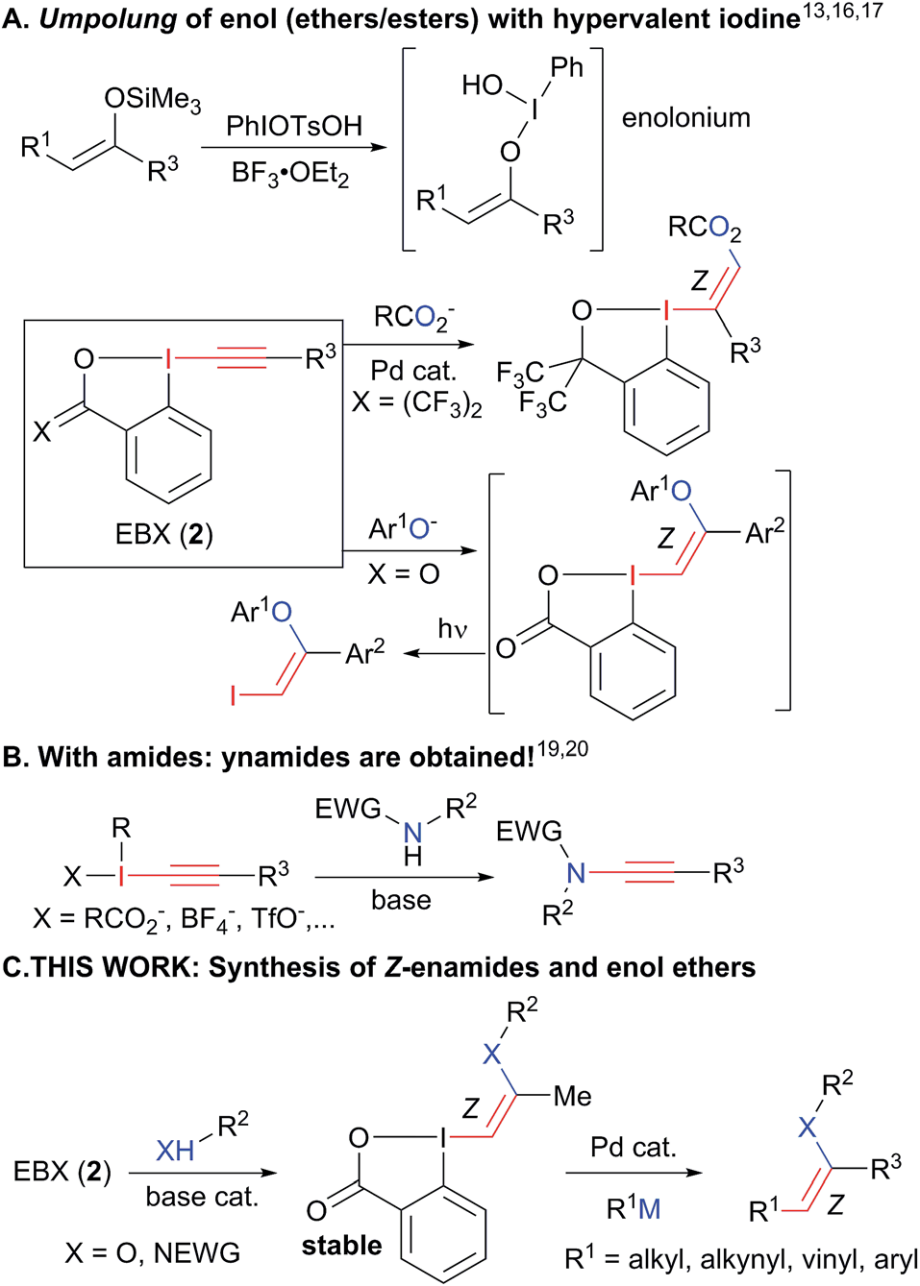
Umpolung with hypervalent iodine reagents: current limitation to enol esters and esters (A). Formation of ynamides with nitrogen nucleophiles (B) and our work on the synthesis of enamides and enol ethers (C).

To succeed in the general use of enamides and enol ethers as electrophiles in C–C bond forming reactions, a more efficient umpolung of their inherent reactivity is therefore required. In this context, hypervalent iodine is well established for its capability to reverse the polarization of nucleophiles.^12^ Recently, Szpilman and coworkers used iodonium salts for the umpolung of enolates (Scheme 2(A)).^13^ The enolonium is generated *in situ* and cannot be isolated. Heteroatom substituted alkenyliodonium salts were reported only in the case of derivatives bearing less electron-rich fluorides and sulphonates substituents.^14^ Cyclic hypervalent iodine reagents, especially benziodoxoles, display enhanced stability.^15^ In 2016, Yoshikai and coworkers reported the palladium-catalyzed addition of carboxylates onto EthynylBenziodoXole (EBX) reagents to give the corresponding oxygen-substituted VinylBenziodoXoles (VBX), and used the latter in cross-coupling reactions.^16^ This was an important breakthrough in the development of stable reagents for the umpolung of enol esters. Nevertheless, the method required the use of a palladium catalyst and more expensive and difficult to access hypervalent iodine reagents bearing two trifluoromethyl groups. Furthermore, no umpolung of enamides was reported. In 2018, Miyake and co-workers demonstrated that phenols can be efficiently added onto aryl-EBX reagents derived from cheaper benzoic acid with high *Z* selectivity without the need of a transition metal catalyst.^17^ However, the formed VBX reagents displayed limited stability and only a single example was isolated in low yield. Therefore, they were immediately converted to iodides under light irradiation, preventing the exploitation of the highly reactive hypervalent bond in other transformations. In contrast to these first successes in the umpolung of enol esters and ethers, there is to the best of our knowledge no report for the umpolung of enamides. Only azide-substituted vinyl hypervalent iodine reagents have been reported by Kitamura and co-workers in 1997.^18^ In fact, it is well-known that the reaction of alkynyliodonium salts^19^ or EBX reagents^20^ with amides give directly ynamides as products (Scheme 2(B)). For this reason, hypervalent iodine reagents could not be used for the umpolung of enamides so far.

Herein, we report the first general synthesis of *Z*-enamides and enol ethers based on an umpolung-cross coupling approach (Scheme 2(C)). Enamide/enol ether-substituted benziodoxolone reagents were obtained by addition of tosyl amides or phenols onto alkyl substituted EBXs using a catalytic amount of base. The mild reaction conditions tolerated numerous functional groups, allowing the modification of drugs and natural products. The reaction proceeded with high *Z* selectivity. The new reagents are stable and could be engaged in a broad range of cross-coupling reactions, enabling the stereoselective synthesis of alkyl, aryl, vinyl and alkynyl enamides and enol ethers.

## 2. Results and discussions

### Synthesis of new VBX reagents

With the aim of accessing nitrogen-substituted VBX reagents, we first investigated the new synthesis recently reported by Olofsson and co-workers involving the reaction of alkenyl boronic acids with *in situ* generated iodine(iii) precursors.^21^ However, we could never isolate the desired reagents using these reaction conditions. Therefore, we decided to re-investigate the addition of amide nucleophiles onto EBX reagents, despite the negative precedence.^19^,^20^ In our previous work on alkynylation of thiols, we observed the formation of sulfur-substituted VBX reagents in trace amounts.^22^ Interestingly, the amount of this side product could be increased from <5% to 20% using a catalytic amount of base. We therefore decided to use similar conditions (10 mol% tetramethylguanidine (TMG) in THF) in the screening of nitrogen nucleophiles for the addition on EBX reagent **2a** (Table 1). Gratifyingly, the desired product **4a** could be obtained in 22% yield using *para*-methoxyphenyl (PMP)-substituted tosyl amide **3a**, whereas carbamate **3b**, amide **3c**, bistosylimide **3d** and phthalimide (**3e**) were not successful (entry 1). In this case, the main issue was decomposition. Besides the desired product, only 2-iodo benzoic acid could be isolated, indicating a reduction at the iodine atom. A slight increase in yield was observed with CH_2_Cl_2_ (entry 2) and alcohols, such as MeOH and EtOH (entries 3 and 4) as solvents. Weaker organic bases led to a further increase in yield (entries 5 and 6). Switching to inorganic bases afforded cleaner reactions and higher yields (entries 7–11). The best result was obtained with cesium carbonate (93% NMR yield, entry 11). Compound **4a** was stable and could be purified with only minor loss by column chromatography (68% isolated yield). Finally, 10 mol% of cesium carbonate was confirmed as the best base loading (entries 12 and 13). In fact, the use of base in catalytic amount is essential to avoid decomposition or formation of the alkyne product as was observed previously.^19^,^20^ The developed optimized conditions are highly convenient, as the reaction can be done in ethanol in an open flask using a 1 : 1 ratio of amide **3a** and EBX **2a** to give **4a** with complete *Z*-stereoselectivity.^23^

**Table 1:** Optimization of the synthesis of amido-VBX **4a**

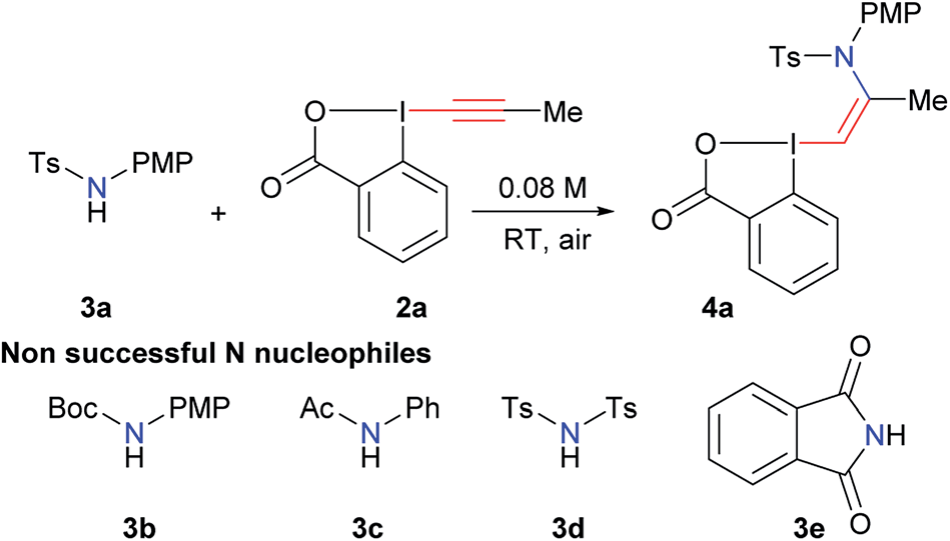			
Entry	Solvent	Base	Yield^*a*^
1	THF	10 mol% TMG	22
2	CH_2_Cl_2_	10 mol% TMG	28
3	MeOH	10 mol% TMG	23
4	EtOH	10 mol% TMG	30
5	EtOH	10 mol% NEt_3_	43
6	EtOH	10 mol% pyridine	38
7	EtOH	10 mol% NaHCO_3_	81
8	EtOH	10 mol% KHCO_3_	79
9	EtOH	10 mol% CsHCO_3_	83
10	EtOH	10 mol% CsOH·H_2_O	54
**11**	**EtOH**	**10 mol% Cs**_**2**_ **CO**_**3**_	**93(68)** ^*b*^
12	EtOH	25 mol% Cs_2_CO_3_	46
13	EtOH	5 mol% Cs_2_CO_3_	39

^*a*^Reactions conditions: 0.10 mmol **3a**, 0.10 mmol **2a**, 10 μmol base, 0.08 M in indicated solvent, RT, air, 14 h. NMR yield using 0.39 mol% of 1,3,5-trimethoxybenzene as internal standard.

^*b*^Isolated yield after column chromatography on silica gel.

**Scheme 3:**
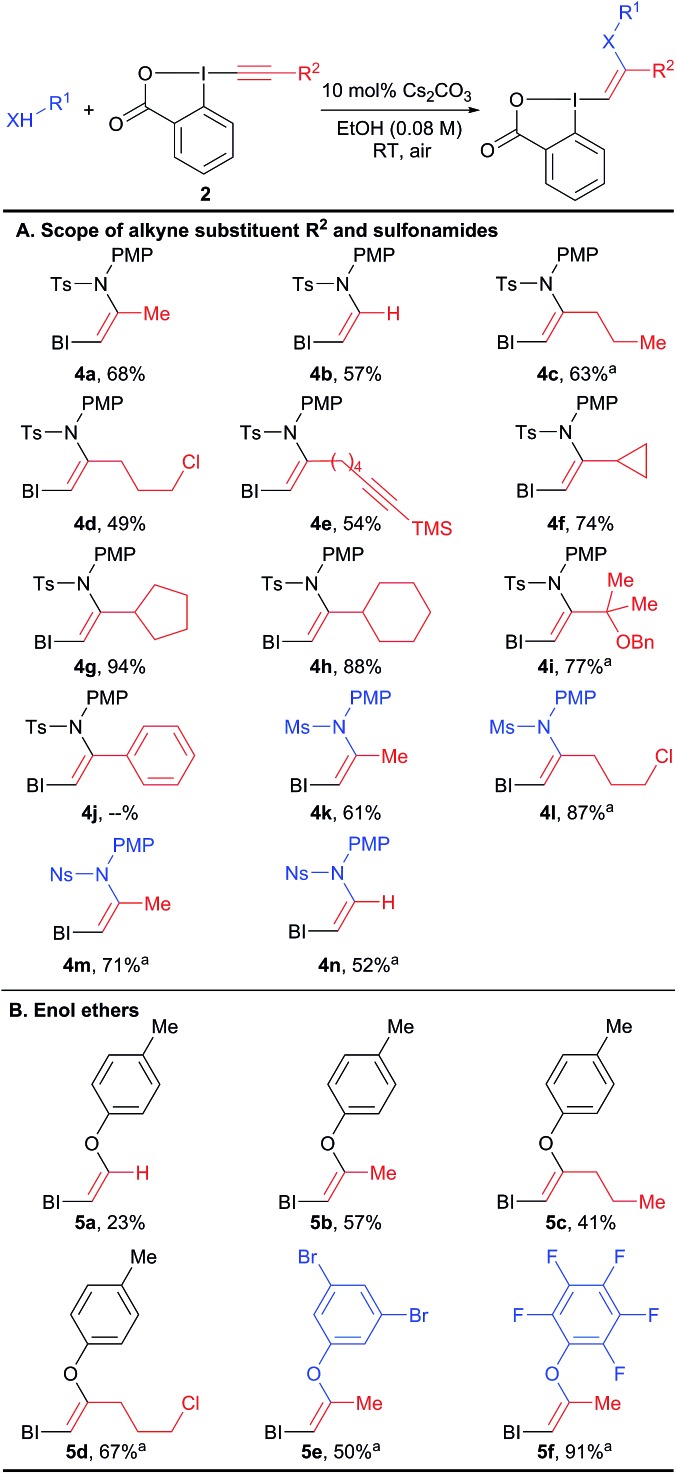
Scope of VBX reagents. Reactions performed on 1.0 mmol scale. BI = benziodoxolone. ^a^Reaction performed on 0.10 mmol scale.

The scope of the reaction was then investigated (Scheme 3) we focused first on the alkyne substituent on EBX **2** (Scheme 3(A)). In addition to methyl-substituted **4a**, the unsubstituted *Z*-enamide **4b** was obtained in 57% yield starting from a silylated EBX reagent. Reagents **4c–e** bearing primary alkyl chains and functional groups such as a chlorine and an alkyne were also obtained in good yield. Cyclopropyl, cyclopentyl and cyclohexyl derivatives **4f–h** could be isolated in excellent yields (74–94%). A sterically encumbered tertiary substituent was also well tolerated (product **4i**). In contrast, an aryl substituent was not tolerated, leading to decomposition. Both a smaller mesyl and a nosyl sulfonamides could be used to give reagents **4k–n** in good yields. Nosyl groups are in principle easier to cleave than tosyl groups. The same protocol could be also applied to phenols as nucleophiles (products **5a–f**, Scheme 3(B)). The obtained enol ethers bear alkyl substituents in contrast to those reported by Miyake and co-workers and displayed enhanced stability.

**Scheme 4:**
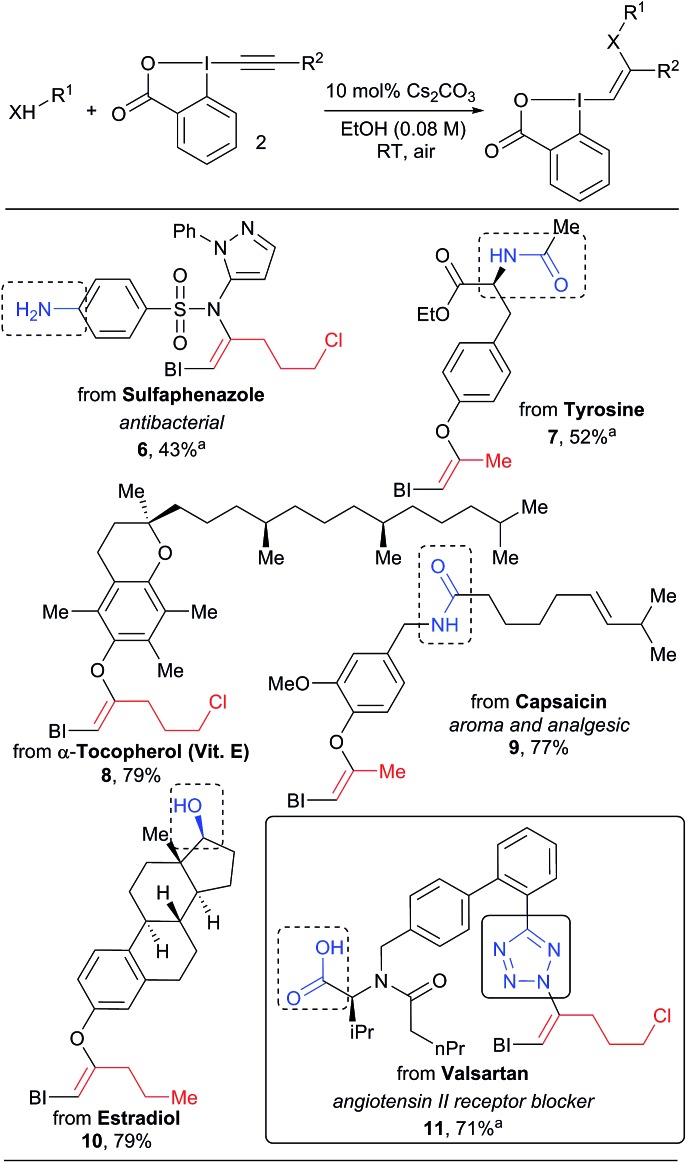
Scope of natural products and drugs. Reactions performed on 1.0 mmol scale. ^a^Reaction performed on 0.10 mmol scale.

In all the previous work involving the synthesis and use of VBX reagents, focus had been restricted to only small organic molecules.^16^–^18^,^21^ When considering the very mild conditions developed in our work, we wondered if the approach could be used for the functionalization of more complex natural products and drugs (Scheme 4). These compounds present multiple heteroatoms and nucleophilic positions, leading to challenges in selectivity. Gratifyingly, our mild protocol allowed the functionalization of the sulfa drug sulfaphenazole in 43% yield to give VBX **6** without reaction of the free aromatic amine. Hypervalent iodine reagents derived from bioactive complex phenols such as tyrosine, α-tocopherol, capsaicin and estradiol derivatives **7–10** were also obtained in 40–79% yield. In this case, both amides and aliphatic alcohols were tolerated. The high selectivity observed is striking and is probably originates from the deprotonation of the most acidic O–H or N–H bond to generate the active nucleophile. The transformation was also successful for other acidic nitrogen functionalities: the tetrazole heterocycle of valsartan reacted as a nucleophile to give benziodoxolone **11**. No reaction with the carboxylic acid was observed, in contrast to the work of Yoshikai and co-workers.^16^ The inertness of the carboxylate is not well-understood at this stage, but it is important to note that Yoshikai's functionalization required a palladium catalyst.

### Speculative reaction mechanism

**Fig. 1:**
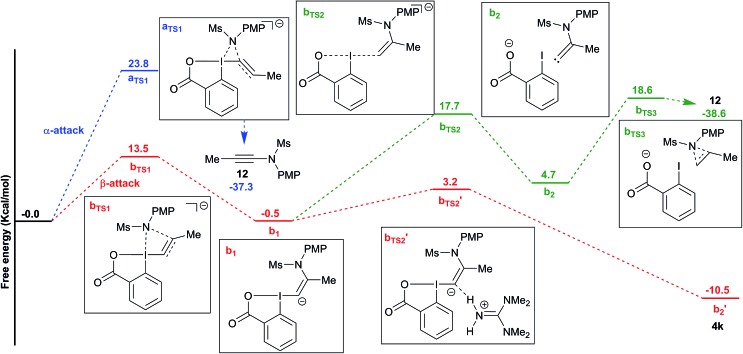
Reaction free energy profile for the addition of amide **3f** to EBX **2a** with TMG in THF.

**Fig. 2:**
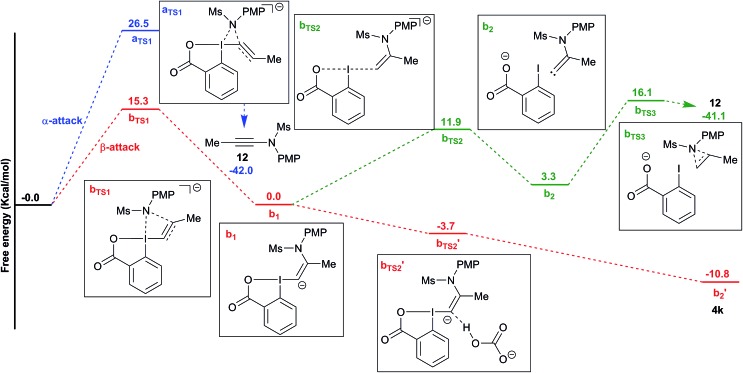
Reaction free energy profile for the addition of amide **3f** to EBX **2a** with cesium carbonate in ethanol.

To better understand the switch in reactivity when using a catalytic amount of base, we turned to computational chemistry. DFT analysis (at the PBE0-dDsC/TZ2P//M06/def2-SVP level) for the addition of amide **3f** on Me-EBX (**2a**) was first performed with TMG as a base and THF as a solvent, the conditions under which the reaction had been discovered. We were able to locate an α and a β-addition transition state **a_TS1_** and **b_TS1_** (Fig. 1), as shown in our previous work on thiol nucleophiles.^22^,^24^ In the case of a nitrogen nucleophile, β addition was favored by 10.3 kcal mol^–1^ leading to intermediate **b_1_**. The protonation of **b1** is very easy with a barrier of only 3.2 kcal mol^–1^ to give vinylbenziodoxolone **4k**, whereas breaking of the C–I bond requires 17.7 kcal mol^–1^, leading to formation of carbene intermediate **b2**. The alkyne product **12** can then be obtained after 1,2-amine shift with a barrier of 18.6 kcal mol^–1^ in a highly exergonic reaction. Interestingly, the barrier for the deprotonation of **4k** back to **b1** is only 13.7 kcal mol^–1^. We then repeated the calculations in ethanol using carbonate as a base (Fig. 2). The energies of both α- and β-additions were slightly higher under these conditions, with β-addition being even more favored (11.2 kcal mol^–1^). From intermediate **b1**, protonation was barrierless and the energy for carbon–iodine bond breaking was significantly lower (from 17.7 to 11.9 kcal mol^–1^). However, the difference between both transition states did not change significantly. Finally, the barrier for 1,2-amine shift was only slightly lower.

**Scheme 5:**
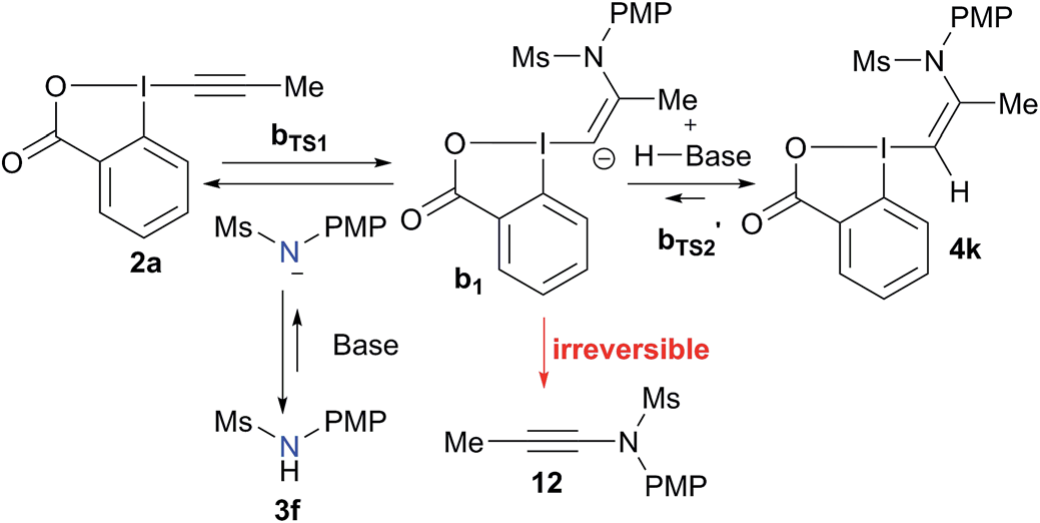
Speculative mechanism for the selective formation of VBX **4k**.

Based on these results, a speculative mechanism for the selective formation of VBX **4k** can be proposed (Scheme 5). In the presence of a base, a small amount of amide **3f** is deprotonated and reacts fast and reversibly with EBX **2a** to form anion **b1**. **b1** is itself in equilibrium with VBX **4k** by re-protonation. The equilibrium of **2a**, **b1** and **4k** lies strongly in favor of **4k**, allowing its isolation once the reaction mixture is neutralized. On the other hand, **b1** reacts slowly and irreversibly *via* carbene **b2** to form alkyne **12**. Higher base concentration leads to increased amount of **b1**, resulting finally in full conversion to ynamide **12**. According to the computation results, the formation of VBX **4k** is favored over ynamides **12** with about the same energy difference under preliminary and optimized conditions. The better yields obtained are probably due to suppression of decomposition pathways.

### Functionalization of the VBX products

**Scheme 6:**
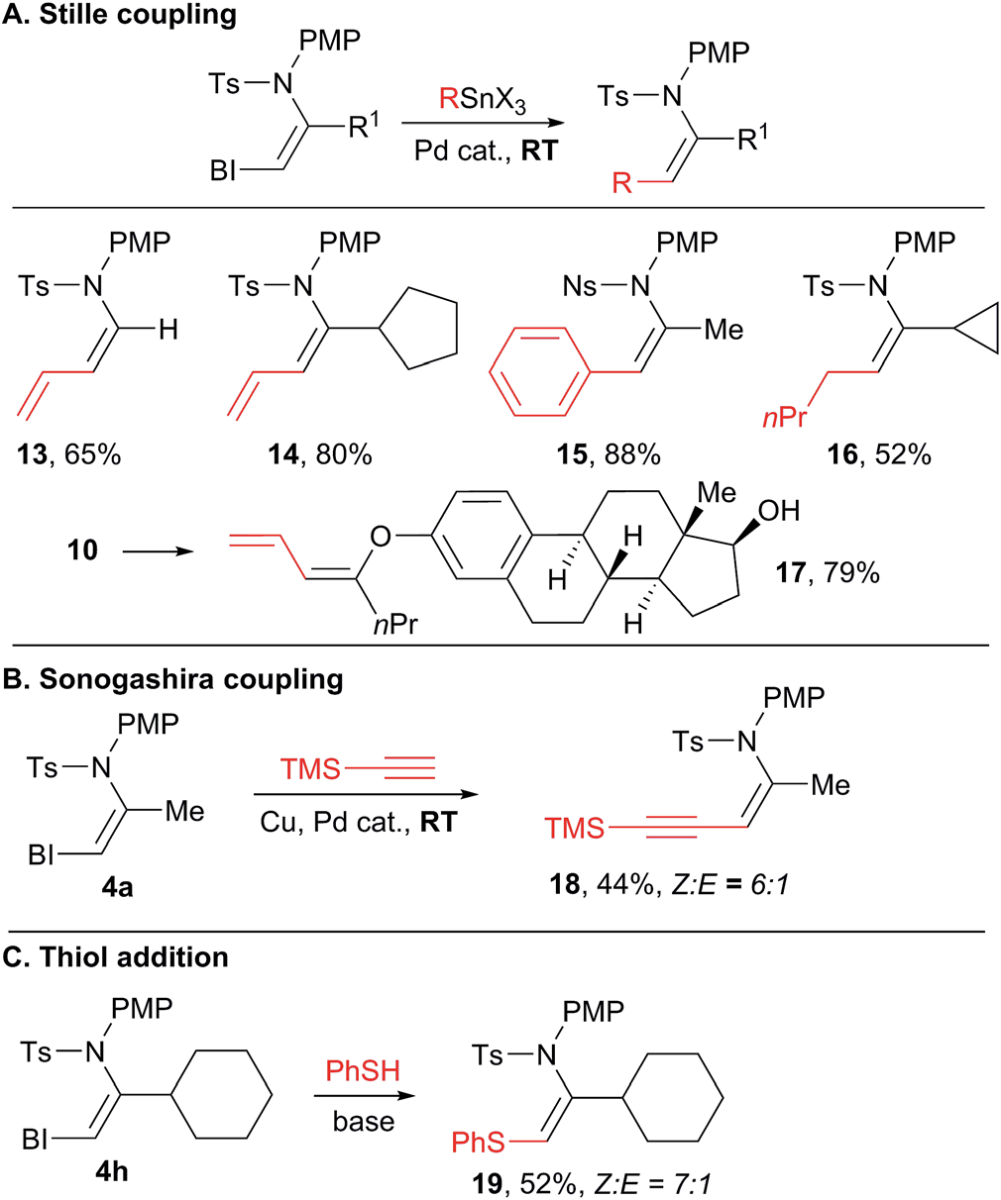
Functionalization of the VBX products. Reaction conditions: (A) 0.10 mmol VBX reagent, 0.20 mmol stannane, 5 mol% Pd(PhCN)_2_Cl_2_, 0.1 M in DMF, 10 h, RT. (B) 0.10 mmol VBX reagent **4a**, 0.30 mmol trimethylsilylacetylene, 5 mol% Pd(PPh_3_)_2_Cl_2_, 20 mol% CuI, 0.10 mmol NEt_3_, 0.1 M in DMF, 10 h, RT. (C) 0.10 mmol VBX reagent **4h**, 0.10 mmol phenylthiol, 0.12 mmol potassium *tert*-butoxide, 0.1 M in DME, 16 h, RT.

With a broad scope of functionalized VBX reagents in hand, we investigated their conversion into the desired enamides (Scheme 6). Palladium-catalyzed Stille cross-coupling was investigated first (Scheme 6(A)). The coupling of vinyl, aryl and alkyl stannyl reagents to give products **13–16** was possible at room temperature. Diene enamides are especially sensitive compounds and only a few synthetic methods have been reported to access them.^25^ Stille cross-coupling with an enol ether also proceeded smoothly at room temperature (product **17**), whereas similar reaction with simple iodides required heating at 80–120 °C.^17^,^26^ In all Stille couplings, complete stereospecificity was observed and only the *Z* products were obtained. Enyne **18** was then obtained in a 6 : 1 *Z* : *E* ratio through a Sonogashira coupling (Scheme 6(B)).^27^ Finally, the addition of a strong thiol nucleophile was possible without a transition metal catalyst to give thioenamide **19** (Scheme 6(C)).8a–c

In general, sulfonyl enamides have been broadly used in synthetic chemistry, for example as partners in cycloaddition reactions,^28^ in addition to iminiums^29^ or in oxidative heterofunctionalization reactions.^30^ Interestingly, most of these studies focused on the use of *E*-enamides, due to the difficulties in accessing the *Z* isomers. Easier access to *Z*-enamides will enhance the utility of these methodologies by enabling the synthesis of other stereoisomers.

**Scheme 7:**
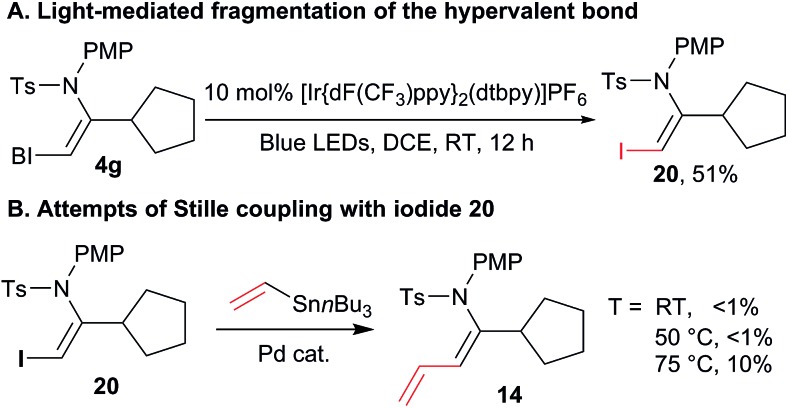
Comparison of the reactivity of enamide iodides and enamide benziodoxolones. Reaction conditions: (A) 0.10 mmol VBX reagent **4g**, 10 mol% [Ir{dF(CF_3_)ppy}2(dtbpy)]PF_6_, 0.1 M in DCE, blue LEDS irradiation, 12 h, RT. (B) 0.10 mmol VBX reagent, 0.20 mmol stannane, 5 mol% Pd(PhCN)_2_Cl_2_, 0.1 M in DMF, 10 h, at the indicated temperature.

Nevertheless, we were missing a direct comparison with the reactivity of simple iodides in the case of enamide substrates. We were unable to design an efficient synthesis of the corresponding iodo enamides using other methods. Although this already demonstrates an important synthetic advantage of our approach, we were still interested in directly comparing the reactivity of standard and hypervalent iodine bonds in the cross-coupling reaction. Fortunately, when the photoredox conditions reported by Miyake and co-workers were applied to VBX **4g**, iodide **20** was obtained in 51% yield (Scheme 7(A)). This demonstrated that Miyake's procedure can also be applied to certain alkyl-substituted VBX reagents. Stille cross-coupling was then attempted with iodide **20**, but no conversion was observed at room temperature and 50 °C. At 75 °C, less then 10% of the desired product was observed by ^1^H NMR, together with significant decomposition. This result definitively demonstrated the higher reactivity and synthetic utility of the VBX enamide reagents.

## 3. Conclusions

In summary, we have developed a highly stereoselective synthesis of *Z*-enamides based on the use of vinylbenziodoxolone reagents. The key for success was the use of a catalytic amount of base, which avoided the formation of the thermodynamically favored ynamides. The obtained nitrogen-substituted VBX reagents were stable even to column chromatography and could be obtained in high yield under very mild reaction conditions, tolerating many functional groups. The reaction could be also extended to phenols as substrates. The high reactivity of the hypervalent iodine bond allowed the formation of aryl, vinyl, alkynyl, alkyl and thio-substituted *Z*-enamides as well as enol ethers with high stereospecificity at room temperature. This general access to *Z*-enamides and enol ethers will facilitate their broader use in synthetic chemistry.

## Conflicts of interest

There are no conflicts to declare.

## Supplementary Material

Supplementary information

Crystal structure data
