# Supplementary material for: Stereoselective synthesis of alkyl-, aryl-, vinyl- and alkynyl-substituted Z-enamides and enol ethers
Source: Chem Sci. 2019 Feb 4;10(11):3223–30. doi: 10.1039/c8sc05573d (PMC6430016; doi:10.1039/c8sc05573d)

Electronic Supplementary Material (ESI) for Chemical Science.
This journal is © The Royal Society of Chemistry 2019

Supporting Information

Table of contents

1. Materials and Methods. ... 2

2. Preparation of starting materials. ... 4

2.1 Preparation of 1-[(trimethylsilyl)ethynyl]-1,2-benziodoxol-3(1*H*)-one 2b. ... 4

2.2 Synthesis of EBX. ... 6

3. Optimization of the synthesis of N-vBX 5a. ... 13

4. Scope of N-vBX and O-vBXs. ... 16

4.1 General Procedure GPX for the Synthesis N-vBX and O-vBX. ... 16

5. Further functionalization employing N-vBX and O-vBX. ... 36

5.1 C-C bond formation. ... 36

5.2 C-Heteroatom bond formation. ... 42

5.3 Control experiment ... 45

6. DFT calculations. ... 47

6.1 Computational Details ... 47

7. Crystal Structure. ... 51

8. Spectra of new compounds ... 52

1. Materials and Methods.

All reactions were carried out in oven dried glassware under an atmosphere of nitrogen, unless stated otherwise. For quantitative flash chromatography, technical grade solvents were used. For flash chromatography for analysis, HPLC grade solvents from Sigma-Aldrich were used. THF, Et_2O, CH_3CN, toluene, hexane and CH_2Cl_2 were dried by passage over activated alumina under nitrogen atmosphere (H_2O content < 10 ppm, *Karl-Fischer* titration). The solvents were degassed through Freeze-Pump-Thaw method when mentioned. All chemicals were purchased from Acros, Aldrich, Fluka, VWR, Aplichem, or Merck and used as such unless otherwise stated. Chromatographic purification was performed as flash chromatography using Macherey-Nagel silica 40-63, 60 Å, with the solvents indicated as eluent under 0.1-0.5 bar pressure. TLC was performed on Merck silica gel 60 F_{254} TLC glass plates or aluminium plates and visualized with UV light, permanganate stain, CAN stain, or Anisaldehyde stain. Melting points were measured on a Büchi B-540 melting point apparatus using open glass capillaries, the data is uncorrected. ^{1}H-NMR spectra were recorded on a Brucker DPX-400 400 MHz spectrometer in $CDCl_3$, DMSO-d_6 CD_3OD, C_6D_6 and CD_2Cl_2, all signals are reported in ppm with the internal chloroform signal at 7.26 ppm, the internal DMSO signal at 2.50 ppm the internal methanol signal at 3.30 ppm, the internal dichloromethane signal at 5.30 ppm as standard. The data is being reported as (s = singlet, d = doublet, t = triplet, q = quadruplet, qi = quintet, m = multiplet or unresolved, br = broad signal, app = apparent, coupling constant(s) in Hz, integration, interpretation).^{13}C-NMR spectra were recorded with ^{1}H-decoupling on a Brucker DPX-400 100 MHz spectrometer in $CDCl_3$, DMSO-d_6, CD_3OD or CD_2Cl_2, all signals are reported in ppm with the internal chloroform signal at 77.0 ppm, the internal DMSO signal at 39.5 ppm, the internal methanol signal at 49.0 ppm and the internal dichloromethane signal at 54.0 ppm as standard. Infrared spectra were recorded on a JASCO FT-IR B4100 spectrophotometer with an ATR PRO410-S and a ZnSe prisma and are reported as cm^{-1} (w = weak, m = medium, s = strong, br = broad). High resolution mass spectrometric measurements were performed by the mass spectrometry service of ISIC at the EPFL on a MICROMASS (ESI) Q-TOF Ultima API. Photoredox transformations were performed with the reaction flask hold using a rack for test tubes placed at the center of a crystallization flask. On this flask were attached the blue LEDs (Ruban LED avec câble à extrémités ouvertes Barthelme Y51516414 182405 24 V 502 cm bleu 1 pc(s), bought directly on www.conrad.ch/fr). The distance

between the LEDs and the test tubes was approximatively 3 to 4 cm. Long irradiation for more than 2 h resulted in temperature increasing up to 34 °C.

2. Preparation of starting materials.

The synthesis of the precursors for EBX reagents **2a-2j** and their starting materials had been already described before in our group.[1,2] The procedures here reported are taken from the cited publications to facilitate reproduction of the results by having all the data in the same file.

The synthesis of R-EBX reagents **2a-2j** except **2c, 2g, 2h** had been already described before. The procedures are taken here from the indicated publications to facilitate reproduction of the results by having all the data in the same file.

<u>2.1 Preparation of 1-[(trimethylsilyl)ethynyl]-1,2-benziodoxol-3(1*H*)-one 2b.</u>

1-Hydroxy-1,2-benziodoxol-3-(1H)-one (22)

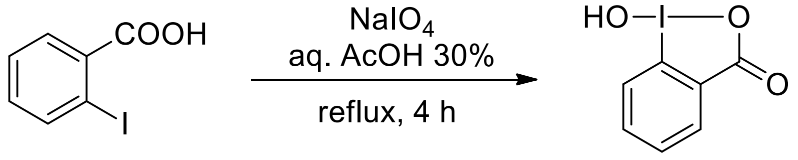

21 **22**

Following a reported procedure,[1] $NaIO_4$ (7.24 g, 33.8 mmol, 1.05 equiv.) and 2-iodobenzoic acid **21** (8.00 g, 32.2 mmol, 1.00 equiv.) were suspended in 30% (v/v) aq. AcOH (48 mL). The mixture was vigorously stirred and refluxed for 4 h. The reaction mixture was then diluted with cold water (180 mL) and allowed to cool to room temperature, protecting it from light. After 1 h, the crude product was collected by filtration, washed on the filter with ice water (3 x 20 mL) and acetone (3 x 20 mL), and air-dried in the dark to give the pure product 1-hydroxy-1,2-benziodoxol-3-(1H)-one **22** (8.3 g, 31 mmol, 98%) as a white solid. **^{1}H NMR** (400 MHz, $(CD_3)_2SO$): δ 8.02 (dd, *J* = 7.7, 1.4 Hz, 1H, Ar*H*), 7.97 (m, 1H, Ar*H*), 7.85 (dd, *J* = 8.2, 0.7 Hz, 1H, Ar*H*), 7.71 (td, *J* = 7.6, 1.2 Hz, 1H, ArH). **^{13}C NMR** (100 MHz, $(CD_3)_2SO$): δ 167.7, 134.5, 131.5, 131.1, 130.4, 126.3, 120.4. **IR** ν 3083 (w), 3060 (w), 2867 (w), 2402 (w), 1601 (m), 1585 (m), 1564 (m), 1440 (m), 1338 (s), 1302 (m), 1148 (m), 1018 (w), 834 (m), 798 (w), 740 (s), 694 (s), 674 (m), 649 (m). The values of the NMR spectra are in accordance with reported literature data.[1]

---

[1] R. Frei, M. D. Wodrich, D. P. Hari, P.-A. Borin, C. Chauvier, J. Waser, *J. Am. Chem. Soc.* 2014, **136**, 16563.
[2] D. P. Hari, J. Waser, *J. Am. Chem. Soc.* 2016, **138**, 2190.

1-[(Trimethylsilyl)ethynyl]-1,2-benziodoxol-3(1*H*)-one (TMS-EBX, 2b)

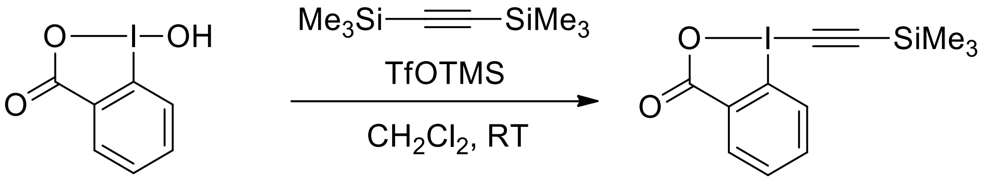

22 **2b**

Following a slight modification of the reported procedure,[3] trimethylsilyl triflate (5.54 mL, 30.7 mmol, 1.10 equiv) was added to a suspension of 2-iodosylbenzoic acid **22** (7.36 g, 28.0 mmol, 1.00 equiv) in CH_2Cl_2 (85 mL) at RT. The resulting yellow mixture was stirred for 1 h, followed by the dropwise addition of bis(trimethylsilyl)acetylene (6.98 mL, 30.7 mmol, 1.10 equiv). The resulting suspension was stirred for 6 h at RT, during this time a white solid was formed. A saturated solution of $NaHCO_3$ was then added and the mixture was stirred vigorously until completely solubilization of the white solid. The two layers were separated and the combined organic extracts were washed with sat. $NaHCO_3$, dried over $MgSO_4$, filtered and evaporated under reduce pressure. Recrystallization from acetonitrile (5 mL) afforded 1-[(trimethylsilyl)ethynyl]-1,2-benziodoxol-3(1*H*)-one **2b** (7.17 g, 20.8 mmol, 74%) as a colorless solid. **Mp:** 143-145°C (dec). **^{1}H NMR**(400 MHz, Chloroform-*d*) δ 8.42 (dd, *J*= 6.4, 1.9 Hz, 1 H; Ar*H*), 8.19 (m, 1 H; Ar*H*), 7.78 (m, 2 H; Ar*H*), 0.32 (s, 9 H; TMS). **^{13}C NMR** (100 MHz, $CDCl_3$) *δ* 166.4, 134.9, 132.6, 131.7, 131.4, 126.1, 117.2, 115.4, 64.2, -0.5. **IR** ν 3389 (w), 2967 (w), 1617 (s), 1609 (s), 1562 (m), 1440 (w), 1350 (m), 1304 (w), 1254 (w), 1246 (w), 1112 (w), 1008 (w), 852 (s), 746 (m), 698 (m), 639 (m). The characterization data for compound **2b** corresponded to the reported values.[4]

---

[3] V. V Zhdankin, C. J. Kuehl, A. P. Krasutsky, J. T. Bolz, A. J. Simonsen, *J. Org. Chem.* 1996, **61**, 6547.
[4] D. Fernández González, J. P. Brand, J. Waser, *Chem. – A Eur. J.* 2010, **16**, 9457.

2.2 Synthesis of EBX.

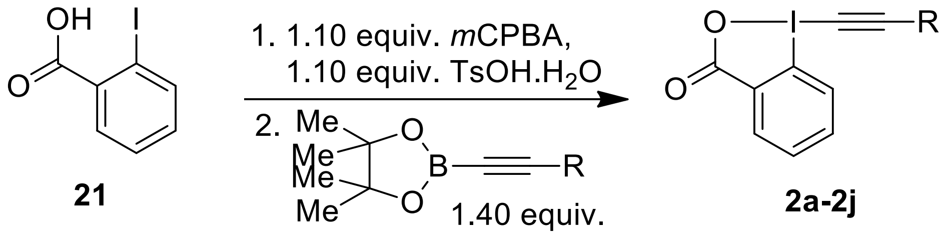

GP1: Following a slightly modified procedure,[5] 2-iodobenzoic acid **21** (1.00 equiv.), *para*-toluenesulfonic acid monohydrate (1.10 equiv.) and *meta*-chloroperoxybenzoic acid (*m*CPBA-70%, 1.10 equiv.) were dissolved in dichloromethane and 2,2,2-trifluoroethanol (1:1 mixture, 0.27 M). The mixture was stirred at room temperature under nitrogen for 1 hour, after which the correspondent alkyl-1-boronic acid pinacol ester (1.40 equiv.) was added in one portion. The reaction mixture was stirred for 2.5 hours at room temperature, filtered and concentrated *in vacuo*. The resulting oil was dissolved in dichloromethane (30 mL) and under vigorous stirring, saturated aq. $NaHCO_3$ (30 mL) was added. The mixture was stirred for 15 minutes, the two layers were separated and the aqueous phase was extracted with additional portions of dichloromethane (3 x 25 mL). The combined organic layers were washed with brine (25 mL), dried over $MgSO_4$, filtered and concentrated *in vacuo*. The crude product was purified by flash column chromatography (DCM:MeOH 9:1) to afford the desired compounds **2a-2j**.

Propynyl-1,2-benziodoxol-3(1*H*)-one (2a)

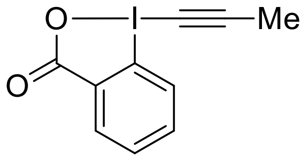

Following **GP1** on 4.30 mmol scale and using propynyl-1-boronic acid pinacol ester (4.85 g, 21.2 mmol, 1.40 equiv.), propynyl-1,2-benziodoxol-3(1*H*)-one **2a** (1.03 g, 3.60 mmol, 84%) was obtained as a white solid. **R*f*** 0.10 (EtOAc). **Mp** 124-150 °C (decomposition). **^{1}H NMR** (400 MHz, Chloroform-*d*) δ 8.41-8.35 (m, 1 H, Ar*H*), 8.22-8.14 (m, 1 H, Ar*H*), 7.79-7.68 (m, 2H, Ar*H*), 2.27 (s, 3H, CCH_3). **^{13}C NMR** ($CDCl_3$, 100 MHz) δ 166.7, 134.8, 132.5, 131.6, 126.4, 115.6, 105.1, 39.0, 5.7

[5] M. J. Bouma, B. Olofsson, *Chem. – A Eur. J.* 2012, **18**, 14242.

(*one carbon aromatic signal not resolved*). **IR** ν 2183 (w), 1607 (s), 1559 (m), 1350 (m), 746 (m), 730 (m). **HRMS** (ESI) $C_{10}H_8IO_2^+$ $[M+H]^+$ 286.9564; found 286.9561.

(Pent-1-ynyl)-1,2-benziodoxol-3(1*H*)-one (2c)

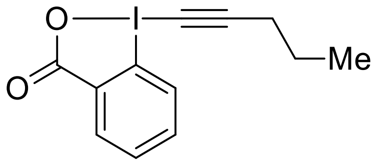

Following **GP1** on 4.00 mmol scale and using 1-pentynyl-1-boronic acid pinacol ester (1.09 g, 4.60 mmol, 1.15 equiv.) (pent-1-ynyl)-1,2-benziodoxol-3(1*H*)-one **2c** (0.754 g, 2.40 mmol, 60%) was obtained as a white oil.[6] **¹H NMR** (400 MHz, Chloroform-*d*) δ 8.40 (ddd, *J* = 7.4, 3.8, 2.3 Hz, 1H, Ar*H*), 8.26 – 8.09 (m, 1H, Ar*H*), 7.75 (dddd, *J* = 6.0, 4.6, 2.8, 1.8 Hz, 2H, Ar*H*), 2.58 (td, *J* = 7.1, 1.6 Hz, 2H, CH_2), 1.68 (dtd, *J* = 14.7, 7.2, 2.1 Hz, 2H, CH_2), 1.08 (td, *J* = 7.6, 2.1 Hz, 3H, CH_3). **¹³C NMR** ($CDCl_3$, 100 MHz) 166.8, 134.6, 132.1, 131.3, 126.2, 115.5, 109.5, 50.4, 38.8, 22.3, 21.6, 13.4. **IR** ν 2960 (w), 2875 (w), 2172 (w), 1732 (m), 1654 (s), 1465 (w), 1439 (w), 1342 (w), 1296 (m), 1252 (m), 1109 (w), 1016 (w), 832 (m), 743 (s). **HRMS** (ESI) calcd for $C_{12}H_{12}IO_2^+$ $[M+H]^+$ 314.9877; found 314.9882.

(5-Chloropent-1-ynyl)-1,2-benziodoxol-3(1*H*)-one (2d)

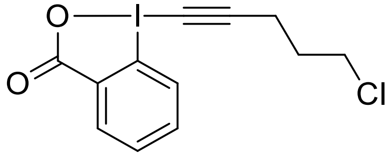

Following **GP1** on 15.2 mmol scale and using 5-chloro-1-pentynyl-1-boronic acid pinacol ester (4.85 g, 21.2 mmol, 1.40 equiv.), (5-chloropent-1-ynyl)-1,2-benziodoxol-3(1*H*)-one **2d** (3.76 g, 10.8 mmol, 71%) was obtained as a white solid. **Mp**: 138.5-141.7 °C. **R_f**: 0.15 (EtOAc 100%). **¹H NMR** (400 MHz, Chloroform-*d*) δ 8.41-8.34 (m, 1H, Ar*H*), 8.22-8.13 (m, 1H, Ar*H*), 7.82-7.68 (m, 2H, Ar*H*), 3.71 (t, *J* = 6.1 Hz, 2H, $ClCH_2CH_2$), 2.82 (t, *J* = 6.9 Hz, 2H, $CCCH_2CH_2$), 2.18-2.05 (m, 2H, $ClCH_2CH_2$). **¹³C NMR** ($CDCl_3$, 100 MHz) δ 166.8, 134.9, 132.5, 131.6, 131.6, 126.4, 115.8, 107.1, 43.4, 41.2, 30.7, 18.0. **IR** ν 2942 (w), 2866 (w), 2171 (w), 2091 (w), 1727 (w), 1617 (s), 1556 (w), 1441 (w), 1339 (m), 1213 (w), 1023 (w), 846 (w), 742 (s). The characterization data corresponded to the reported values.[5]

[6] *NB: the compound was isolated as an extremely viscous oil and retains organic solvent.*

2-Cyclopropylethynyl-1,2-benziodoxol-3(1*H*)-one (2f)

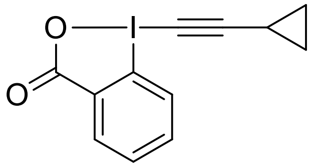

Following **GP1** on 25.8 mmol scale and using 5-chloro-1-pentynyl-1-boronic acid pinacol ester (4.85 g, 21.2 mmol, 1.40 equiv.), (cyclopropylethynyl)trimethylsilane (5.00 g, 36.2 mmol, 1.40 equiv.) 2-cyclopropylethynyl-1,2-benziodoxol-3(1*H*)-one **2f** (2.11 g, 6.76 mmol, 26%) was obtained as a white solid. **Mp**: 174.2–177.6 °C (Dec.). **R$_f$**: 0.46 (EtOAc:MeOH, 9:1). **^{1}H NMR** (400 MHz, Chloroform-*d*) δ 8.34 (dd, *J* = 7.0, 2.1 Hz, 1H, Ar*H*), 8.18-8.09 (m, 1H, Ar*H*), 7.81-7.63 (m, 2H, Ar*H*), 1.59 (tt, *J* = 8.2, 5.0 Hz, 1H, C*H*), 1.07-0.85 (m, 4H, CH_2CH_2). **^{13}C NMR** ($CDCl_3$, 100 MHz) δ 166.7, 134.7, 132.3, 131.7, 131.4, 126.2, 115.9, 113.3, 35.0, 9.8, 1.1. **IR** ν 3464 (w), 3077 (w), 3012 (w), 2238 (w), 2159 (m), 1607 (s), 1559 (m), 1438 (m), 1338 (m), 1298 (m), 833 (m), 744 (s), 691 (m). **HRMS** (ESI) calcd. for $C_{12}H_{10}IO_2^+$ $[M+H]^+$ 312.9720; found 312.9719. Data reported in literature.[1]

2-Cyclopentylethynyl-1,2-benziodoxol-3(1*H*)-one (2g)

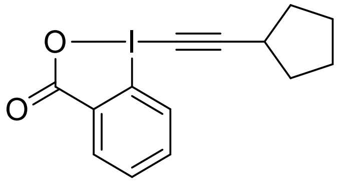

Following **GP1** on 4.00 mmol scale and using ethynylcyclopentane (0.649 g, 5.60 mmol, 1.40 equiv.) at 50 °C, 2-cyclopentylethynyl-1,2-benziodoxol-3(1*H*)-one **2g** (0.950 g, 2.79 mmol, 70%) was obtained as a white amorphous solid. **R$_f$**: 0.40 (DCM:MeOH 9:1). **^{1}H NMR** (400 MHz, Chloroform-*d*) δ 8.28 (t, *J* = 6.0 Hz, 1H, Ar*H*), 8.08 (t, *J* = 6.4 Hz, 1H, Ar*H*), 7.66 (tt, *J* = 13.4, 7.0 Hz, 2H, Ar*H*), 2.91 (q, *J* = 6.7 Hz, 1H, C*H*), 1.96 (dd, *J* = 13.6, 7.5 Hz, 2H, CH_2), 1.68 (d, *J* = 13.9 Hz, 4H, CH_2), 1.63 – 1.48 (m, 2H, CH_2). **^{13}C NMR** ($CDCl_3$, 100 MHz) δ 166.7, 134.4, 131.9, 131.5, 131.0, 126.1, 115.5, 113.7, 38.3, 33.5, 31.3, 24.9. **IR** ν 2960 (w), 2868 (w), 2165 (w), 1649 (s), 1610 (s), 1560 (m), 1439 (m), 1333 (m), 1295 (m), 1222 (w), 1008 (m), 833 (w), 752 (m). **HRMS** (ESI) calcd for $C_{14}H_{14}IO_2^+$ $[M+H]^+$ 341.0033; found 341.0036.

2-Cyclohexylethynyl-1,2-benziodoxol-3(1*H*)-one (2h)

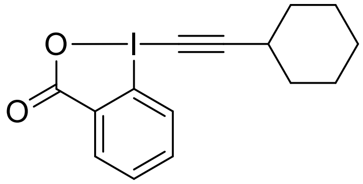

Following **GP1** on 4.00 mmol scale and using ethynylcyclohexane (0.732 g, 5.60 mmol, 1.40 equiv) 2-cyclohexylethynyl-1,2-benziodoxol-3(1*H*)-one **2h** (0.850 g, 2.40 mmol, 60%) was obtained as a white amorphous solid. **R$_f$**: 0.44 (DCM:MeOH 9:1). **^{1}H NMR** (400 MHz, Chloroform-*d*) δ 8.28 (t, *J* = 6.0 Hz, 1H, Ar*H*), 8.10 (t, *J* = 5.8 Hz, 1H, Ar*H*), 7.65 (dp, *J* = 12.9, 6.6 Hz, 2H, Ar*H*), 2.68 (h, *J* = 4.7, 4.2 Hz, 1H, C*H*), 1.82 (d, *J* = 12.5 Hz, 2H, CH_2), 1.67

(d, J = 10.7 Hz, 2H, CH_2), 1.46 (t, J = 10.4 Hz, 3H, CH_2), 1.29 (d, J = 10.2 Hz, 3H, CH_2). **^{13}C NMR** ($CDCl_3$, 100 MHz) δ 166.7, 134.3, 131.9, 131.4, 130.9, 126.1, 115.5, 113.4, 38.7, 31.9, 30.4, 25.3, 24.4. **IR** ν 2899 (m), 2877 (m), 1634 (s), 1579 (s), 1494 (w), 1307 (s), 1241 (w), 1049 (w), 980 (w), 876 (w), 817 (w). **HRMS** (ESI) calcd for $C_{15}H_{16}IO_2^+$ $[M+H]^+$ 355.0190; found 355.0192.

8-(Trimethylsilyl)octa-1,7-diyn-1-yl-1,2-benziodoxol-3(1*H*)-one (2e)

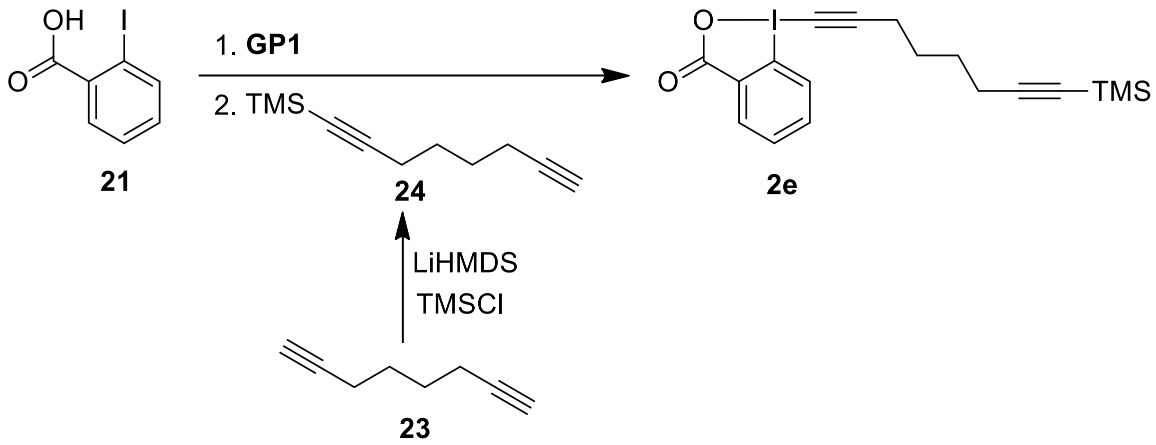

Following a slightly modified procedure,[1] to a solution of 1,7-octadiyne **23** (10.6 g, 100 mmol, 1.00 equiv) in dry THF (150 mL) was added at -78 °C under nitrogen 1 M lithium bis(trimethylsilyl)amide in THF (LiHMDS, 100 mL, 100 mmol, 1.00 equiv.). The solution was stirred at -78 °C for 30 minutes, after which trimethylsilyl chloride (TMSCl, 13.0 mL, 100 mmol, 1.00 equiv.) was added dropwise. The reaction was warmed to room temperature and stirred for 2 h. The reaction was cooled to 0 °C and quenched by adding water (10 mL). The mixture was diluted with 1 M HCl (200 mL) and extracted with diethyl ether (100 mL and 2 x 75 mL). The combined organic layers were washed with brine (200 mL), dried over $MgSO_4$, filtered and concentrated in vacuo. The crude product was purified by vacuum distillation using a 20 cm Vigreux column (oil bath set to 98 °C at 0.3 mbar) furnishing pure trimethyl(octa-1,7-diyn-1-yl)silane **24** (8.37 g, 46.9 mmol, 47%) as a colorless liquid. **R_f**: 0.2 (Pentane). **^{1}H NMR** ($CDCl_3$, 400 MHz) δ 2.28-2.17 (m, 4H), 1.93 (t, J = 2.7 Hz, 1H, CCH), 1.68-1.57 (m, 4H), 0.13 (s, 9H, TMS). **^{13}C NMR** ($CDCl_3$, 100 MHz) δ 107.0, 84.9, 84.2, 68.6, 27.7, 27.6, 19.5, 18.1, 0.3. **IR** ν 3309 (w), 2951 (w), 2175 (w), 1250 (m), 912 (w), 841 (s), 761 (m), 734 (m). Data reported in literature.[1]

Following a slightly modified procedure, 2-iodobenzoic acid **21** (8.43 g, 33.3 mmol, 1.00 equiv.), *para*-toluenesulfonic acid monohydrate ($TsOH.H_2O$, 6.40 g, 33.3 mmol, 1.00 equiv.) and *meta*-

chloroperoxybenzoic acid (*m*CPBA-70%, 9.04 g, 36.7 mmol, 1.10 equiv.) were dissolved in CH_2Cl_2 (60 mL) and 2,2,2-trifluoroethanol (60 mL). The mixture was stirred at room temperature under nitrogen for 1 h, after which trimethyl(octa-1,7-diyn-1-yl)silane **24** (8.32 g, 46.7 mmol, 1.40 equiv.) was added. The reaction mixture was stirred for 15 h at room temperature and then filtered and concentrated in vacuo. The resulting light being solid was dissolved in CH_2Cl_2 (500 mL) and under vigorous stirring, saturated solution of $NaHCO_3$ (500 mL) was added. The mixture was stirred for 1 h, the two layers were separated and the aqueous layer was extracted with additional portions of CH_2Cl_2 (3 x 150 mL). The combined organic layers were dried over $MgSO_4$, filtered and concentrated in vacuo. The crude product was purified by flash column chromatography using ethyl acetate to afford **2e** (4.20 g, 9.90 mmol, 30%) as a white solid. **Mp**: 152.3–155.6 °C. **R$_f$**: 0.59 (EtOAc:MeOH 9:1). **^{1}H NMR** ($CDCl_3$, 400 MHz) δ 8.37 (dd, *J* = 6.7, 2.3 Hz, 1H, Ar*H*), 8.17 (dd, *J* = 7.8, 1.5 Hz, 1H, Ar*H*), 7.82-7.66 (m, 2H, Ar*H*), 2.63 (t, *J* = 6.8 Hz, 2H,), 2.29 (t, *J* = 6.7 Hz, 2H), 1.83-1.62 (m, 4H), 0.13 (s, 9H, TMS). **^{13}C NMR** ($CDCl_3$, 100 MHz) δ 166.7, 134.8, 132.4, 131.7, 131.5, 126.3, 115.7, 109.1, 106.4, 85.4, 40.0, 27.7, 27.3, 20.2, 19.4, 0.3. **IR** ν 2955 (w), 2170 (w), 1647 (m), 1621 (s), 1439 (w), 1329 (m), 1296 (w), 1249 (m), 840 (s), 746 (s). **HRMS** (ESI) calcd. for $C_{18}H_{22}IO_2Si^+$ $[M+H]^+$ 425.0428; found 425.0433. Data reported in literature.[1]

3-(Benzyloxy)-3-methyl-but-1-yn-1-yl)-1,2-benziodoxol-3(1H)-one (2i)

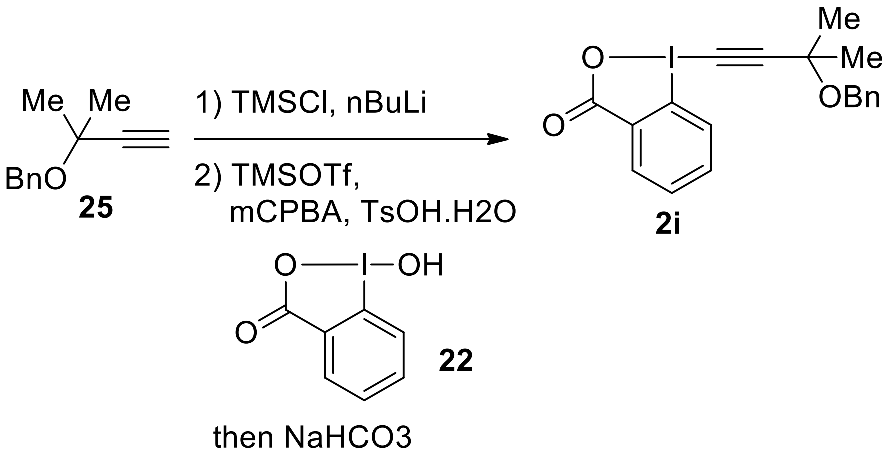

Following a reported procedure,[1] **25** (850 mg, 4.90 mmol, 1.00 equiv.) was dissolved in dry THF (10 mL). Next, ^{n}BuLi (2.5 M in hexane, 5.10 mL, 13.0 mmol, 2.60 equiv.) was added through syringe dropwise over 10 minutes and the reaction mixture was stirred for another 10 minutes to get a brownish-red solution. Next, TMSCl (0.700 mL, 5.50 mmol, 1.10 equiv.) was added dropwise to get a clear solution and the reaction mixture was stirred for 1.5 h at 0 °C. The resulting reaction

mixture was continuously stirred at room temperature for 2.5 h until a white solid precipitated. It was then diluted with hexane (30 mL), washed with water (3 x 20 mL), brine (20 mL), dried over $MgSO_4$, filtered and concentrated in vacuo. The crude product was purified by flash chromatography using EtOAc:Pentane 1:20 as mobile phase to afford (3-(benzyloxy)-3-methylbut-1-yn-1-yl)trimethylsilane (362 mg, 1.47 mmol, 33%), which was used directly in the next step.

Trimethylsilyltriflate (1.60 mL, 8.56 mmol, 1.10 equiv.) was added dropwise to a stirred solution of 2-iodosylbenzoic acid **22** (2.12 g, 7.99 mmol, 1.00 equiv.) in acetonitrile (40 mL) at 0 °C. After 15 minutes, (3-(benzyloxy)-3-methylbut-1-yn-1-yl)trimethylsilane (2.07 g, 8.89 mmol, 1.05 equiv.) was added dropwise, followed, after 30 min, by the addition of pyridine (6.00 mL). The mixture was stirred for 20 minutes. The solvent was then removed under reduced pressure and the crude oil was dissolved in CH_2Cl_2 (100 mL). The organic layer was washed with 0.5 M HCl (100 mL) and the aqueous layer was extracted with CH_2Cl_2 (100 mL). The organic layers were combined, washed with a saturated solution of $NaHCO_3$ (2 x 100 mL), brine (100 mL), dried over $MgSO_4$, filtered and the solvent was evaporated under reduced pressure. Recrystallization from hot EtOAc afforded **2i** (770 mg, 0.183 mmol, 23%) as a light yellow solid. **Mp**: 146.6-148.0 °C. **^{1}H NMR** ($CDCl_3$, 400 MHz): δ 8.39 (dd, *J* = 7.3, 1.8 Hz, 1H, Ar*H*), 8.11 (dd, *J* = 8.2, 1.1 Hz, 1H, Ar*H*), 7.78-7.62 (m, 2H, Ar*H*), 7.39-7.31 (m, 4H, Ar*H*), 7.31-7.27 (m, 1H, Ar*H*), 4.70 (s, 2H, ArC*H$_2$*), 1.69 (s, 6H, 2 x C*H$_3$*). **^{13}C NMR** ($CDCl_3$, 100 MHz) δ 166.6, 138.3, 135.0, 132.6, 131.7, 131.4, 128.6, 127.9, 127.6, 126.1, 115.8, 110.0, 71.9, 67.2, 45.5, 28.8. **IR** ν 2986 (w), 2868 (w), 2159 (w), 1618 (s), 1561 (m), 1446 (w), 1330 (m), 1299 (m), 1224 (m), 1159 (m), 1054 (m), 888 (w), 834 (m), 742 (s). Data reported in literature.[1]

1-[Phenylethynyl]-1,2-benziodoxol-3(1*H*)-one (2j)

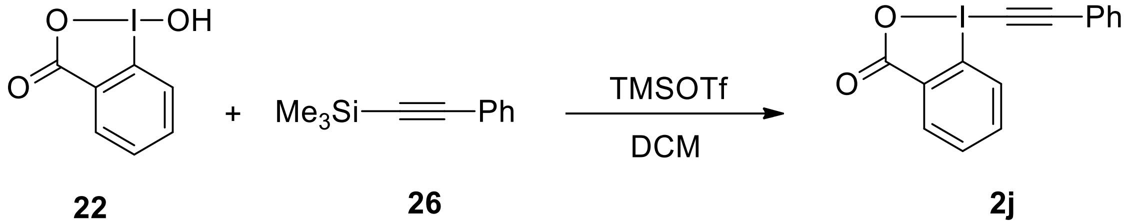

22 **26** **2j**

Following a reported procedure,[7] trimethylsilyl triflate (7.50 mL, 41.5 mmol, 1.10 equiv.) was added to a suspension of 2-iodosylbenzoic acid **22** (10.0 g, 37.7 mmol, 1.00 equiv.) in CH_2Cl_2 (100

[7] S. Nicolai, C. Piemontesi, J. Waser, *Angew. Chem. Int. Ed.* 2011, **50**, 4680.

mL) at room temperature. The resulting yellow mixture was stirred for 1 h, followed by the dropwise addition of trimethyl(phenylethynyl)silane **26** (8.10 mL, 41.5 mmol, 1.10 equiv.) (slightly exothermic). The resulting suspension was stirred for 6 h at room temperature, during this time a white solid was formed. A saturated solution of $NaHCO_3$ (100 mL) was then added and the mixture was stirred vigorously. The resulting suspension was filtered on a glass filter of porosity 4. The two layers of the mother liquors were separated and the organic layer was washed with saturated solution of $NaHCO_3$ (100 mL), dried over $MgSO_4$, filtered and evaporated under reduced pressure. The resulting mixture was combined with the solid obtained by filtration and boiled in CH_3CN (*ca* 300 mL). The mixture was cooled down, filtered and dried under high vacuum to afford **2j** (6.08 g, 17.4 mmol, 46 %) as a white solid. **Mp** (Dec.) 155.0–160.0 °C (lit 153-155°C). **^{1}H NMR** (400 MHz, $CDCl_3$) δ 8.46 (m, 1H, Ar*H*), 8.28 (m, 1H, Ar*H*), 7.80 (m, 2H, Ar*H*), 7.63 (m, 2H, Ar*H*), 7.48 (m, 3H, Ar*H*). **^{13}C NMR** (100 MHz, $CDCl_3$) δ 163.9, 134.9, 132.9, 132.5, 131.6, 131.3. 130.8, 128.8, 126.2, 120.5, 116.2, 106.6, 50.2. Data reported in literature.[7]

3. Optimization of the synthesis of N-vBX 5a.

Table 2.1: Solvent screening:

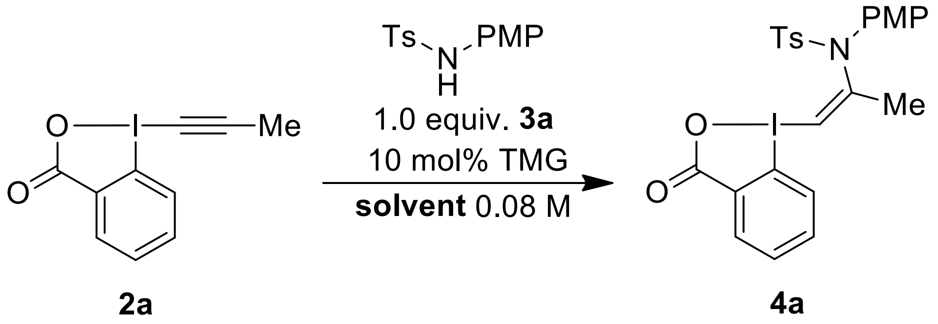

Entry	Solvent	Yield%[a] 4a
1	THF	22
2	EtOH	30
3	MeOH	23
4	DCM	28
**5**	TFE	30[b]
6	$CHCl_3$	25[b]
7	Toluene	18
8	DMF	-[b]
9	MeCN	22

a) Substrate **2a** (0.100 mmol), sulfonamide **3a** (0.100 mmol), TMG (10 mol%), and **solvent** (0.08 M) at 25 °C. NMR yield given, calculated using 38.0 µmol of 1,3,5-trimethoxybenzene as internal standard. b) Decomposition observed.

Table 2.2: Screening of the base:

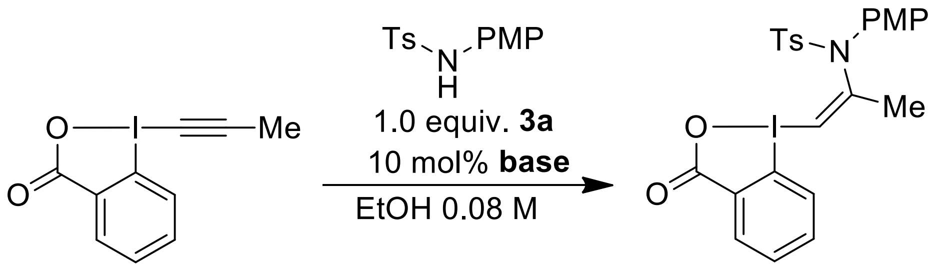

2a **4a**

Entry	Base	Yield% 4a[a]
1	TMG	30
2	TEA	43
3	Pyridine	38
4	NaOH	-[b]
5	KOH	-[b]
**6**	CsOH	54
7	$NaHCO_3$	82
8	$CsHCO_3$	83
9	$KHCO_3$	79
10	Na_2CO_3	84
11	CS_2CO_3	94(68)[c]
12	K_2CO_3	18

a) Substrate **2a** (0.100 mmol), sulfonamide **3a** (0.100 mmol), TMG (10 mol%), and **solvent** (0.08 M) at 25 °C. NMR yield given, calculated using 38.0 µmol of 1,3,5-trimethoxybenzene as internal standard. b) Decomposition observed. c) Isolated yield after column chromatography is given.

Table 2.3: screening of Base equivalents:

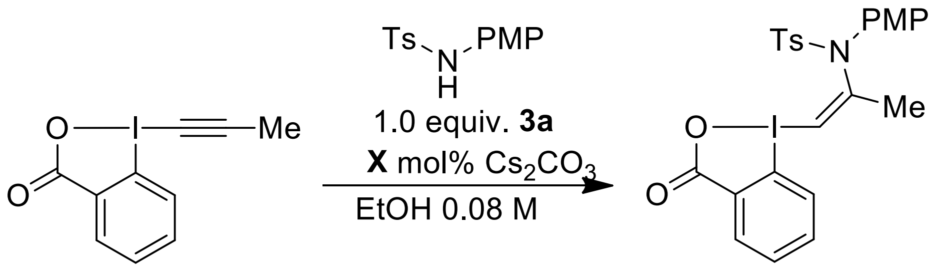

2a

4a

Entry	Base (equiv.)	Yield% 4a[a]
1	1.0	3.0[b]
2	0.50	25.1
3	0.10	94(68)[c]
4	0.25	46.4
5	0.05	39.1[d]
6	0.01	6.8[d]

a) Substrate **2a** (0.100 mmol), sulfonamide **3a** (0.100 mmol), TMG (10 mol%), and **solvent** (0.08 M) at 25 °C. NMR yield given, calculated using 38.0 µmol of 1,3,5-trimethoxybenzene as internal standard. b) Decomposition observed. c) Isolated yield after column chromatography is given. d) incomplete conversion, starting reagent present in the reaction mixture.

4. Scope of N-vBX and O-vBXs.

4.1 General Procedure GPX for the Synthesis N-vBX and O-vBX.

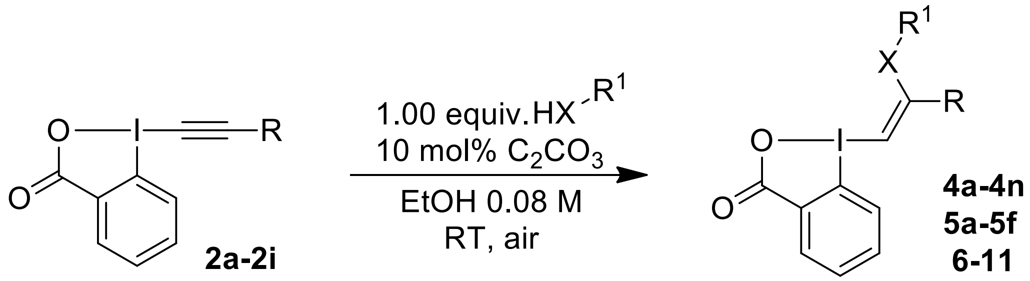

Note: prior to the reaction, the glassware requires to be carefully cleaned with aqua regia to remove all metal traces; the commercially available starting material were purified through a short plug of silica prior to being used.

GP2: In a glass vial, the correspondent sulfonamide or phenol (0.100 or 1.00 mmol, 1.00 equiv.) was dissolved in 12.5 mL of EtOH (0.08 M). Cs_2CO_3 (10 mol%, 10.0 µmol or 0.100 mmol) was added and the mixture stirred vigorously for 5'. Then the corresponding EBX **2a-2i** was added in one portion (0.100 or 1.00 mmol, 1.00 equiv.) and the reaction was left stirring for 12 hours if not specifically specified otherwise. The reaction was stopped, the EtOH removed under reduced pressure and the crude purified via column chromatography using DCM:MeOH (20:1) as eluent.

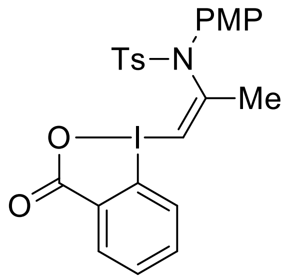

4a, 68%

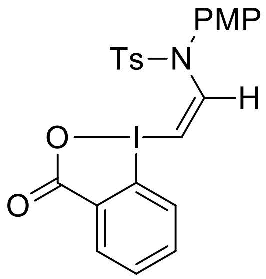

4b, 57%

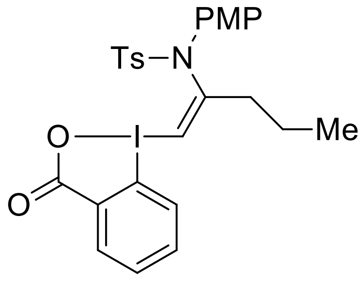

4c, 63%

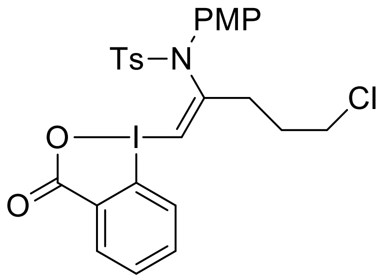

4d, 49%

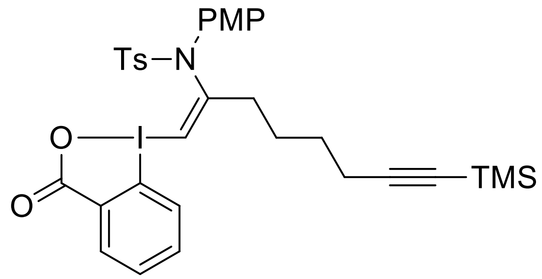

4e, 54%

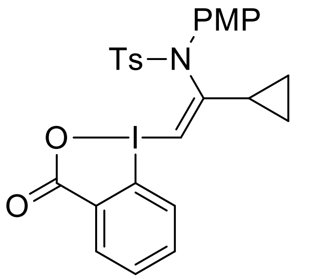

4f, 74%

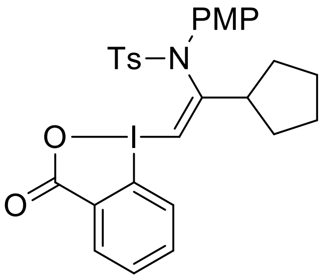

4g, 94%

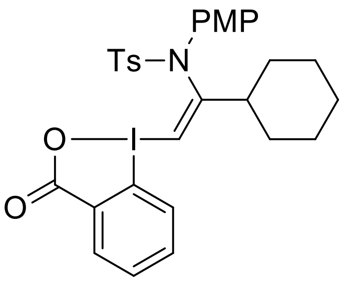

4h, 88%

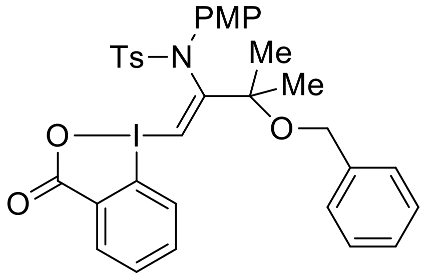

4i, 77%

(Z)-*N*-(1-Prop-1-en-2-yl)-*N*-(4-methoxyphenyl)-4-methylbenzenesulfonamide-1,2-benziodoxol-3-(1*H*)-one (4a)

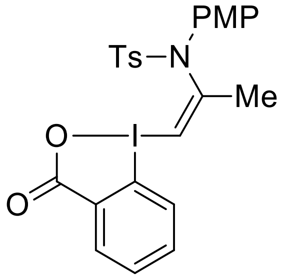

Starting from EBX **2a** (286 mg, 1.00 mmol), (Z)-*N*-(1-prop-1-en-2-yl)-*N*-(4-methoxyphenyl)-4-methylbenzenesulfonamide-1,2-benziodoxol-3-(1*H*)-one **4a** (383 mg, 0.680 mmol, 68% yield) was obtained, as a white solid. **Rf:** 0.30 (DCM:MeOH 9:1). **Mp**: 92.4 °C- 96.3 °C **^{1}H NMR** (400 MHz, Chloroform-*d*) δ 8.39 (dd, *J* = 7.4, 1.8 Hz, 1H, Ar*H*), 7.62 – 7.55 (m, 3H, Ar*H*), 7.51 (ddd, *J* = 9.0, 7.2, 1.9 Hz, 1H, Ar*H*), 7.35 (dd, *J* = 8.1, 1.1 Hz, 1H, Ar*H*), 7.31 – 7.27 (m, 2H, Ar*H*), 6.99 – 6.93 (m, 2H, Ar*H*), 6.80 (d, *J* = 1.4 Hz, 1H, vinyl*H*), 6.77 – 6.71 (m, 2H, Ar*H*), 3.73 (s, 3H, O*Me*), 2.43 (s, 3H, CH_3), 2.21 (s, 3H, CH_3). **^{13}C NMR** (101 MHz, Chloroform-*d*) δ 166.9, 160.0, 152.6, 145.2, 135.4, 133.8, 133.3, 132.78, 130.6, 130.3, 129.9, 129.8, 128.0, 126.1, 114.8, 114.6, 105.4, 55.5, 22.9, 21.6. **IR** ν 2970 (m), 1757 (w), 1654 (s), 1575 (s), 1481 (s), 1230 (m), 1195 (w), 1170 (w), 1081 (w). **HRMS** (ESI) calcd for $C_{24}H_{23}INO_5S^+$ [M+H]$^+$ 564.0336; found 564.0339. *The structure of the obtained regioisomer was confirmed by crystal structure, please see Section 7 for the details.*

(Z)-*N*-(1-Vin-2-yl)-*N*-(4-methoxyphenyl)-4-methylbenzenesulfonamide-1,2-benziodoxol-3-(1*H*)-one (4b)

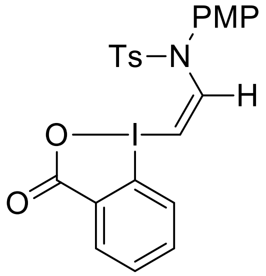

Starting from EBX **2b** (344 mg, 1.00 mmol), (Z)-*N*-(1-vin-2-yl)-*N*-(4-methoxyphenyl)-4-methylbenzenesulfonamide-1,2-benziodoxol-3-(1H)-one **4b** (316 mg, 0.575 mmol, 57% yield) was obtained, as a white amorphous solid. Rf: 0.40 (DCM:MeOH 9:1). **^{1}H NMR** (400 MHz, Methanol-*d*$_4$) δ 8.34 (d, *J* = 8.0 Hz, 1H, vinyl*H*), 8.15 (ddd, *J* = 6.7, 3.6, 2.0 Hz, 1H, Ar*H*), 7.67 – 7.63 (m, 3H, Ar*H*), 7.63 – 7.59 (m, 2H, Ar*H*), 7.44 – 7.36 (m, 2H, Ar*H*), 6.59 (s, 4H, Ar*H*), 5.77 (d, *J* = 8.0 Hz, 1H, vinyl*H*), 3.68 (s, 3H, O*Me*), 2.45 (s, 3H, CH_3). **^{13}C NMR** (101 MHz, Methanol-*d*$_4$) δ 169.7, 163.7, 147.2, 143.1, 135.3, 135.1, 133.8, 133.6, 133.2, 131.6, 131.2, 129.3, 128.1, 126.7, 116.9, 116.2, 72.5, 56.2, 21.6. **IR** ν 2963 (s), 2930 (s), 1728 (w), 1620 (m), 1426 (s), 1290 (m), 1111 (m), 1056 (s), 1016 (s), 748 (m). **HRMS** (ESI) $C_{23}H_{21}INO_5S$ [M+H]$^+$ 550.0107; found 550.0219. *The structure of the Z-regioisomer was assigned by NMR correlation to compound **4a**.*

(Z)-*N*-(1-Pent-1-en-2-yl)-*N*-(4-methoxyphenyl)-4-methylbenzenesulfonamide-1,2-benziodoxol-3-(1*H*)-one (4c)

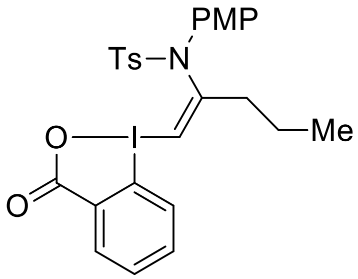

Starting from EBX **2c** (27.7 mg, 0.100 mmol), (Z)-*N*-(1-pent-1-en-2-yl)-*N*-(4-methoxyphenyl)-4-methylbenzenesulfonamide-1,2-benziodoxol-3-(1*H*)-one **4c** (37.4 mg, 63.0 µmol, 63% yield) was obtained, as a pale orangy oil. Rf: 0.34 (DCM:MeOH 9:1). **^{1}H NMR** (400 MHz, Chloroform-*d* + *AcOH 50.0 µL*) δ 8.46 (dd, *J* = 7.5, 1.8 Hz, 1H, Ar*H*), 7.67 – 7.59 (m, 1H, Ar*H*), 7.56 (m, 3H, Ar*H*), 7.37 – 7.31 (m, 1H, Ar*H*), 7.28 (m, 2H), 7.01 (d, *J* = 9.0 Hz, 2H, Ar*H*), 6.78 – 6.71 (m, 3H, 2H Ar*H* + 1H vinyl*H*), 3.75 (s, 3H, O*Me*), 2.42 (s, 3H, CH_3), 2.41 – 2.36 (m, 2H, CH_2), 1.59 (q, *J* = 7.5 Hz, 2H, CH_2), 0.96 (t, *J* = 7.3 Hz, 3H, CH_3). **^{13}C NMR** (101 MHz, Chloroform-*d* + *AcOH 50.0 µL*) δ 166.9, 159.9, 156.4, 145.1, 135.2, 133.9, 133.3, 132.8, 130.6, 130.3, 129.8, 129.7, 128.1, 125.9, 114.8, 114.7, 104.2, 55.4, 37.8, 21.6, 20.8, 13.5. **IR** ν 2941 (w), 1715 (s), 1521 (m), 1500 (m), 1395 (m), 1367 (m), 1282 (m), 1248 (m), 1174 (s), 1071 (m), 1042 (m), 977 (w), 861 (m). **HRMS** (ESI) calcd for $C_{26}H_{27}INO_5S^+$ [M+H]$^+$ 592.0649; found 592.0647. *The structure of the Z-regioisomer was assigned by NMR correlation to compound **4a**.*

(Z)-*N*-(5-Chloro-1-pent-1-en-2-yl)-*N*-(4-methoxyphenyl)-4-methylbenzenesulfonamide-1,2-benziodoxol-3-(1*H*)-one (4d)

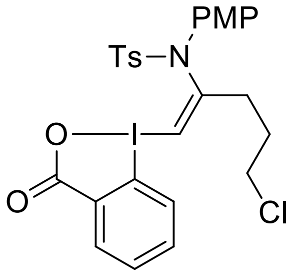

Starting from EBX **2d** (581 mg, 1.00 mmol), (Z)-*N*-(5-chloro-1-pent-1-en-2-yl)-*N*-(4-methoxyphenyl)-4-methylbenzenesulfonamide-1,2-benziodoxol-3-(1*H*)-one **4d** (307 mg, 0.491 mmol, 49% yield) was obtained, as a pale orange solid. **Rf:** 0.30 (DCM:MeOH 9:1). **^{1}H NMR** (400 MHz, Methylene Chloride-d_2 + AcOH *50.0 µL*) δ 8.34 (dd, *J* = 7.3, 2.0 Hz, 1H, Ar*H*), 7.68 – 7.55 (m, 4H, Ar*H*), 7.41 (dd, *J* = 8.0, 1.2 Hz, 1H, Ar*H*), 7.38 – 7.30 (m, 2H, Ar*H*), 7.00 (d, *J* = 9.0 Hz, 2H, Ar*H*), 6.89 (s, 1H, vinyl*H*), 6.77 (d, *J* = 9.0 Hz, 2H, Ar*H*), 3.75 (s, 3H, O*Me*), 3.57 (t, *J* = 6.2 Hz, 2H, CH_2), 2.59 – 2.50 (m, 2H, CH_2), 2.44 (s, 3H, CH_3), 1.99 (dq, *J* = 7.8, 6.2 Hz, 2H, CH_2). **^{13}C NMR** (101 MHz, Methylene Chloride-d_2) δ 167.3, 160.8, 155.3, 146.1, 135.7, 134.4, 134.0, 133.0, 131.3, 130.9, 130.6, 130.1, 128.6, 126.8, 115.5, 106.1, 56.1, 44.3, 33.6, 30.8, 21.9 (*1 Carbon aromatic signal non resolved*). **IR** ν 2971 (w), 1667 (w), 1478 (m), 1378 (s), 1275 (s), 1095 (m), 1048 (s), 881 (s). **HRMS** (ESI) calcd

for $C_{26}H_{26}ClINO_5S^+$ [M+H]$^+$ 626.0259; found 626.0264. *The structure of the Z-regioisomer was assigned by NMR correlation to compound **4a**.*

(Z)-*N*-(8-(Trimethylsilyl)oct-1-en-7-yn-2-yl)-*N*-(4-methoxyphenyl)-4-methylbenzene sulfonamide-1,2-benziodoxol-3-(1*H*)-one (4e)

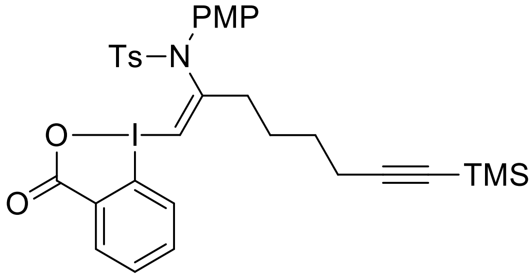

Starting from EBX **2e** (424 mg, 1.00 mmol), (Z)-*N*-(8-(trimethylsilyl)oct-1-en-7-yn-2-yl)-*N*-(4-methoxyphenyl)-4-methylbenzene sulfonamide-1,2-benziodoxol-3-(1*H*)-one **4e** (380 mg, 0.542 mmol, 54% yield) was obtained, as an orange oil. **Rf:** 0.44 (DCM:MeOH 9:1). **^{1}H NMR** (400 MHz, Chloroform-*d*) δ 8.37 (dd, *J* = 7.3, 2.0 Hz, 1H, Ar*H*), 7.58 – 7.49 (m, 4H, Ar*H*), 7.34 (dd, *J* = 7.9, 1.3 Hz, 1H, Ar*H*), 7.25 (d, *J* = 8.0 Hz, 2H, Ar*H*), 6.95 (d, *J* = 9.0 Hz, 2H, Ar*H*), 6.82 (s, 1H, vinyl*H*), 6.71 (d, *J* = 9.0 Hz, 2H, Ar*H*), 3.71 (s, 3H, O*Me*), 2.38 (m, *J* = 11.5 Hz, 4H, CH_3 + CH_2), 2.18 (t, *J* = 6.9 Hz, 2H, CH_2), 1.64 (dd, *J* = 10.3, 5.0 Hz, 2H, CH_2), 1.50 (q, *J* = 7.0 Hz, 2H, CH_2), 1.19 (t, *J* = 7.1 Hz, 1H, CH_2), 0.09 (s, 9H, Si(Me_3)$_3$). **^{13}C NMR** (101 MHz, Chloroform-*d*) δ 166.8, 159.9, 155.9, 145.1, 135.2, 133.8, 133.3, 132.7, 130.6, 130.1, 129.8, 129.6, 127.9, 125.9, 114.8, 114.7, 106.2, 104.8, 85.2, 55.4, 35.2, 27.6, 26.3, 21.5, 19.3, 0.0. **IR** ν 2951 (w), 2837 (w), 2172 (w), 1731 (w), 1624 (m), 1607 (m), 1506 (m), 1350 (m), 1245 (s), 1160 (s), 843 (s). **HRMS** (ESI/QTOF) Calcd for $C_{32}H_{37}INO_5SSi^+$ [M + H]$^+$ 702.1201; found 702.1206. *The structure of the Z-regioisomer was assigned by NMR correlation to compound **4a**.*

(Z)-*N*-(1-Vin-2-yl-2-cyclopropyl)-*N*-(4-methoxyphenyl)-4-methylbenzenesulfonamide-1,2-benziodoxol-3-(1*H*)-one (4f)

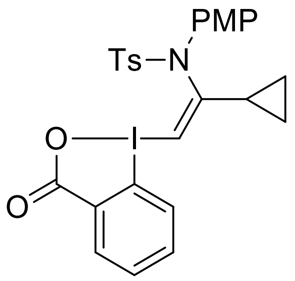

Starting from EBX **2f** (277 mg, 1.00 mmol), (Z)-*N*-(1-vin-2-yl-2-cyclopropyl)-*N*-(4-methoxyphenyl)-4-methylbenzenesulfonamide-1,2-benziodoxol-3-(1*H*)-one **4f** (435 mg, 0.738 mmol, 74% yield) was obtained, as a white amorphous solid. **Rf:** 0.40 (DCM:MeOH 9:1). **^{1}H NMR** (400 MHz, Chloroform-*d*) δ 8.39 – 8.24 (m, 1H, Ar*H*), 7.51 (m, 3H, Ar*H*), 7.46 – 7.41 (m, 1H, Ar*H*), 7.26 – 7.17 (m, 2H, Ar*H*), 7.10 (d, *J* = 8.0 Hz, 1H, Ar*H*), 6.97 (d, *J* = 8.5 Hz, 2H, Ar*H*), 6.66 (d, *J* = 8.6 Hz, 2H, Ar*H*), 6.57 (s, 1H,

vinyl*H*), 3.67 (s, 3H, O*Me*), 2.36 (s, 3H, *CH3*), 1.42 (td, *J* = 8.2, 4.2 Hz, 1H, *CH*), 0.88 – 0.78 (m, 2H, *CH2*), 0.64 (m, 2H, *CH2*). **^{13}C NMR** (101 MHz, Chloroform-*d*) δ 167.0, 159.8, 158.9, 145.0, 135.5, 133.8, 133.3, 132.8, 130.6, 130.3, 129.9, 129.3, 128.1, 125.7, 114.6, 114.2, 101.5, 55.4, 21.6, 16.9, 10.1. **IR** ν 2974 (w), 1614 (s), 1507 (s), 1348 (s), 1301 (m), 1242 (m), 1159 (s), 1089 (m), 1031 (m), 832 (m), 748 (m), 669 (s). **HRMS** (ESI) calcd for $C_{26}H_{25}INO_5S^+$ $[M+H]^+$ 590.0493; found 590.0505. *The structure of the Z-regioisomer was assigned by NMR correlation to compound **4a**.*

(Z)-*N*-(1-Vin-2-yl-2-cyclopentyl)-*N*-(4-methoxyphenyl)-4-methylbenzenesulfonamide-1,2-benziodoxol-3-(1*H*)-one (4g)

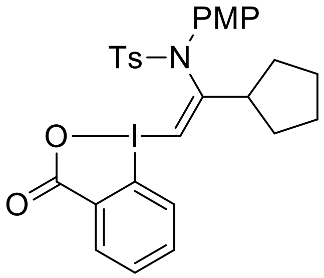

Starting from EBX **2g** (340 mg, 1.00 mmol), (Z)-*N*-(1-vin-2-yl-2-cyclopentyl)-*N*-(4-methoxyphenyl)-4-methylbenzenesulfonamide-1,2-benziodoxol-3-(1*H*)-one **4g** (581 mg, 0.940 mmol, 94% yield) was obtained, as an white resin. **Rf:** 0.40 (DCM:MeOH 9:1). **^{1}H NMR** (400 MHz, Chloroform-*d*) δ 8.39 – 8.30 (m, 1H, Ar*H*), 7.57 – 7.50 (m, 4H, Ar*H*), 7.32 – 7.27 (m, 1H, Ar*H*), 7.23 (d, *J* = 8.2 Hz, 2H, Ar*H*), 6.94 (d, *J* = 9.0 Hz, 2H, v), 6.90 (s, 1H, vinyl*H*), 6.71 (d, *J* = 9.0 Hz, 2H, Ar*H*), 3.70 (s, 3H, O*Me*), 2.55 – 2.43 (m, 1H, *CH*), 2.37 (s, 3H, *CH3*), 1.73 (qd, *J* = 12.8, 10.0, 6.2 Hz, 4H, *CH2*), 1.57 – 1.42 (m, 4H, *CH2*). **^{13}C NMR** (101 MHz, Chloroform-*d*) δ 166.9, 161.0, 159.7, 144.9, 135.4, 133.8, 133.3, 132.6, 130.5, 129.9, 129.8, 129.7, 127.9, 125.7, 114.8, 103.4, 55.3, 46.2, 34.2, 24.8, 21.5 (*1 Carbon aromatic signal non resolved*). **IR** ν 2953 (w), 1621 (s), 1583 (m), 1507 (s), 1439 (w), 1345 (s), 1257 (m), 1158 (s), 1088 (w), 1030 (w), 828 (m), 745 (s), 672 (s). **HRMS** (ESI) calcd for $C_{28}H_{29}INO_5S^+$ $[M+H]^+$ 618.0806; found 618.0806. *The structure of the Z-regioisomer was assigned by NMR correlation to compound **4a**.*

(Z)-*N*-(1-Vin-2-yl-2-cyclohexyl)-*N*-(4-methoxyphenyl)-4-methylbenzenesulfonamide-1,2-benziodoxol-3-(1*H*)-one (4h)

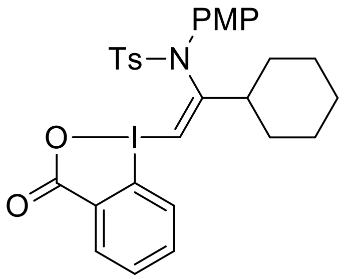

Starting from EBX **2h** (354 mg, 1.00 mmol), (Z)-*N*-(1-vin-2-yl-2-cyclohexyl)-*N*-(4-methoxyphenyl)-4-methylbenzenesulfonamide-1,2-benziodoxol-3-(1*H*)-one **4h** (553 mg, 0.876 mmol, 88% yield) was obtained, as a white resin. **Rf:** 0.40 (DCM:MeOH 9:1). **^{1}H NMR** (400 MHz, Chloroform-*d*) δ 8.42 (dd, *J* = 7.1, 2.2 Hz, 1H, Ar*H*), 7.59 (qd, *J* = 7.3, 1.6 Hz, 2H, Ar*H*), 7.53 (d, *J* = 8.2 Hz, 2H, Ar*H*), 7.35 (dd, *J* = 7.7, 1.5 Hz, 1H, Ar*H*), 7.24 (d, *J* = 8.1 Hz, 2H, Ar*H*), 6.99 (d, *J* = 8.9 Hz, 2H, Ar*H*), 6.78 – 6.70 (m, 3H, 2H Ar*H* + 1H vinyl*H*), 3.74 (s, 3H, O*Me*), 2.39 (s, 3H, CH_3), 2.13 (tt, *J* = 11.8, 3.0 Hz, 1H, *CH*), 1.86 (d, *J* = 12.5 Hz, 2H, CH_2), 1.82 – 1.71 (m, 2H, CH_2), 1.66 (d, *J* = 11.1 Hz, 1H, CH_2), 1.29 (qd, *J* = 12.5, 3.2 Hz, 2H, CH_2), 1.19 – 1.04 (m, 3H, CH_2). **^{13}C NMR** (101 MHz, Chloroform-*d*) δ 166.9, 161.9, 159.9, 145.0, 135.1, 133.9, 133.4, 132.9, 130.6, 130.2, 129.8, 129.7, 128.2, 125.7, 114.9, 103.6, 55.4, 44.6, 33.4, 26.2, 25.5, 21.6 (*1 Carbon aromatic signal non resolved*). **IR** ν 2939 (w), 2855 (w), 1624 (m), 1506 (m), 1439 (w), 1344 (m), 1260 (m), 1226 (w), 1157 (m), 1088 (w), 1035 (w), 831 (w), 750 (s), 669 (m). **HRMS** (ESI) calcd for $C_{29}H_{31}INO_5S^+$ $[M+H]^+$ 632.0962; found 632.0981. *The structure of the Z-regioisomer was assigned by NMR correlation to compound **4a**.*

(Z)-*N*-(3-(Benzyloxy)-3-methylbut-1-en-2-yl)-*N*-(4-methoxyphenyl)-4-methylbenzene sulfonamide-1,2-benziodoxol-3-(1*H*)-one (4i)

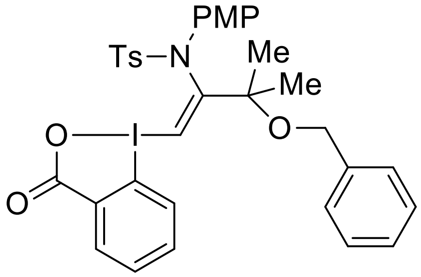

Starting from EBX **2i** (42.0 mg, 0.100 mmol), (Z)-*N*-(3-(benzyloxy)-3-methylbut-1-en-2-yl)-*N*-(4-methoxyphenyl)-4-methylbenzene sulfonamide-1,2-benziodoxol-3-(1*H*)-one **4i** (53.4 mg, 77.0 μmol, 77% yield) was obtained, as a white amorphous solid. **Rf:** 0.40 (DCM:MeOH 9:1). **^{1}H NMR** (400 MHz, Chloroform-*d* + DMF) δ 8.45 (dd, *J* = 7.5, 1.7 Hz, 1H, Ar*H*), 7.63 (td, *J* = 7.4, 0.9 Hz, 1H, Ar*H*), 7.56 – 7.50 (m, 3H, Ar*H*), 7.46 (ddd, *J* = 8.8, 7.2, 1.7 Hz, 1H, Ar*H*), 7.37 – 7.27 (m, 4H, 3H Ar*H* + 1H vinyl*H*), 7.25 – 7.22 (m, 2H, Ar*H*), 7.19 (d, *J* = 8.2 Hz, 2H, Ar*H*), 7.15 (d, *J* = 9.0 Hz, 2H, Ar*H*), 6.74 (d, *J* = 9.0 Hz, 2H, Ar*H*), 4.48 – 4.40 (m, 1H, CH_2O), 4.35 (d, *J* = 11.4 Hz, 1H, CH_2O), 3.75 (s, 3H, O*Me*), 2.39 (s, 3H, CH_3), 1.59 (s, 3H,

CH_3), 1.28 (s, 3H, *CH_3*). **^{13}C NMR** (101 MHz, Chloroform-*d*) δ 166.6, 160.1, 159.4, 145.3, 137.9, 135.6, 133.7, 133.1, 130.9, 130.6, 129.8, 128.9, 128.4, 128.1, 127.6, 126.9, 125.7, 115.1, 114.7, 109.9, 81.0, 65.1, 55.4, 29.7, 25.9, 21.6 (*1 Carbon aromatic signal non resolved*). **IR** ν 2992 (m), 2956 (w), 1627 (w), 1507 (m), 1438 (w), 1342 (w), 1266 (m), 1157 (m), 1067 (m), 1036 (m), 826 (m), 745 (s), 670 (s). **HRMS** (ESI) calcd for $C_{33}H_{33}INO_6S^+$ $[M+H]^+$ 698.1068; found 698.1086. *The structure of the Z-regioisomer was assigned by NMR correlation to compound **4a**.*

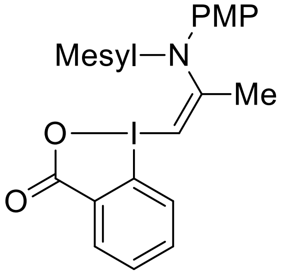

4k, 61%

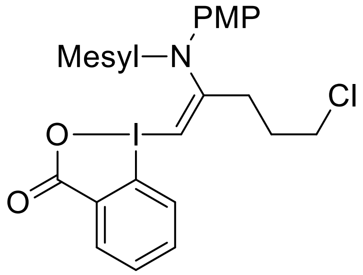

4l, 87%

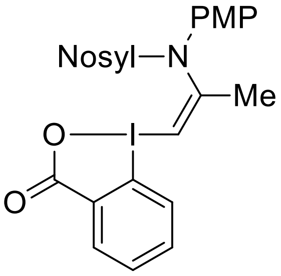

4m, 61%

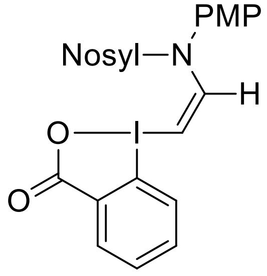

4n, 52%

(Z)-*N*-(1-Prop-1-en-2-yl)-N-(4-methoxyphenyl)-methylsulfonamide-1,2-benziodoxol-3-(1H)-one (4k)

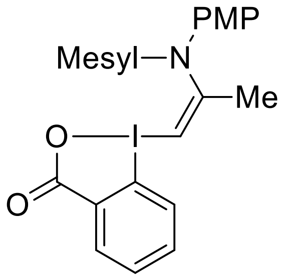

Starting from EBX **2a** (286 mg, 1.00 mmol), (Z)-*N*-(1-prop-1-en-2-yl)-*N*-(4-methoxyphenyl)-methylsulfonamide-1,2-benziodoxol-3-(1*H*)-one **4k** (296 mg, 0.606 mmol, 61% yield) was obtained, as a pale pink amorphous solid. **Rf:** 0.40 (DCM:MeOH 9:1). **^{1}H NMR** (400 MHz, Chloroform-*d*) δ 8.39 – 8.33 (m, 1H, Ar*H*), 7.61 – 7.52 (m, 2H, Ar*H*), 7.43 – 7.38 (m, 1H, Ar*H*), 7.28 (d, *J* = 9.0 Hz, 2H, Ar*H*), 6.88 (d, *J* = 1.4 Hz, 1H, vinyl*H*), 6.85 (d, *J* = 9.0 Hz, 2H, Ar*H*), 3.77 (s, 3H, O*Me*), 3.11 (s, 3H, *CH_3*), 2.43 (s, 3H,

CH_3). **^{13}C NMR** (101 MHz, Chloroform-*d*) δ 166.9, 160.0, 151.8, 133.5, 133.5, 132.6, 130.7, 129.6, 129.5, 126.3, 115.2, 114.3, 105.0, 55.5, 39.9, 23.2. **IR** ν 2965 (w), 2925 (w), 1604 (s), 1558 (w), 1506 (s), 1438 (w), 1337 (s), 1319 (m), 1249 (s), 1149 (s), 1030 (m), 965 (m), 832 (m), 747 (s). **HRMS** (ESI) $C_{18}H_{19}INO_5S^+$ $[M+H]^+$ 488.0023; found 488.0023. *The structure of the Z-regioisomer was assigned by NMR correlation to compound **4a**.*

(Z)-*N*-(5-Chloro-1-pent-1-en-2-yl)-N-(4-methoxyphenyl)-methylsulfonamide-1,2-benziodoxol -3-(1H)-one (4l)

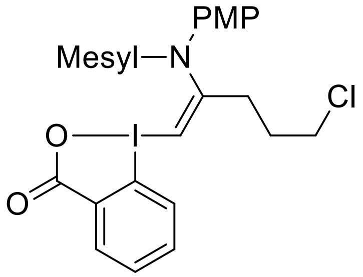

Starting from EBX **2d** (38.7 mg, 0.100 mmol), (Z)-*N*-(5-chloro-1-pent-1-en-2-yl)-*N*-(4-methoxyphenyl)-methylsulfonamide-1,2-benziodoxol -3-(1*H*)-one **4l** was obtained as white sticky solid (48.0 mg, 87.0 µmol, 87 %). **Rf:** 0.60 (DCM:MeOH 9:1). **^{1}H NMR** (400 MHz, CD_2Cl_2) δ 8.38 (ddd, *J* = 6.9, 2.3, 1.5 Hz, 1H, Ar*H*), 7.74 – 7.61 (m, 2H, Ar*H*), 7.53 – 7.47 (m, 1H, Ar*H*), 7.34 – 7.26 (m, 2H, Ar*H*), 6.94 – 6.85 (m, 3H, 2H Ar*H* + 1H vinyl*H*), 3.79 (s, 3H, O*Me*), 3.65 (t, *J* = 6.1 Hz, 2H, C*H_2*), 3.15 (s, 3H, C*H_3*), 2.65 (t, J = 7.4 Hz, 2H, C*H_2*), 2.12 (q, J = 13.0, 6.4 Hz, 2H, C*H_2*). **^{13}C NMR** (101 MHz, CD_2Cl_2) δ 166.8, 160.7, 153.9, 134.3, 133.9, 132.9, 131.3, 129.7, 129.61, 126.8, 115.8, 115.3, 106.2, 56.0, 44.1, 40.3, 33.4, 30.0. found 549.9957. **IR** ν 3662 (w), 3437 (w), 3049 (w), 2973 (m), 2901 (m), 2840 (w), 1607 (s), 1583 (m), 1558 (m), 1507 (s), 1440 (m), 1413 (w), 1337 (s), 1300 (m), 1247 (s), 1147 (s), 1131 (m), 1112 (m), 1069 (m), 1031 (m), 1004 (m), 971 (m), 828 (m), 804 (m), 744 (s), 685 (m), 650 (w), 605 (w). **HRMS** (ESI) calcd for $C_{20}H_{22}ClINO_5S^+$ $[M+H]^+$ 549.9946; found 549.9957. *The structure of the Z-regioisomer was assigned by NMR correlation to compound **4a**.*

(Z)-*N*-(1-Prop-1-en-2-yl)-*N*-(4-methoxyphenyl)-4-nitrobenzenesulfonamide-1,2-benziodoxol-3-(1*H*)-one (4m)

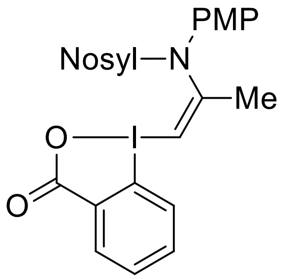

Starting from EBX **2a** (28.6 mg, 0.100 mmol), (Z)-*N*-(1-prop-1-en-2-yl)-*N*-(4-methoxyphenyl)-4-nitrobenzenesulfonamide-1,2-benziodoxol-3-(1*H*)-one **4m** (42.1 mg, 71.0 µmol, 71% yield) was obtained as a yellow amorphous solid. **Rf:** 0.28 (DCM:MeOH 9:1). **^{1}H NMR** (400 MHz, Chloroform-*d*) δ 8.42 (dt, *J* = 7.5, 1.5 Hz, 1H, Ar*H*), 8.39 – 8.31 (m, 2H, Ar*H*), 7.95 – 7.86 (m, 2H, Ar*H*), 7.60 (t, *J* = 7.3 Hz, 1H, Ar*H*), 7.52 (tt, *J* = 7.2, 1.5 Hz, 1H, Ar*H*), 7.32 (d, *J* = 8.1 Hz, 1H, Ar*H*), 7.02 – 6.94 (m, 2H, Ar*H*), 6.88 (s, 1H, vinyl*H*), 6.81 – 6.74 (m, 2H, Ar*H*), 3.76 (d, *J* = 1.2 Hz, 3H, O*Me*), 2.28 (s, 3H, CH_3). **^{13}C NMR** (101 MHz, Chloroform-*d*) δ 166.7, 160.4, 151.9, 150.6, 144.0, 133.6, 133.5, 132.9, 130.8, 130.2, 129.3, 128.9, 125.9, 124.5, 115.2, 114.7, 106.7, 55.6, 23.2. **IR** ν 3054 (w), 2934 (w), 2838 (w), 1604 (s), 1507 (s), 1437 (m), 1338 (s), 1249 (s), 1149 (s), 1031 (m), 965 (m), 831 (s), 733 (s). **HRMS** (ESI) calcd for calcd for $C_{23}H_{19}IN_2NaO_7S^+$ $[M+Na]^+$ 616.9850; found 616.9849. *The structure of the Z-regioisomer was assigned by NMR correlation to compound **4a**.*

(Z)-*N*-(1-Vin-2-yl)-N-(4-methoxyphenyl)-4-nitrobenzenesulfonamide-1,2-benziodoxol-3-(1*H*)-one (4n)

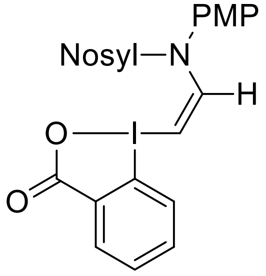

Starting from **2b** (30.8 mg, 0.100 mmol) and **xx** (34.4 mg, 0.100 mmol), the crude product was purified by preparative TLC (DCM/MeOH = 90:10) to afford (Z)-*N*-(1-vin-2-yl)-N-(4-methoxyphenyl)-4-nitrobenzene sulfonamide-1,2-benziodoxol-3-(1*H*)-one **4n** as yellow sticky solid (30 mg, 52 µmol, 52 %). **Rf:** 0.44 (EtOAc:MeOH 9:1). **^{1}H NMR** (400 MHz, Methanol-d4) δ 8.43 (d, *J* = 9.0 Hz, 2H, Ar*H*), 8.36 (d, *J* = 8.0 Hz, 1H, Ar*H*), 8.21 – 8.10 (m, 1H, Ar*H*), 8.00 (d, *J* = 8.6 Hz, 2H, Ar*H*), 7.72 – 7.59 (m, 3H, Ar*H*), 6.72 – 6.57 (m, 4H, Ar*H*), 5.92 (d, J = 8.1 Hz, 1H, vinyl*H*), 3.68 (s, 3H, OCH_3). **^{13}C NMR** (101 MHz, MeOD) δ 169.7, 163.4, 152.5, 143.5, 142.6, 135.2, 133.8, 133.6, 133.3, 131.7, 130.8, 128.3, 126.4, 125.8, 116.9, 116.5, 75.2, 56.3. **IR** ν 3384 (m), 2488 (m), 2233 (m), 2137 (w), 2071 (m), 1934 (w), 1652 (w), 1607 (m), 1581 (m), 1532 (m), 1509 (m), 1454 (w), 1405 (w), 1352 (m), 1309 (m), 1292 (w), 1255 (m), 1170 (m), 1147 (m), 1123 (m), 1088 (s), 1065 (m), 1024 (m), 973 (s), 832 (m), 738 (m),

663 (m), 622 (m). **HRMS** (ESI) calcd for $C_{22}H_{18}IN_2O_7S^+$ $[M+H]^+$ 580.9874; found 580.9885. *The structure of the Z-regioisomer was assigned by NMR correlation to compound **4a**.*

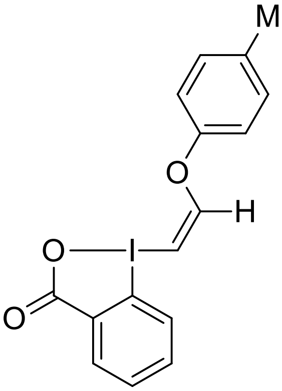

5a, 23%

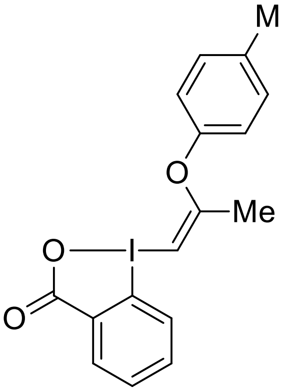

5b, 40%

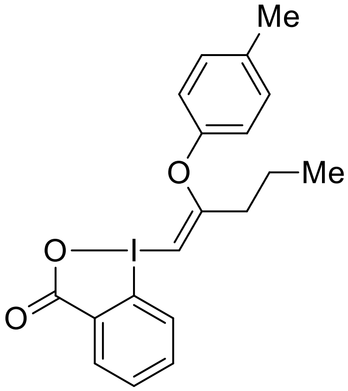

5c, 41%

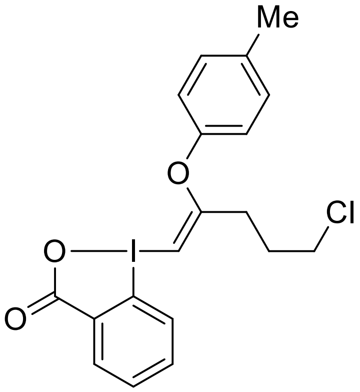

5d, 67%

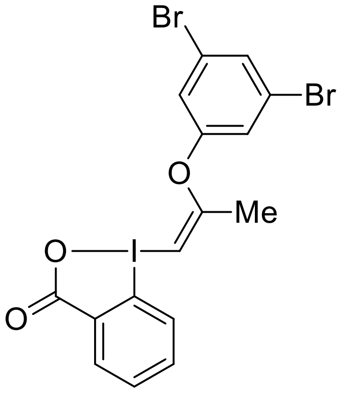

5e, 50%

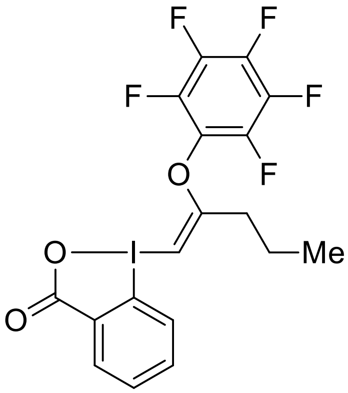

5f, 50%

(Z)-(1-Vinyl-2-oxy)-4-methylbenzene-1,2-benziodoxol-3-(1*H*)-one (5a)

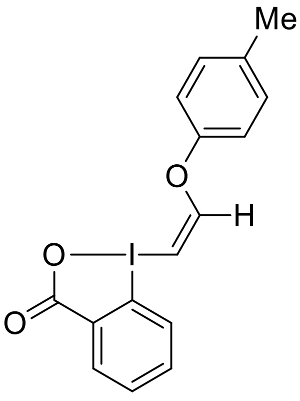

Starting from EBX **2b** (344 mg, 1.00 mmol), (Z)-(1-vinyl-2-oxy)-4-methylbenzene-1,2-benziodoxol-3-(1*H*)-one **5a** (87.5 mg, 0.230 mmol, 23% yield) was obtained as a white resin. **Rf:** 0.52 (DCM:MeOH 9:1). **^{1}H NMR** (400 MHz, Chloroform-*d*) δ 8.44 – 8.26 (m, 1H, Ar*H*), 7.64 – 7.57 (m, 1H, vinyl*H*), 7.53 – 7.47 (m, 2H, Ar*H*), 7.45 (d, *J* = 4.7 Hz, 1H, Ar*H*), 7.05 (d, *J* = 8.1 Hz, 2H, Ar*H*), 6.83 (d, *J* = 8.2 Hz, 2H, Ar*H*), 5.99 (d, *J* = 4.7 Hz, 1H, vinyl*H*), 2.25 (s, 3H, CH_3). **^{13}C NMR** (101 MHz,

Chloroform-*d*) δ 167.3, 156.6, 153.7, 135.1, 133.6, 133.5, 132.9, 130.5 (3 Carbon signals), 126.0, 117.2, 113.7, 78.9, 20.7. **IR** ν 2973 (w), 2878 (w), 1596 (m), 1507 (m), 1338 (w), 1226 (s), 1089 (m), 1048 (s), 879 (m), 736 (s). **HRMS** (ESI) calcd for $C_{16}H_{14}IO_3^+$ $[M+H]^+$ 380.9982; found 380.9984. *The structure of the Z-regioisomer was assigned by NMR correlation to compound **4a**.*

(Z)-(1-Prop-1-en-2-yl-2-oxy)-4-methylbenzene-1,2-benziodoxol-3-(1*H*)-one (5b)

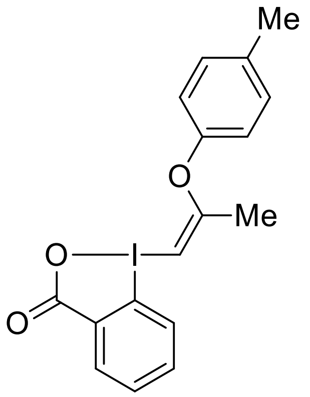

Starting from EBX **2a** (286 mg, 1.00 mmol), (Z)-(1-prop-1-en-2-yl-2-oxy)-4-methylbenzene-1,2-benziodoxol-3-(1*H*)-one **5b** (226 mg, 0.572 mmol, 57% yield) was obtained, as a white amorphous solid. **Rf:** 0.48 (DCM:MeOH 9:1). **^{1}H NMR** (400 MHz, Chloroform-*d*) δ 8.40 – 8.34 (m, 1H, Ar*H*), 7.64 – 7.52 (m, 3H, Ar*H*), 7.11 – 7.04 (m, 2H, Ar*H*), 6.77 (d, *J* = 8.5 Hz, 2H, Ar*H*), 5.80 (d, *J* = 1.1 Hz, 1H, vinyl*H*), 2.27 (s, 3H, *CH₃*), 2.18 (d, *J* = 0.9 Hz, 3H, *CH₃*). **^{13}C NMR** (101 MHz, Chloroform-*d*) δ 167.1, 166.8, 151.2, 135.4, 133.7, 133.1, 132.7, 130.4, 130.3, 125.3, 120.0, 113.7, 77.3 (1 Carbon signal overlaps with Chloroform-*d)*, 20.7, 19.2. **IR** ν 1603 (s), 1559 (w), 1505 (s), 1437 (w), 1357 (w), 1275 (w), 1211 (m), 837 (w). **HRMS** (ESI) calcd for $C_{17}H_{16}IO_3^+$ $[M+H]^+$ 395.0139; found 395.0148. *The structure of the Z-regioisomer was assigned by NMR correlation to compound **4a**.*

(Z)-(1-Pent-1-en-2-yl-2-oxy)-4-methylbenzene-1,2-benziodoxol-3-(1*H*)-one (5c)

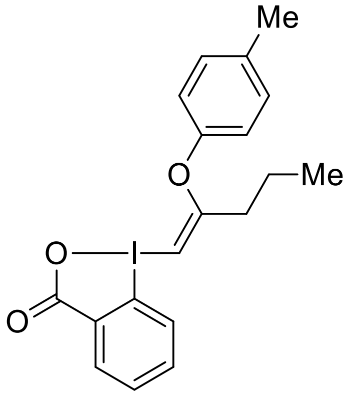

Starting from EBX **2c** (314 mg, 1.00 mmol), (Z)-(1-pent-1-en-2-yl-2-oxy)-4-methylbenzene-1,2-benziodoxol-3-(1*H*)-one **5c** (172 mg, 0.407 mmol, 41% yield) was obtained as a white amorphous solid. **Rf:** 0.50 (DCM:MeOH 9:1). **^{1}H NMR** (400 MHz, Chloroform-*d*) δ 8.49 – 8.31 (m, 1H, Ar*H*), 7.60 (m, 3H, Ar*H*), 7.09 (d, *J* = 7.9 Hz, 2H, Ar*H*), 6.78 (d, *J* = 7.9 Hz, 2H, Ar*H*), 5.85 (s, 1H, vinyl*H*), 2.48 (t, *J* = 7.6 Hz, 2H, *CH₂*), 2.29 (s, 3H, *CH₃*), 1.60 (q, *J* = 7.5 Hz, 2H, *CH₂*), 0.96 (t, *J* = 7.4 Hz, 3H, *CH₃*). **^{13}C NMR** (101 MHz, Chloroform-*d*) δ 170.4, 166.5, 151.5, 134.9, 133.8, 133.1, 132.9, 130.6, 130.5, 125.1, 119.1, 113.9, 80.1, 34.4, 20.7, 20.4, 13.5. **IR** ν 1601 (w), 1505 (w), 1430 (w), 1266 (m), 1205 (m), 1143 (w), 740

(s), 703 (m), 660 (m). **HRMS** (ESI) calcd for $C_{19}H_{20}IO_3^+$ $[M+H]^+$ 423.0452; found 423.0452. *The structure of the Z-regioisomer was assigned by NMR correlation to compound **4a**.*

(Z)-(5-Chloro-1-pent-1-en-2-yl-2-oxy)-4-methylbenzene-1,2-benziodoxol-3-(1*H*)-one (5d)

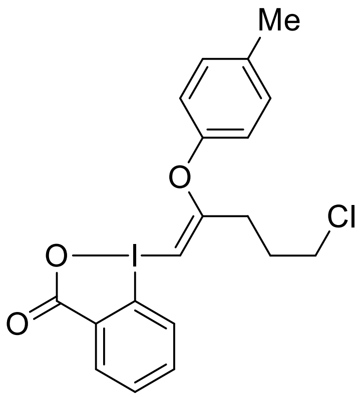

Starting from EBX **2d** (34.9 mg, 0.100 mmol), (Z)-(5-chloro-1-pent-1-en-2-yl-2-oxy)-4-methylbenzene-1,2-benziodoxol-3-(1*H*)-one **5d** (30.4 mg, 67.0 µmol, 67% yield – *92% purity*) was obtained, as a white amorphous solid. **Rf:** 0.44 (DCM:MeOH 9:1).[8] **^{1}H NMR** (400 MHz, Chloroform-*d*) δ 8.44 – 8.34 (m, 1H, Ar*H*), 7.64 – 7.56 (m, 3H, Ar*H*), 7.14 – 7.03 (m, 2H, Ar*H*), 6.79 (d, *J* = 8.5 Hz, 2H, Ar*H*), 6.02 (s, 1H, vinyl*H*), 3.55 (t, *J* = 6.2 Hz, 2H, CH_2), 2.79 – 2.65 (m, 2H, CH_2), 2.29 (s, 3H, CH_3), 2.01 (dq, *J* = 8.3, 6.4 Hz, 2H, CH_2). **^{13}C NMR** (101 MHz, Chloroform-*d*) δ 168.3, 166.7, 151.3, 135.1, 133.7, 133.3, 132.8, 130.6, 125.4, 118.9, 115.4, 114.0, 81.9, 43.4, 29.6, 29.3, 20.7. **IR** ν 2970 (w), 1603 (s), 1506 (s), 1351 (w), 1274 (m), 1211 (m), 1048 (w), 837 (w), 750 (m), 670 (s). **HRMS** (ESI/QTOF) calcd for $C_{19}H_{19}ClIO_3^+$ $[M + H]^+$ 457.0062; found 457.0070. *The structure of the Z-regioisomer was assigned by NMR correlation to compound **4a**.*

(Z)-(1-Prop-1-en-2-yl-2-oxy)-3,5-dibromobenzene-1,2-benziodoxol-3-(1*H*)-one (5e)

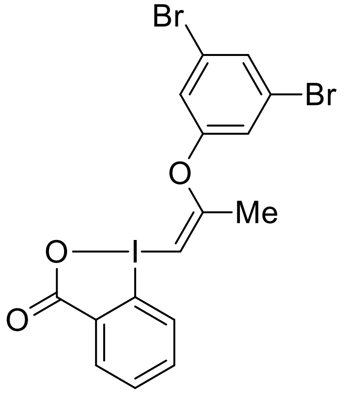

Starting from EBX **2a** (57.2 mg, 0.100 mmol), (Z)-(1-prop-1-en-2-yl-2-oxy)-3,5-dibromobenzene-1,2-benziodoxol-3-(1*H*)-one **5e** (27.1 mg, 50.0 µmol, 50% yield) was obtained, as a white amorphous solid. **Rf:** 0.38 (DCM:MeOH 9:1). **^{1}H NMR** (400 MHz, Methanol-d_4) δ 8.32 (dd, *J* = 7.5, 1.8 Hz, 1H, Ar*H*), 7.94 (dd, *J* = 8.1, 1.1 Hz, 1H, Ar*H*), 7.83 (td, *J* = 8.2, 7.7, 1.8 Hz, 1H, Ar*H*), 7.76 (td, *J* = 7.3, 1.1 Hz, 1H, Ar*H*), 7.63 (t, *J* = 1.6 Hz, 1H, Ar*H*), 7.34 (d, *J* = 1.6 Hz, 2H, Ar*H*), 6.40 (d, *J* = 1.0 Hz, 1H, vinyl*H*), 2.37 (s, 3H, CH_3). **^{13}C NMR** (101 MHz, Methanol-d_4) δ 170.4, 167.9, 156.1, 142.3, 136.0, 133.7, 133.1, 132.5, 132.0, 131.6, 129.2,

---

[8] *The proton and carbon spectra contain 8% of iodobenzoic acid as impurity.*

124.5, 123.8, 114.3, 80.4, 18.8. **IR** ν 2975 (w), 2882 (w), 1618 (w), 1576 (w), 1391 (w), 1320 (w), 1271 (w), 1126 (w), 1095 (m), 1050 (s), 881 (m), 741 (m). **HRMS** (ESI) calcd for $C_{16}H_{12}{}^{79}Br_2IO_3^+$ $[M+H]^+$ 536.8192; found 536.8194. *The structure of the Z-regioisomer was assigned by NMR correlation to compound* ***4a****.*

(Z)-(1-Pent-1-en-2-yl-2-oxy)-2,3,4,5-pentafluorobenzene-1,2-benziodoxol-3-(1*H*)-one (5f)

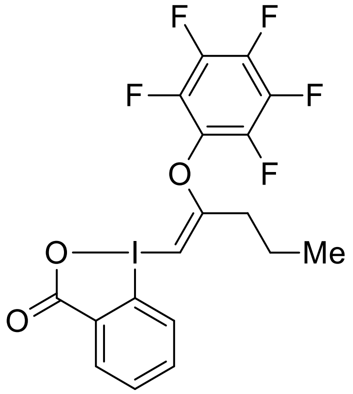

Starting from EBX **2c** (31.4 mg, 0.100 mmol), (Z)-(1-pent-1-en-2-yl-2-oxy)-2,3,4,5-pentafluorobenzene-1,2-benziodoxol-3-(1*H*)-one **5f** (45.3 mg, 91.0 µmol, 91% yield) was obtained, as a white amorphous solid. **Rf:** 0.42 (DCM:MeOH 9:1). **^{1}H NMR** (400 MHz, Chloroform-*d*) δ 8.47 – 8.37 (m, 1H, Ar*H*), 7.69 – 7.53 (m, 3H, Ar*H*), 6.03 (s, 1H, vinyl*H*), 2.41 (t, *J* = 7.6 Hz, 2H, CH_2), 1.66 (h, *J* = 7.4 Hz, 2H, CH_2), 1.03 (t, *J* = 7.3 Hz, 3H, CH_3). **^{13}C NMR** (101 MHz, Chloroform-*d*) δ 168.2, 166.8, 142.8 (dd, *J* = 12.5, 4.0 Hz), 140.6 – 139.9 (m), 139.7 – 139.1 (m), 137.2 – 136.7 (m), 133.6, 133.2, 132.9, 130.8, 127.9 (td, *J* = 14.3, 13.5, 4.0 Hz), 125.5, 113.9, 80.6, 33.5, 20.1, 13.4. **IR** ν 1695 (w), 1616 (w), 1517 (s), 1472 (w), 1341 (w), 1236 (w), 1159 (w), 997 (m), 670 (m). **HRMS** (ESI) calcd for $C_{18}H_{13}F_5IO_3^+$ $[M+H]^+$ 498.9824; found 498.9822. *The structure of the Z-regioisomer was assigned by NMR correlation to compound* ***4a****.*

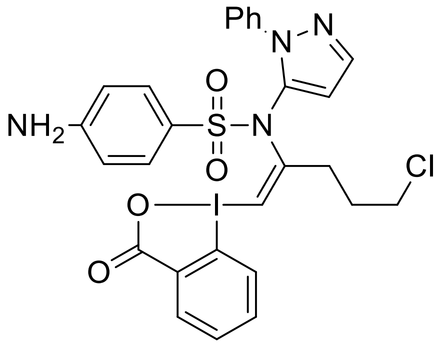

from Sulfaphenazole
6, 43%

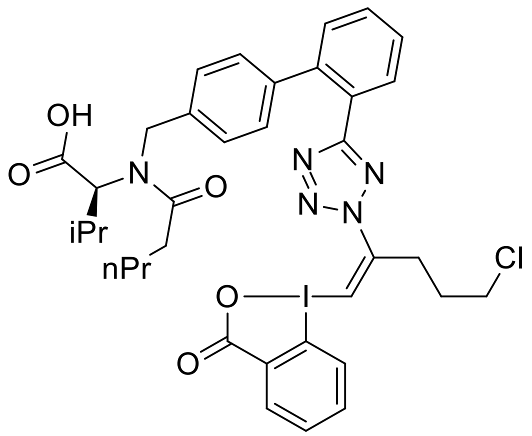

from Valsartan
11, 71%

(Z)-*N*-(5-Chloro-1-pent-1-en-2-yl)-*N*-Sulfaphenazole-1,2-benziodoxol-3-(1*H*)-one (6)

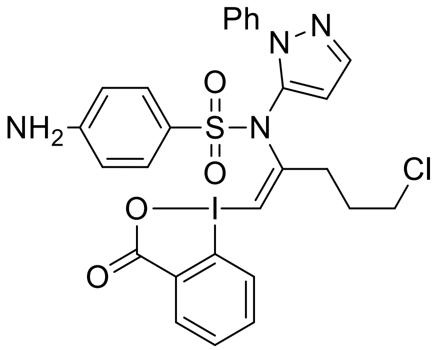

Starting from EBX **2d** (34.9 mg, 0.100 mmol) and commercially available Sulfaphenazole (31.4, 0.100 mmol, 1.00 equiv.), (Z)-*N*-(5-chloro-1-pent-1-en-2-yl)-*N*-Sulfaphenazole-1,2-benziodoxol-3-(1*H*)-one **6** (29.1 mg, 43.9 µmol, 43% yield) was obtained, as a pale orange amorphous solid. **Rf:** 0.25 (DCM:MeOH 9:1). **^{1}H NMR** (400 MHz, Methanol-d_4) δ 8.31 (dd, *J* = 7.5, 1.9 Hz, 1H, Ar*H*), 7.75 (dd, *J* = 7.3, 1.0 Hz, 1H, Ar*H*), 7.72 (s, 1H, Ar*H*), 7.71 – 7.66 (m, 1H, Ar*H*), 7.56 – 7.51 (m, 2H, Ar*H*), 7.30 (dd, *J* = 8.1, 1.1 Hz, 1H, Ar*H*), 7.26 – 7.22 (m, 3H, Ar*H*), 6.92 – 6.86 (m, 2H, Ar*H*), 6.84 – 6.77 (m, 3H, Ar*H*), 6.35 (d, *J* = 2.1 Hz, 1H, vinyl*H*), 3.55 (t, *J* = 6.2 Hz, 2H, CH_2), 2.49 – 2.41 (m, 2H, CH_2), 1.88 (p, *J* = 6.6 Hz, 2H, CH_2). **^{13}C NMR** (101 MHz, Methanol-d_4 + Chloroform-*d*) δ 170.1, 156.8, 153.7, 140.7, 138.9, 137.1, 135.1, 134.2, 133.3, 132.3, 131.7, 130.4, 129.9, 128.7, 128.1, 120.9, 116.5, 114.3, 107.2, 105.8, 44.4, 33.7, 31.6. **IR** ν 2976 (w), 2898 (w), 2863 (w), 1654 (w), 1616 (w), 1456 (w), 1379 (w), 1279 (w), 1086 (m), 1048 (s), 880 (m), 650 (s). **HRMS** (ESI) calcd for $C_{27}H_{25}ClIN_4O_4S^+$ $[M+H]^+$ 662.0372; found 663.0342. *The structure of the Z-regioisomer was assigned by NMR correlation to compound **4a**.*

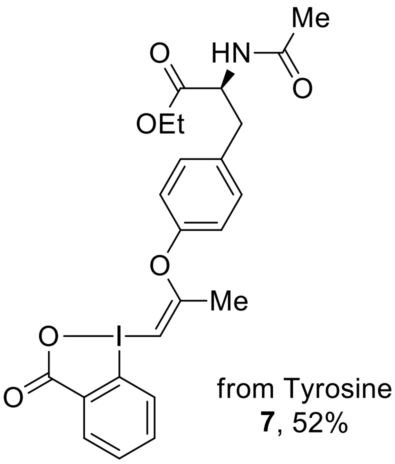

from Tyrosine
7, 52%

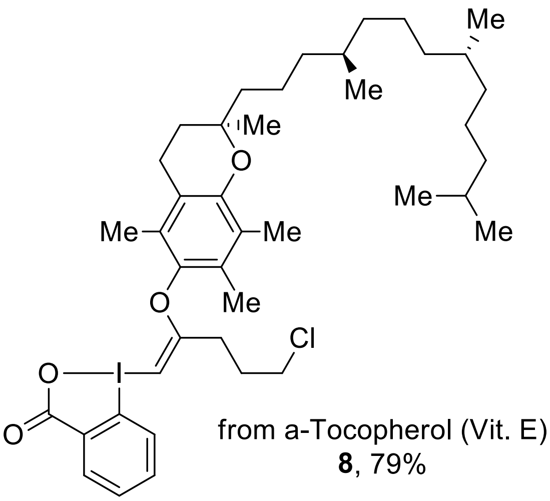

from a-Tocopherol (Vit. E)
8, 79%

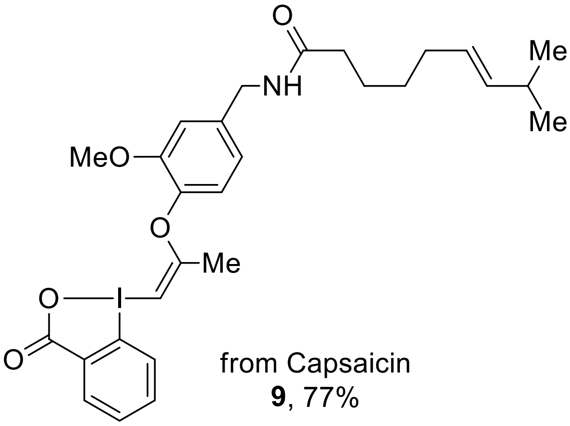

from Capsaicin
9, 77%

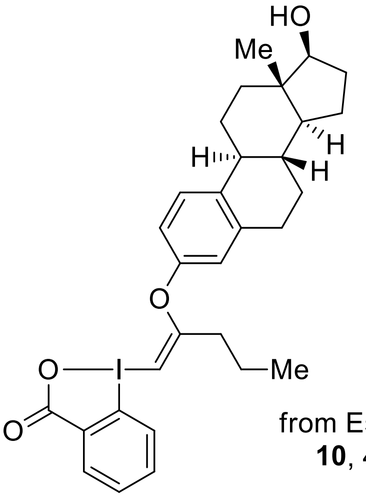

from Estradiol
10, 40%

(Z)-(1-Prop-1-en-2-yl)-2-Tyrosine-1,2-benziodoxol-3-(1*H*)-one (7)

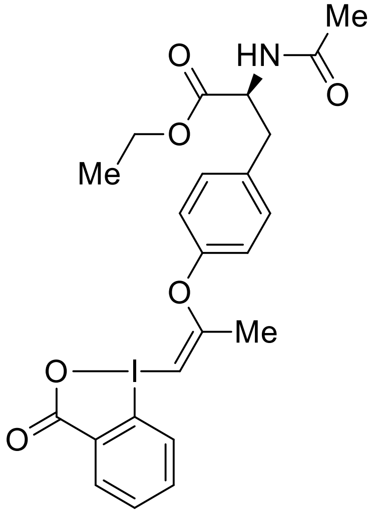

Starting from EBX **2a** (57.2 mg, 0.100 mmol) and commercially available (D)-Tyrosine monohydrate (26.9 mg, 0.100 mmol, 1.00 equiv.), (Z)-(1-prop-1-en-2-yl)-2-Tyrosine-1,2-benziodoxol-3-(1*H*)-one **7** (25.2 mg, 52.3 µmol, 52% yield) was obtained, as a pale yellow amorphous solid. **Rf:** 0.38 (DCM:MeOH 9:1). **^{1}H NMR** (400 MHz, Chloroform-*d*) *Rotamers ratio 6:1* δ 8.37 (ddd, *J* = 5.9, 2.8, 1.4 Hz, 1H, Ar*H major*), 8.32 – 8.27 (m, 1H, Ar*H minor*), 7.67 (d, *J* = 8.1 Hz, 1H, Ar*H minor*), 7.62 – 7.56 (m, 3H, Ar*H*, *major*), 7.53 – 7.49 (m, 1H *major*), 7.24 (m, 1H, Ar*H minor overlapping with* Chloroform-*d*), 7.14 – 7.03 (m, 2H + 2H, Ar*H major* + Ar*H minor*), 6.83 (d, *J* = 8.5 Hz, 2H, Ar*H major*), 6.48 (d, *J* = 7.8 Hz, 1H, N*H*Ac *minor*), 6.40 (d, *J* =

7.7 Hz, 1H, N*H*Ac *major*), 5.81 (s, 1H, vinyl*H major)*, 5.80 (s, 1H, vinyl*H minor*), 4.87 – 4.82 (m, 1H, *CH minor*), 4.78 (dt, *J* = 7.7, 6.0 Hz, 1H, *CH major*), 4.43 (q, *J* = 7.9 Hz, 2H, CH_2 *minor*), 4.13 (dtd, *J* = 14.3, 7.4, 1.8 Hz, 2H, CH_2 *major*), 3.20 – 3.01 (m, 2H + 2H, CH_2 *major* + CH_2 *minor*), 2.41 (s, 3H, CH_3 *minor*), 2.20 (s, 3H, CH_3 *major*), 1.96 (s, 3H, CH_3 *minor)*, 1.94 (s, 3H, CH_3 *major*), 1.21 (m, 3H + 3H, CH_3 *major* + CH_3 *minor*). **^{13}C NMR** (101 MHz, Chloroform-*d*) *only major rotamer expressed* δ 172.2, 171.4, 169.8, 169.2, 166.9, 166.6, 152.5, 152.0, 134.8, 133.9, 133.7, 133.6, 133.4, 133.2, 132.9, 132.8, 131.4, 130.9, 130.7, 130.6, 125.3, 125.0, 124.5, 121.2, 120.1, 114.2, 114.1, 113.8, 78.4, 74.3, 72.3, 61.5, 53.2, 37.3, 37.1, 23.1, 21.3, 21.1, 19.4, 14.1 (2 Carbon signals not expressed). **IR** ν 2362 (w), 1734 (w), 1599 (m), 1506 (w), 1438 (w), 1377 (w), 1266 (m), 1215 (w), 1127 (w), 1022 (w), 733 (s). **HRMS** (ESI) calcd for $C_{23}H_{25}INO_6^+$ $[M+H]^+$ 538.0721; found 538.0726. *The structure of the Z-regioisomer was assigned by NMR correlation to compound* ***4a****.*

(Z)-(5-Chloro-1-pent-1-en-2-yl)-2-α-Tocopherol-1,2-benziodoxol-3-(1*H*)-one (8)

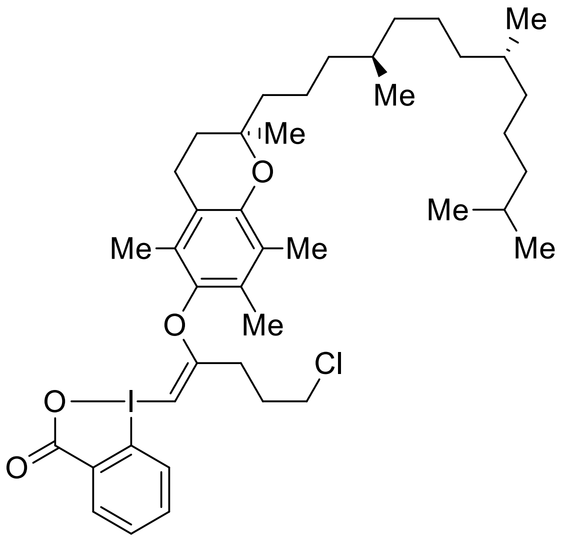

Starting from EBX **2d** (349 mg, 1.00 mmol) and commercially available α-Tocopherol (305 mg, 1.00 mmol), (Z)-(5-chloro-1-pent-1-en-2-yl)-2-α-Tocopherol-1,2-benziodoxol-3-(1*H*)-one **8** (517 mg, 0.791 mmol, 79% yield) was obtained, as a yellow oil. **Rf:** 0.55 (DCM:MeOH 9:1). **^{1}H NMR** (400 MHz, Chloroform-*d*) δ 8.39 – 8.27 (m, 1H, Ar*H*), 7.62 (td, *J* = 5.6, 4.5, 3.2 Hz, 1H, Ar*H*), 7.57 – 7.49 (m, 2H, Ar*H*), 5.68 (d, *J* = 11.8 Hz, 1H, vinyl*H*), 3.58 – 3.47 (m, 2H, CH_2), 2.43 (ddt, *J* = 22.6, 15.2, 7.0 Hz, 4H, CH_2 + *CH*), 2.02 (m, 5H, CH_2 + CH_3), 1.91 (s, 3H, CH_3), 1.87 (s, 3H, CH_3), 1.77 – 1.69 (m,

2H, *CH_2*), 1.55 – 1.41 (m, 3H, *CH_2 + CH_3*), 1.39 – 0.96 (m, 23H, *CH + CH_2 + CH_3*), 0.80 (ddd, *J* = 12.1, 6.7, 2.5 Hz, 12H, *CH_3*). **^{13}C NMR** (101 MHz, Chloroform-*d*) *major + minor diasteromers*[9] δ 170.4, 169.9, 166.6, 149.7, 149.6, 142.2, 142.1, 133.9, 133.9, 132.8, 132.6, 132.6, 130.3, 130.2, 129.75, 129.6, 126.9, 126.9, 125.4, 125.3, 125.2, 123.9, 123.7, 118.3, 118.1, 114.8, 113.8, 113.7, 75.3, 72.1, 71.3, 43.5, 43.5, 40.6, 39.2, 39.2, 39.1, 37.3, 37.3, 37.3, 37.3, 37.2, 37.1, 37.1, 32.6, 32.6, 32., 30.9, 30.8, 30.0, 29.5, 29.4, 29.3, 27.8, 27.8, 24.6, 24.6, 24.3, 24.3, 23.9, 22.9, 22.6, 22.5, 20.9, 20.8, 20.4, 20.4, 19.6, 19.5, 19.5, 13.0, 13.0, 12.2, 12.2, 11.7, 11.7 (*2 minor aromatic and 2 minor aliphatic carbon signals not expressed*). **IR** ν 2888 (s), 1569 (m), 1505 (w), 1495 (m), 1395 (m), 1369 (m), 1280 (m), 1204 (w), 1123 (w), 986 (w). **HRMS** (ESI) calcd for $C_{41}H_{61}ClIO_4^+$ $[M+H]^+$ 779.3298; found 779.3302. *The structure of the Z-regioisomer was assigned by NMR correlation to compound **4a**.*

(Z)-(1-Prop-1-en-2-yl)-2-Capsaicin-1,2-benziodoxol-3-(1*H*)-one (9)

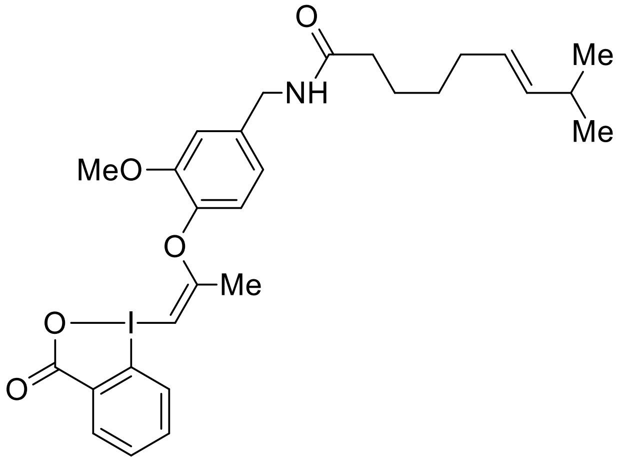

Starting from EBX **2a** (286 mg, 1.00 mmol) and commercially available Capsaicin (305 mg, 1.00 mmol), (Z)-(1-prop-1-en-2-yl)-2-Capsaicin-1,2-benziodoxol-3-(1*H*)-one **9** (455 mg, 0.769 mmol, 77% yield) was obtained, as a yellow oil. **Rf:** 0.60 (DCM:MeOH 9:1). **^{1}H NMR** (400 MHz, Chloroform-*d*) *major + minor rotamers ratio 12:1* δ 8.23 – 8.16 (m, 1H, Ar*H major*), 8.13 (t, *J* = 4.5 Hz, 1H, Ar*H minor*), 7.61 (q, *J* = 5.3, 4.3 Hz, 1H, Ar*H major*), 7.57 – 7.52 (m, 1H, Ar*H major*), 7.48 – 7.37 (m, 3H, *ArH major*), 7.35 – 7.29 (m, 2H, Ar*H minor*), 7.01 (s, 1H, Ar*H minor*), 6.93 (t, *J* = 7.4 Hz, 2H, Ar*H minor*), 6.86 – 6.78 (m, 1H, Ar*H major*), 6.72 – 6.64 (m, 2H, vinyl*H major + NH major*), 6.63 – 6.56 (m, 2H, Ar*H minor + NH minor*), 5.79 (s, 1H, vinyl*H minor*), 5.53 (s, 1H, vinyl*H major*), 5.33 – 5.19 (m, 2H, 1H vinyl*H major* + 2H vinyl*H minor*), 4.34 (d, *J* = 6.1 Hz, 2H, *CH_2NH minor*), 4.28 (d, *J* = 5.9 Hz, 2H, *CH_2NH major*), 3.87 – 3.81 (m, 3H, *OMe minor*), 3.64 – 3.51 (m, 3H, *OMe major*), 2.22 (td, *J* = 7.7, 2.9 Hz, 2H, *CH_2 major*), 2.17 – 2.11 (m, 4H, *CH_2 minor*), 2.08 (s, 3H, *CH_3 minor*), 1.98 (s, 3H, *CH_3 major*), 1.94 – 1.85 (m, 1H, *CH major*), 1.64 – 1.53 (m, 2H, *CH major*), 1.43 (m, 2H, *CH_2 minor*), 1.30 (dd, *J* = 9.2, 6.3 Hz, 1H, *CH_2 major*), 1.25 – 1.13 (m, 3H,

[9] In presence of acidic solvent, the ether opens and the tertiary carbocation isomerizes.

CH_2 major), 0.88 (d, *J* = 6.7 Hz, 4H, *CH_3 major + CH_3 minor + CH_3 minor*), 0.78 (d, *J* = 6.6 Hz, 3H, *CH_3 major*). **^{13}C NMR** (101 MHz, Chloroform-*d*) *major + minor rotamers* δ 175.4, 173.7, 173.6, 171.2, 168.8, 168.5, 167.3, 167.2, 150.9, 150.4, 140.5, 139.9, 139.1, 138.8, 137.7, 133.5, 133.4, 132.8, 132.4, 130.4, 130.1, 128.9, 128.6, 127.3, 126.5, 126.1, 124.9, 124.6, 122.3, 121.9, 120.5, 119.9, 114.4, 113.5, 112.4, 111.8, 72.9, 42.6, 55.5, 38.8, 38.7, 36.4, 36.2, 32.2, 30.8, 29.6, 29.3, 29.2, 27.8, 27.1, 27.0, 25.8, 25.3, 22.5, 22.5, 18.9. **IR** ν 2954 (w), 2926 (w), 1611 (s), 1508 (m), 1466 (w), 1360 (w), 1288 (s), 1211 (w), 1158 (w), 748 (w). **HRMS** (ESI) calcd for $C_{28}H_{35}INO_5^+$ $[M+H]^+$ 592.1554; found 592.1553. *The structure of the Z-regioisomer was assigned by NMR correlation to compound **4a**.*

(Z)-(1-Pent-1-en-2-yl)-2-Estradiol-1,2-benziodoxol-3-(1*H*)-one (10)

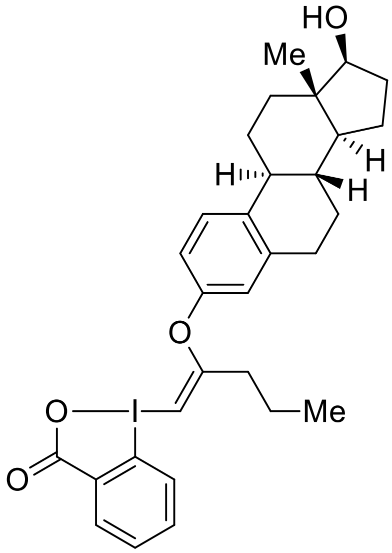

Starting from EBX **2c** (314 mg, 1.00 mmol) and commercially available Estradiol (272 mg, 1.00 mmol), (Z)-(1-pent-1-en-2-yl)-2-Estradiol-1,2-benziodoxol-3-(1*H*)-one **10** (615 mg, 0.789 mmol, 79% yield) was obtained, as a white amorphous solid. **Rf:** 0.40 (DCM:MeOH 9:1). **^{1}H NMR** (400 MHz, Chloroform-*d*) δ 8.42 – 8.34 (m, 1H, Ar*H*), 7.58 (m, 3H, Ar*H*), 7.16 (d, *J* = 8.5 Hz, 1H, Ar*H*), 6.63 (dd, *J* = 8.5, 2.7 Hz, 1H, Ar*H*), 6.56 (d, *J* = 2.6 Hz, 1H, Ar*H*), 5.90 (s, 1H, vinyl*H*), 3.70 (t, *J* = 8.5 Hz, 1H, *CH*), 2.81 – 2.66 (m, 2H, *CH_2*), 2.49 (t, *J* = 7.5 Hz, 2H, *CH_2*), 2.21 (dd, *J* = 13.4, 3.5 Hz, 1H, *CH*), 2.16 – 2.03 (m, 2H, *CH_2*), 1.92 (dt, *J* = 12.6, 3.3 Hz, 1H, *CH*), 1.82 (ddt, *J* = 11.8, 5.7, 2.6 Hz, 1H, *CH*), 1.62 (m, 3H, *CH_2*), 1.50 – 1.20 (m, 7H, *CH_2 + OH*), 1.12 (m, 1H, *CH_2*), 0.96 (t, *J* = 7.4 Hz, 3H, *CH_3*), 0.74 (s, 3H, *CH_3*). **^{13}C NMR** (101 MHz, Chloroform-*d*) δ 170.6, 166.6, 151.5, 138.9, 137.5, 133.7, 133.1, 132.9, 130.6, 126.9, 125.2, 119.0, 116.1, 113.8, 81.7, 80.0, 49.9, 43.9, 43.1, 38.4, 36.6, 34.47, 30.5, 29.5, 26.9, 26.1, 23.1, 20.5, 13.5, 11.0. **IR** ν 2931 (w), 2870 (w), 1600 (s), 1559 (w), 1492 (m), 1437 (w), 1345 (m), 1228 (m), 1153 (w), 1058 (w), 1006 (w), 831 (w), 738 (s). **HRMS** (ESI) calcd for $C_{30}H_{36}IO_4^+$ $[M+H]^+$ 587.1653; found 587.1655. *The structure of the Z-regioisomer was assigned by NMR correlation to compound **4a**.*

(Z)-*N*-(5-Chloro-1-pent-1-en-2-yl)-*N*-Valsartan-1,2-benziodoxol-3-(1*H*)-one (11)

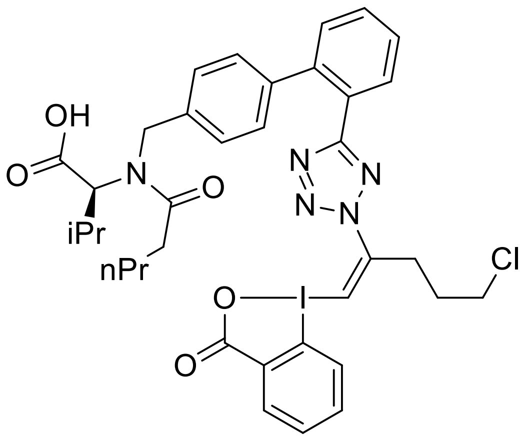

Starting from EBX **2d** (39.0 mg, 0.100 mmol) and commercially available Valsartan (44.0 mg, 0.100 mmol), (Z)-*N*-(5-chloro-1-pent-1-en-2-yl)-*N*-Valsartan-1,2-benziodoxol-3-(1*H*)-one **11** was obtained as a white sticky solid (56.0 mg, 71.0 μmol, 71%). *Mixture of rotamers observed* **^{1}H NMR** (400 MHz, Methanol-d_4) δ 8.26 (td, *J* = 7.0, 6.6, 1.9 Hz, 1H, Ar*H*), 7.97 – 7.84 (m, 2H, Ar*H*), 7.81 – 7.69 (m, 2H, Ar*H*), 7.65 (m, 1H, Ar*H*), 7.61 – 7.46 (m, 2H, Ar*H*), 7.34 (d, *J* = 5.7 Hz, 1H, Ar*H*), 7.26 (d, *J* = 8.2 Hz, 1H, Ar*H*), 7.23 – 7.14 (m, 2H, 1H Ar*H* + 1H vinyl*H*), 7.07 (d, *J* = 8.1 Hz, 1H, Ar*H*), 4.64 – 4.49 (m, 1H, ArCH_2N), 4.49 – 4.36 (m, 1H, ArCH_2N), 4.06 (d, J = 10.6 Hz, 1H, NC*H*COOH), 3.71 (td, J = 6.2, 2.4 Hz, 2H, C$H_2$$CH_2$$CH_2$Cl), 3.36 (dd, J = 9.5, 6.8 Hz, 2H, $CH_2$$CH_2CH_2$Cl), 2.62 – 2.37 (m, 1H, NCHC*H*(CH_3)$_2$), 2.35 – 2.06 (m, 4H, CH_2C$H_2$$CH_2$Cl, NCOC$H_2$$CH_2$$CH_2$$CH_3$), 1.62 – 1.51 (m, 1H, NCOCH_2C$H_2$$CH_2$$CH_3$), 1.48 – 1.38 (m, 1H, NCOCH_2C$H_2$$CH_2$$CH_3$), 1.38 – 1.27 (m, 1H, NCO$CH_2$$CH_2CH_2$$CH_3$), 1.17 (h, J = 7.5 Hz, 1H, NCO$CH_2$$CH_2CH_2$$CH_3$), 1.00 – 0.75 (m, 9H, C$H_3$). **^{13}C NMR** (101 MHz, Methanol-d_4) δ 177.0, 173.6, 166.0, 146.6, 143.3, 141.0, 138.2, 135.6, 134.5, 133.4, 132.3, 132.1, 131.9, 131.7, 131.4, 130.5, 129.9, 129.1, 128.4, 125.8, 116.7, 94.8, 65.0, 44.6, 34.6, 33.1, 31.3, 29.1, 28.5, 23.4, 20.6, 20.1, 19.3, 14.2. **IR** ν 2962 (m), 2876 (m), 2825 (w), 1727 (m), 1725 (m), 1645 (s), 1624 (s), 1616 (s), 1604 (s), 1557 (m), 1542 (m), 1530 (w), 1512 (w), 1473 (m), 1460 (m), 1436 (m), 1415 (m), 1376 (m), 1358 (m), 1329 (m), 1298 (m), 1269 (m), 1232 (m), 1206 (m), 1171 (m), 1132 (w), 1103 (m), 1007 (m), 999 (m), 980 (m), 943 (m), 914 (m), 896 (m), 828 (m), 812 (m), 787 (m), 760 (s), 746 (s), 715 (m), 703 (m), 687 (m), 674 (m), 650 (m). **HRMS** (ESI) calcd for $C_{36}H_{39}ClIN_5NaO_5^+$ $[M+Na]^+$ 806.1577; found 806.1579. *The structure of the Z-regioisomer was assigned by NMR correlation to compound* ***4a***.

5. Further functionalization employing N-vBX and O-vBX.

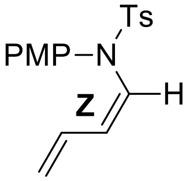

13, 65%

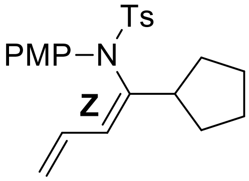

14, 80%

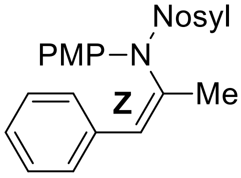

15, 88%

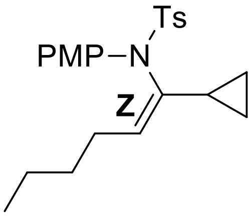

16, 52%

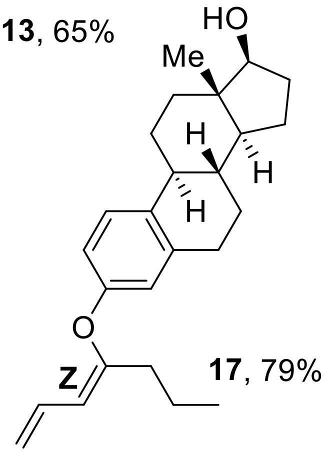

17, 79%

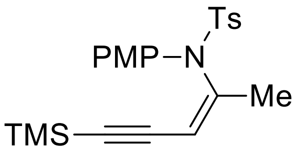

18, 44%

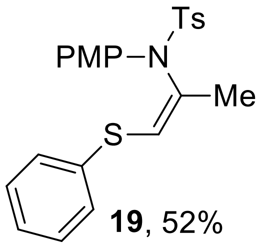

19, 52%

5.1 C-C bond formation.

General Procedure GP3 for the C-C bond formation.

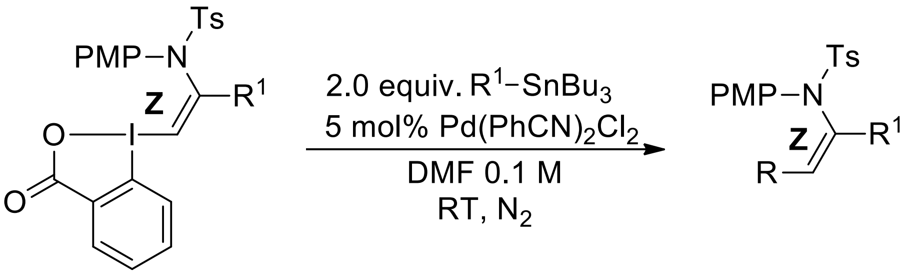

GP3: Following a reported procedure,[10] N-vBX (0.100 mmol, 1.00 equiv.), $Pd(PhCN)_2Cl_2$ (1.90 mg, 5.00 µmol, 5 mol%) and commercially available alkyl stannane (0.200 mmol, 2.00 equiv.) were added to a flame-dried vial. Upon sealing and oxygen removing under vacuum, the vial was backfilled with nitrogen (process repeated for three cycles). Dry DMF (1.00 mL, 0.1 M) was added under nitrogen atmosphere and the reaction was left stirring at room temperature for 10 hours. Then the reaction was stopped, EtOAc (10 mL) was added and the organic layer was washed with NaCl

[10] J. Wu, X. Deng, H. Hirao, N. Yoshikai, *J. Am. Chem. Soc.* 2016, **138**, 9105.

(3x30 mL). The solvent was removed under reduced pressure and the crude purified via column chromatography (gradient Pentane:EtOAc 20:1-10:1).

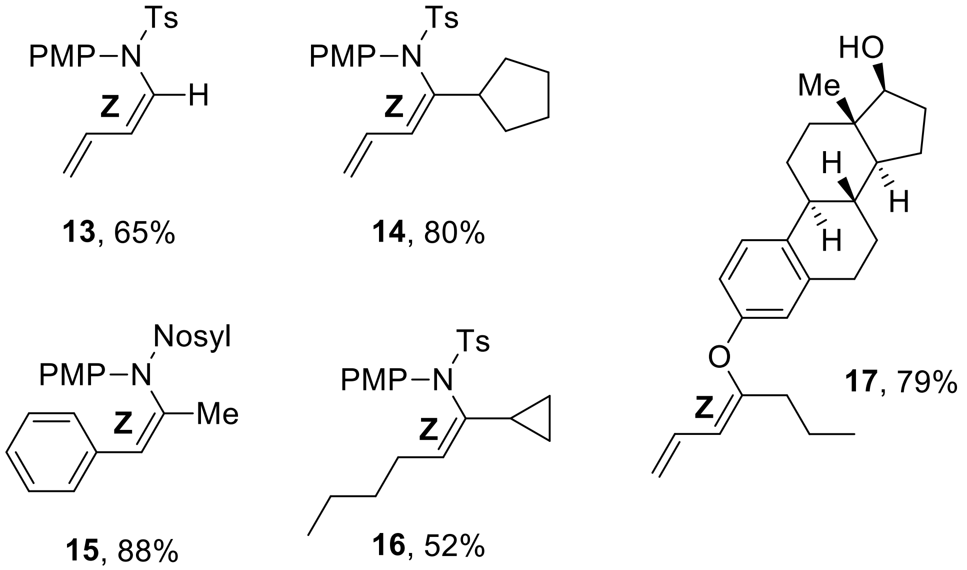

(Z)-*N*-(Buta-1,3-dien-1-yl)-N-(4-methoxyphenyl)-4-methylbenzenesulfonamide (13)

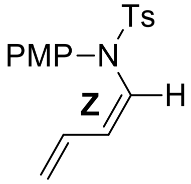

Starting from N-vBX **4b** (55.0 mg, 0.100 mmol) and commercially available tributyl(vinyl)stannane (60.5 µL, 0.200 mmol, 2.0 equiv.), (Z)-*N*-(buta-1,3-dien-1-yl)-N-(4-methoxyphenyl)-4-methylbenzenesulfonamide **13** (21.4 mg, 65.0 µmol, 65%) was obtained as a yellow oil. **Rf**: 0.13 (Pentane:EtOAc 20:1). **^{1}H NMR** (400 MHz, Chloroform-*d*) δ 7.53 – 7.45 (m, 2H, Ar*H*), 7.29 – 7.21 (m, 2H, Ar*H*), 7.03 – 6.95 (m, 2H, Ar*H*), 6.82 (dd, *J* = 8.8, 1.9 Hz, 2H, Ar*H*), 6.45 (d, *J* = 9.1 Hz, 1H, vinyl*H*), 5.73 (dt, *J* = 16.6, 10.7 Hz, 1H, vinyl*H*), 5.42 (dd, *J* = 11.3, 9.0 Hz, 1H, vinyl*H*), 4.98 (d, *J* = 16.8 Hz, 1H, vinyl*H*), 4.77 (d, *J* = 10.2 Hz, 1H, vinyl*H*), 3.81 (d, *J* = 1.9 Hz, 3H, O*Me*), 2.42 (s, 3H, C*H$_3$*).[11] **^{13}C NMR** (101 MHz, Chloroform-*d*) δ 159.2, 143.9, 134.8, 132.5, 130.1, 130.0, 129.5, 127.8, 126.3, 117.7, 116.8, 114.4, 55.4, 21.6. **IR** ν 2997 (w), 2953 (w), 2903 (w), 1636 (w), 1507 (w), 1441 (w), 1358 (w), 1252 (w), 1171 (m), 1123 (w), 1068 (w), 977 (w), 913 (s). **HRMS** (ESI) calcd for $C_{18}H_{19}NNaO_3S^+$ [M+Na]$^+$ 352.0978; found 352.0977.

[11] The compound was isolated in 92% purity, traces of PMPNHTs amide **3a** resulting of decomposition can be found in the ^{1}H NMR spectrum.

(Z)-*N*-(Cyclopentylbuta-1,3-dien-1-yl)-N-(4-methoxyphenyl)-4-methylbenzenesulfonamide (14)

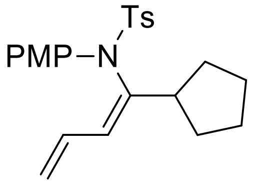

Starting from N-vBX **4g** (62.0 mg, 0.100 mmol) and commercially available tributyl(vinyl)stannane (61.0 µL, 0.200 mmol, 2.0 equiv.), (Z)-N-(Cyclopentylbuta-1,3-dien-1-yl)-N-(4-methoxyphenyl)-4-methylbenzenesulfonamide **14** (32.0 mg, 80.0 µmol, 80%) was obtained as a yellow oil.[12] *3:1 rotamers ratio*. **Rf**: 0.53 (Pentane:EtOAc 9:1). **^{1}H NMR** (400 MHz, Methylene Chloride-*d*$_2$) *3:1 rotamers ratio* δ 7.67 – 7.60 (m, 2H, *ArH minor*), 7.58 – 7.51 (m, 2H, *ArH major*), 7.45 – 7.36 (m, 2H, *ArH minor*), 7.24 (m, 4H, 2H *ArH major* + 2H *ArH minor*), 7.19 – 7.11 (m, 2H, *ArH major*), 6.84 – 6.76 (m, 4H, 2H *ArH major* + 2H *ArH minor*), 6.68 – 6.56 (m, 2H, 2H vinyl*H major* + 2H vinyl*H minor*), 6.18 (dd, *J* = 10.6, 1.0 Hz, 2H, 1H vinyl*H major* + 1H vinyl*H minor*), 5.32 – 5.25 (m, 2H, 1H vinyl*H major* + 1H vinyl*H minor*), 5.10 (dd, *J* = 10.2, 2.0 Hz, 2H, 1H vinyl*H major* + 1H vinyl*H minor*), 3.78 (d, *J* = 2.2 Hz, 6H, 3H *CH_3 major* + 3H *CH_3 minor*), 2.65 (h, *J* = 7.2 Hz, 1H, *CH minor*), 2.46 (td, *J* = 9.6, 3.7 Hz, 1H, *CH major*), 2.41 (s, 3H, *CH_3 major*), 2.39 (s, 3H, *CH_3 minor*), 1.93 (m, 2H, *CH_2 minor*), 1.79 (m, 4H, 1H *CH_2 major* + 3H *CH_2 minor*), 1.73 – 1.62 (m, 5H, 2H *CH_2 major* + 3H *CH_2 minor*), 1.59 – 1.36 (m, 5H, *CH_2 major*). **^{13}C NMR** *major* (101 MHz, Chloroform-*d*) δ 158.8, 144.8, 143.4, 137.8, 133.2, 130.4, 129.6, 129.3, 128.0, 126.4, 118.8, 114.3, 55.5, 46.1, 32.9, 31.2, 25.3, 24.9, 21.7. **^{13}C NMR** *minor* (101 MHz, Chloroform-*d*) δ 159.2, 144.7, 143.6, 137.3, 132.7, 130.8, 129.7, 129.1, 128.4, 126.2, 119.5, 114.1, 47.8, 43.00, 33.4, 32.5, 26.6, 24.9, 18.3. **IR** ν 2953 (m), 2873 (w), 2844 (w), 1603 (m), 1505 (s), 1458 (m), 1347 (m), 1298 (m), 1249 (m), 1163 (s), 1094 (m), 1035 (m), 912 (m), 814 (m), 732 (m), 710 (m), 671 (s). **HRMS** (ESI/QTOF) m/z: [M + H]$^+$ Calcd for $C_{23}H_{28}NO_3S^+$ 398.1784; Found 398.1780.

(Z)-*N*-(4-Methoxyphenyl)-4-nitro-*N*-(1-phenylprop-1-en-2-yl)benzenesulfonamide (15)

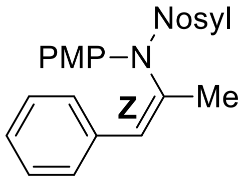

Starting from N-vBX **4m** (59.4 mg, 0.100 mmol), and commercially available tributyl(phenyl)stannane (65.5 µL, 0.200 mmol, 2.00 equiv.), (Z)-*N*-(4-methoxyphenyl)-4-nitro-*N*-(1-phenylprop-1-en-2-yl)benzenesulfonamide **15**

[12] The compound is highly unstable in acidic solvents.

(37.3 mg, 0.088 mmol, 88% yield) was obtained, as yellow sticky solid. **Rf:** 0.88 (DCM:MeOH 9:1). **^{1}H NMR** (400 MHz, Chloroform-*d*) δ 8.21 – 8.12 (m, 2H, Ar*H*), 7.73 – 7.64 (m, 2H, Ar*H*), 7.42 – 7.34 (m, 2H, Ar*H*), 7.26 (d, J = 3.4 Hz, 3H, Ar*H*), 6.99 – 6.89 (m, 2H, Ar*H*), 6.73 – 6.61 (m, 2H, Ar*H*), 6.40 (s, 1H, C*H*CN), 3.75 (s, 3H, OC*H3*), 2.17 (d, J = 1.3 Hz, 3H, C*H3*). **^{13}C NMR** (101 MHz, Chloroform-*d*) δ 159.1, 150.0, 145.7, 136.0, 135.0, 132.0, 130.4, 129.1, 128.9, 128.4 (*3 Carbon signals under this peak*), 127.9, 123.9, 114.3, 55.5, 23.3 **IR** ν 3652 (w), 3603 (w), 3556 (w), 3372 (w), 3278 (w), 3098 (w), 2987 (w), 2899 (w), 2611 (w), 2264 (s), 2118 (w), 1828 (w), 1777 (w), 1642 (m), 1546 (m), 1472 (m), 1407 (m), 1380 (m), 1317 (m), 1278 (m), 1198 (m), 1100 (s), 990 (m), 955 (m), 912 (m), 879 (m), 849 (m), 834 (s), 797 (m), 775 (m), 748 (m), 738 (m), 714 (m), 693 (m), 656 (m), 644 (w), 626 (w). **HRMS** (ESI) calcd for $C_{22}H_{20}N_2NaO_5S^+$ $[M+Na]^+$ 447.0985; found 447.0991.

(Z)-*N*-(1-cyclopropylhex-1-en-1-yl)-*N*-(4-methoxyphenyl)-4-methylbenzenesulfonamide (16)

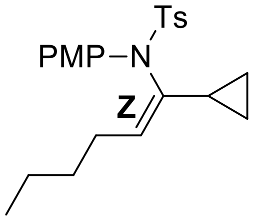

Starting from N-vBX **4f** (58.9 mg, 0.100 mmol) and commercially available tributyl(perfluoroethyl)stannane (65.3 µL, 0.200 mmol, 2.0 equiv.), (Z)-*N*-(1-cyclopropylhex-1-en-1-yl)-*N*-(4-methoxyphenyl)-4-methylbenzene sulfonamide **16** (20.7 mg, 52.0 µmol, 52%) was obtained as a white oil. **Rf**: 0.14 (Pentane:EtOAc 20:1). **^{1}H NMR** (400 MHz, Methanol-*d4*) δ 7.61 (d, *J* = 8.4 Hz, 2H, Ar*H*), 7.33 (d, *J* = 8.2 Hz, 2H, Ar*H*), 7.20 (d, *J* = 9.0 Hz, 2H, Ar*H*), 6.86 (d, *J* = 9.0 Hz, 2H, Ar*H*), 5.37 (td, *J* = 7.3, 1.1 Hz, 1H, vinyl*H*), 3.80 (s, 3H, O*Me*), 2.43 (s, 3H, *CH3*), 2.27 – 2.12 (m, 2H, *CH2*), 1.47 – 1.38 (m, 1H, *CH*), 1.29 (dt, *J* = 7.4, 3.1 Hz, 4H, *CH2*), 0.94 – 0.85 (m, 3H, *CH3*), 0.68 – 0.60 (m, 2H, *CH2*), 0.50 – 0.39 (m, 2H, *CH2*).[13] . **^{13}C NMR** (101 MHz, Chloroform-*d*) δ 158.6, 142.9, 140.1, 138.3, 133.1, 129.9, 129.2, 128.8, 127.8, 113.9, 55.4, 31.2, 27.9, 22.6, 21.5, 15.8, 13.9, 7.1. **IR** ν 2944 (s), 2888 (m), 1624 (w), 1484 (w), 1315 (m), 1275 (m), 1201 (w), 1145 (w), 1127 (w), 1113 (m), 1050 (m), 1008 (w). **HRMS** (ESI) calcd for $C_{23}H_{30}NO_3S^+$ $[M+H]^+$ 400.1868; found 399.1943.

[13] The compound was isolated in 92% purity, traces of PMPNHTs amide **3a** resulting of decomposition can be found in the ^{1}H NMR spectrum.

(8*R*,9*S*,13*S*,14*S*,17*S*)-3-((Z)-hepta-1,3-dien-4-yloxy)-13-methyl-7,8,9,11,12,13,14,15,16,17-decahydro-6*H*-cyclopenta[*a*]phenanthren-17-ol (17)

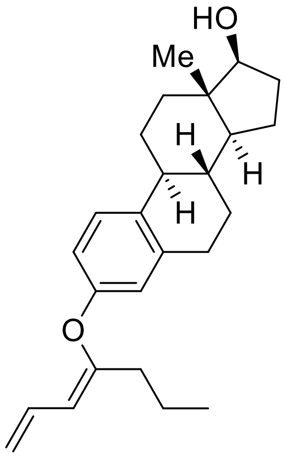

Starting from O-vBX **10** (58.7 mg, 0.100 mmol) and commercially available tributyl(vinyl)stannane (60.5 µL, 0.200 mmol, 2.0 equiv.), (8*R*,9*S*,13*S*,14*S*,17*S*)-3-((Z)-hepta-1,3-dien-4-yloxy)-13-methyl-7,8,9,11,12,13,14,15,16,17-decahydro-6*H*-cyclopenta[*a*]phenanthren-17-ol **17** (28.9 mg, 79.0 µmol, 79%) was obtained as a colorless oil. **Rf**: 0.20 (Pentane:EtOAc 10:1). **^{1}H NMR** (400 MHz, Chloroform-*d*) δ 7.20 (d, *J* = 8.5 Hz, 1H, Ar*H*), 6.72 (dd, *J* = 8.5, 2.7 Hz, 1H, Ar*H*), 6.65 (d, *J* = 2.7 Hz, 1H, Ar*H*), 6.58 (dt, *J* = 17.2, 10.5 Hz, 1H, vinyl*H*), 5.71 (d, *J* = 10.7 Hz, 1H, vinyl*H*), 5.14 (dd, *J* = 17.3, 2.1 Hz, 1H, vinyl*H*), 4.93 (dd, *J* = 10.4, 2.1 Hz, 1H, vinyl*H*), 3.73 (t, *J* = 8.5 Hz, 1H, vinyl*H*), 2.88 – 2.78 (m, 2H, CH_2), 2.31 (dq, *J* = 13.2, 3.8 Hz, 1H, C*H*), 2.24 – 2.08 (m, 4H, CH_2), 1.95 (dt, *J* = 12.6, 3.4 Hz, 1H, C*H*), 1.91 – 1.85 (m, 1H, C*H*), 1.71 (dddd, *J* = 12.3, 9.8, 6.9, 3.0 Hz, 1H, C*H*), 1.56 – 1.44 (m, 6H, CH_2 + *OH* + H_2O), 1.41 – 1.24 (m, 4H, CH_2), 1.23 – 1.15 (m, 1H, C*H*), 0.91 (t, *J* = 7.4 Hz, 3H, CH_3), 0.79 (s, 3H, CH_3). **^{13}C NMR** (101 MHz, Chloroform-*d*) δ 154.4, 153.0, 138.2, 133.9, 130.8, 126.4, 116.5, 116.4, 114.9, 113.8, 81.9, 50.1, 44.0, 43.2, 38.7, 36.7, 34.2, 30.6, 29.7, 27.2, 26.2, 23., 20., 13.6, 11.0. **IR** ν 3670 (w), 3416 (w), 2960 (s), 2916 (s), 1661 (m), 1610 (w), 1492 (s), 1416 (m), 1381 (w), 1313 (w), 1232 (s), 1132 (w), 1058 (s), 1006 (m), 902 (s), 734 (s). **HRMS** (ESI) calcd for $C_{25}H_{35}O_2^+$ [M+H]$^+$ 367.2632; found 367.2626.

Synthesis of (Z)-*N*-(1-methyl-4-(trimethylsilyl)but-1-en-3-yn-1-yl)-*N*-(4-methoxyphenyl) -4-methylbenzenesulfonamide (18)

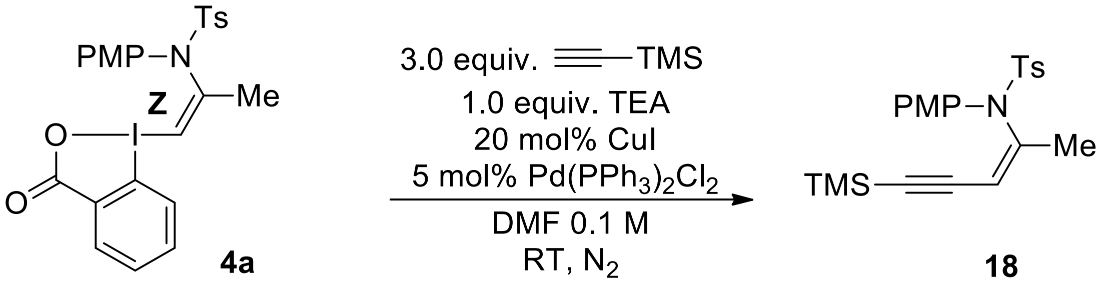

Following a reported procedure,[6] N-vBX **4a** (62.0 mg, 0.100 mmol, 1 equiv.), $Pd(PPh_3)_2Cl_2$ (3.51 mg, 5.00 µmol, 5 mol%), CuI (3.81 mg, 20.0 µmol, 20 mol%), TEA (14.0 µL, 0.100 mmol, 1.00 equiv.) and ethynyltrimethylsilane (43.0 µL, 0.300 mmol, 3.00 equiv.) were added to a flame-dried vial. Upon sealing and oxygen removing under vacuum, the vial was backfilled with nitrogen (process repeated for three cycles). Dry DMF (1.00 mL, 0.1 M) was added under nitrogen atmosphere and the reaction was left stirring at room temperature for 10 hours. Then the reaction was stopped, EtOAc (10 mL) was added and the organic layer was washed with NaCl (3x30 mL). The solvent was removed under reduced pressure and the crude product purified via column chromatography (gradient Pentane:EtOAc 20:1-10:1). (Z)-*N*-(1-cyclopentyl-4-(trimethylsilyl)but-1-en-3-yn-1-yl)-*N*-(4-methoxyphenyl) -4-methylbenzenesulfonamide **18** (18.0 mg, 44.0 µmol, 44%) was obtained as a yellow oil. *6:1 Z:E ratio*. **Rf**: 0.25 (Pentane:EtOAc 20:1). **^{1}H NMR** *major Z*: (400 MHz, Methanol-*d*$_4$) δ 7.64 (d, *J* = 8.4 Hz, 2H, Ar*H*), 7.33 (d, *J* = 8.1 Hz, 2H, Ar*H*), 7.31 – 7.25 (m, 2H, Ar*H*), 6.83 (d, *J* = 9.0 Hz, 2H, Ar*H*), 5.61 (d, *J* = 1.4 Hz, 1H, vinyl*H*), 3.79 (s, 3H, O*Me*), 2.43 (s, 3H, CH_3), 2.16 (d, *J* = 1.3 Hz, 3H, CH_3), 0.16 (s, 9H, Si(CH_3)$_3$). *Minor E*: (400 MHz, Methanol-*d*$_4$) δ 7.63 (m, 2H, Ar*H*), 7.41 (d, *J* = 8.2 Hz, 2H, Ar*H*), 7.14 – 7.10 (m, 2H, Ar*H*), 6.96 – 6.92 (m, 2H, Ar*H*), 5.53 (d, *J* = 1.0 Hz, 1H, *CH*), 3.83 (s, 3H, O*Me*), 2.46 (s, 3H, CH_3), 1.93 (d, *J* = 0.9 Hz, 3H, CH_3), 0.17 (s, 9H, Si(CH_3)$_3$). **^{13}C NMR** *major:* (101 MHz, Chloroform-*d*) δ 159.1, 148.4, 143.3, 137.7, 132.4, 130.8, 129.3, 128.1, 127.7, 113.9, 110.5, 100.9, 55.4, 22.9, 21.6, -0.3. **IR** ν 3367 (w), 2978 (w), 2934 (w), 1713 (m), 1507 (m), 1448 (w), 1367 (m), 1248 (m), 1166 (s), 1094 (w), 1033 (w), 976 (w), 847 (m), 794 (w), 674 (w). **HRMS** (ESI) calcd for $C_{22}H_{27}NO_3SSi$ 414.1481; 414.1560.

5.2 C-Heteroatom bond formation.

(Z)-*N*-(1-Cyclohexyl-2-(phenylthio)vinyl)-*N*-(4-methoxyphenyl)-4-methylbenzene-sulfonamide (19)

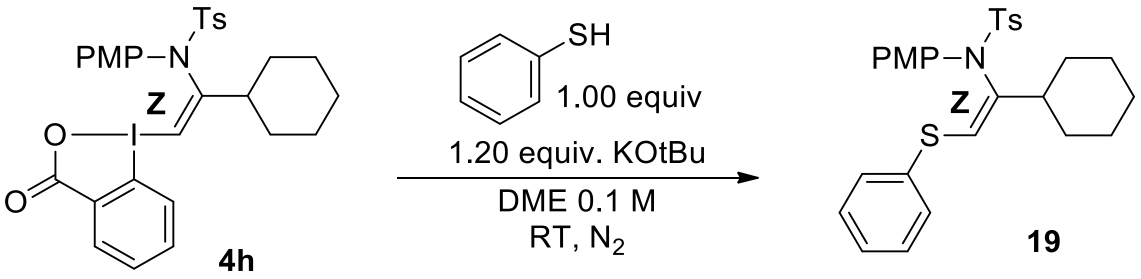

Commercially available thiophenol (10.0 µL, 0.100 mmol) and potassium 2-methylpropan-2-olate (13.0 mg, 0.120 mmol, 1.20 equiv.) were added to an oven-dried 5 mL microwave vial. The vial was capped with a rubber septum. Anhydrous DME (1.00 mL, 0.1 M) was introduced to the vial by syringe at 0 °C and the solution was stirred at room temperature for 10 min. N-vBX **4h** (63.0 mg, 0.100 mmol) was then added to the reaction mixture at room temperature under open air. The reaction mixture was stirred at room temperature for 16 h. The solvent was removed under reduced pressure and the crude material was purified by preparative TLC (Pentane:EtOAc 4:1) to afford (Z)-*N*-(1-cyclohexyl-2-(phenylthio)vinyl)-*N*-(4-methoxyphenyl)-4-methylbenzene-sulfonamide **19** in an un-separable *Z:E* mixture, as yellow sticky solid (26.0 mg, 52.0 µmol, 52%). *7:1 Z:E ratio.* **Rf:** 0.43 (Pentane:EtOAc 4:1). **^{1}H NMR** (400 MHz, Chloroform-*d*) *7:1 Z:E ratio* δ 7.68 – 7.63 (m, 2H, *ArH major*), 7.42 (dd, J = 8.7, 3.0 Hz, 2H, *ArH minor*), 7.39 – 7.27 (m, 14H, 7H *ArH major* + 7H *ArH minor*), 7.18 – 7.13 (m, 2H, *ArH major*), 7.12 – 7.08 (m, 2H, *ArH minor*), 6.98 – 6.93 (m, 1H, *ArH minor*), 6.84 (d, J = 2.3 Hz, 1H, *ArH minor*), 6.84 – 6.76 (m, 2H, *ArH major*), 6.24 (d, J = 0.7 Hz, 1H, vinyl*H major*), 6.00 (s, 1H, vinyl*H minor*), 3.83 (s, 3H, *OCH_3 minor*), 3.80 (s, 3H, *OCH_3 major*), 2.36 (s, 6H, 3H *CH_3 major* + 3H *CH_3 minor*), 2.14 – 1.96 (m, 6H, *CH_2 + CH major*), 1.76 (dd, J = 7.1, 3.4 Hz, 2H, *CH_2 major*), 1.71 – 1.61 (m, 11H, *CH_2 + CH minor*), 1.29 – 1.10 (m, 6H, *CH_2 major*). **^{13}C NMR** *major* (101 MHz, Chloroform-*d*) δ 159.1, 146.0, 143.4, 137.2, 136.3, 132.3, 130.4, 129.7, 129.2, 129.0, 128.5, 126.9, 124.2, 114.4, 55.5, 43.9, 32.9, 26.7, 26.2, 21.7. **IR** ν 3691 (w), 3674 (w), 2987 (s), 2975 (s), 2934 (s), 2899 (s), 1605 (w), 1583 (w), 1507 (s), 1478 (w), 1446 (m), 1442 (m), 1403 (m), 1397 (m), 1382 (m), 1343 (m), 1300 (m), 1253 (s), 1231 (m), 1163 (s), 1088 (s), 1078 (s), 1067 (s), 1037 (s), 963 (w), 910 (w), 894 (w), 867 (w), 832 (m),

816 (m), 802 (w), 744 (m), 708 (m), 683 (m), 667 (m), 650 (m). **HRMS** (ESI) calcd for $C_{28}H_{31}NNaO_3S_2^+$ $[M+Na]^+$ 516.1638; found 516.1648.

(Z)-*N*-(1-Cyclopentyl-2-iodovinyl)-*N*-(4-methoxyphenyl)-4-methylbenzenesulfonamide (20)

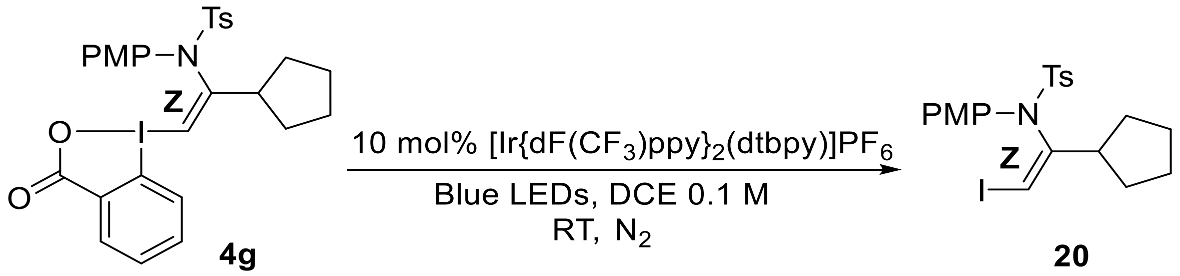

N-vBX **4g** (61.7, 0.100 mmol, 1.00 equiv) and [Ir{dF(CF$_3$)ppy}2(dtbpy)]PF$_6$ (11.2 mg, 10.0 μmol, 10 mol%) were added to an oven-dried 5 mL microwave vial. The vial was capped with a rubber septum. Anhydrous DCE (1.00 ml, 0.1 M) was introduced to the vial by syringe at 0 °C and the solution degassed then stirred at room temperature under irradiation with a blue light LED's for 12 h. The solvent was removed under reduced pressure and the crude material was purified by preparative TLC (Pentane/Ethyl acetate = 80:20) to afford (Z)-*N*-(1-cyclopentyl-2-iodovinyl)-N-(4-methoxyphenyl)-4-methylbenzenesulfonamide **20** as pale yellow oil (25.6 mg, 51.0 μmol, 51%). **Rf**: 0.60 (Pentane:EtOAc 10:1) **^{1}H NMR** (400 MHz, Chloroform-*d*) δ 7.65 (d, *J* = 8.1 Hz, 2H, Ar*H*), 7.48 – 7.39 (m, 2H, Ar*H*), 7.18 (d, *J* = 8.1 Hz, 2H, Ar*H*), 6.80 (d, *J* = 9.0 Hz, 2H, Ar*H*), 6.54 (d, *J* = 1.0 Hz, 1H, vinyl*H*), 3.79 (s, 3H, O*Me*), 2.66 (dt, *J* = 10.5, 7.3 Hz, 1H, C*H*), 2.38 (s, 3H, C*H$_3$*), 1.95 (s, 2H, C*H$_2$*), 1.80 – 1.65 (m, 2H, C*H$_2$*), 1.63 – 1.46 (m, 4H, C*H$_2$*).[14] **^{13}C NMR** (101 MHz, Chloroform-*d*) δ 159.1, 154.9, 143.4, 137.1, 131.7, 130.2, 128.9, 128.4, 114.1, 80.3, 55.4, 47.6, 33.2, 24.7, 21.5. **IR** ν 2982 (w), 2887 (w), 1737 (m), 1717 (m), 1527 (m), 1393 (w), 1369 (w), 1268 (w), 1178 (m), 1079 (m), 861 (m), 758 (s), 634 (s). **HRMS** (ESI/QTOF) calcd for $C_{21}H_{25}INO_3S^+$ [M+H]$^+$ 498.0594; Found 498.0601.

[14] ca 4% of PMPNHTs amide **3a** as by product.

5.3 Control experiment

Control experiment for the synthesis of (Z)-*N*-(Cyclopentylbuta-1,3-dien-1-yl)-N-(4-methoxyphenyl)-4-methylbenzenesulfonamide (14)

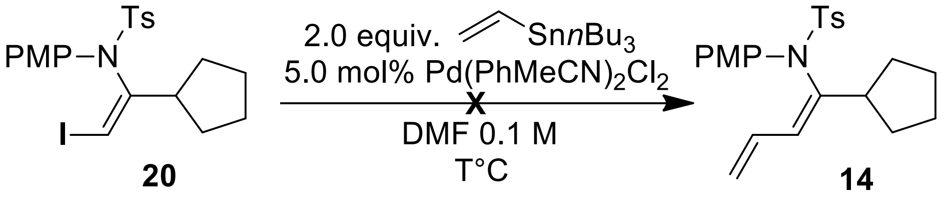

Following a reported procedure,[6] (Z)-*N*-(1-cyclopentyl-2-iodovinyl)-N-(4-methoxyphenyl)-4-methylbenzenesulfonamide **20** (49.7 mg, 0.100 mmol, 1.00 equiv.), Pd(PhCN)$_2$Cl$_2$ (1.90 mg, 5.00 µmol, 5 mol%) and commercially available tributyl(vinyl)stannane (60.5 µL, 0.200 mmol, 2.00 equiv.) were added to a flame-dried vial. Upon sealing and oxygen removing under vacuum, the vial was backfilled with nitrogen (process repeated for three cycles). Dry DMF (1.00 mL, 0.1 M) was added under nitrogen atmosphere and the reaction was left stirring at room temperature for 10 hours. Because no conversion was observed, the temperature was increased to 50 °C and stirred for 12 hours. To push the reaction the temperature was again increased to 75 °C for 12 hours and finally to 80 °C for 12 more hours. According to NMR, only 7 % of (Z)-N-(Cyclopentylbuta-1,3-dien-1-yl)-N-(4-methoxyphenyl)-4-methylbenzenesulfonamide **14** was formed (internal yield calculated using 50.0 µmol 1,3,5-trimethoxybenzene as an internal standard).

NMR spectra of the control experiment with integration.

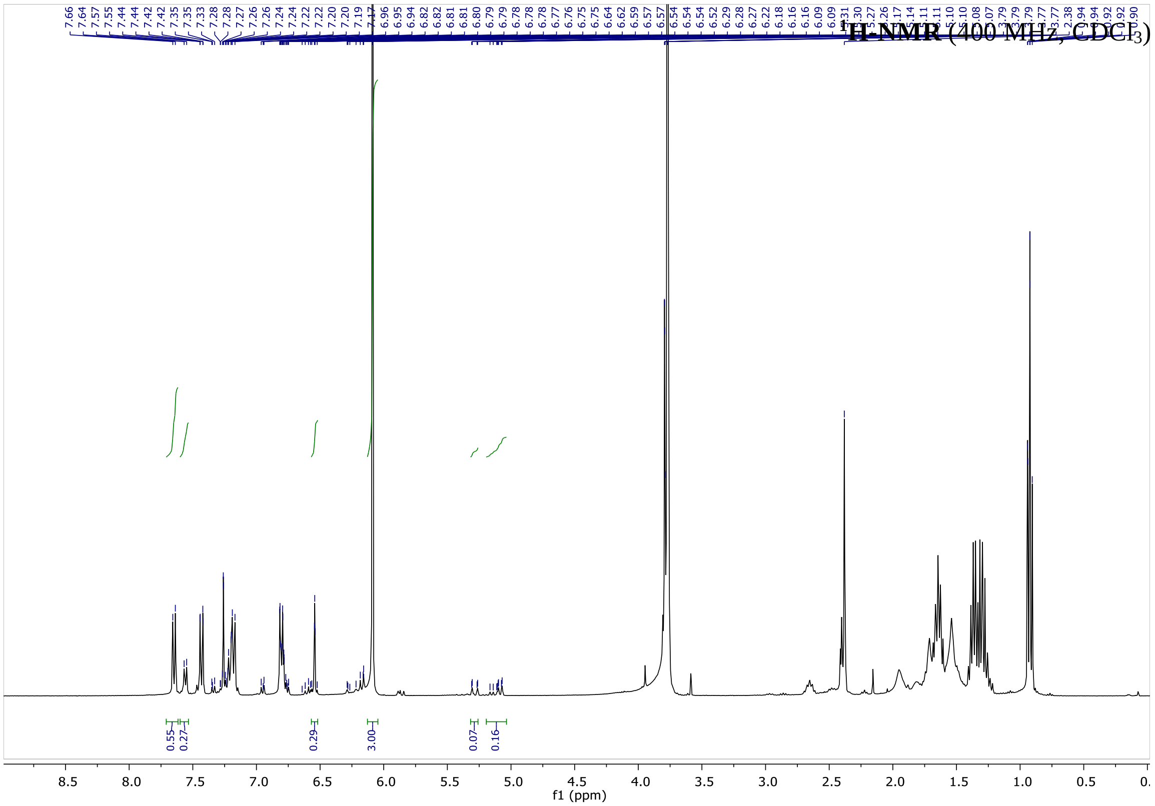

Stacked NMR spectra of 14 and the control experiment with internal standard.

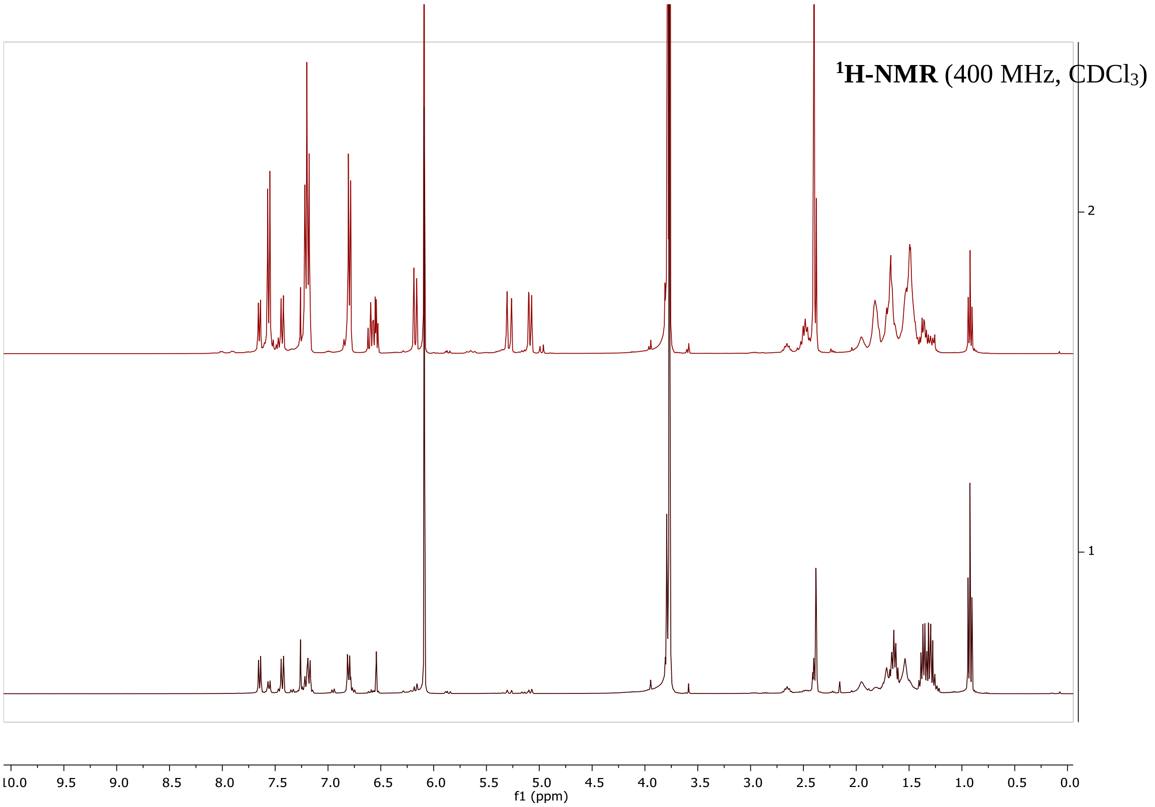

6. DFT calculations.

6.1 Computational Details

The geometries of all minima and transition states were optimized using the M06[15,16] density functional in tandem with the def2-SVP[17] basis set in implicit solvent (tetrahydrofuran) using the SMD solvation model[18] as implemented in Gaussian09. To remove known problems with the size of the integration grid for the Minnesota family of density functionals,[19] the "ultrafine" grid was employed. Alternative energy estimates were then obtained through single point computations on the optimized geometries using a density-dependent dispersion correction[20,21,22,23] (-dDsC) appended to the PBE0[24,25] functional (PBE0-dDsC) with the triple-ζ TZ2P basis set as implemented in ADF.[26,27] Final reported free energies include PBE0-dDsC electronic energies, M06 free energy corrections obtained using the rigid-rotor harmonic oscillator proposed by Grimme[28] and implemented in the "Goodvibes" program of Paton and Funes-Ardoiz,[29] and PBE0-dDsC solvation corrections (in tetrahydrofuran) obtained from COSMO-RS.[30] All structures were confirmed as either minima or transition states on the potential energy surface through inspection of the number of imaginary frequencies (zero for minima, one for transition states). The Cartesian coordinates of all structures are provided as separate .xyz files in the Supporting Information.

The Cartesian coordinates of all structures are provided as separate .xyz files in the Supporting Information.

---

[15] Y. Zhao, D. G. Truhlar, *Acc. Chem. Res.* 2008, **41**, 157.
[16] Y. Zhao, D. G. Truhlar, *Theor. Chem. Acc.* 2008, **120**, 215.
[17] F. Weigend, R. Ahlrichs, *Phys. Chem. Chem. Phys.* 2005, **7**, 3297.
[18] A. V. Marenich, C. J. Cramer, D. G. Truhlar, *J. Phys. Chem. B* 2009, **113**, 6378.
[19] S. E. Wheeler, K. N. Houk, *J. Chem. Theory Comput.* 2010, **6**, 395.
[20] S. N. Steinmann, C. Corminboeuf, *J. Chem. Theory Comput.* 2010, **6**, 1990.
[21] S. N. Steinmann, C. Corminboeuf, *J. Chem. Theory Comput.* 2011, **7**, 3567.
[22] S. N. Steinmann, C. Corminboeuf, *J. Chem. Phys.* 2011, **134**, 044117.
[23] C. Corminboeuf, S. N. Steinmann, C. Corminboeuf, *Chimia (Aarau).* 2011, **65**, 240.
[24] J. P. Perdew, K. Burke, M. Ernzerhof, *Phys. Rev. Lett.* 1996, **77**, 3865.
[25] C. Adamo, V. Barone, *J. Chem. Phys.* 1999, **110**, 6158.
[26] C. Fonseca Guerra, J. G. Snijders, G. te Velde, E. J. Baerends, *Theor. Chem. Accounts Theory, Comput. Model. (Theoretica Chim. Acta)* 1998, **99**, 391.
[27] G. te Velde, F. M. Bickelhaupt, E. J. Baerends, C. Fonseca Guerra, S. J. A. van Gisbergen, J. G. Snijders, T. Ziegler, *J. Comput. Chem.* 2001, **22**, 931.
[28] S. Grimme, *Chem. - A Eur. J.* 2012, **18**, 9955.
[29] I. Funes-Ardoiz, R. S. Paton, 2016, DOI 10.5281/ZENODO.60811.
[30] A. Klamt, *Wiley Interdiscip. Rev. Comput. Mol. Sci.* 2011, **1**, 699.

Table 6.1. Computed electronic energies, free energy corrections, and solvation energies for relevant species. Values in hartree. Free energies reported in the manuscript including PBE0-dDsC/TZ2P electronic energies, M06 free energy corrections (determined using the rigid-rotor harmonic oscillator, see computational details), and COSMO-RS solvation energies (in tetrahydrofuran).

	M06/def2-SVP Electronic Energy	Free Energy Correction	PBE0-dDsC/TZ2P Electronic Energy	COSMO-RS Solvation Energy
TMG	-362.553635	0.166251	-5.242654	-0.089571
**Nitrogen Species**				
A(0)	-1821.222348	0.259905	-12.209248	-0.096613
A(TS1)	-1821.188215	0.259748	-12.180098	-0.087636
A(1)	-1821.210048	0.260851	-12.194007	-0.093123
A(TS2)	-1821.207355	0.260584	-12.191873	-0.095854
A(2)	-1821.282275	0.260737	-12.265200	-0.100880
B(TS1)	-1821.203016	0.260586	-12.197311	-0.087663
B(1)	-1821.226857	0.262900	-12.216197	-0.093423
B(TS2)	-1821.195683	0.260066	-12.171282	-0.106571
B(2)	-1821.221360	0.259640	-12.199005	-0.099069
B(TS3)	-1821.192865	0.258682	-12.171448	-0.103495
B(3)	-1821.287287	0.261662	-12.272860	-0.096294
B(1')	-2183.812158	0.453483	-17.588355	-0.070982
B(TS2')	-2183.811262	0.451042	-17.593132	-0.064750
B(2')	-2183.841353	0.452674	-17.632039	-0.049263
**Oxygen Species**				
A(0)	-1178.437471	0.209640	-10.065466	-0.084499
A(TS1)	-1178.396835	0.208713	-10.031237	-0.081637
A(1)	-1178.480705	0.211077	-10.100300	-0.100546
B(TS1)	-1178.415170	0.210251	-10.048481	-0.082586
B(1)	-1178.441468	0.213173	-10.074517	-0.087198
B(TS2)	-1178.406803	0.210425	-10.024610	-0.100021
B(2)	-1178.411084	0.209626	-10.030996	-0.100219
B(TS3)	-1178.383809	0.208714	-10.017328	-0.085883
B(3)	-1178.486020	0.211767	-10.111641	-0.092600
B(1')	-1541.020402	0.401298	-15.441087	-0.065578
B(TS2')	-1541.020379	0.399981	-15.443390	-0.063358
B(2')	-1541.056371	0.404323	-15.490209	-0.043582

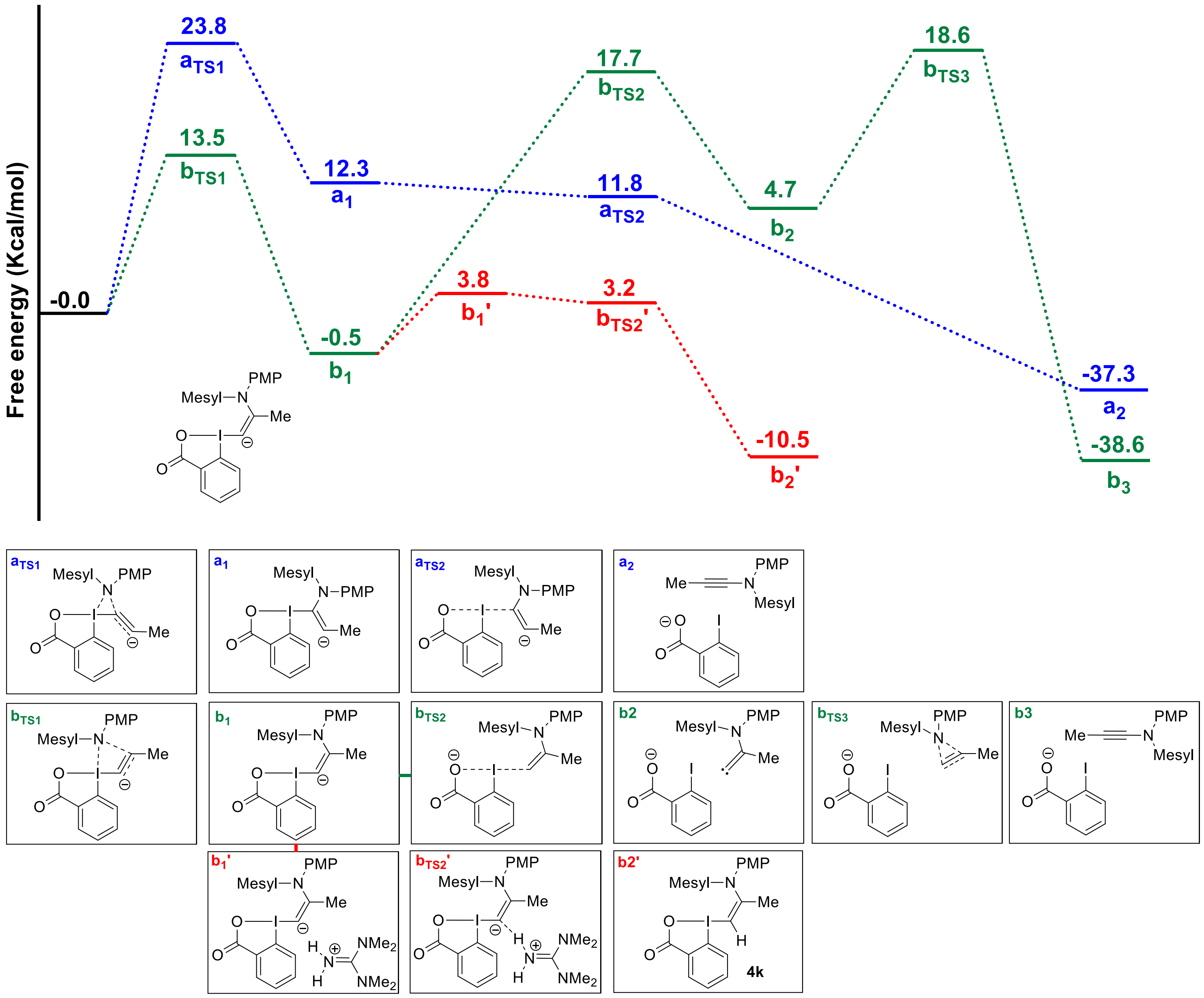
Free energy (Kcal/mol)
-0.0
23.8
aTS1
13.5
bTS1
12.3
a1
-0.5
b1
3.8
b1'
17.7
bTS2
11.8
aTS2
3.2
bTS2'
4.7
b2
-10.5
b2'
18.6
bTS3
-37.3
a2
-38.6
b3
PMP
Mesyl
Me
4k
NMe2

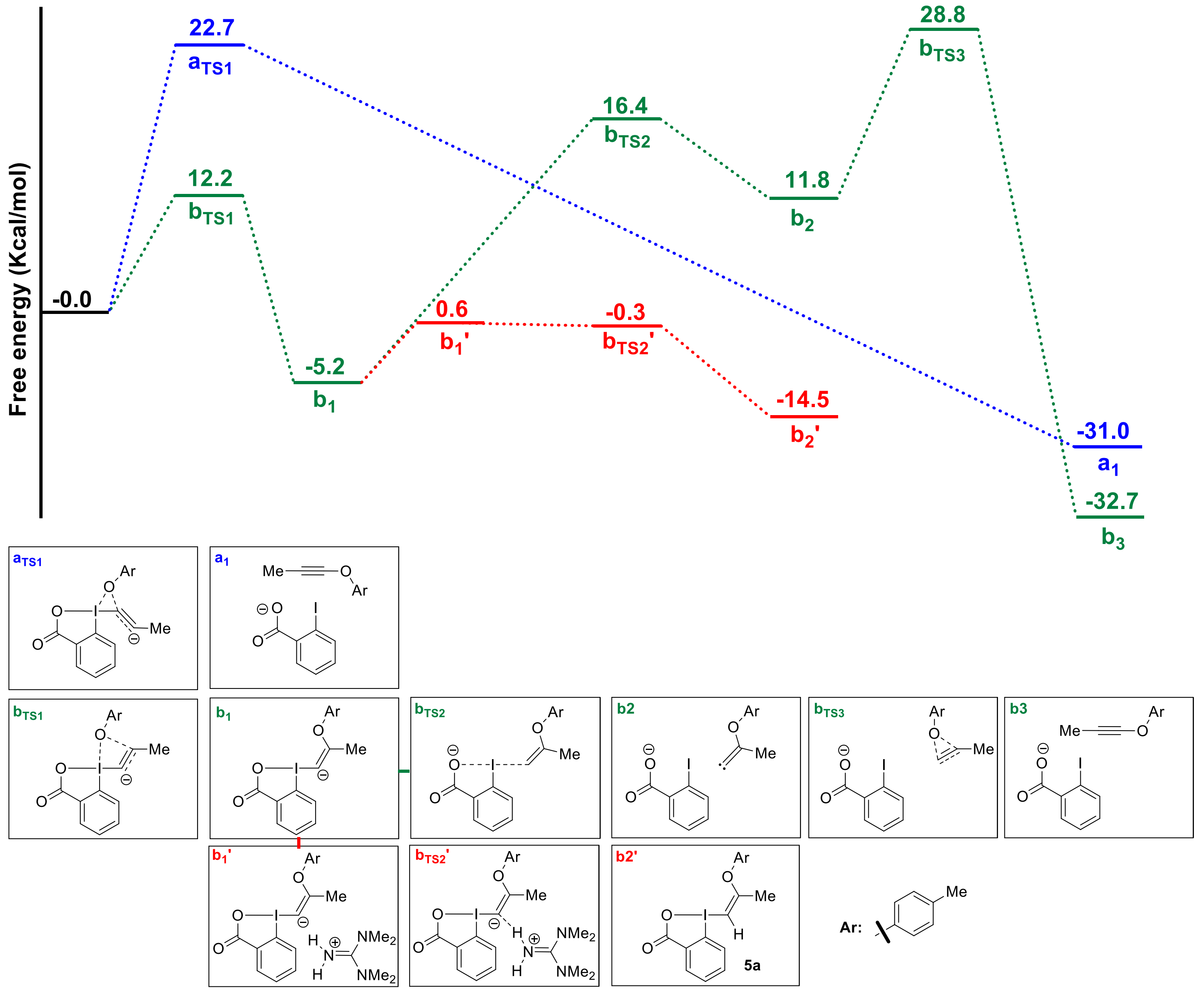
Free energy (Kcal/mol)
-0.0
22.7
aTS1
12.2
bTS1
-5.2
b1
0.6
b1'
16.4
bTS2
-0.3
bTS2'
11.8
b2
-14.5
b2'
28.8
bTS3
-31.0
a1
-32.7
b3
aTS1
a1
bTS1
b1
bTS2
b2
bTS3
b3
b1'
bTS2'
b2'
5a
Ar:
Ar
Me
NMe2

7. Crystal Structure.

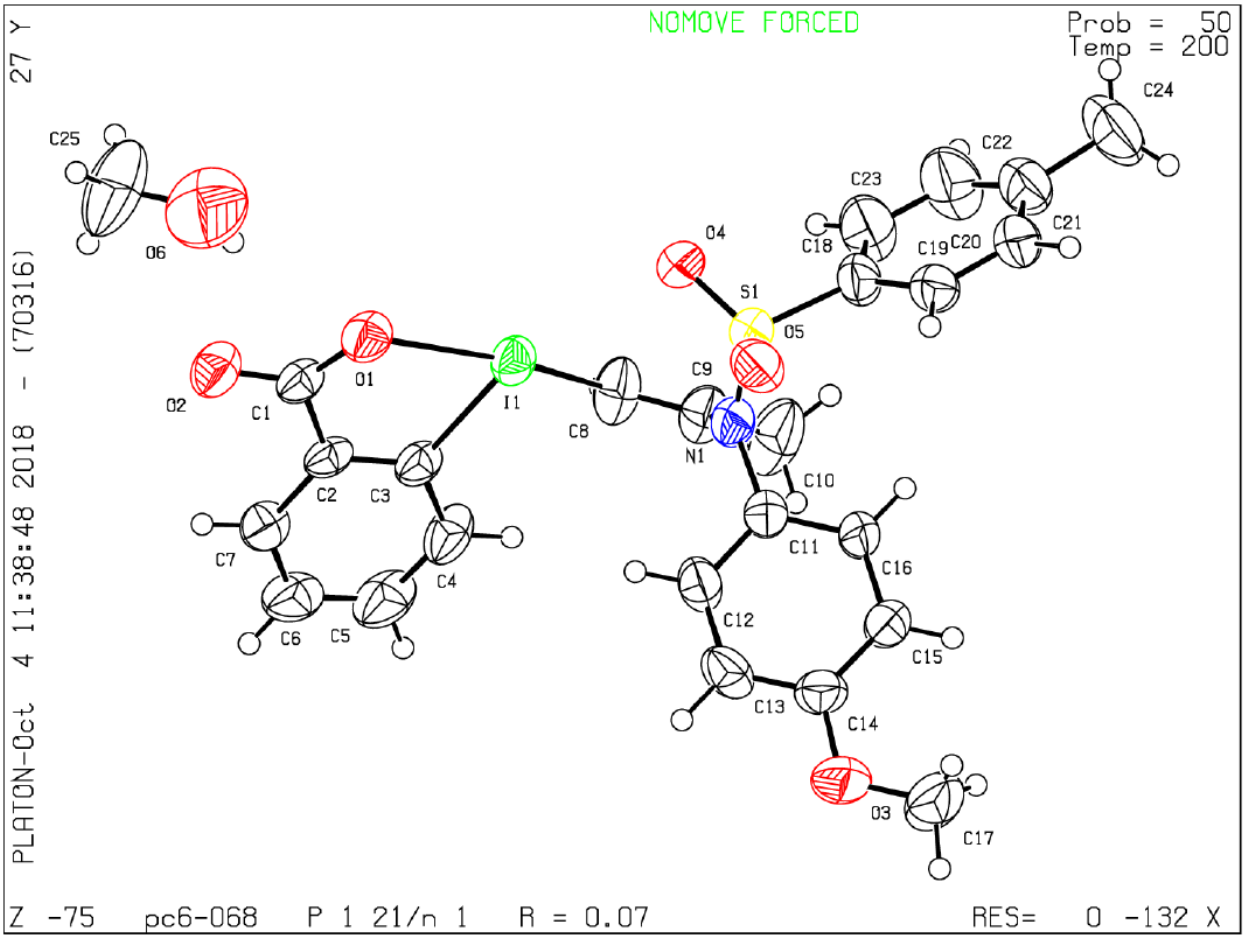

A single crystal was grown by slow diffusion of the solution of **4a** in MeOH mixture. Supplementary crystallographic data for this compound have been deposited at Cambridge Crystallographic Data Centre (**1876011**) and can be obtained free of charge via www.ccdc.cam.ac.uk/data_request/cif.

8. Spectra of new compounds

(Pent-1-ynyl)-1,2-benziodoxol-3(1*H*)-one (2c)

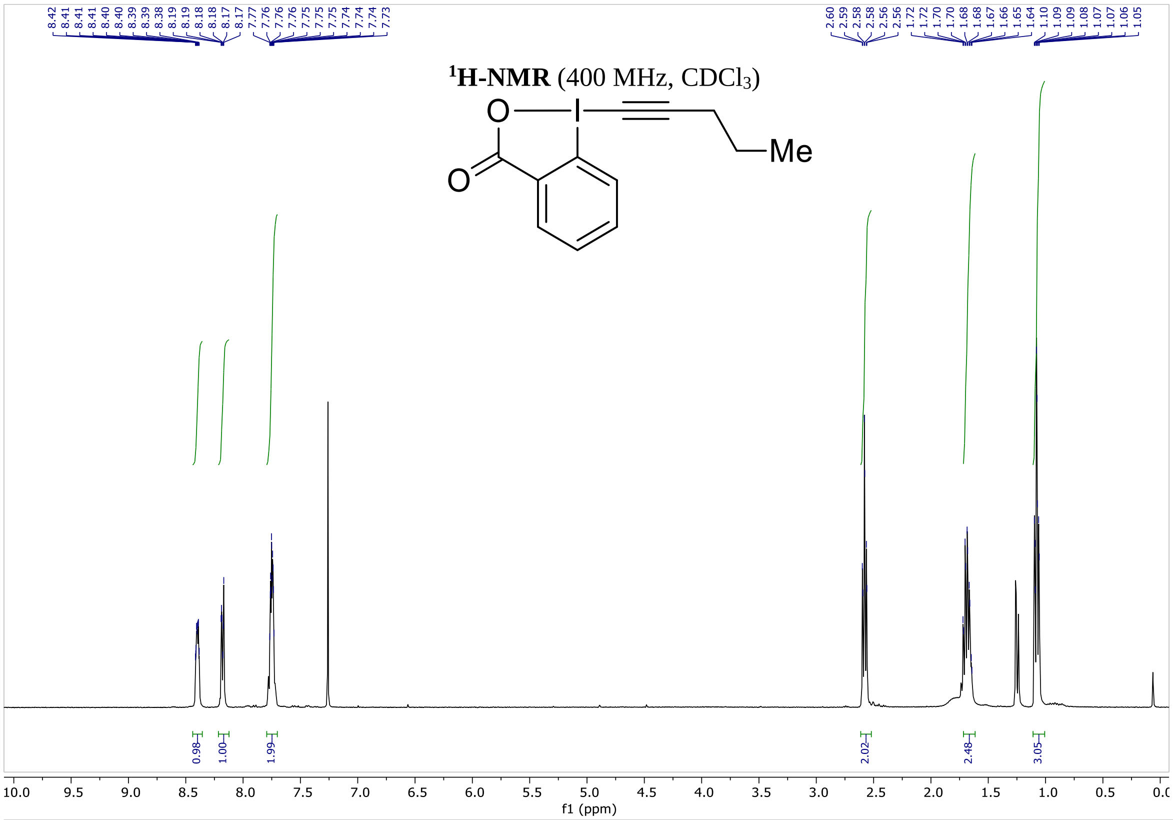

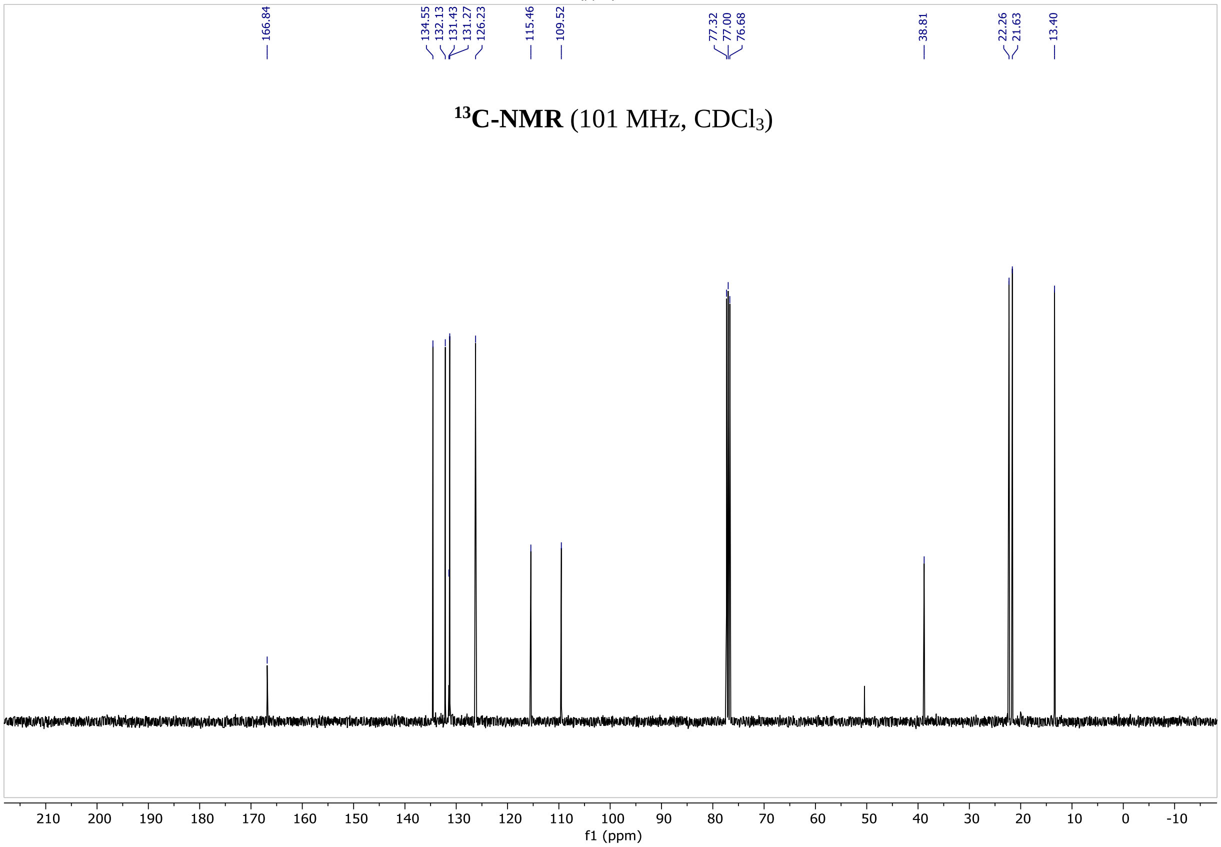

2-Cyclopentylethynyl-1,2-benziodoxol-3(1*H*)-one (2g)

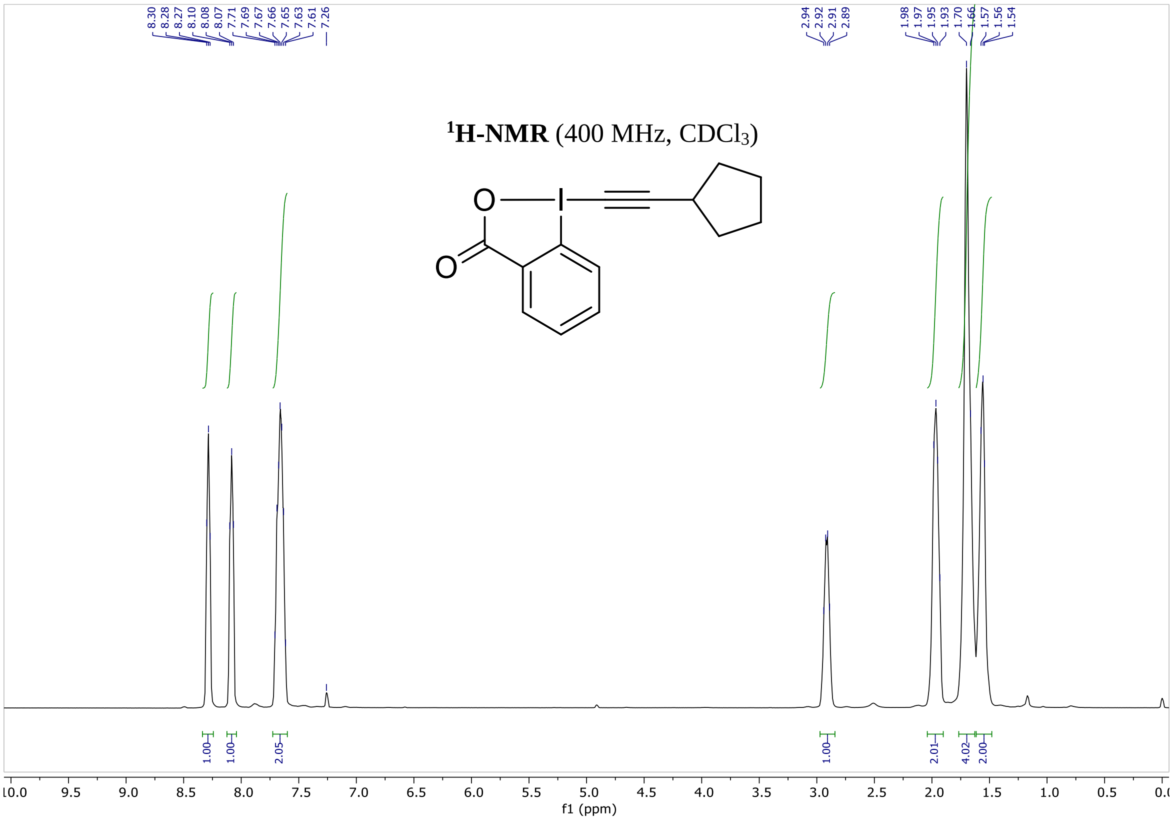

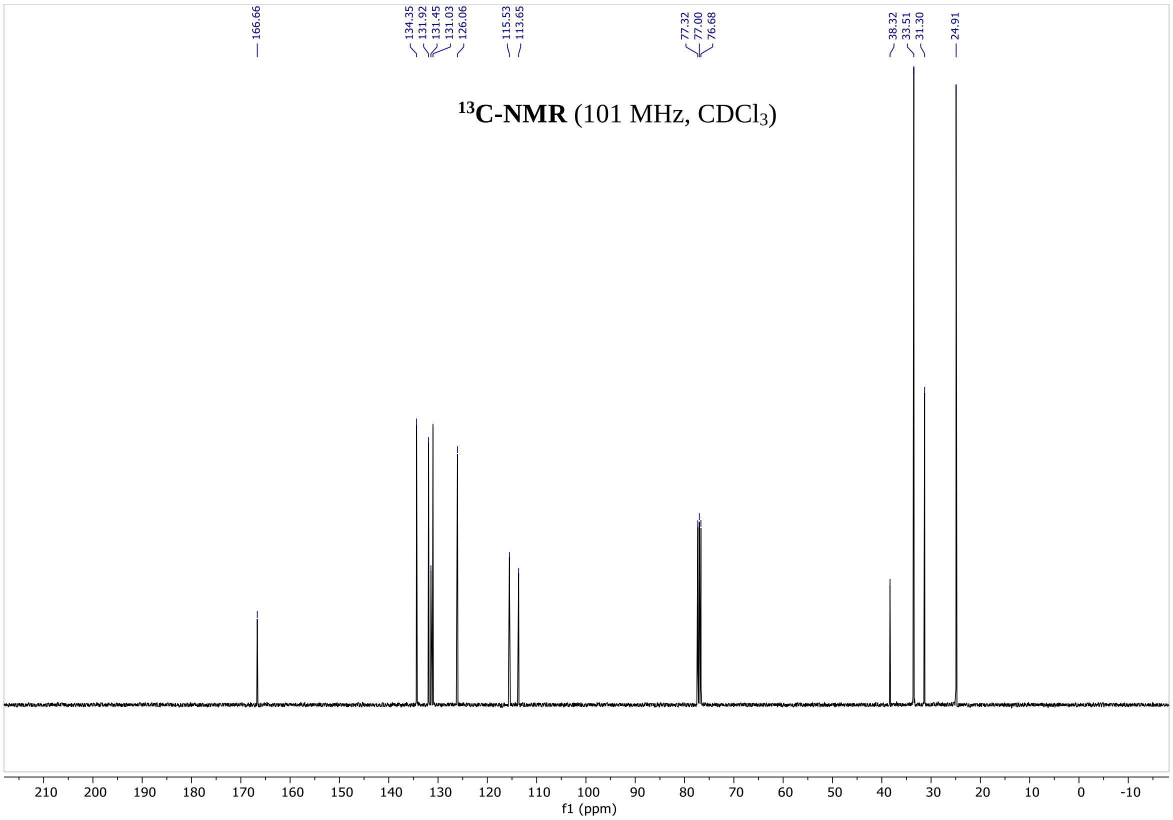

2-Cyclohexylethynyl-1,2-benziodoxol-3(1*H*)-one (2h)

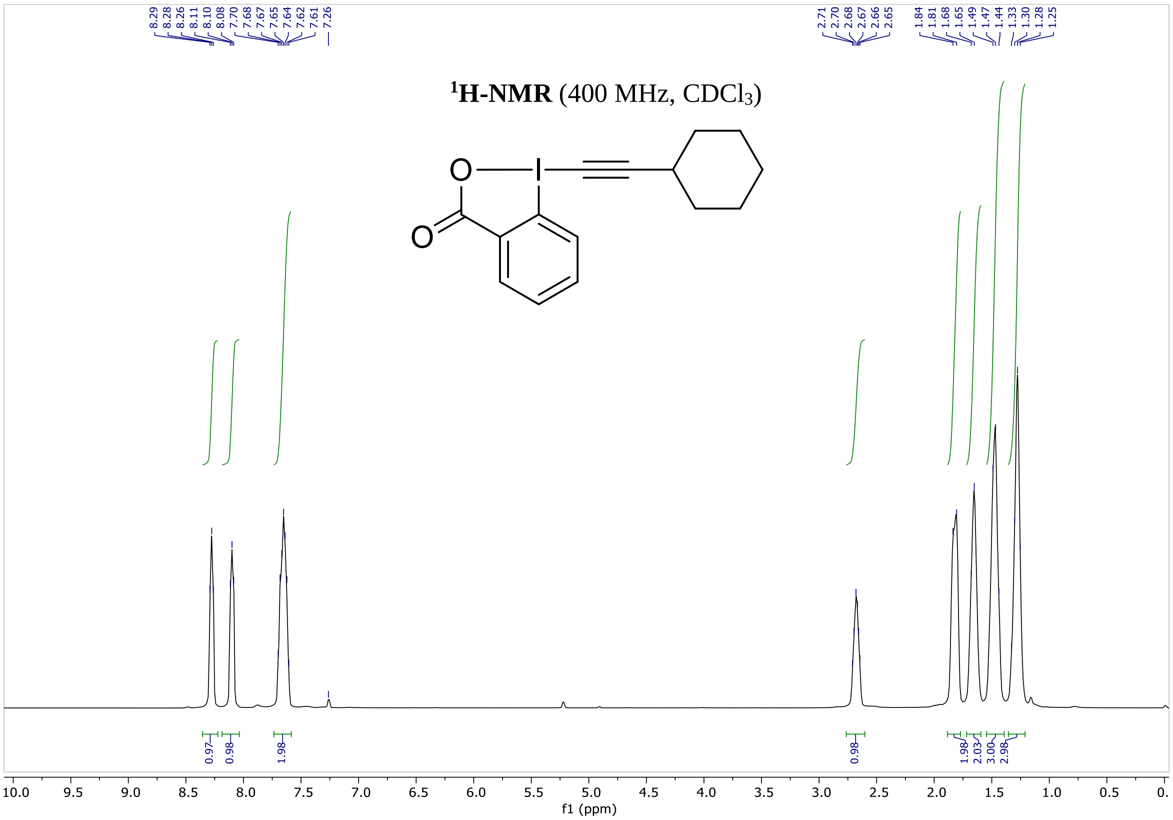

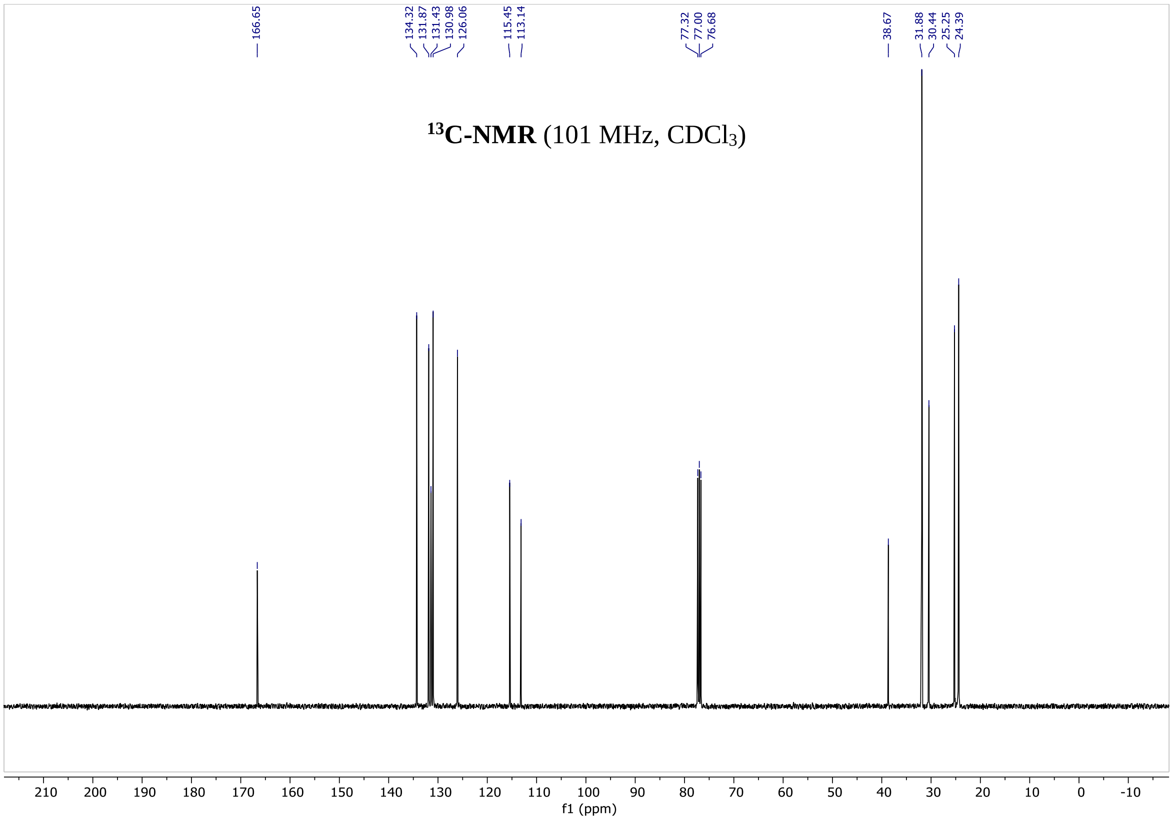

(Z)-*N*-(1-prop-1-en-2-yl)-*N*-(4-methoxyphenyl)-4-methylbenzenesulfonamide-1,2-benziodoxol-3-(1*H*)-one (4a)

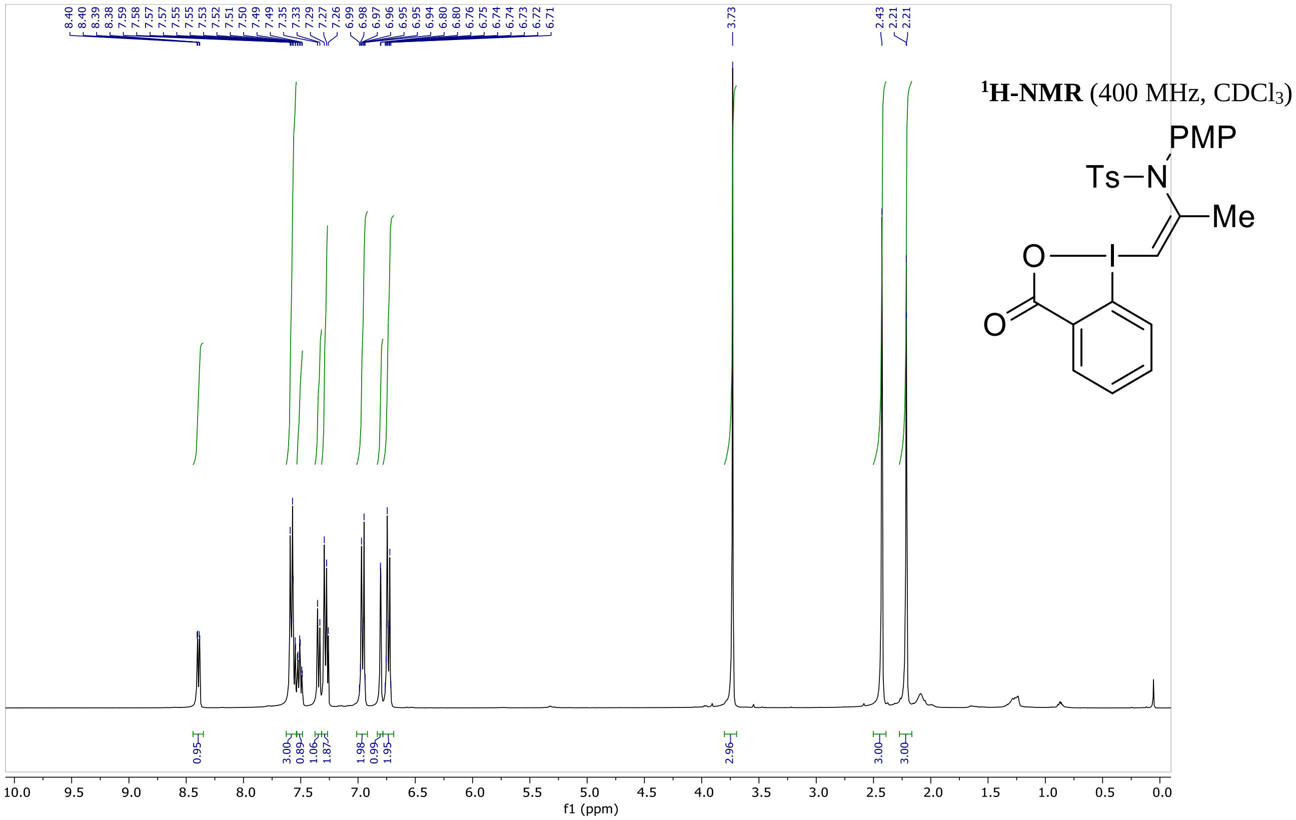

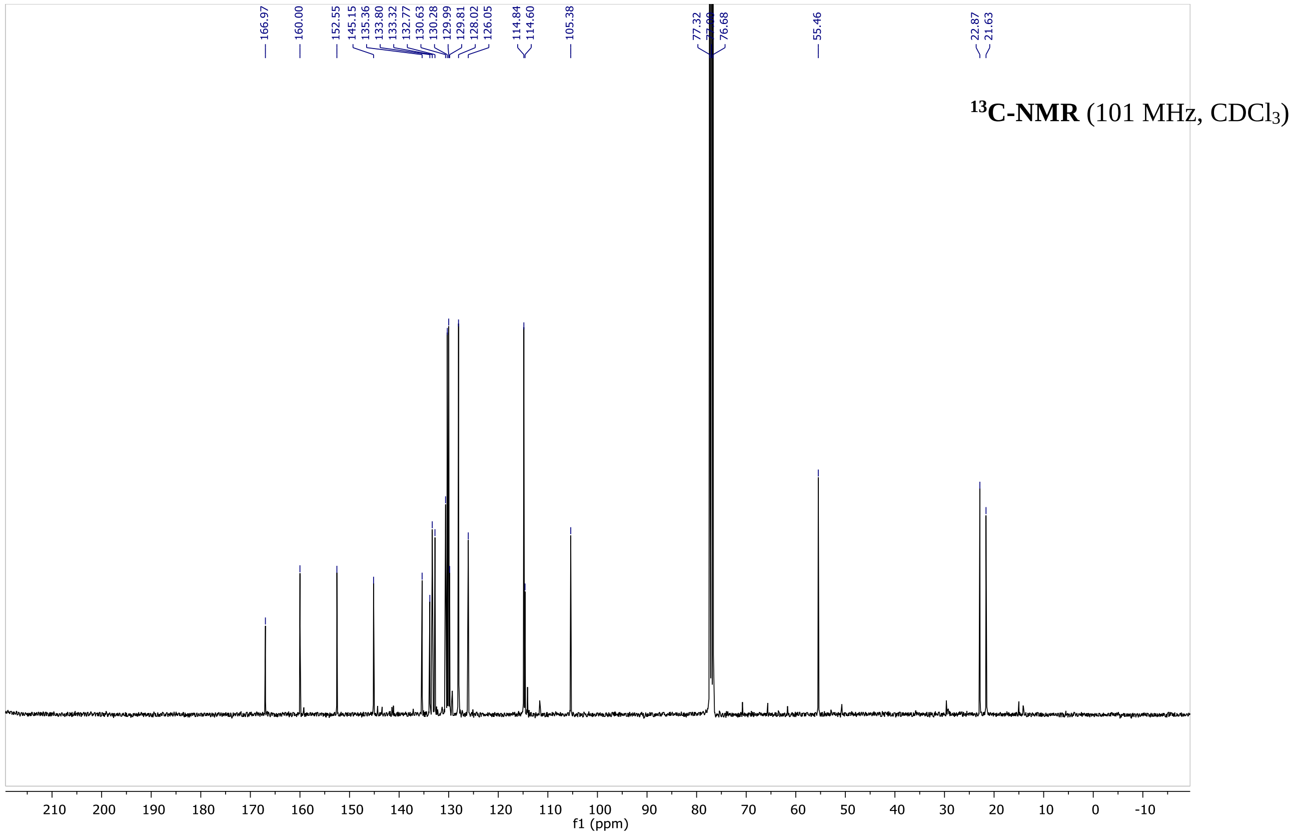

(Z)-*N*-(1-vin-2-yl)-*N*-(4-methoxyphenyl)-4-methylbenzenesulfonamide-1,2-benziodoxol-3-(1*H*)-one (4b)

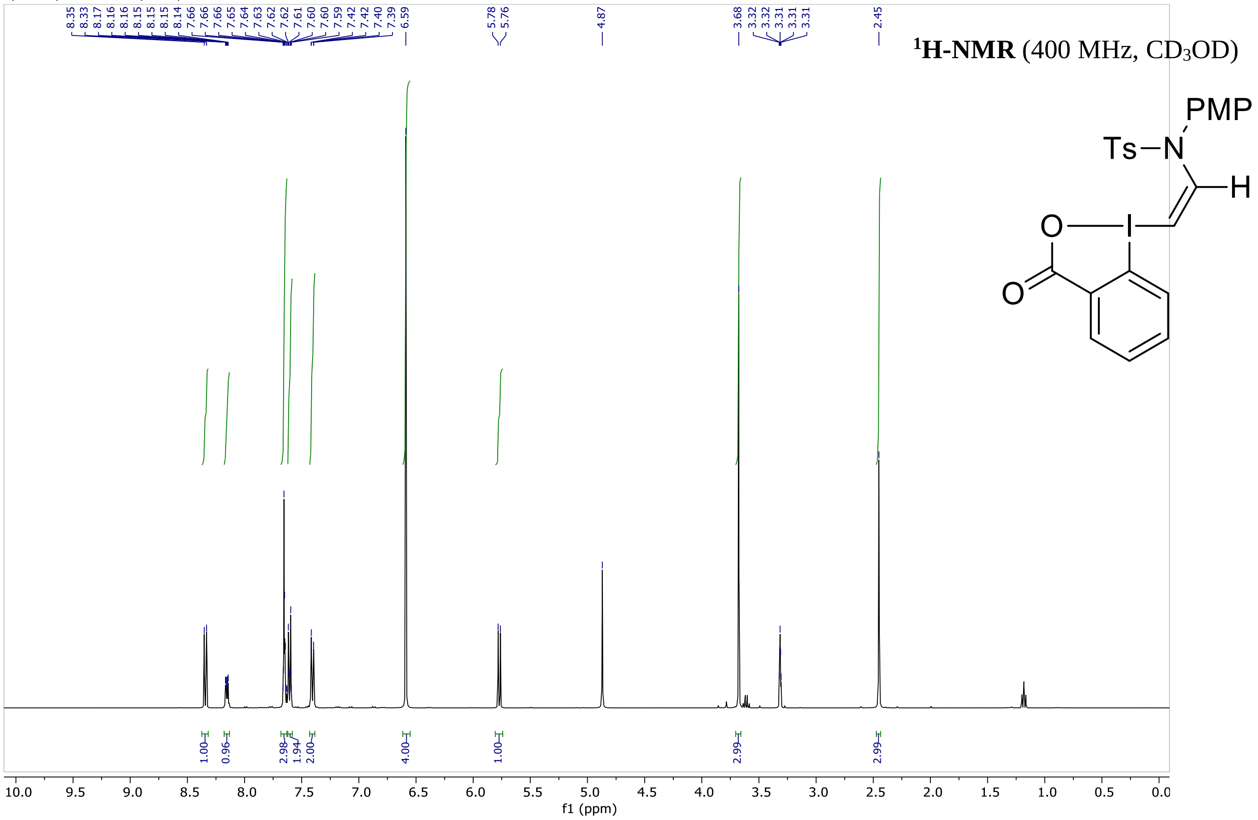

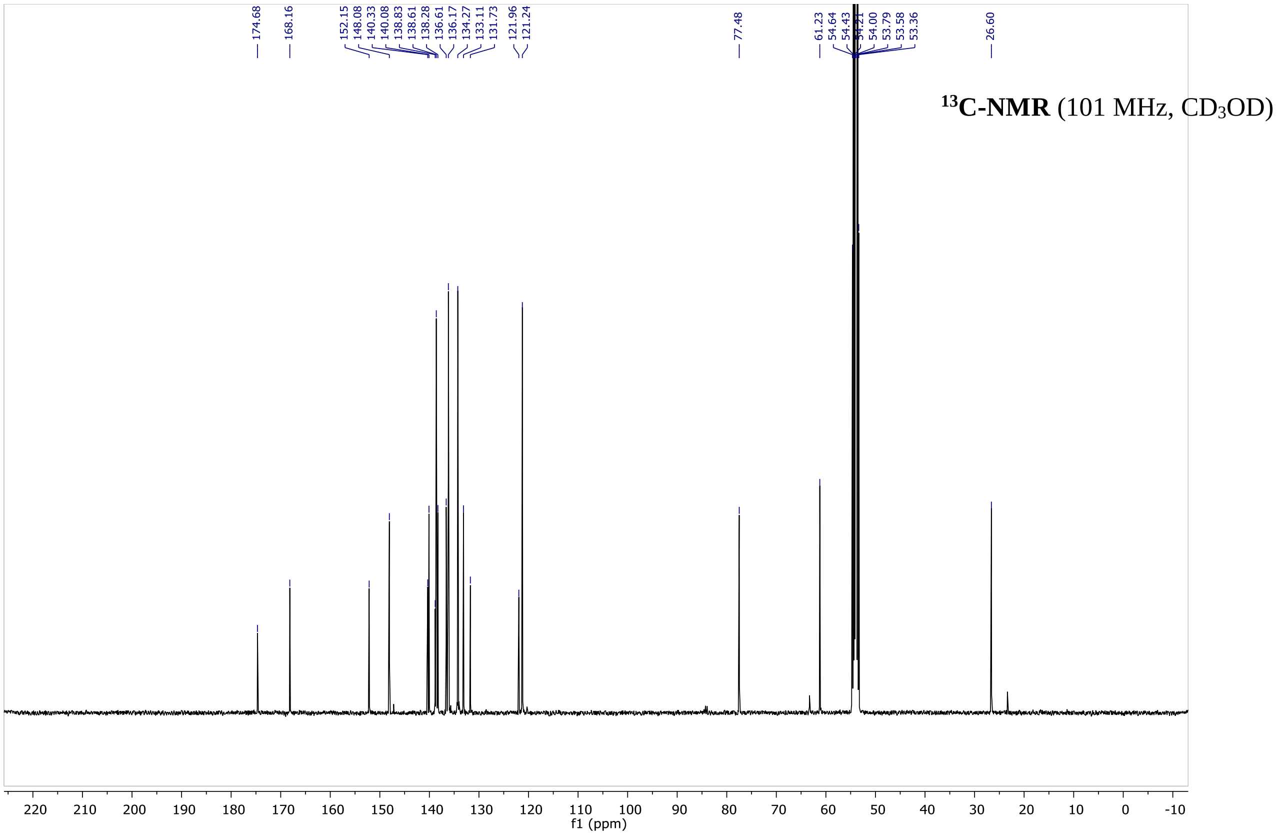

(Z)-*N*-(1-pent-1-en-2-yl)-*N*-(4-methoxyphenyl)-4-methylbenzenesulfonamide-1,2-benziodoxol-3-(1*H*)-one (4c)

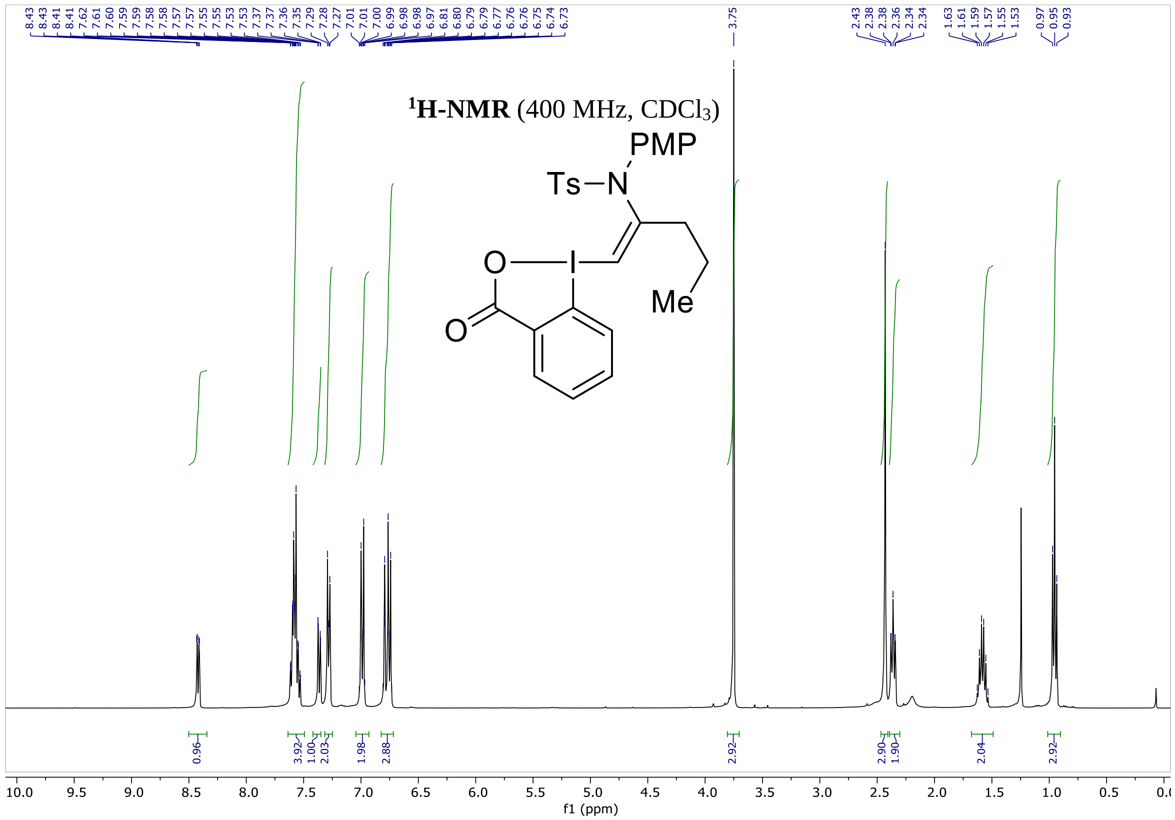

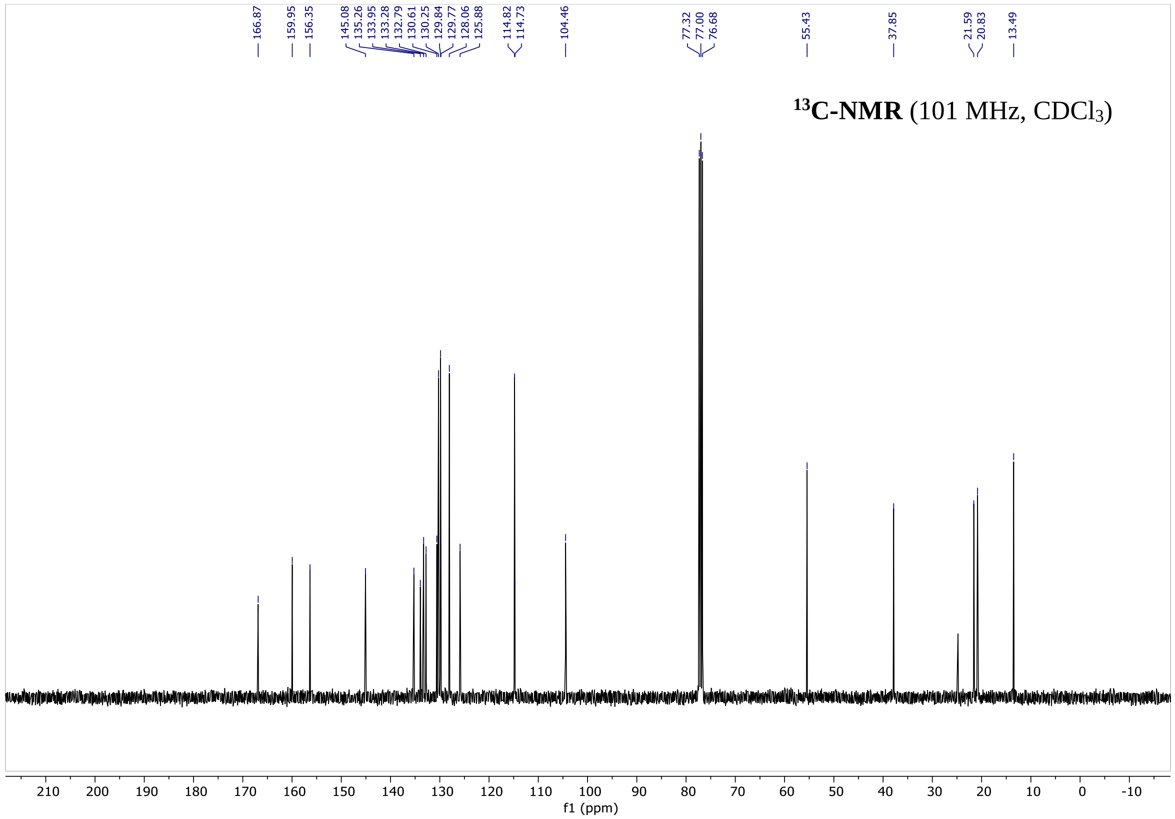

(Z)-*N*-(5-chloro-1-pent-1-en-2-yl)-*N*-(4-methoxyphenyl)-4-methylbenzenesulfonamide-1,2-benziodoxol-3-(1*H*)-one (4d)

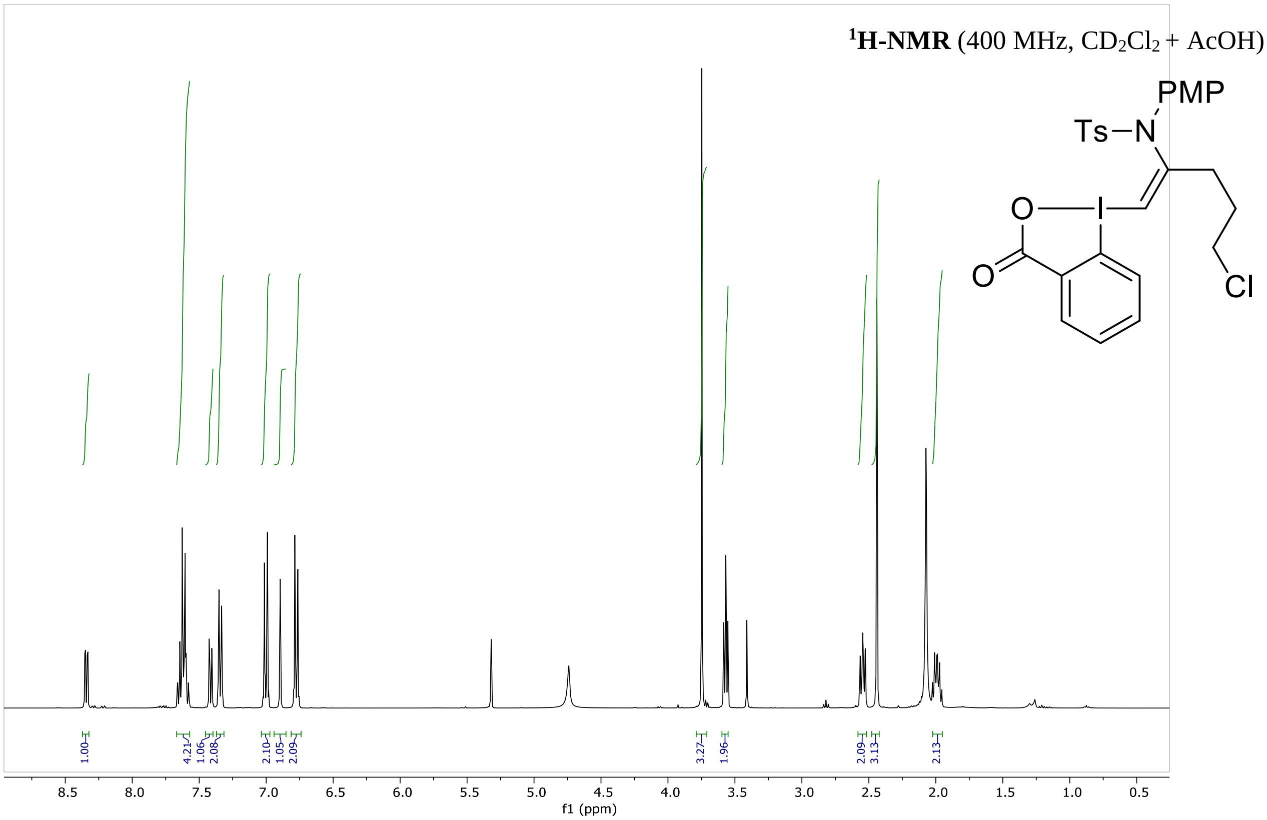

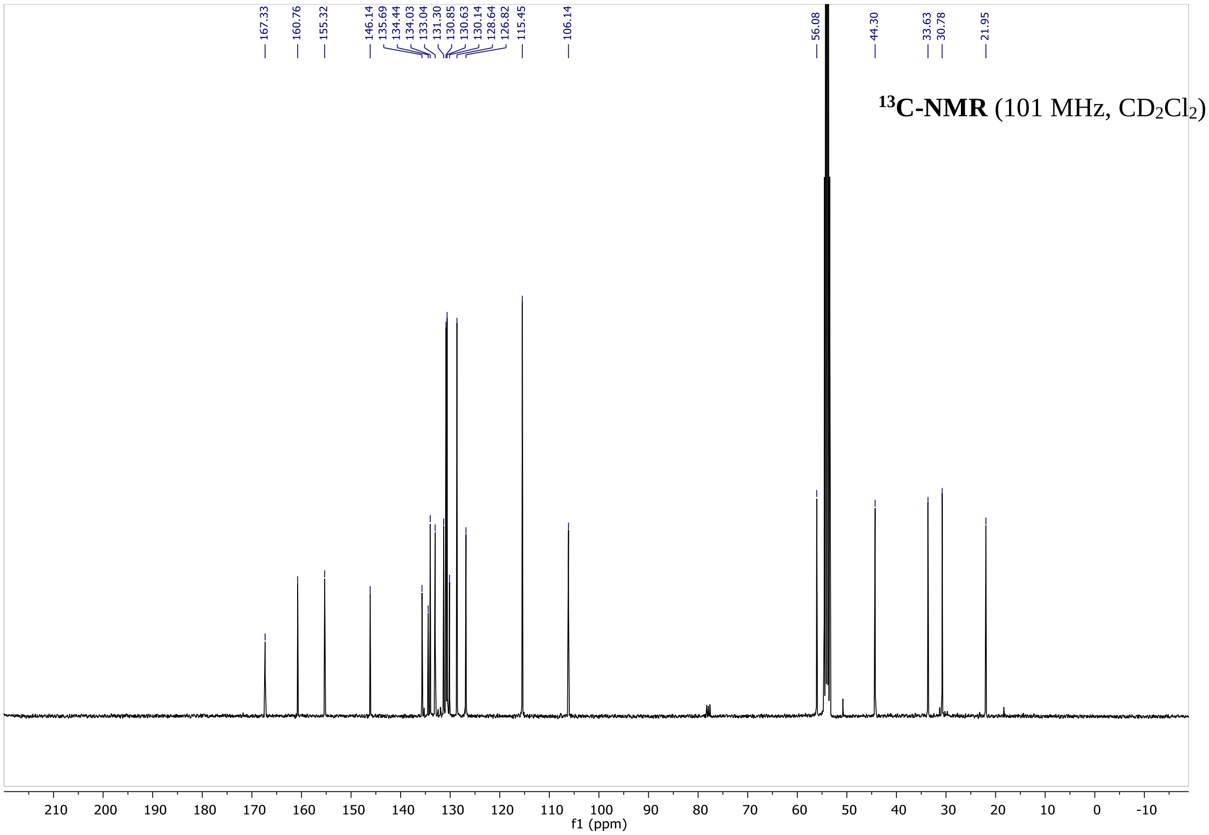

(Z)-*N*-(8-(trimethylsilyl)oct-1-en-7-yn-2-yl)-*N*-(4-methoxyphenyl)-4-methylbenzene sulfonamide-1,2-benziodoxol-3-(1*H*)-one (4e)

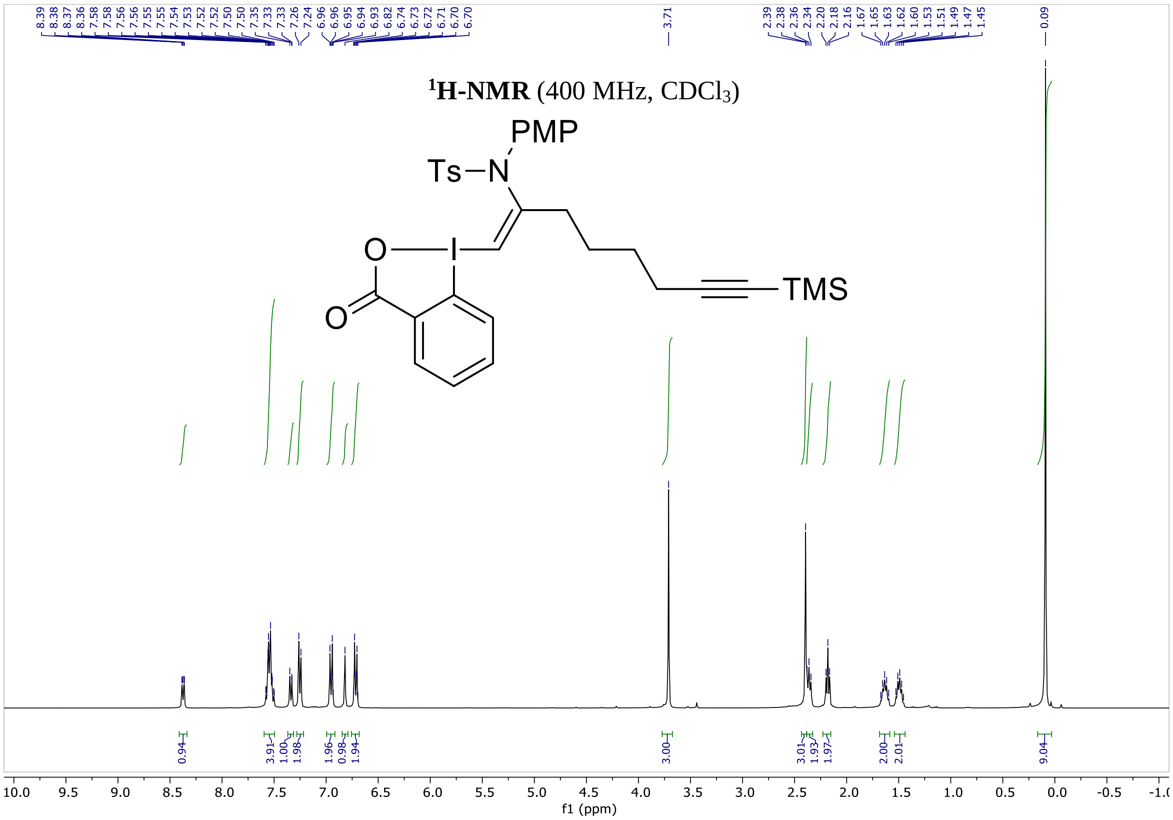

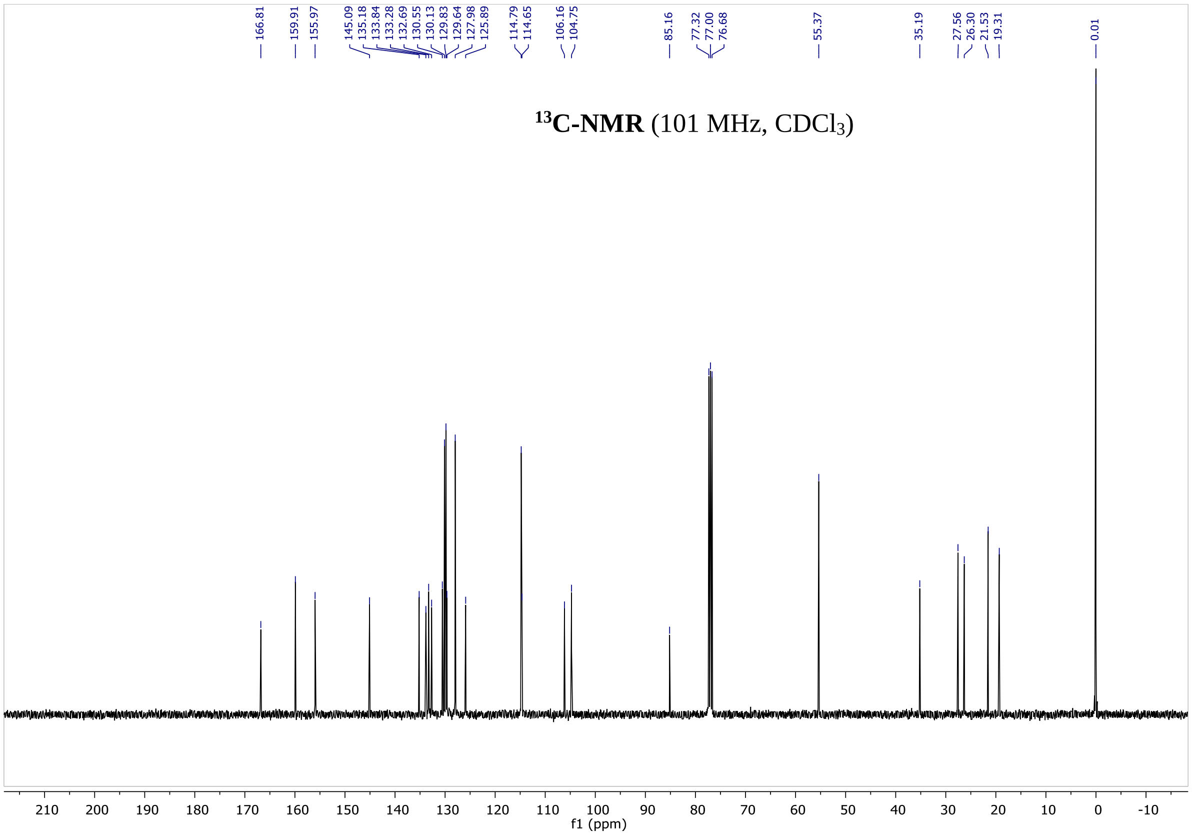

(Z)-*N*-(1-vin-2-yl-2-cyclopropyl)-*N*-(4-methoxyphenyl)-4-methylbenzenesulfonamide-1,2-benziodoxol-3-(1*H*)-one (4f)

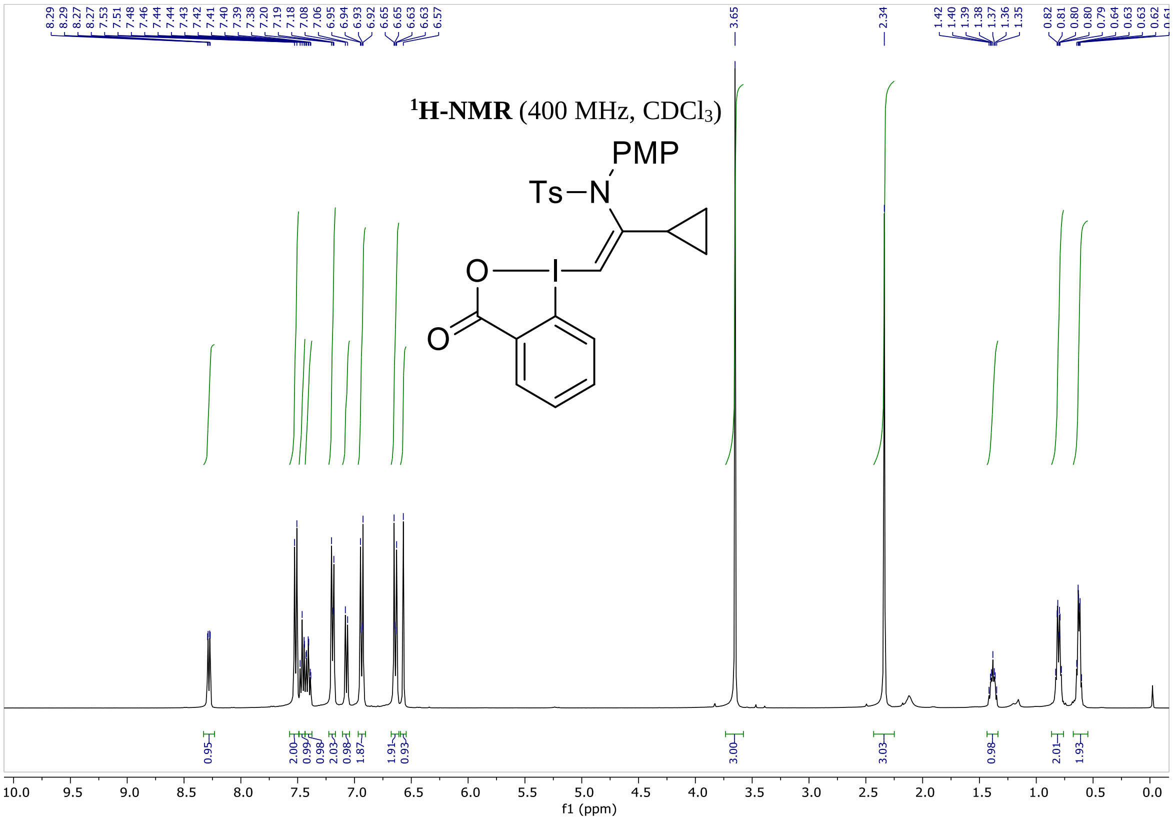

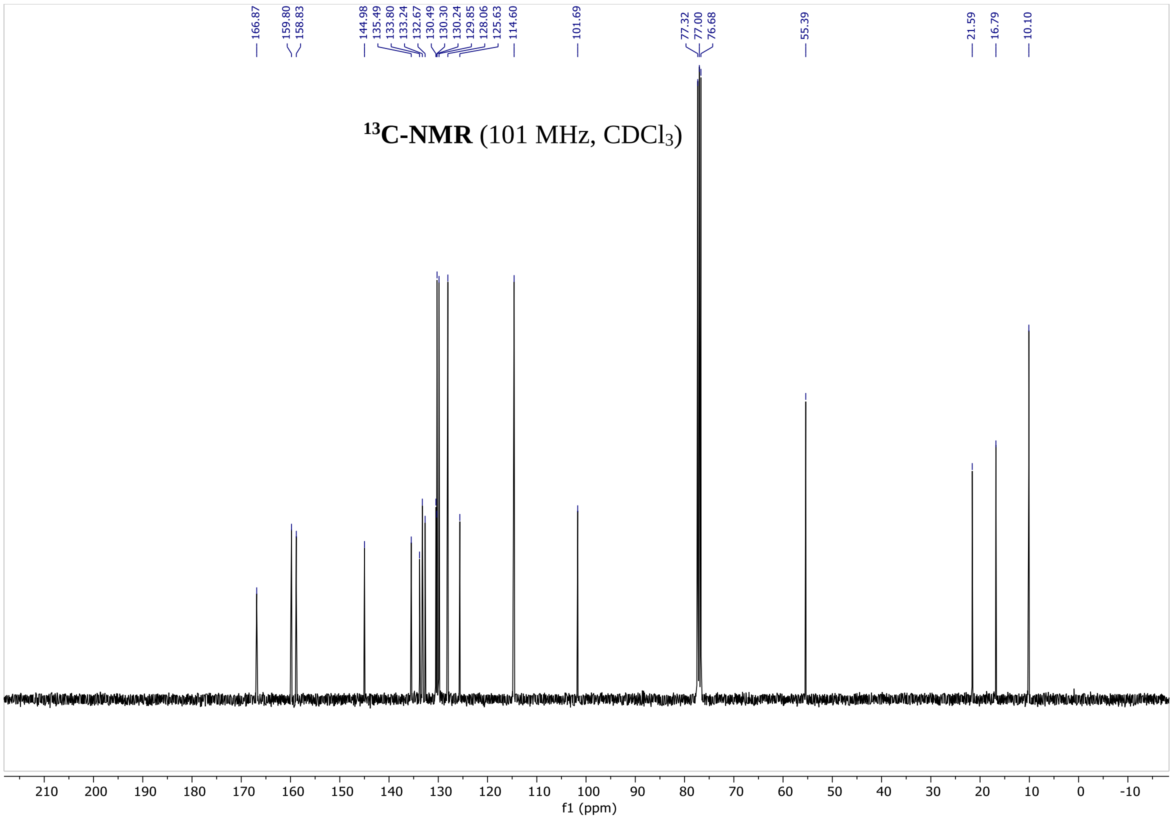

(Z)-*N*-(1-vin-2-yl-2-cyclopentyl)-*N*-(4-methoxyphenyl)-4-methylbenzenesulfonamide-1,2-benziodoxol-3-(1*H*)-one (4g)

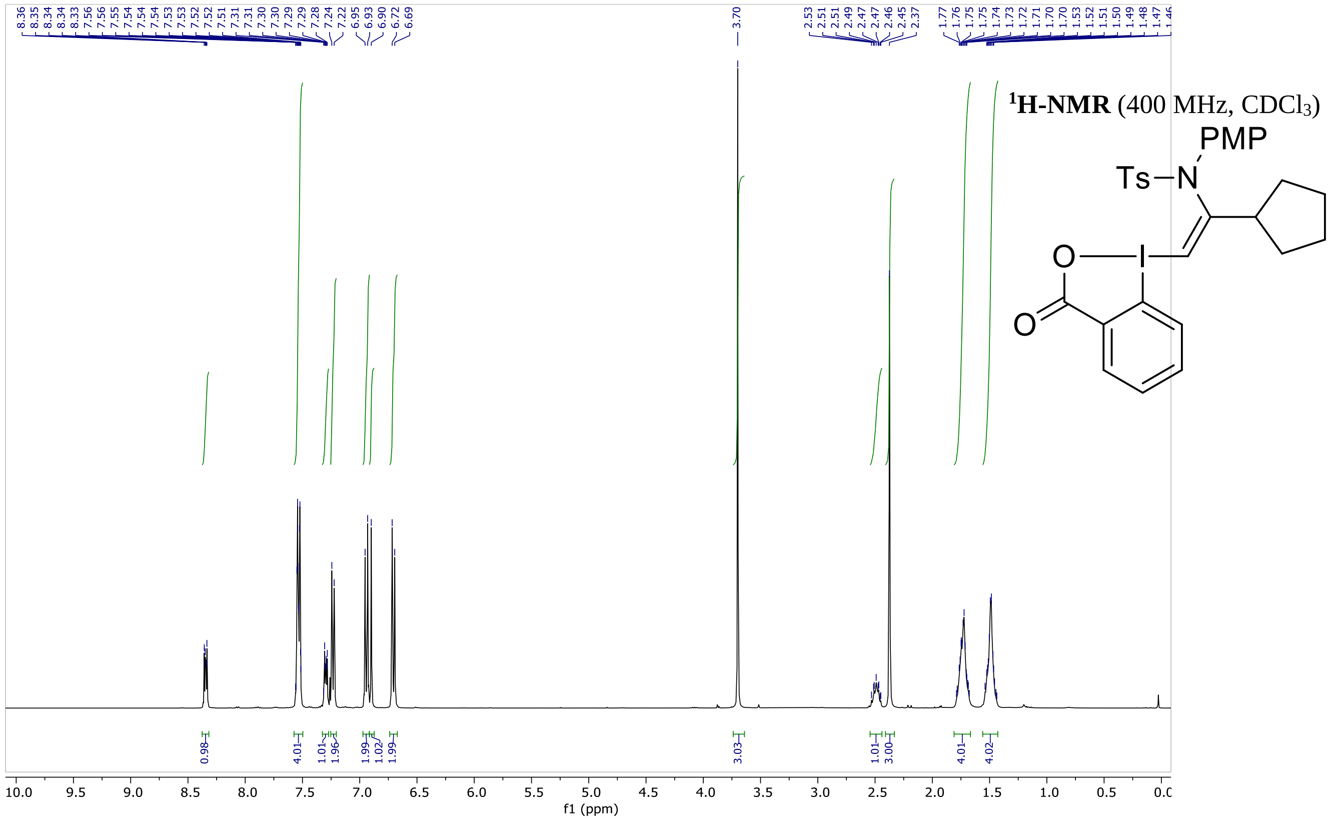

[1]**H-NMR** (400 MHz, $CDCl_3$)

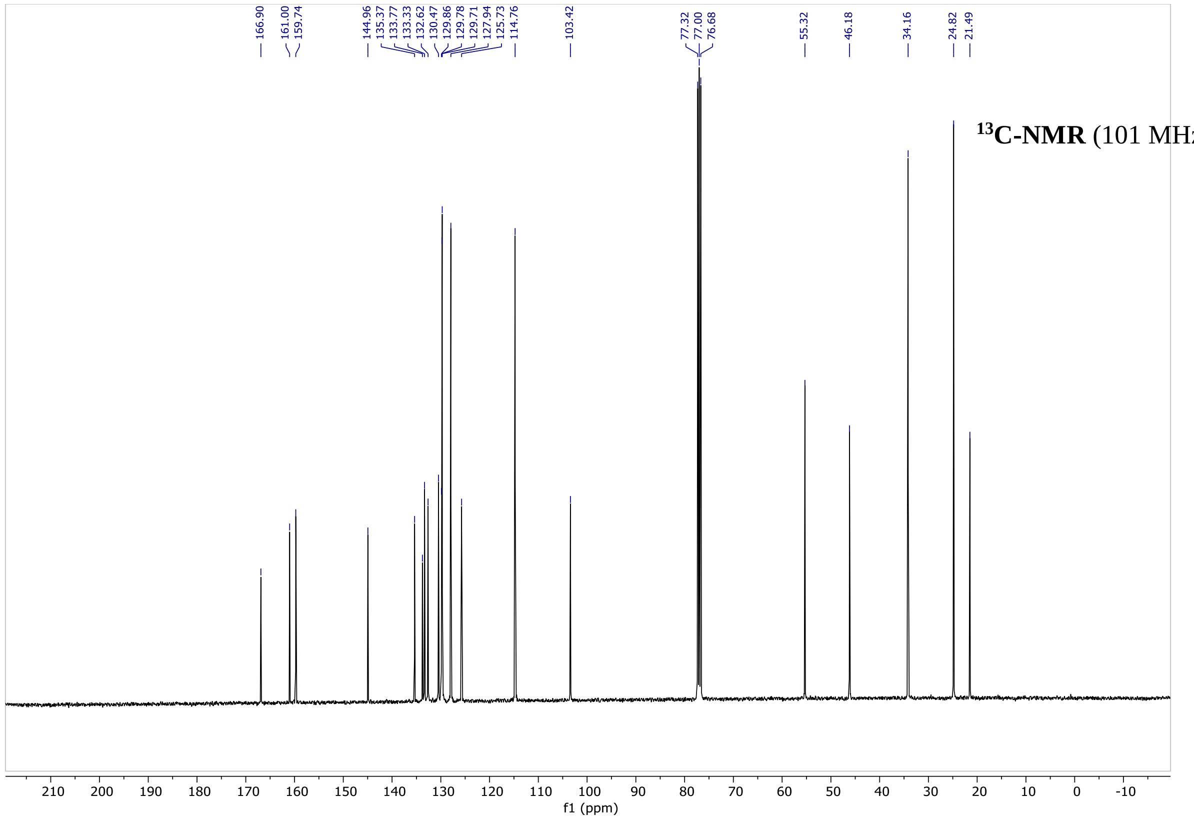

[13]**C-NMR** (101 MHz, $CDCl_3$)

(Z)-*N*-(1-vin-2-yl-2-cyclohexyl)-*N*-(4-methoxyphenyl)-4-methylbenzenesulfonamide-1,2-benziodoxol-3-(1*H*)-one (4h)

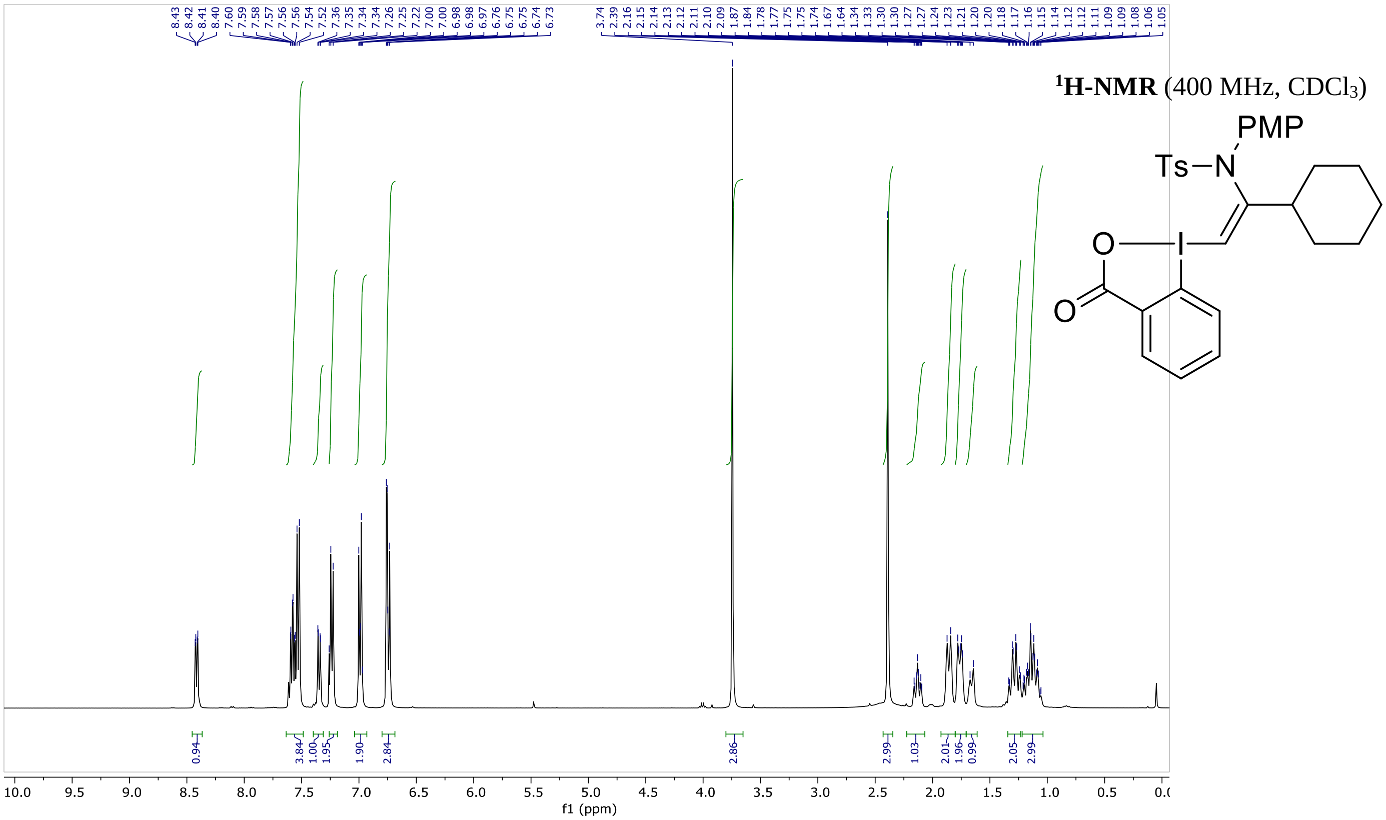

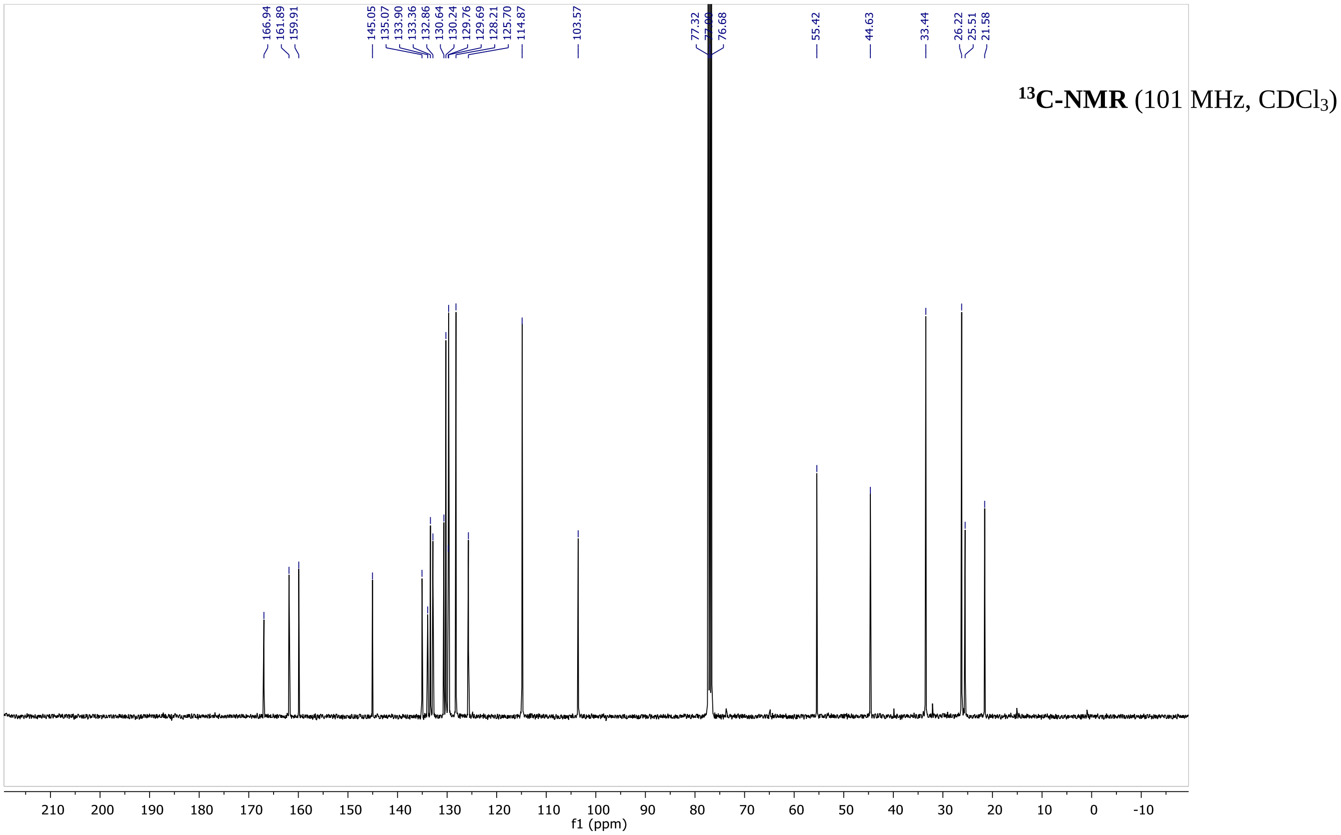

(Z)-*N*-(3-(benzyloxy)-3-methylbut-1-en-2-yl)-*N*-(4-methoxyphenyl)-4-methylbenzene sulfonamide-1,2-benziodoxol-3-(1*H*)-one (4i)

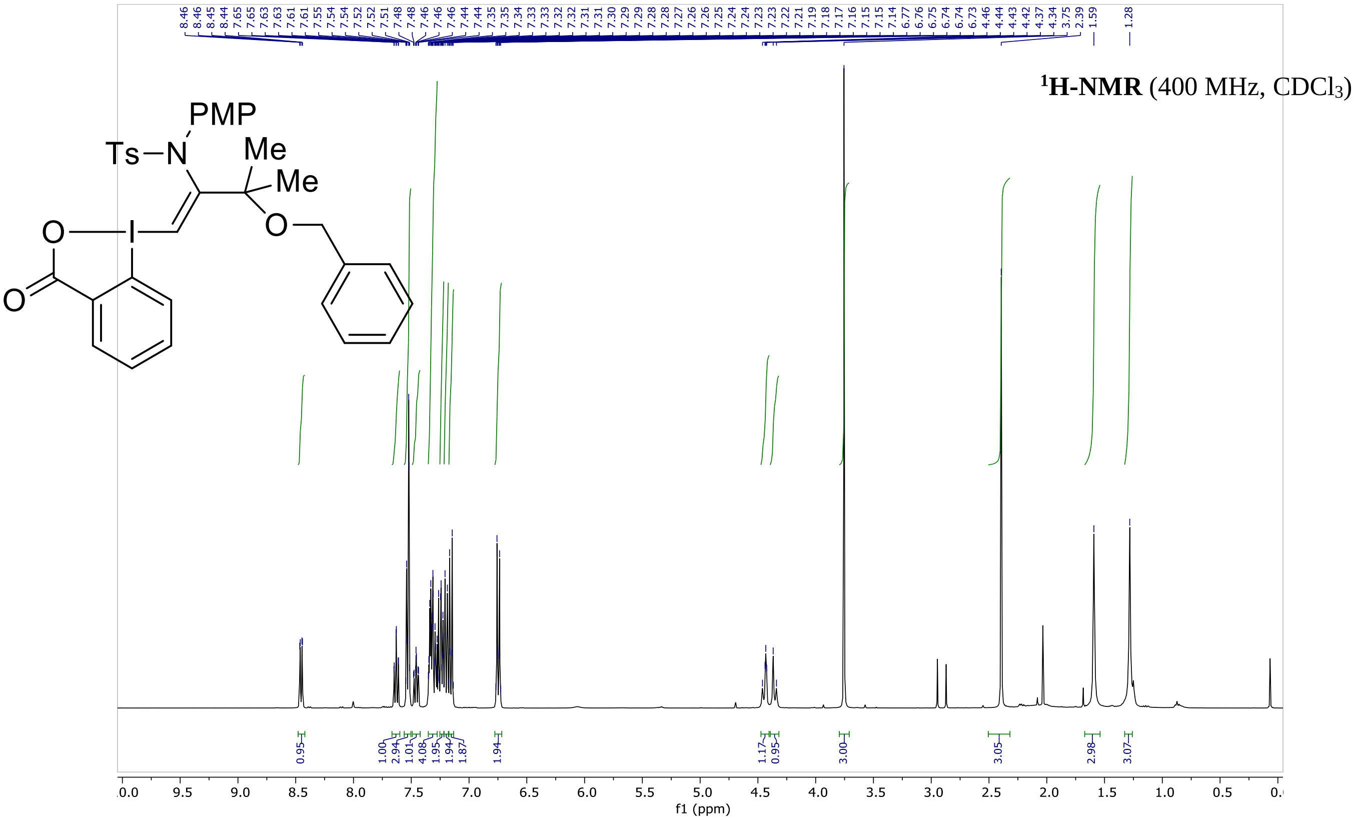

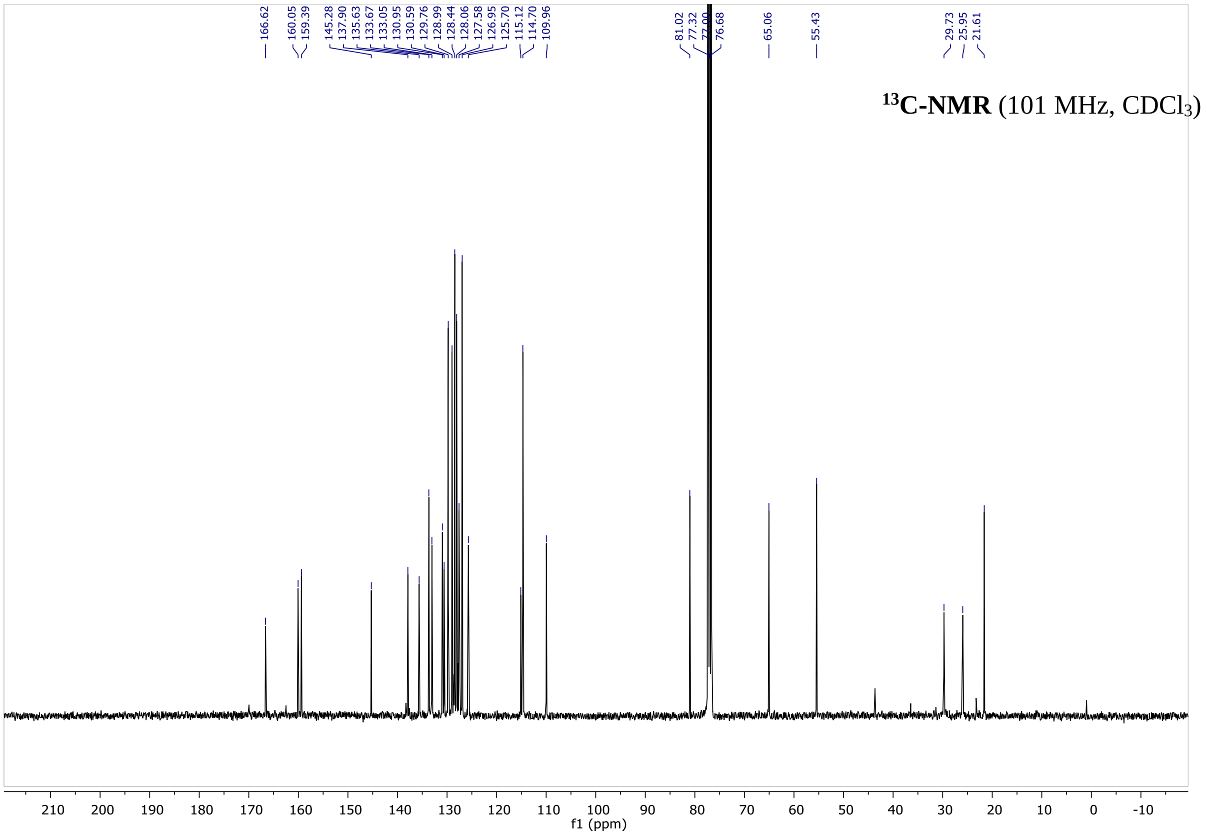

(Z)-*N*-(1-prop-1-en-2-yl)-N-(4-methoxyphenyl)-methylsulfonamide-1,2-benziodoxol-3-(1H)-one (4k)

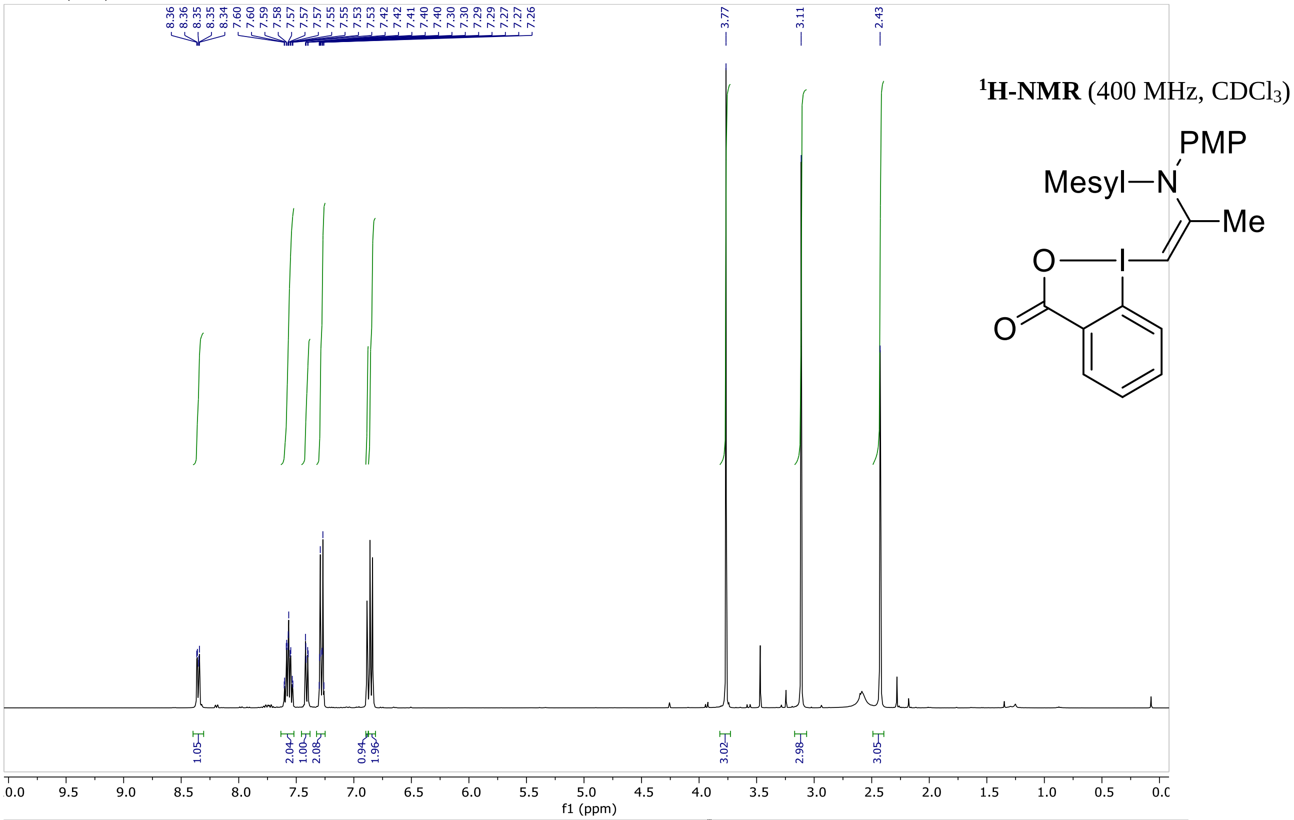

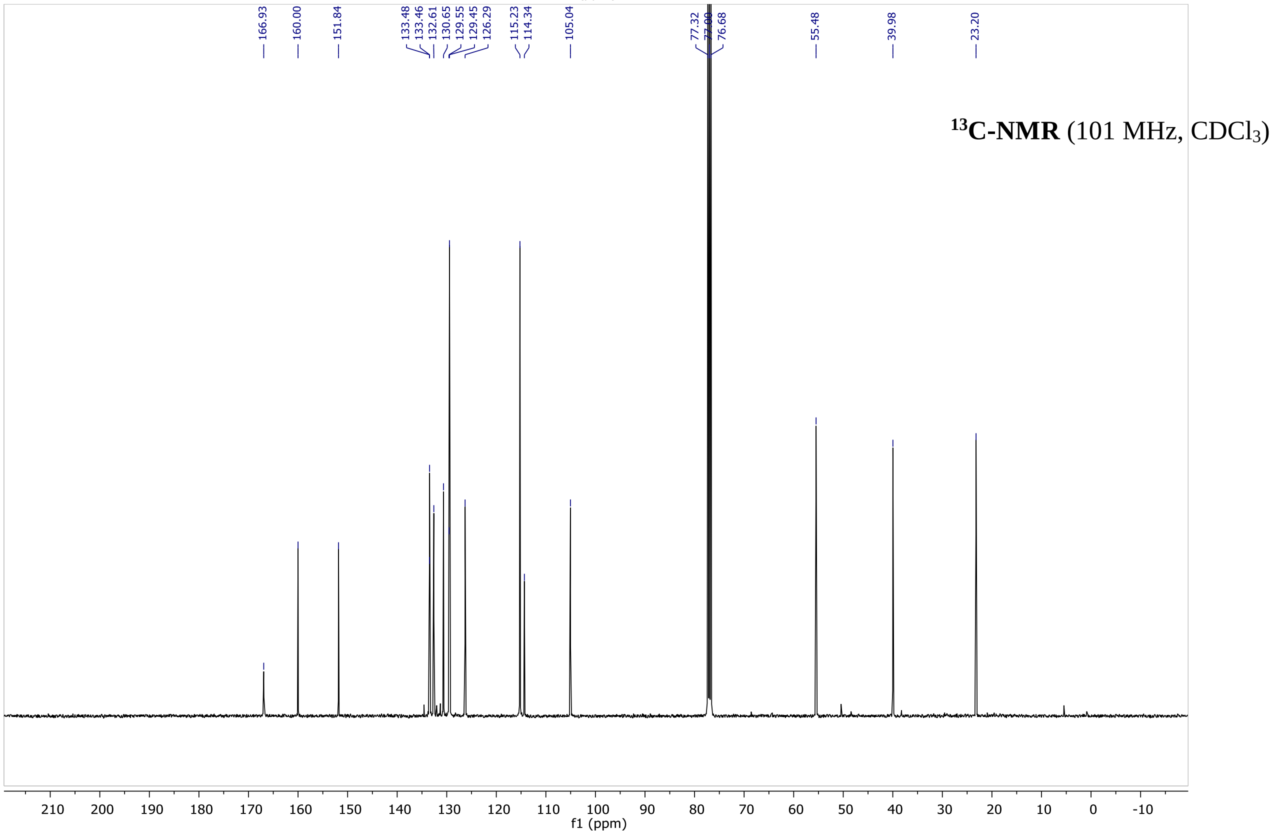

(Z)-*N*-(5-chloro-1-pent-1-en-2-yl)-N-(4-methoxyphenyl)-methylsulfonamide-1,2-benziodoxol -3-(1H)-one (4l)

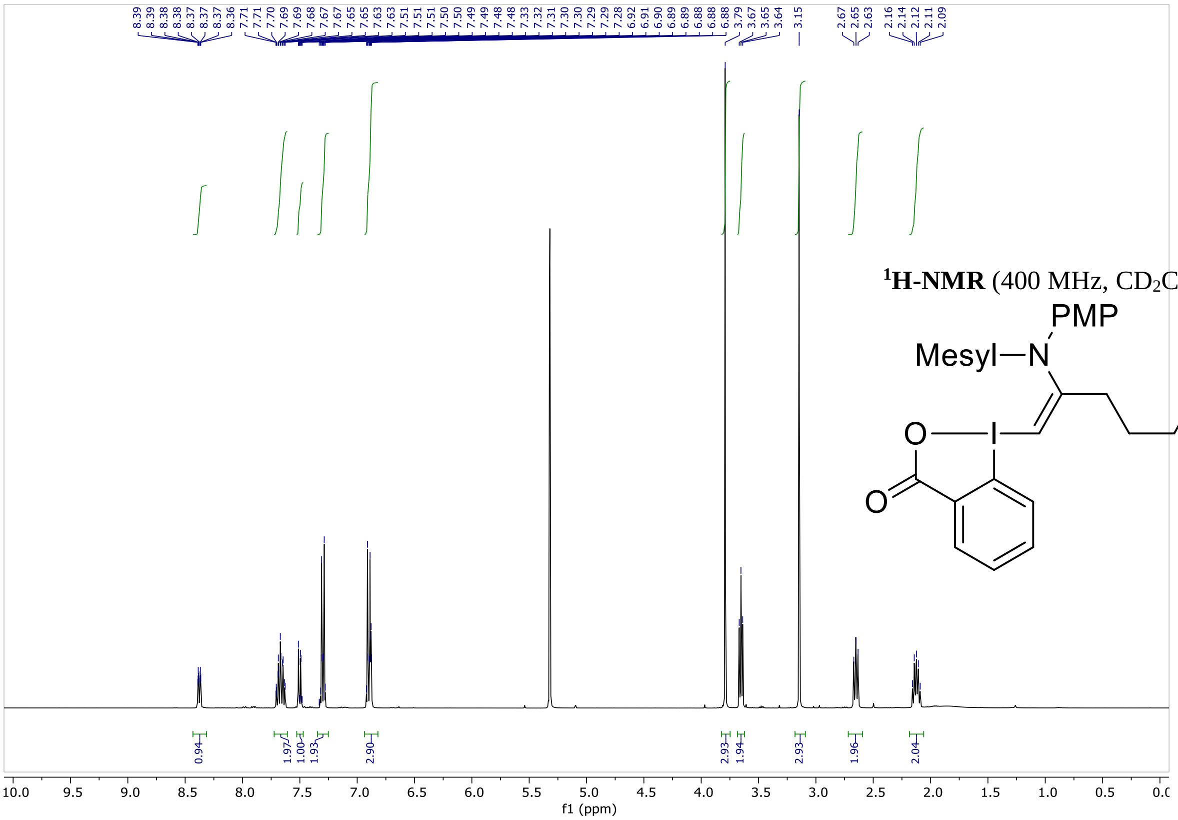

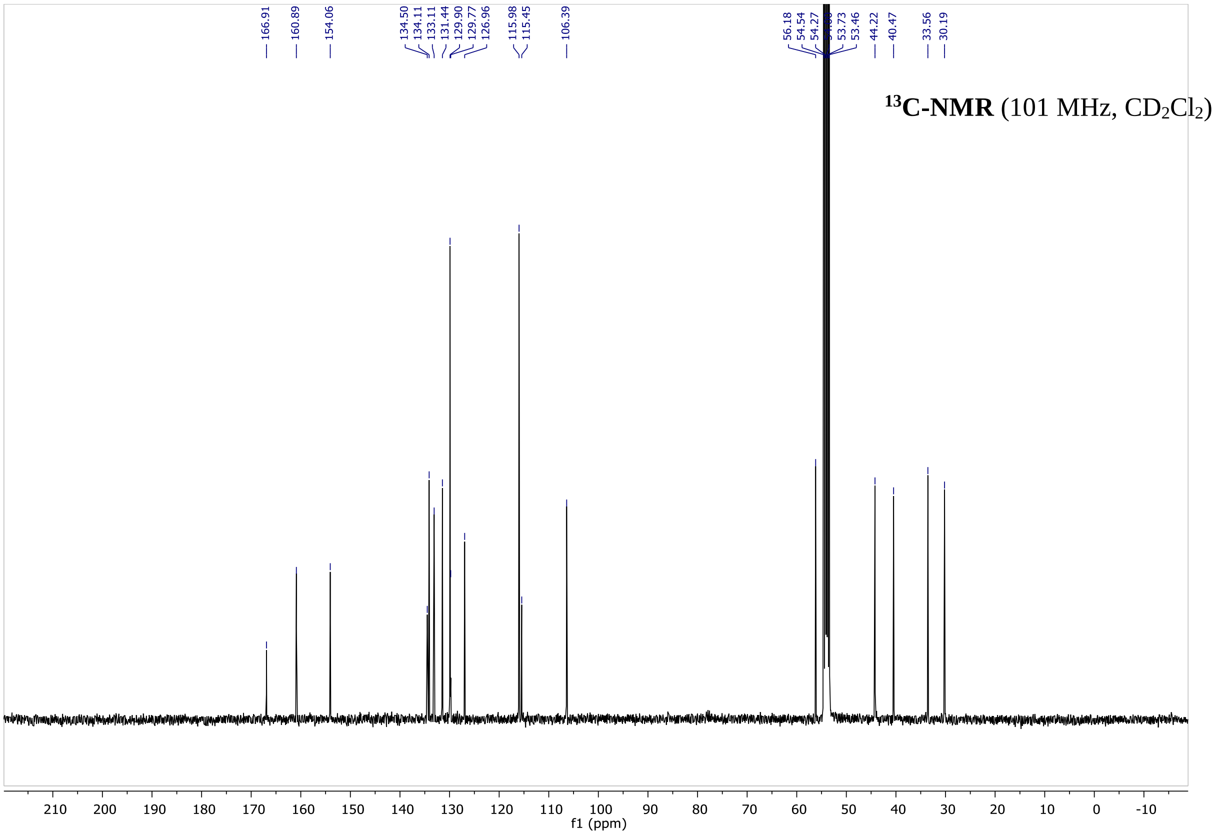

(Z)-*N*-(1-prop-1-en-2-yl)-*N*-(4-methoxyphenyl)-4-nitrobenzenesulfonamide-1,2-benziodoxol-3-(1*H*)-one (4m)

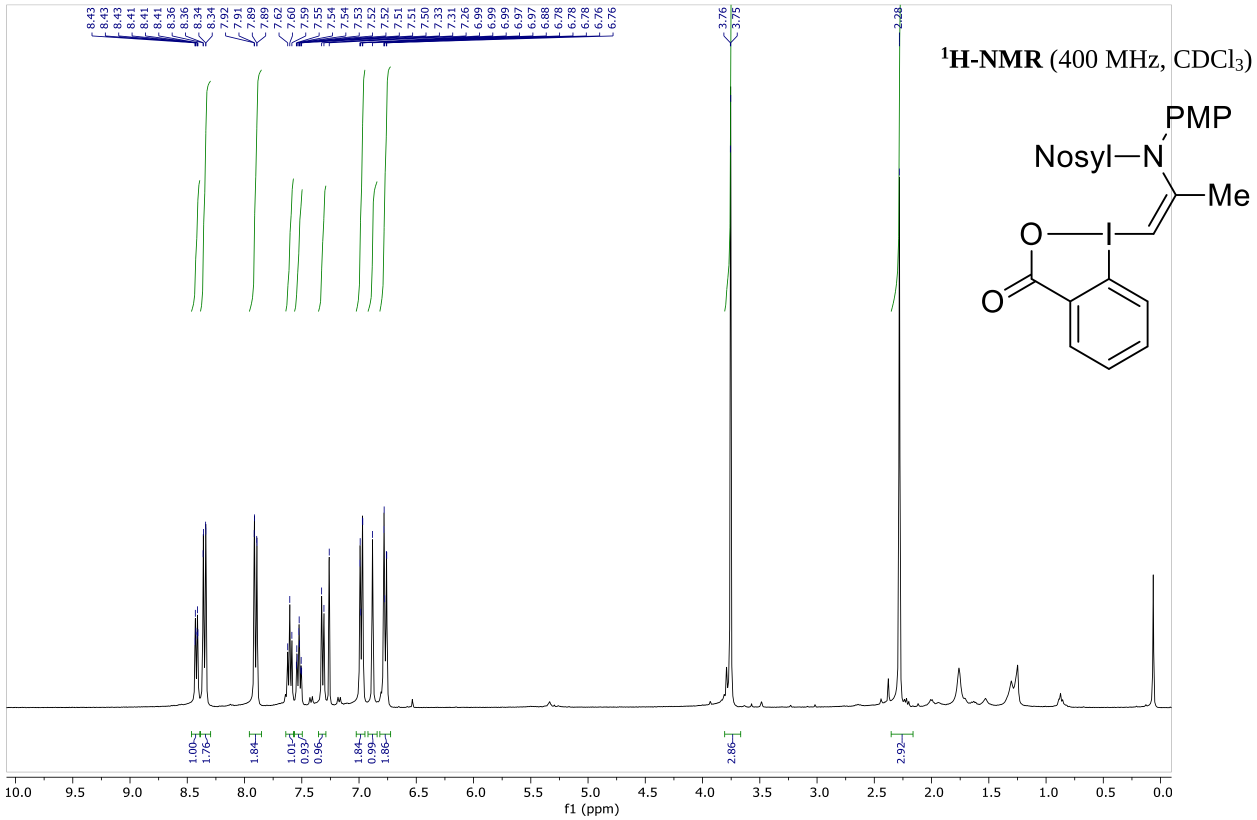

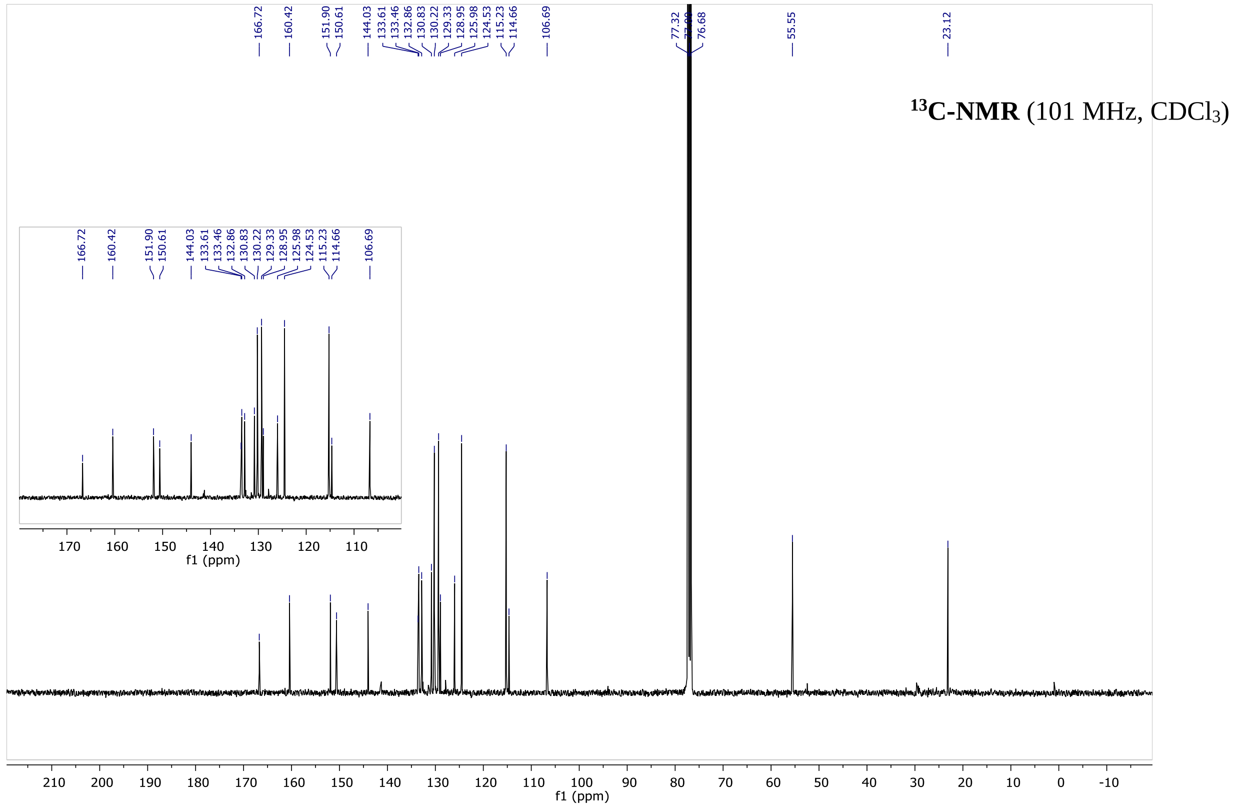

(Z)-*N*-(1-vin-2-yl)-N-(4-methoxyphenyl)-4-nitrobenzenesulfonamide-1,2-benziodoxol-3-(1*H*)-one (4n)

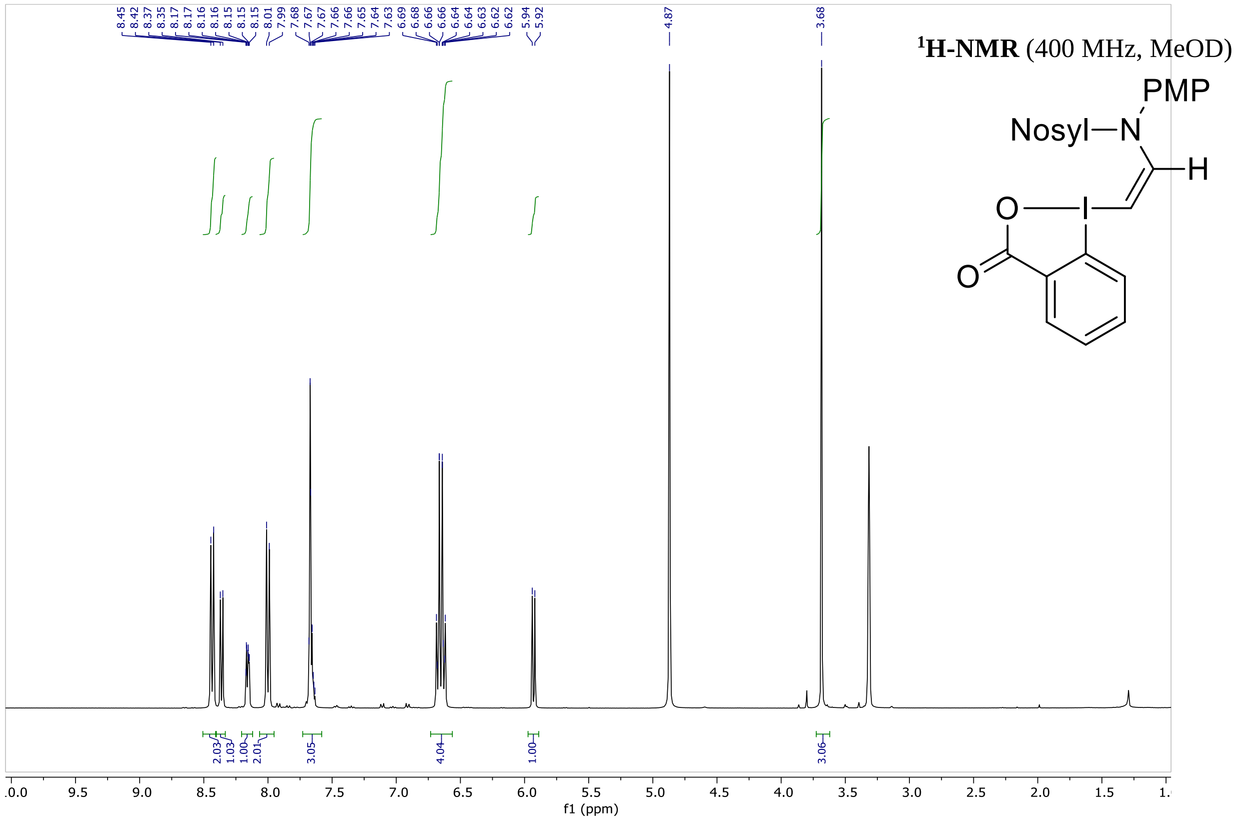

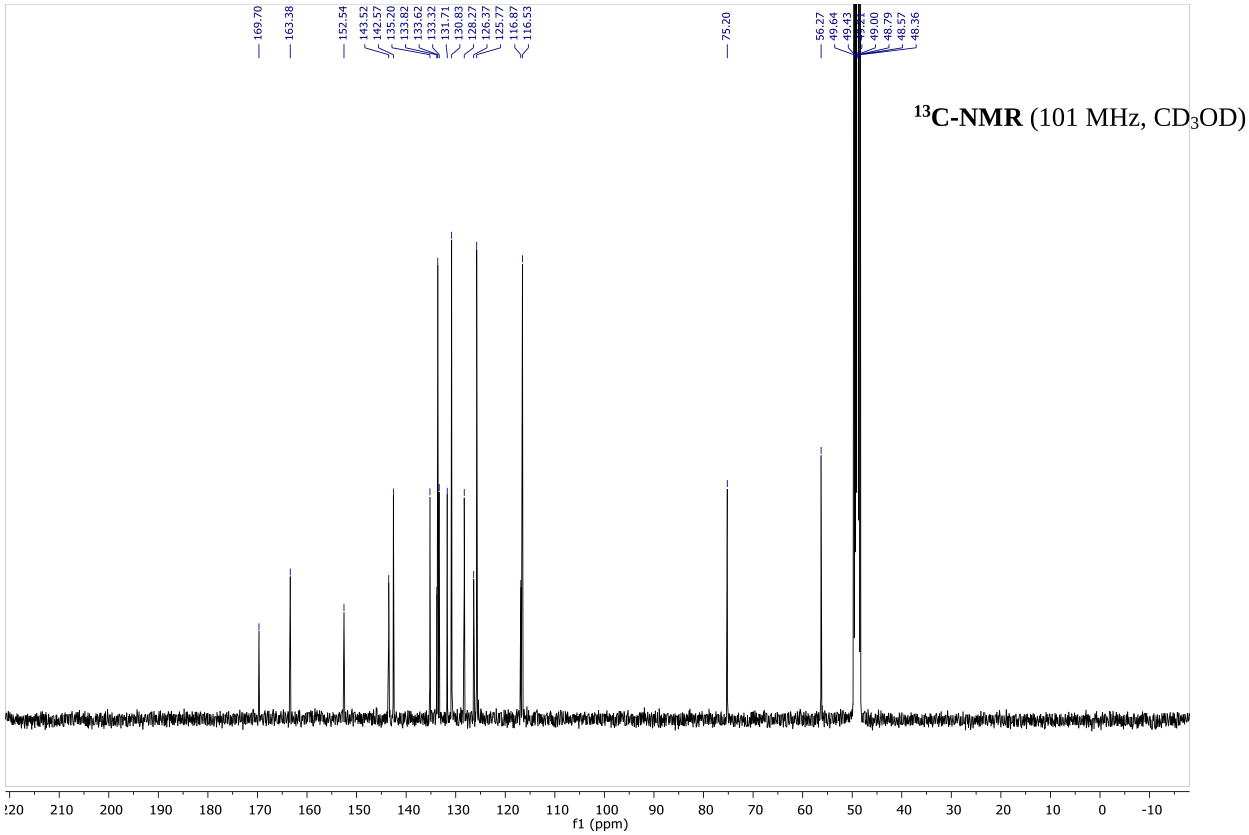

(Z)-(1-vinyl-2-oxy)-4-methylbenzene-1,2-benziodoxol-3-(1*H*)-one (5a)

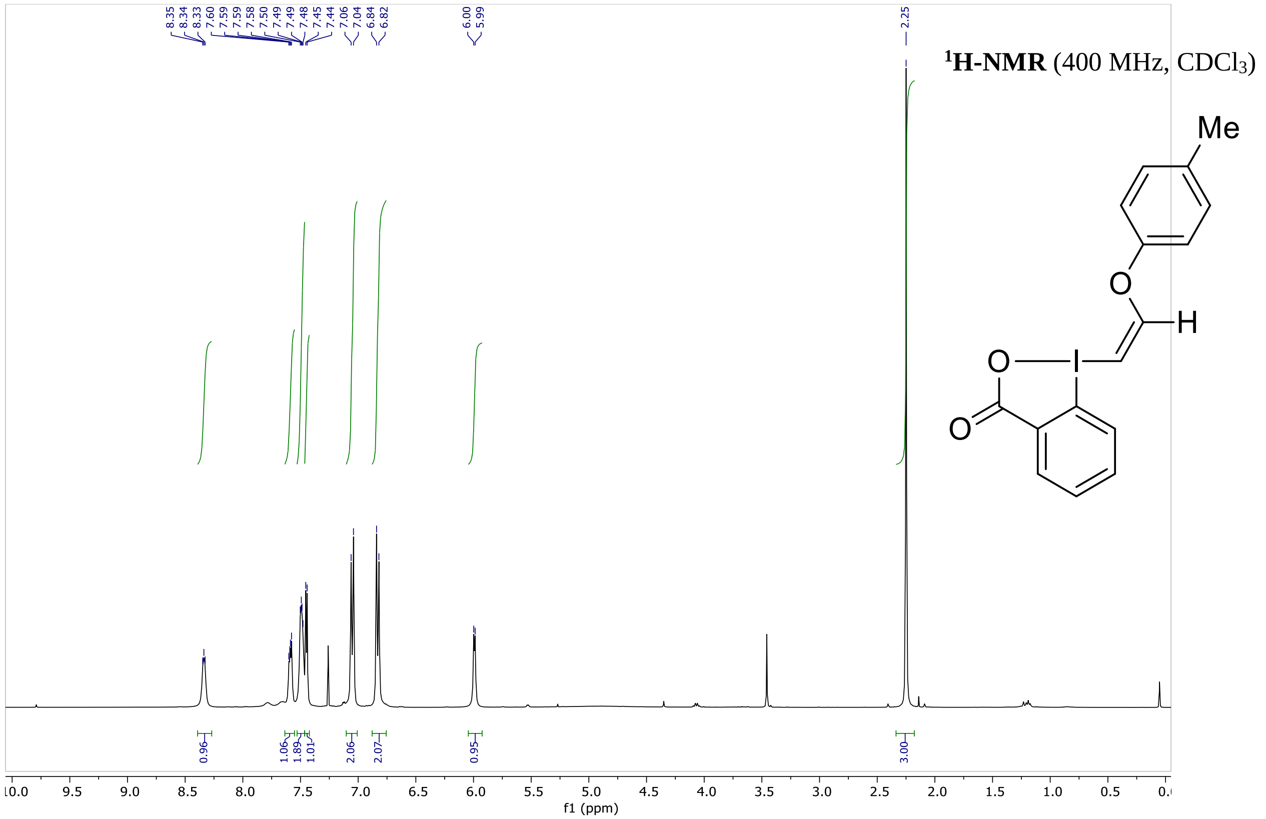

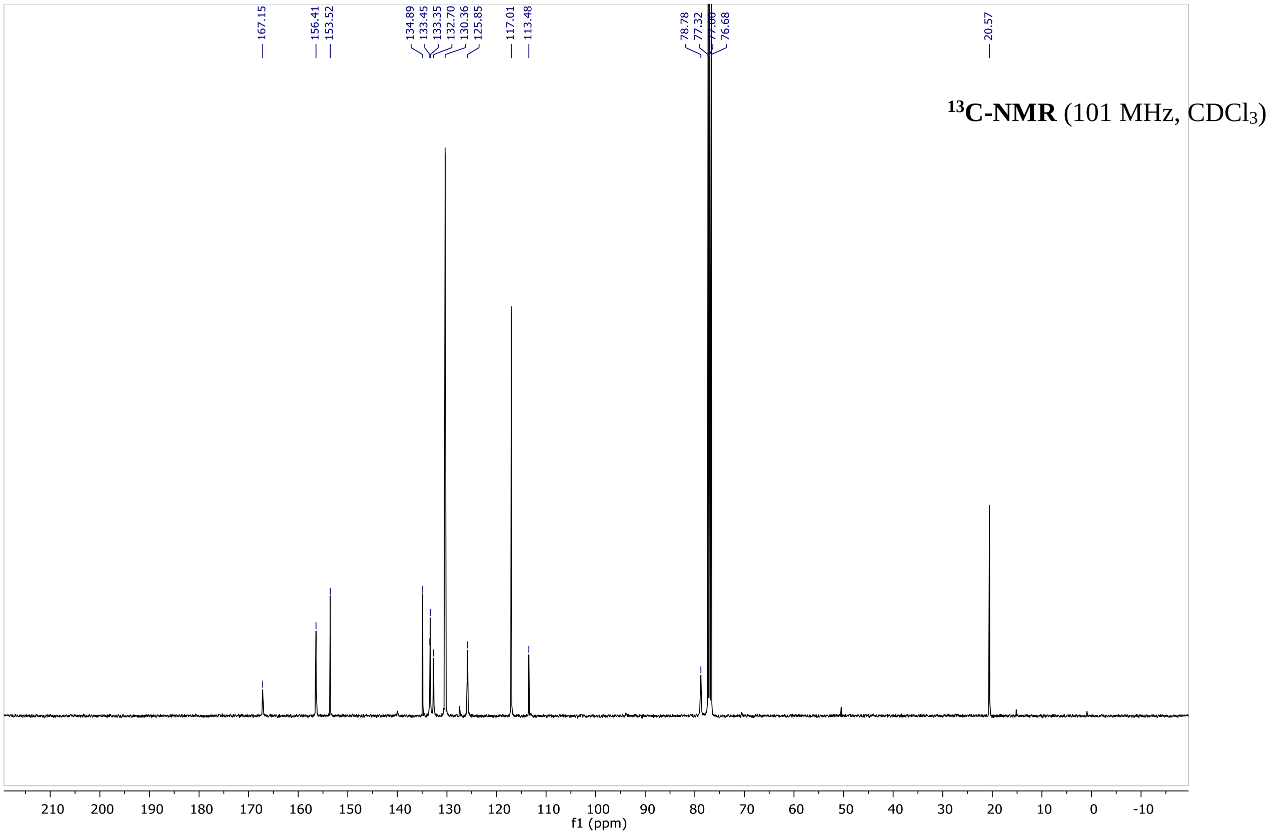

(Z)-(1-prop-1-en-2-yl-2-oxy)-4-methylbenzene-1,2-benziodoxol-3-(1*H*)-one (5b)

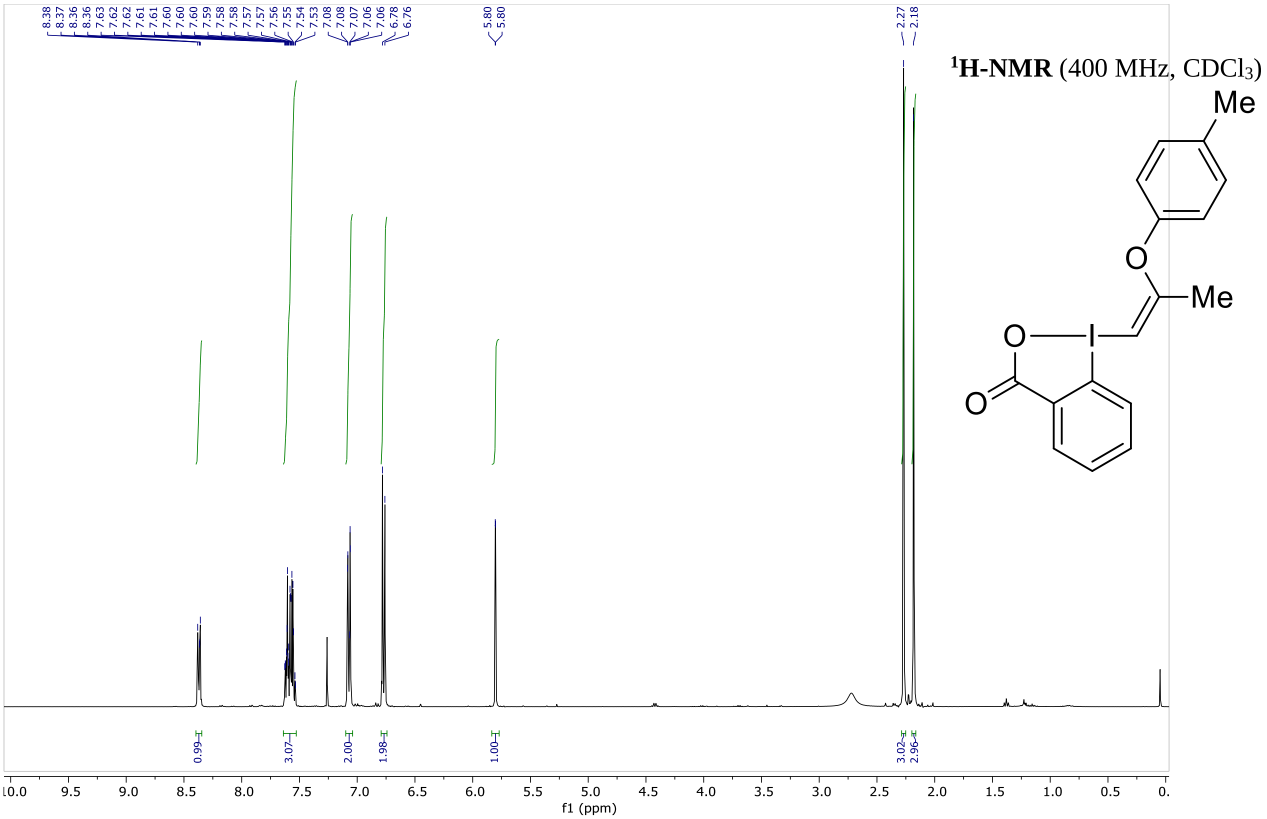

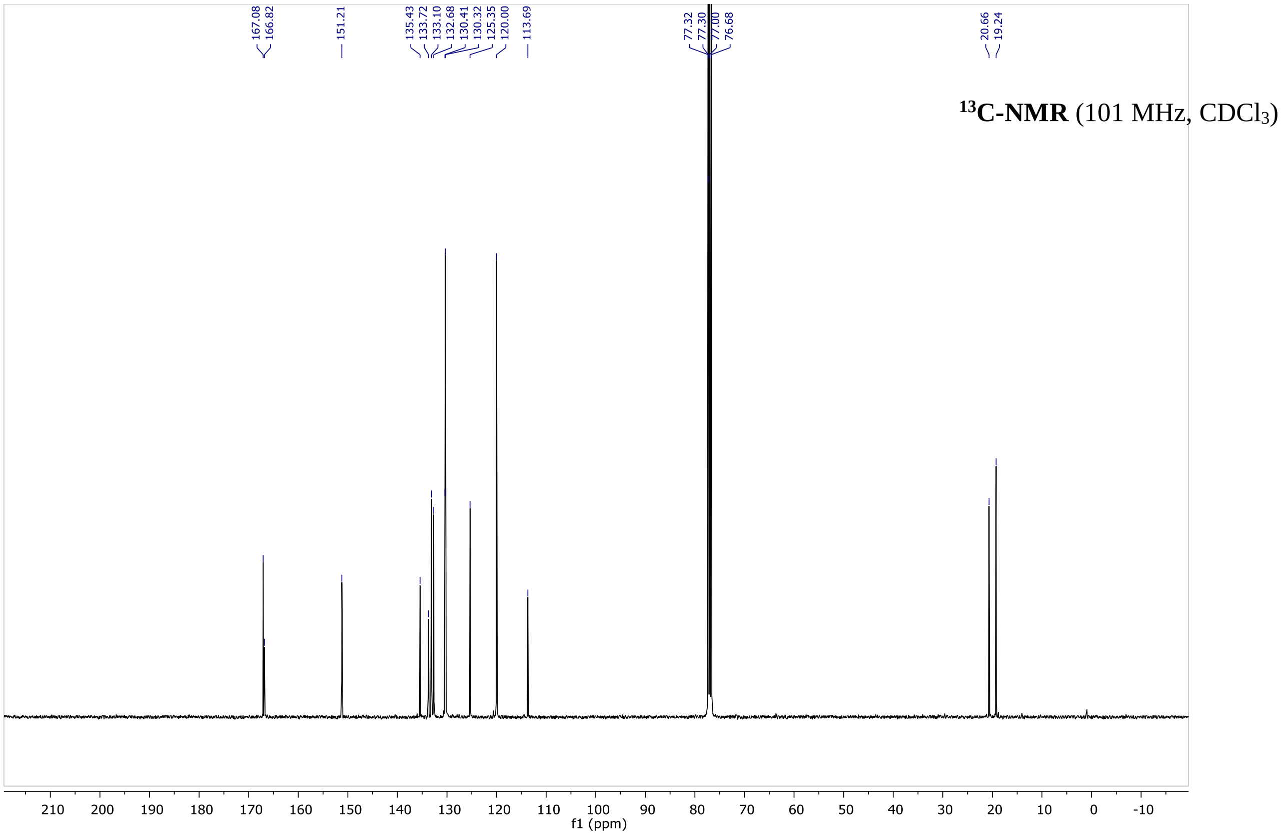

(Z)-(1-pent-1-en-2-yl-2-oxy)-4-methylbenzene-1,2-benziodoxol-3-(1*H*)-one (5c)

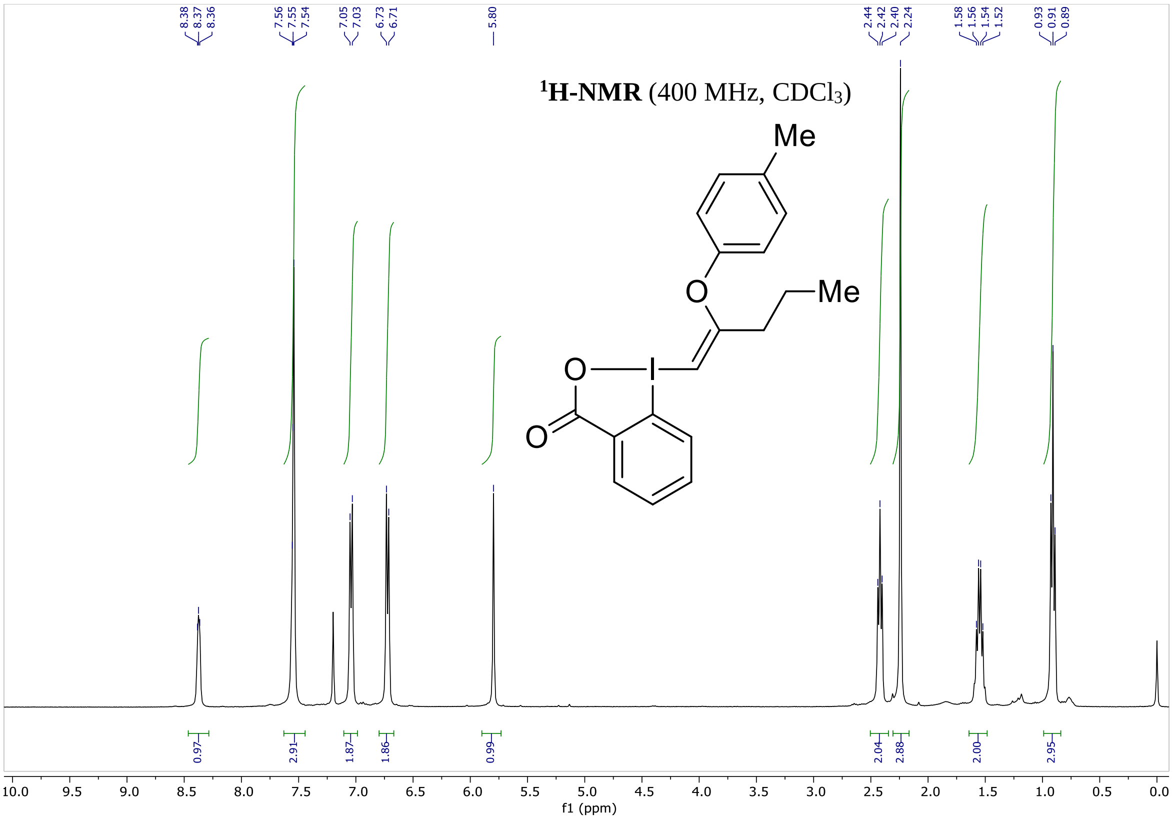

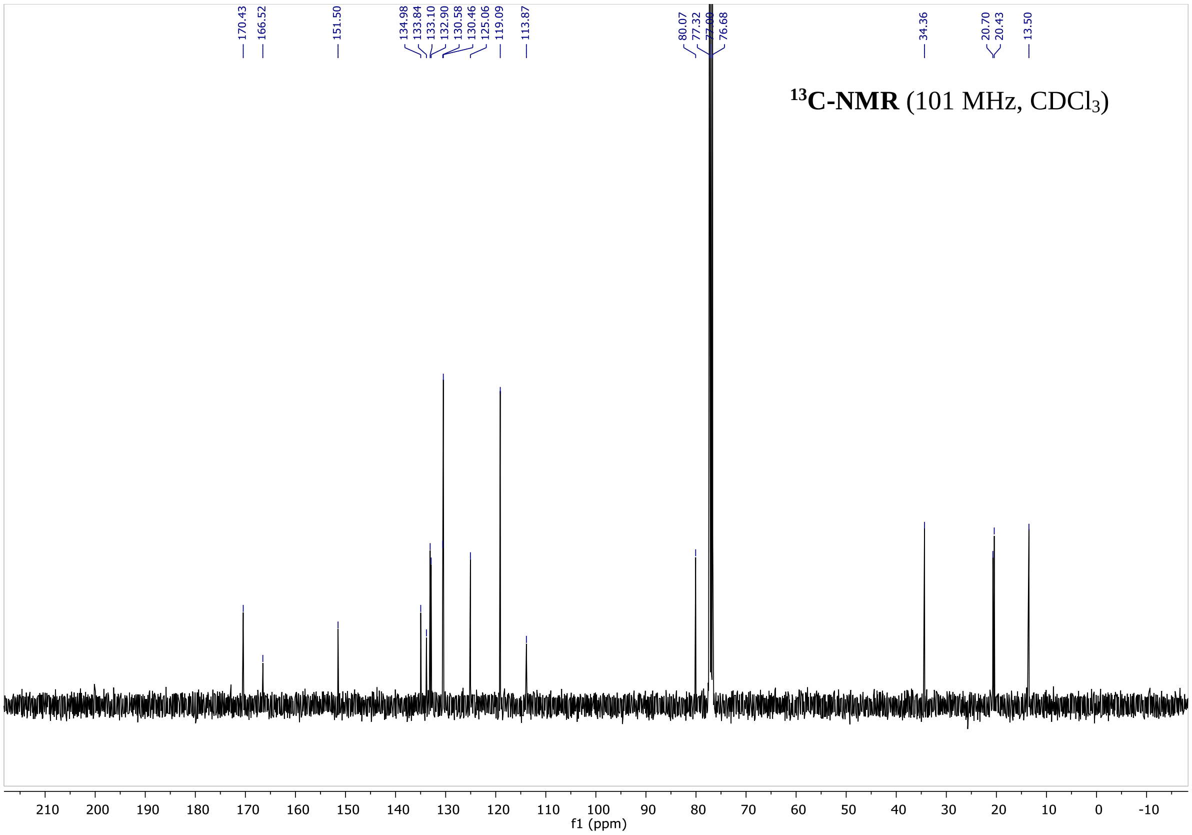

(Z)-(5-chloro-1-pent-1-en-2-yl-2-oxy)-4-methylbenzene-1,2-benziodoxol-3-(1*H*)-one (5d)

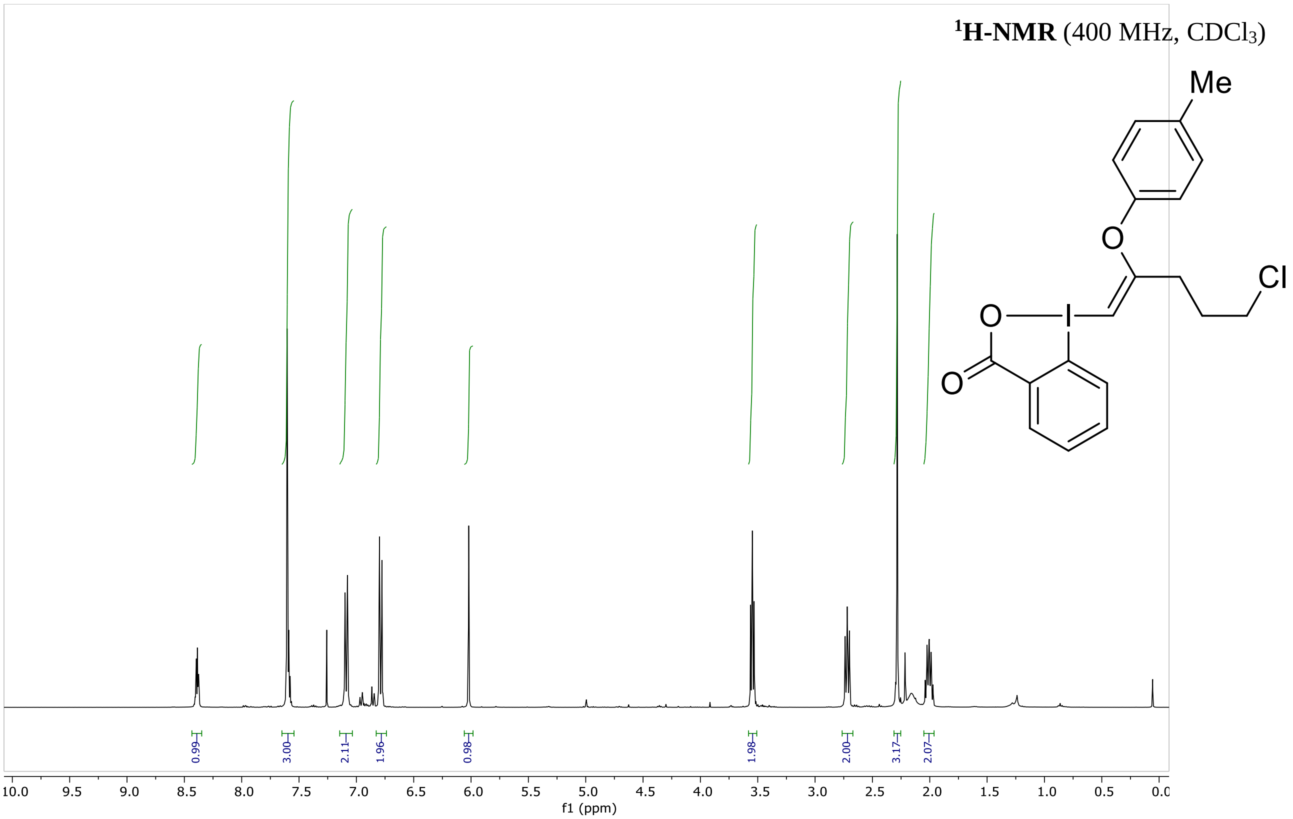

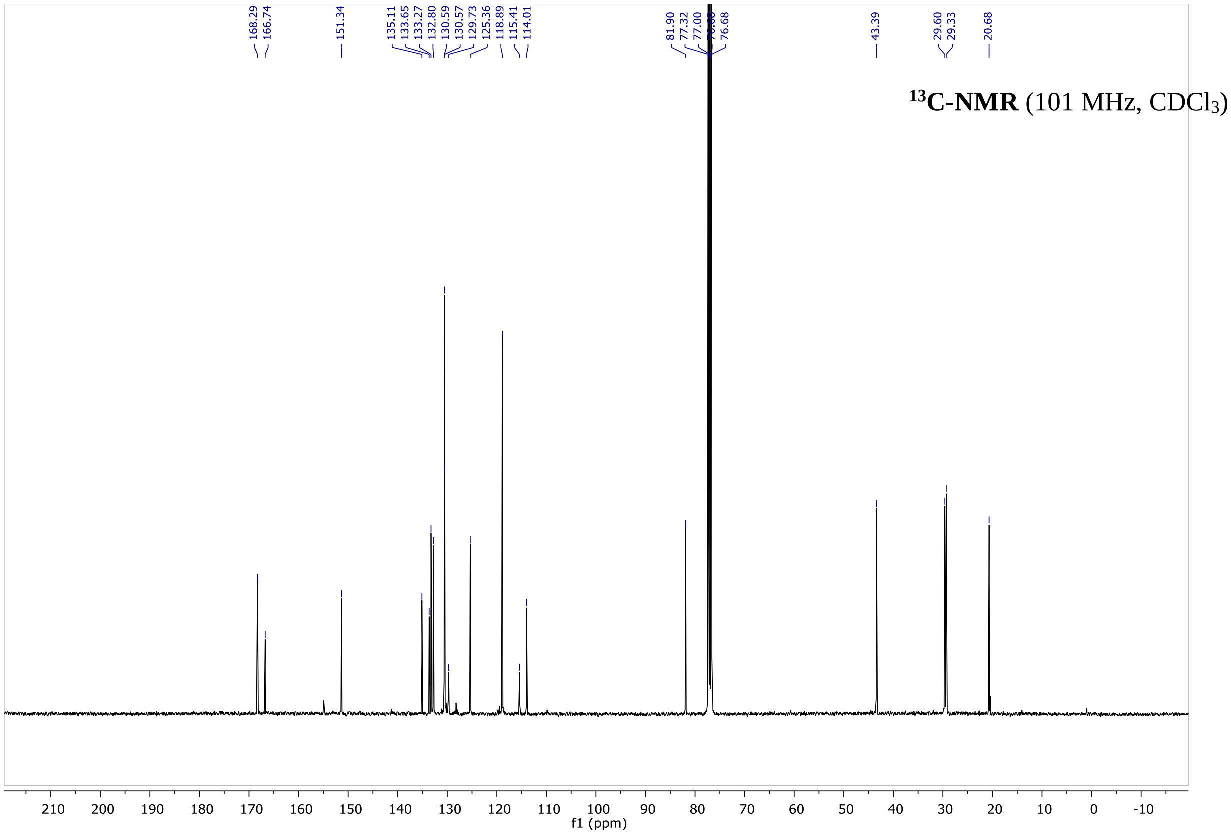

(Z)-(1-prop-1-en-2-yl-2-oxy)-3,5-dibromobenzene-1,2-benziodoxol-3-(1*H*)-one (5e)

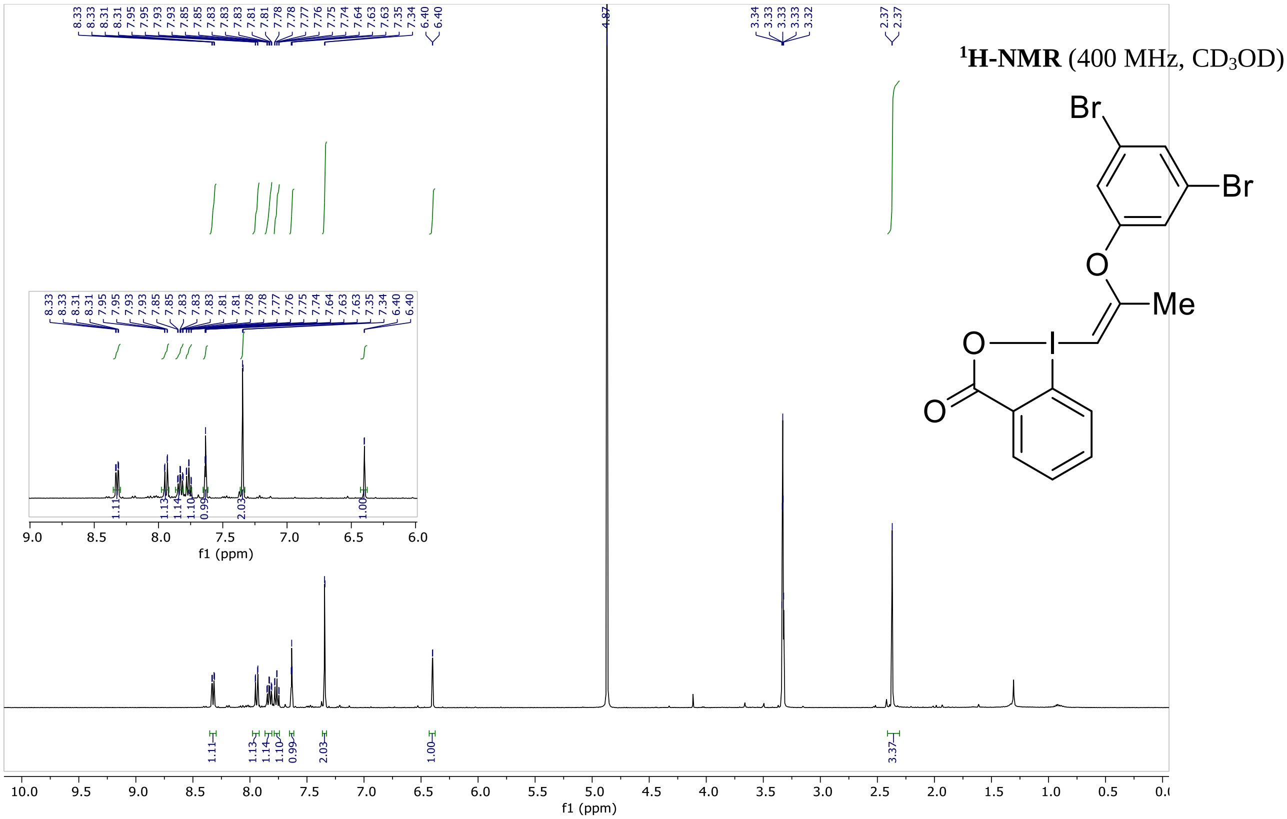

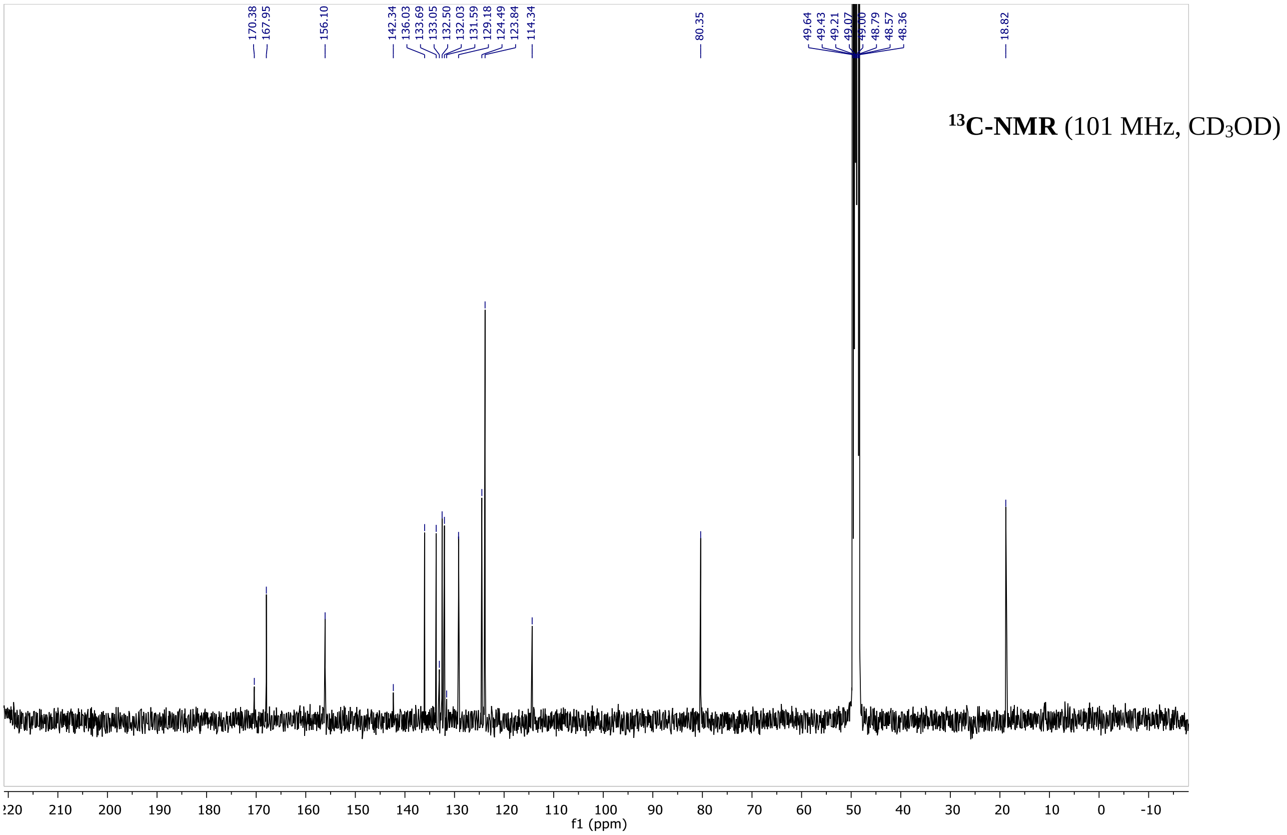

(Z)-(1-pent-1-en-2-yl-2-oxy)-2,3,4,5-pentafluorobenzene-1,2-benziodoxol-3-(1*H*)-one (5f)

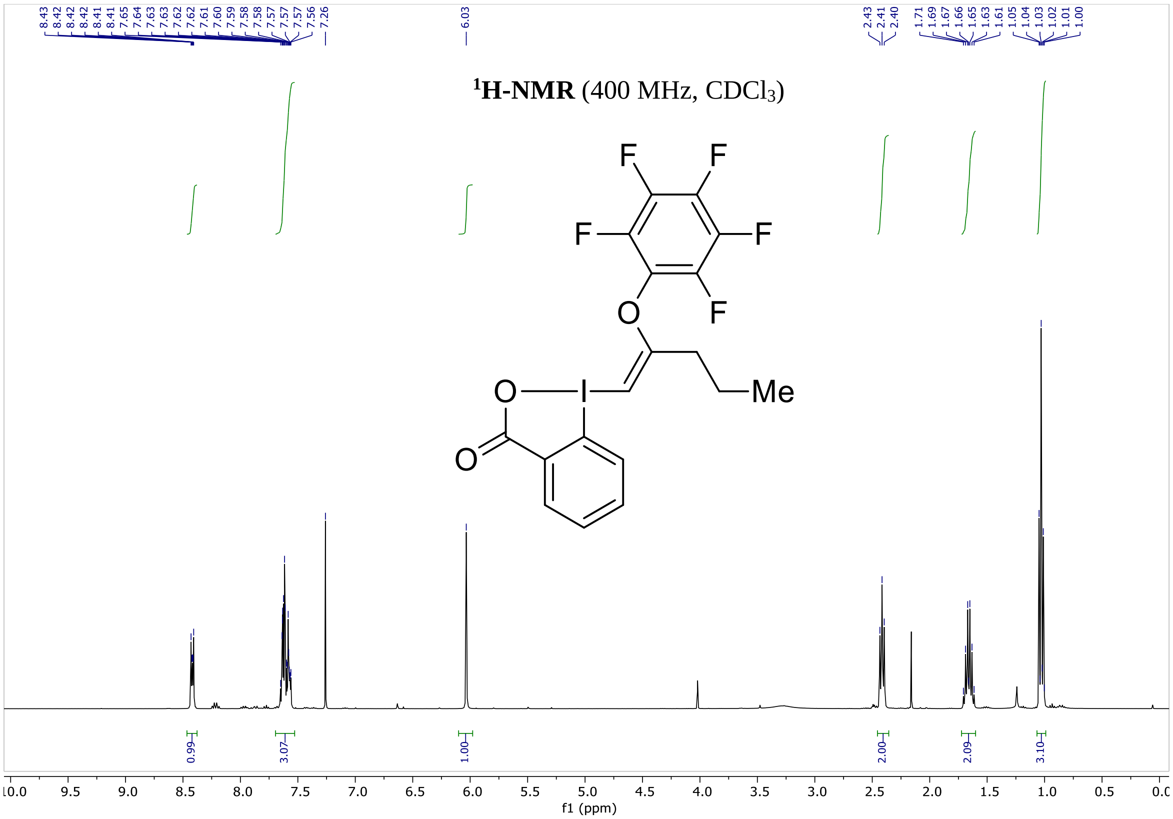

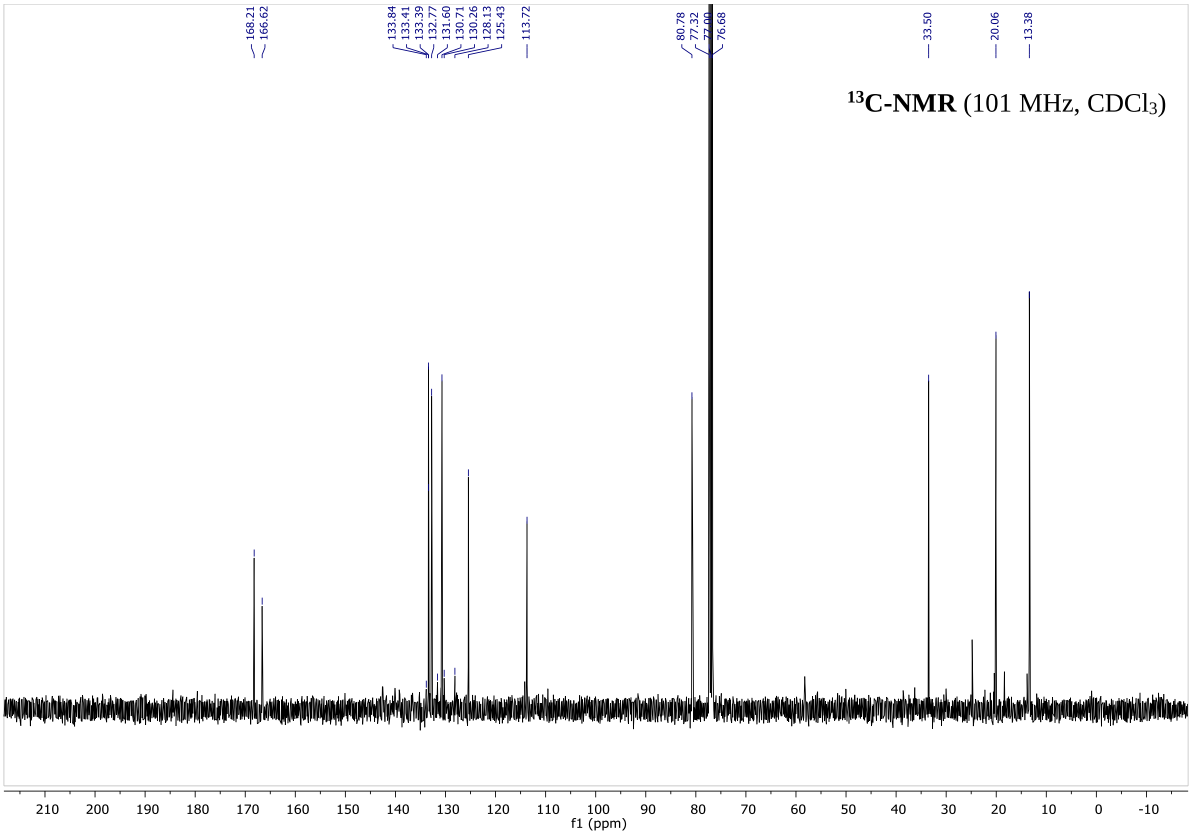

(Z)-*N*-(5-chloro-1-pent-1-en-2-yl)-*N*-Sulfaphenazole-1,2-benziodoxol-3-(1*H*)-one (6)

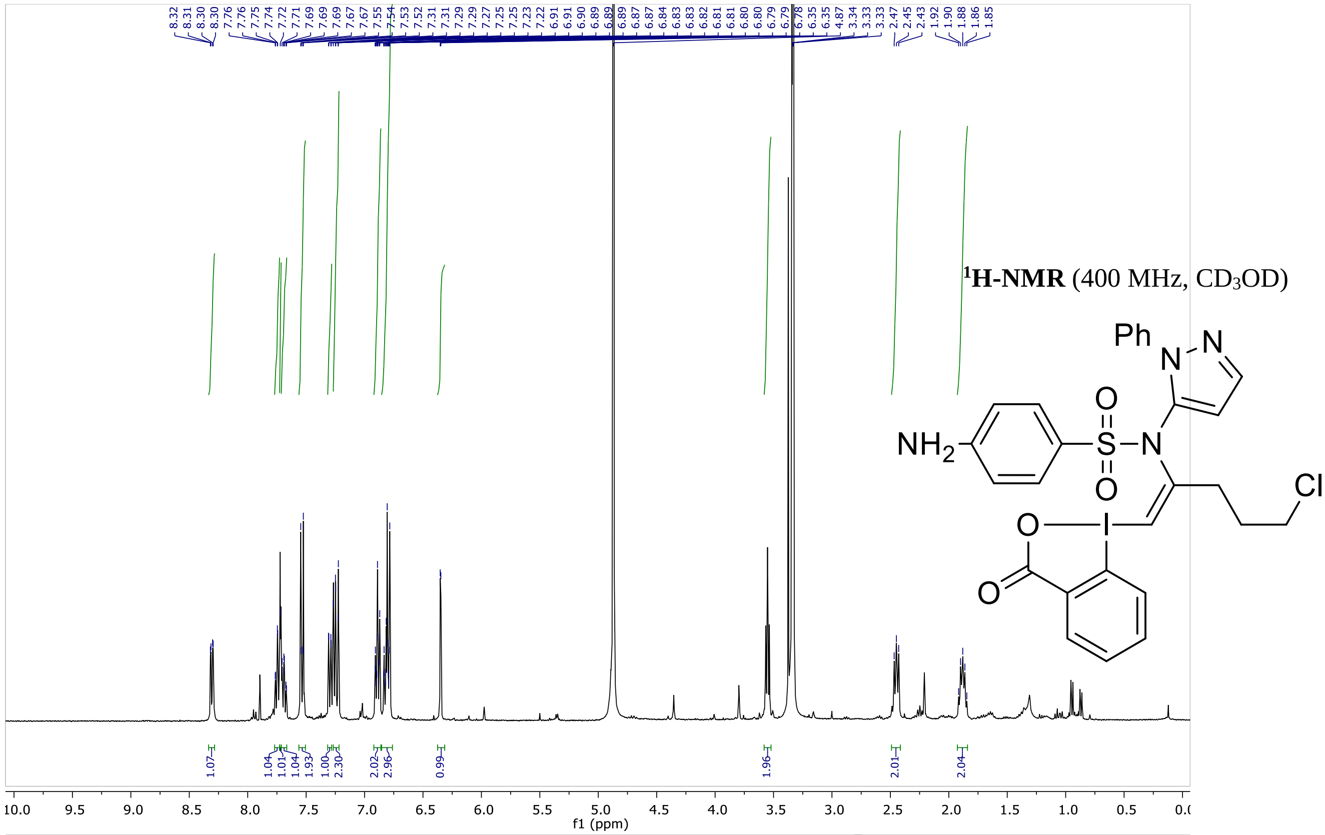

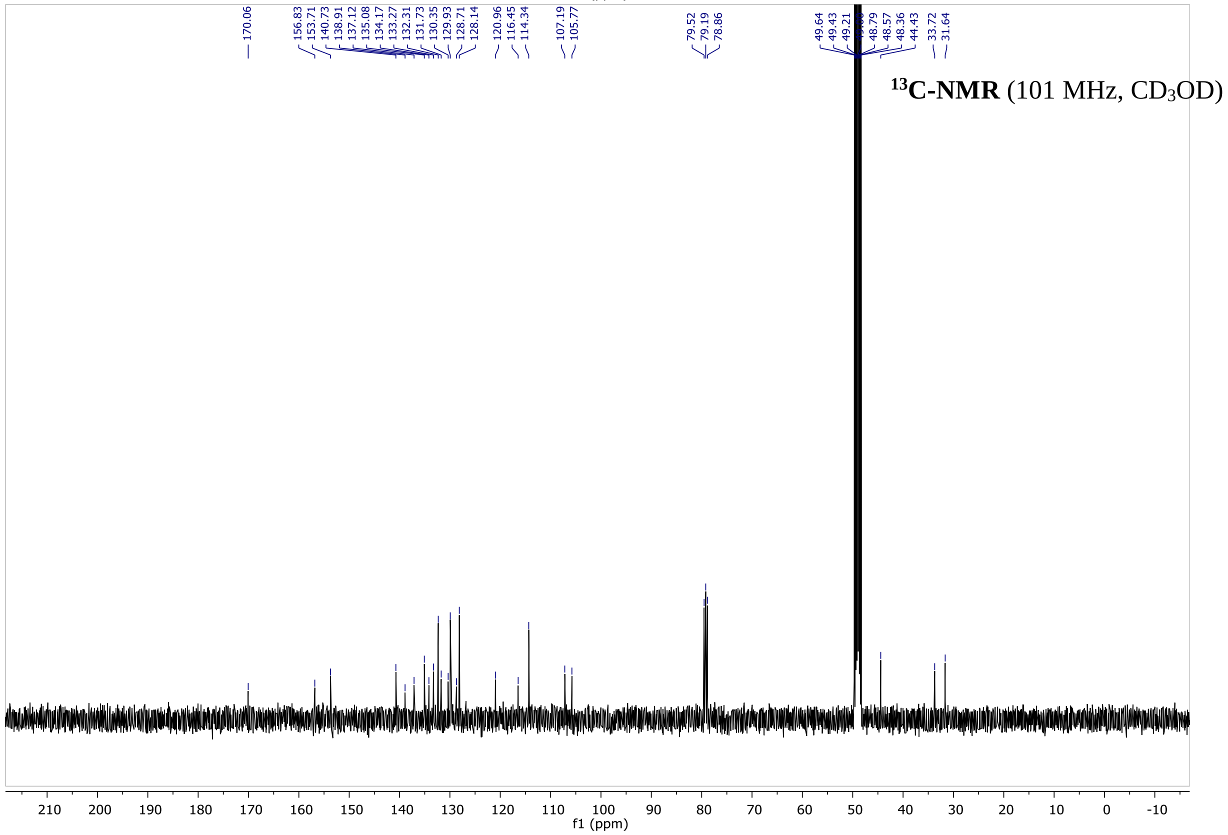

(Z)-(1-prop-1-en-2-yl)-2-Tyrosine-1,2-benziodoxol-3-(1*H*)-one (7)

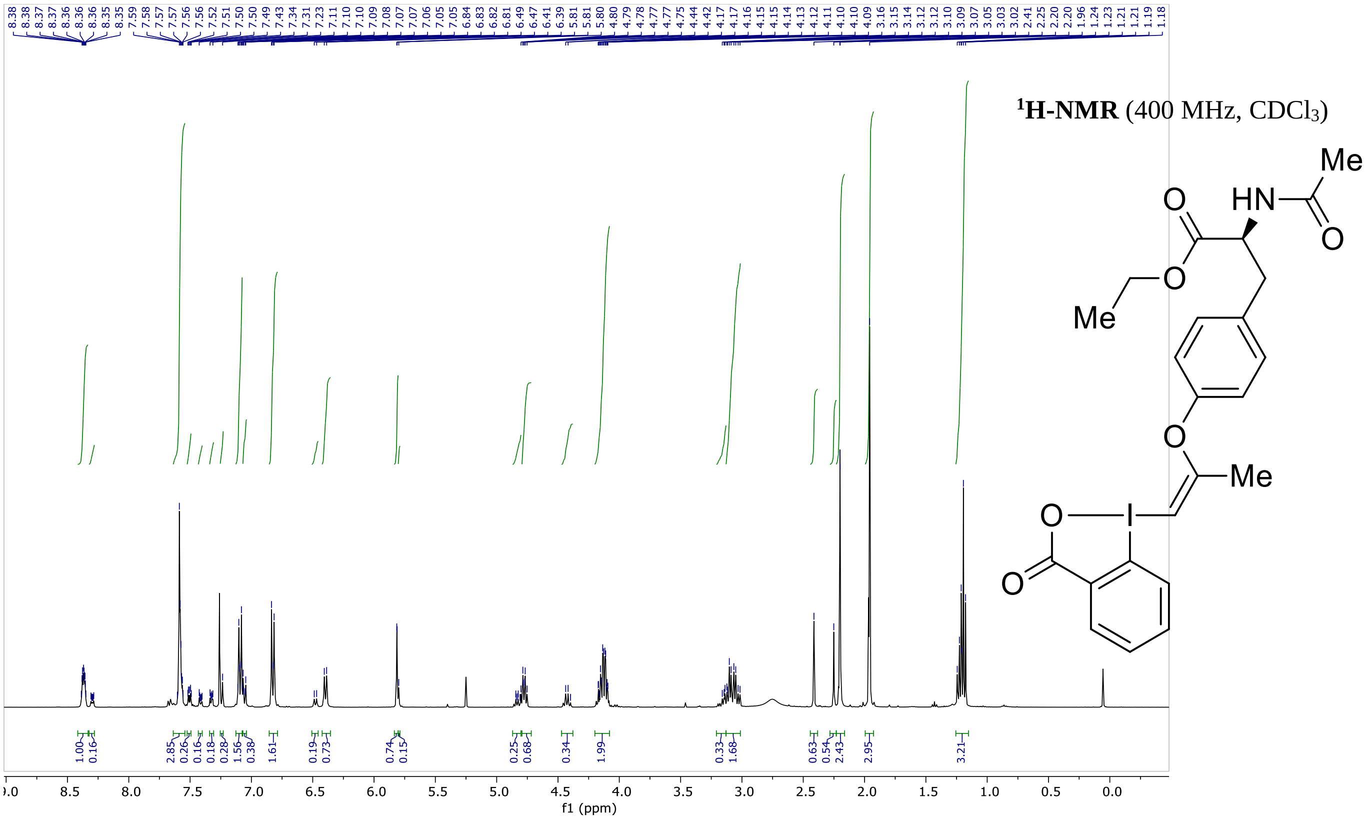

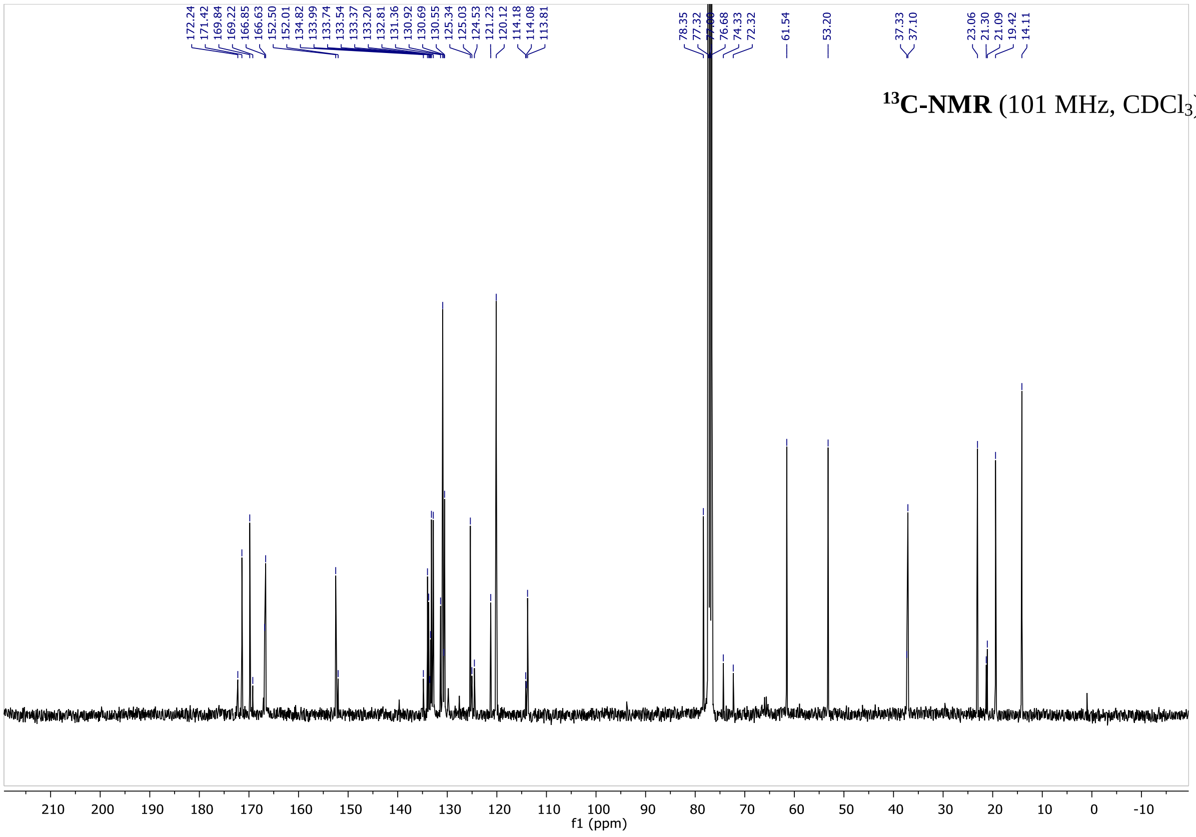

(Z)-(5-chloro-1-pent-1-en-2-yl)-2-α-Tocopherol-1,2-benziodoxol-3-(1*H*)-one (8)

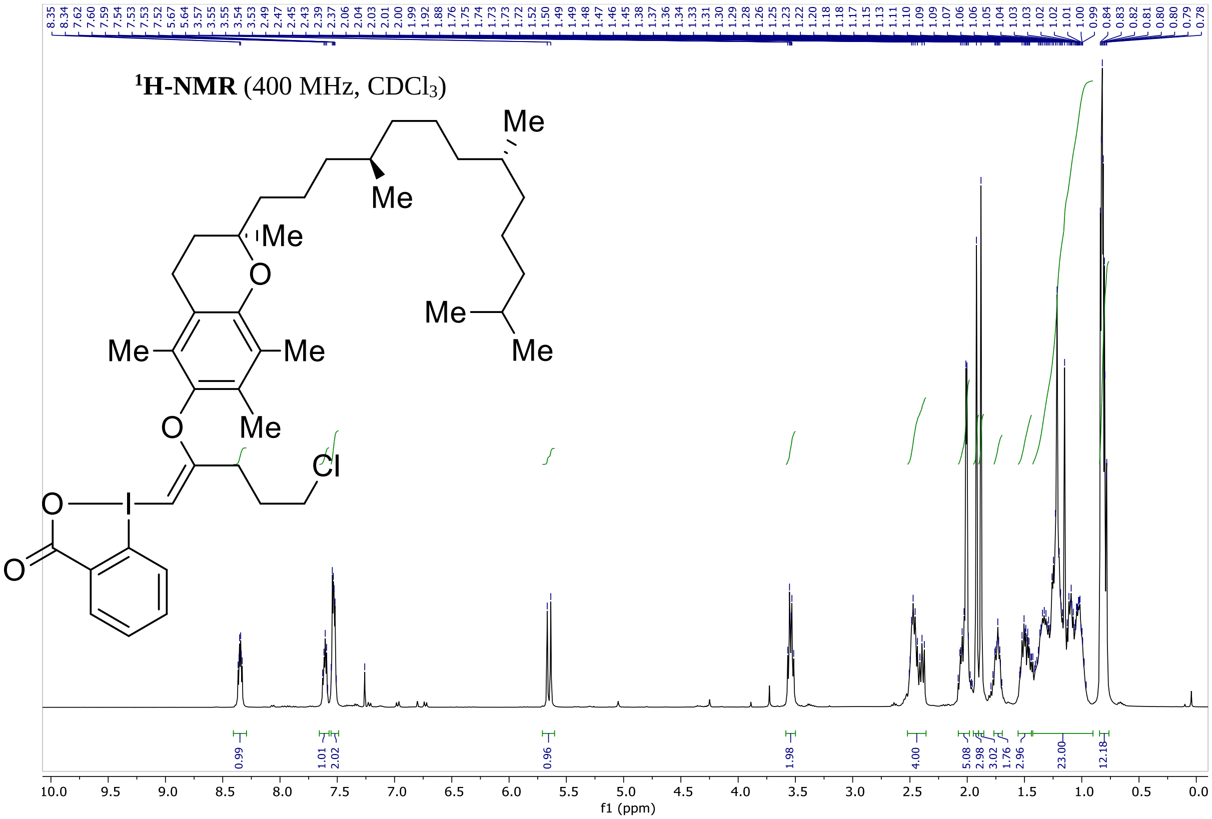

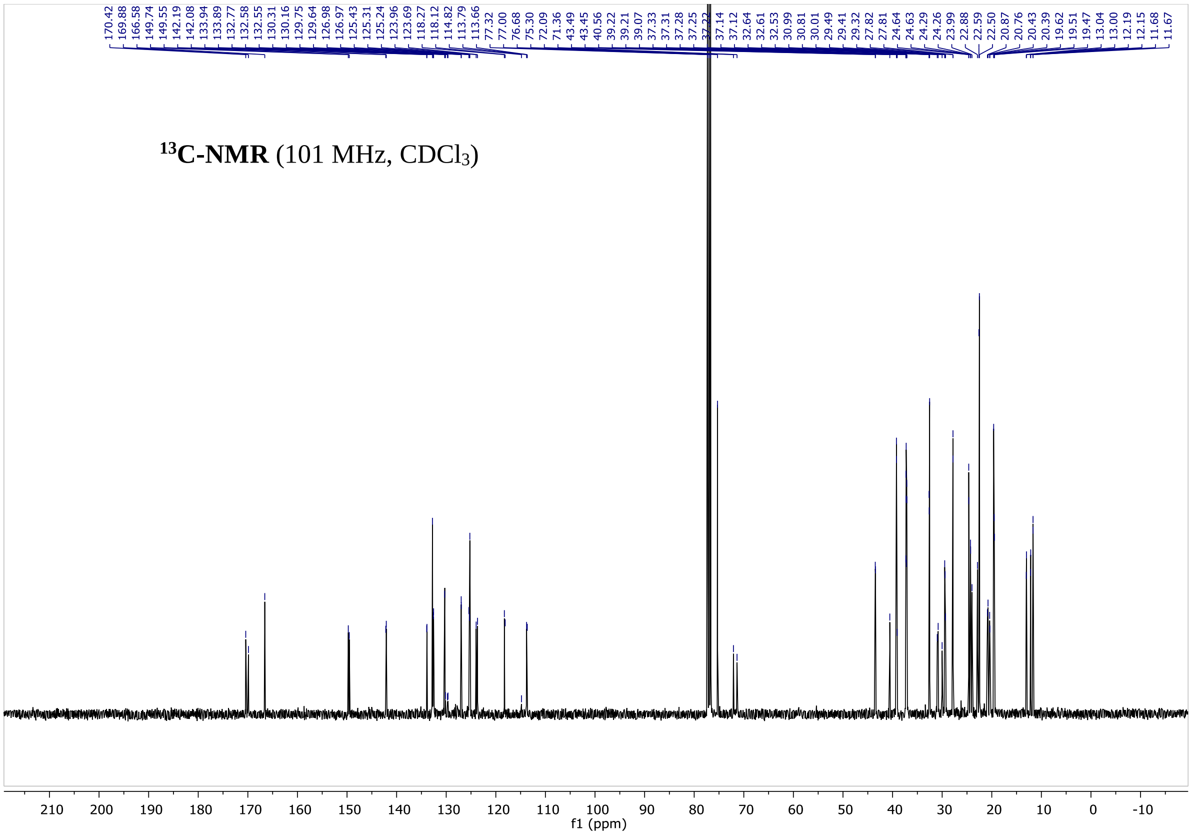

(Z)-(1-prop-1-en-2-yl)-2-Capsaicin-1,2-benziodoxol-3-(1*H*)-one (9)

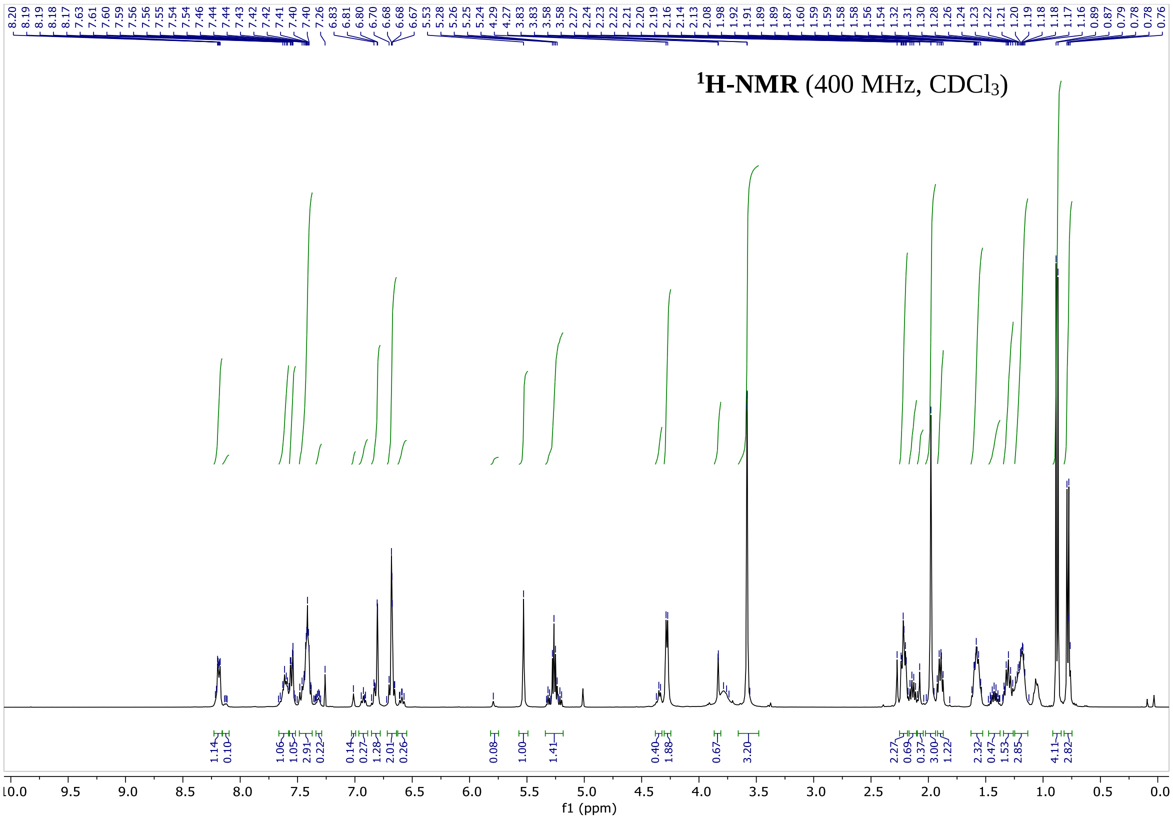

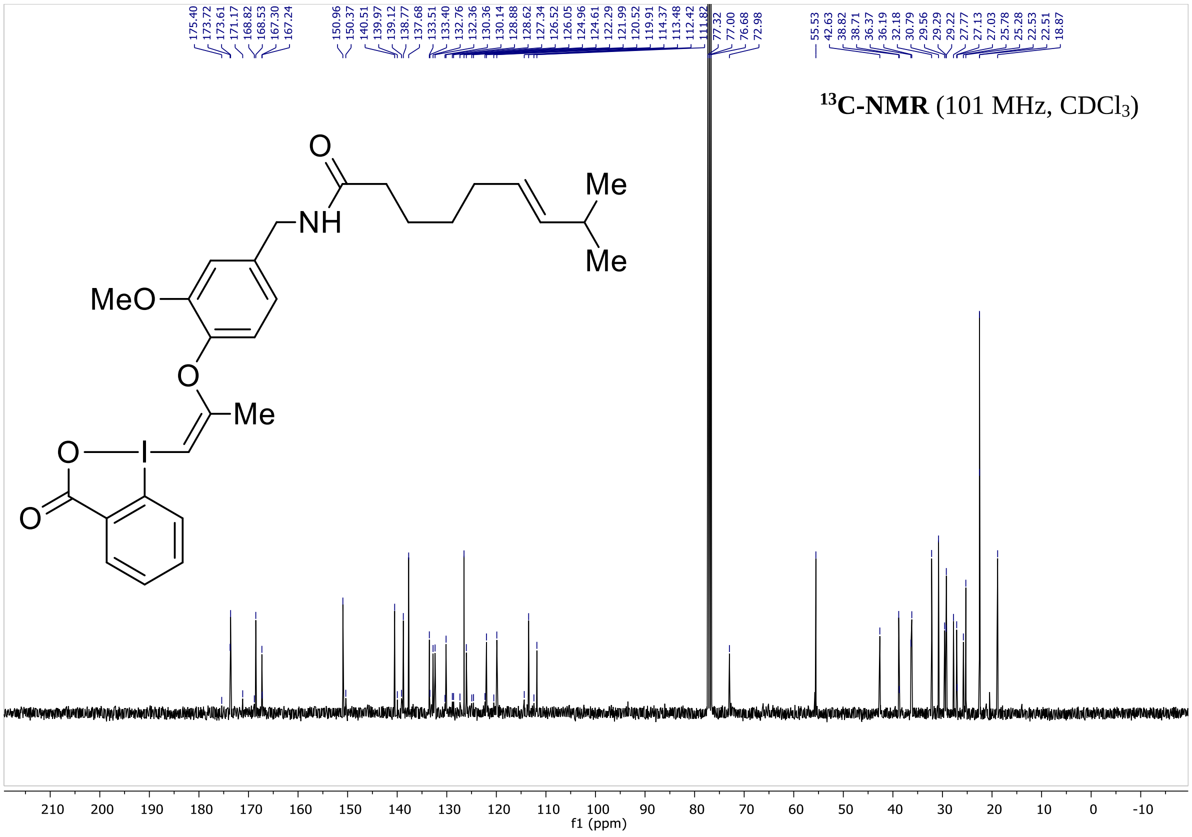

(Z)-(1-pent-1-en-2-yl)-2-Estradiol-1,2-benziodoxol-3-(1*H*)-one (10)

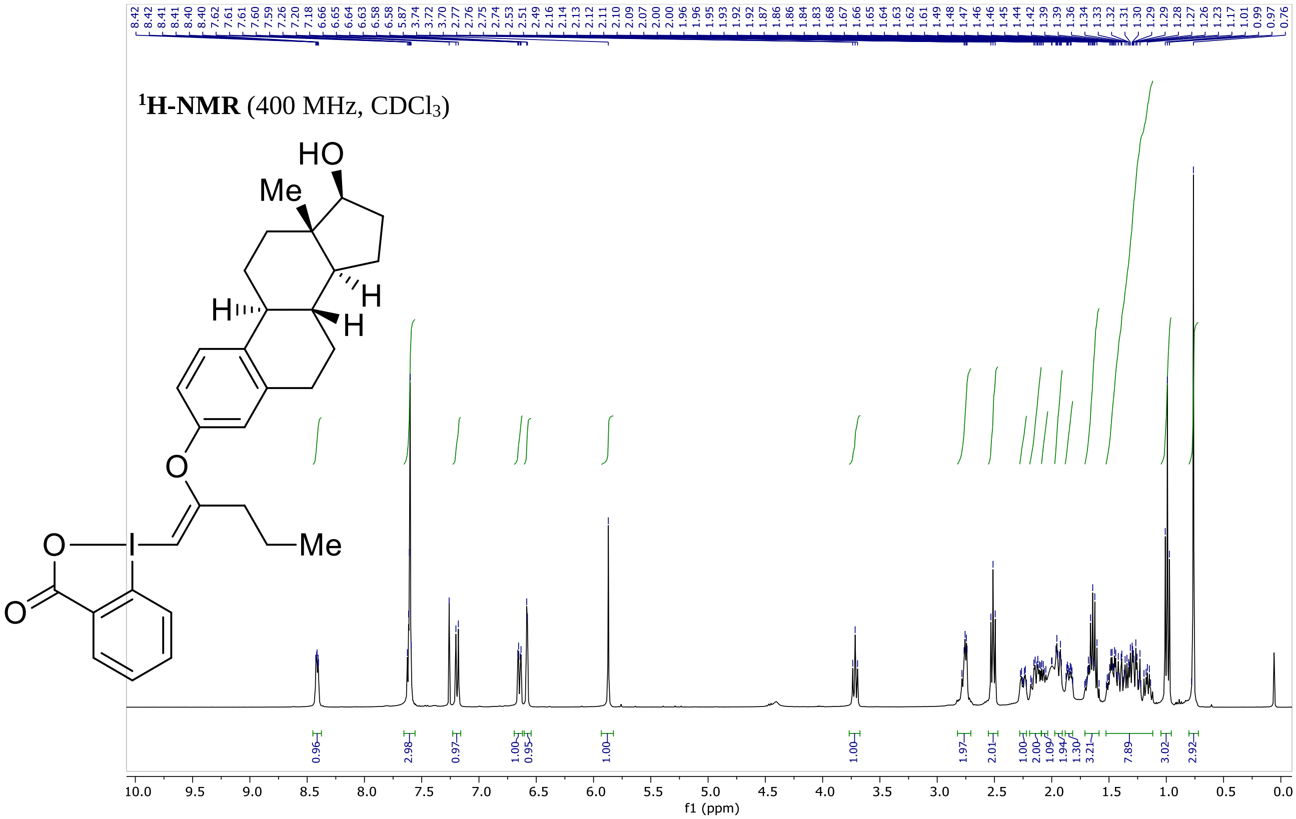

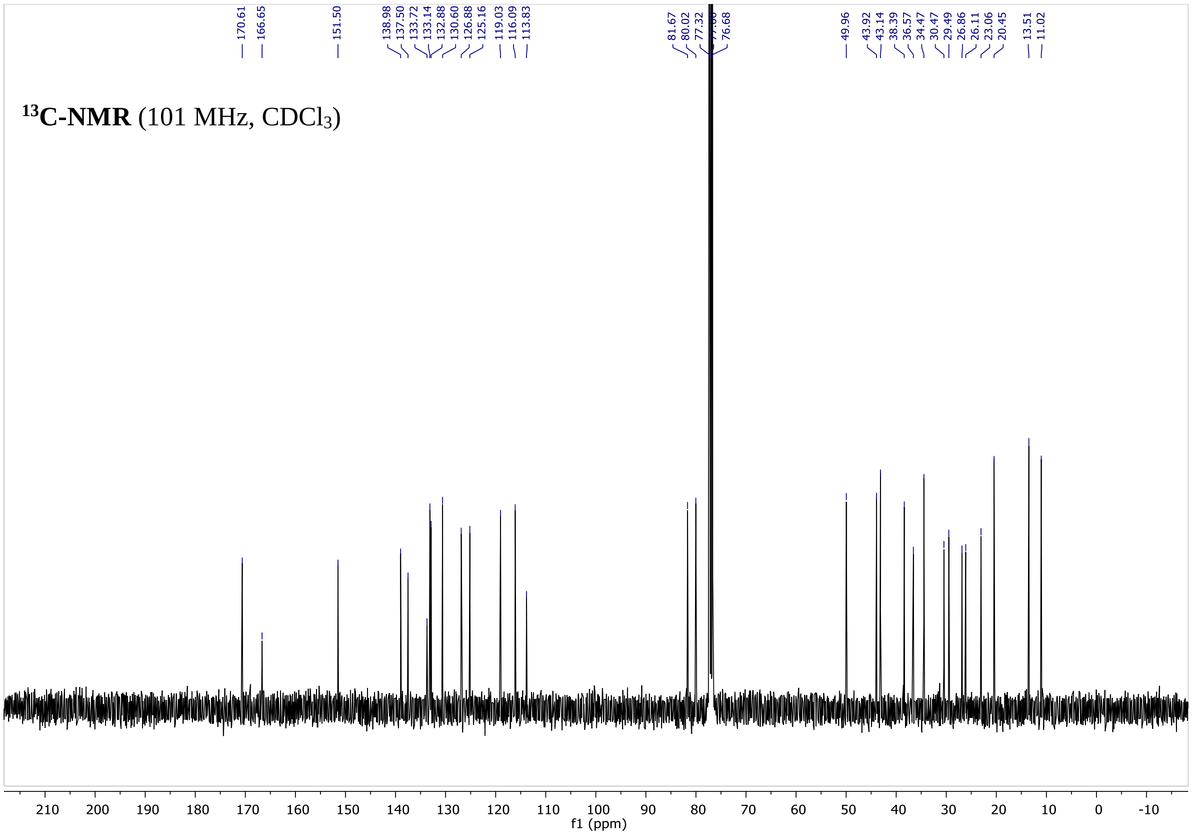

(Z)-*N*-(5-chloro-1-pent-1-en-2-yl)-*N*-Valsartan-1,2-benziodoxol-3-(1*H*)-one (11)

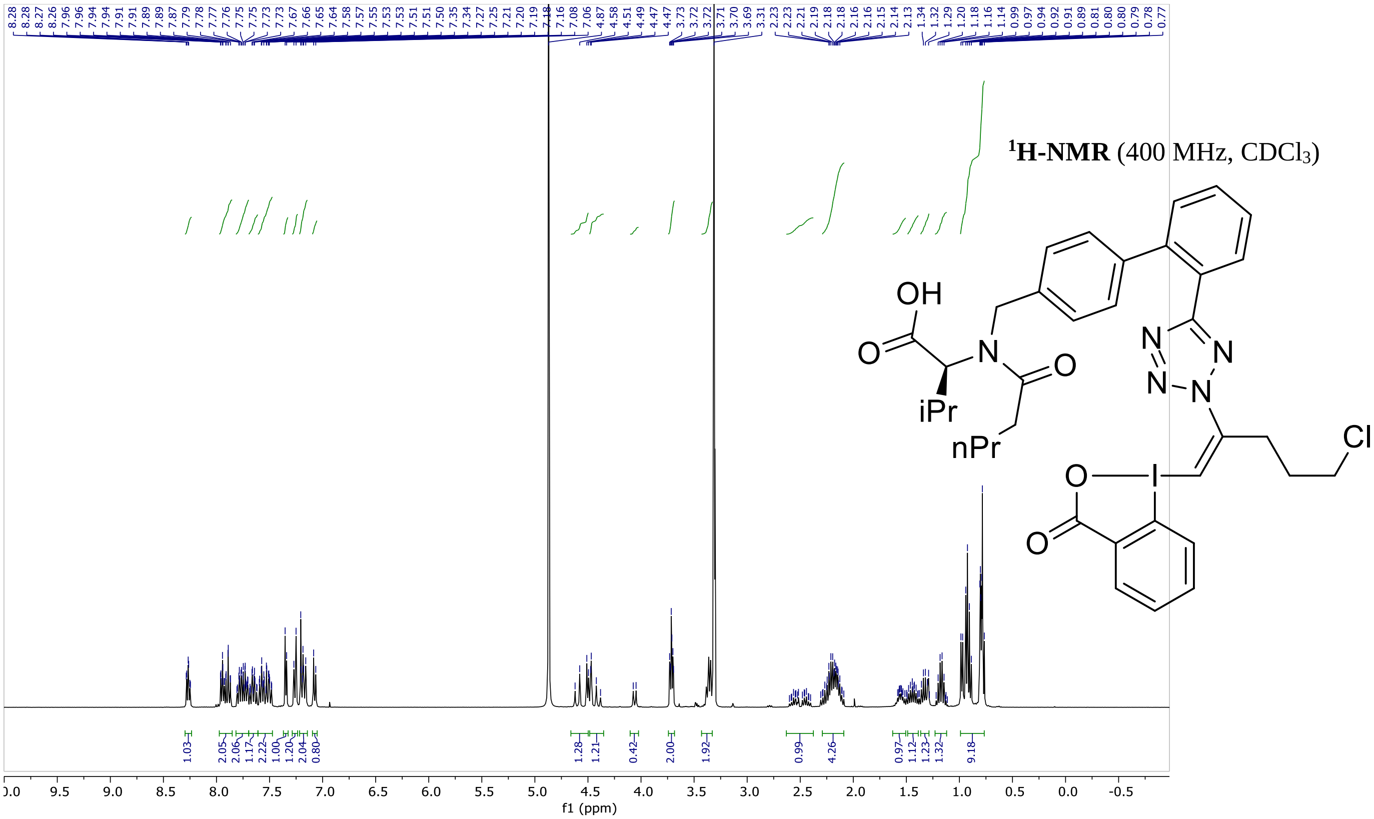

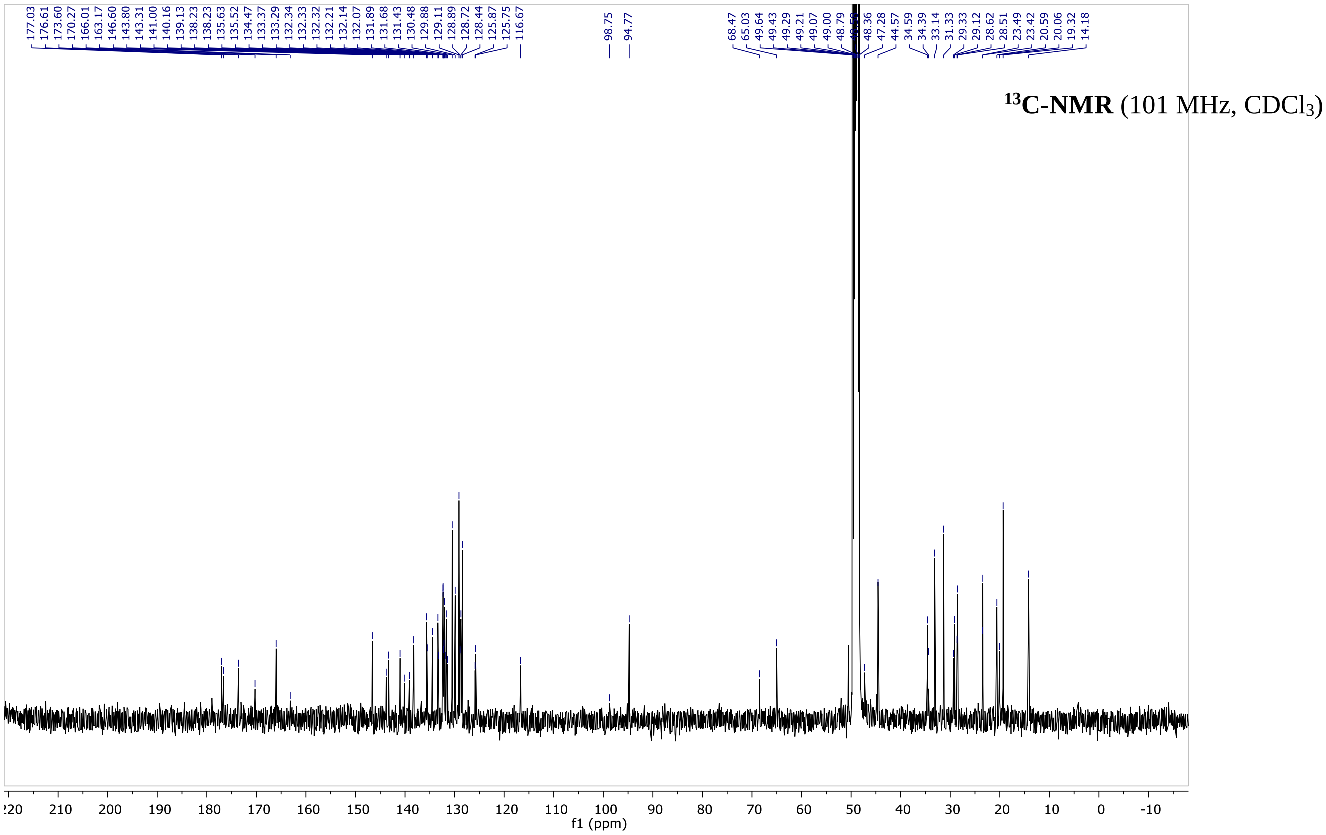

Z)-*N*-(buta-1,3-dien-1-yl)-N-(4-methoxyphenyl)-4-methylbenzenesulfonamide (13)

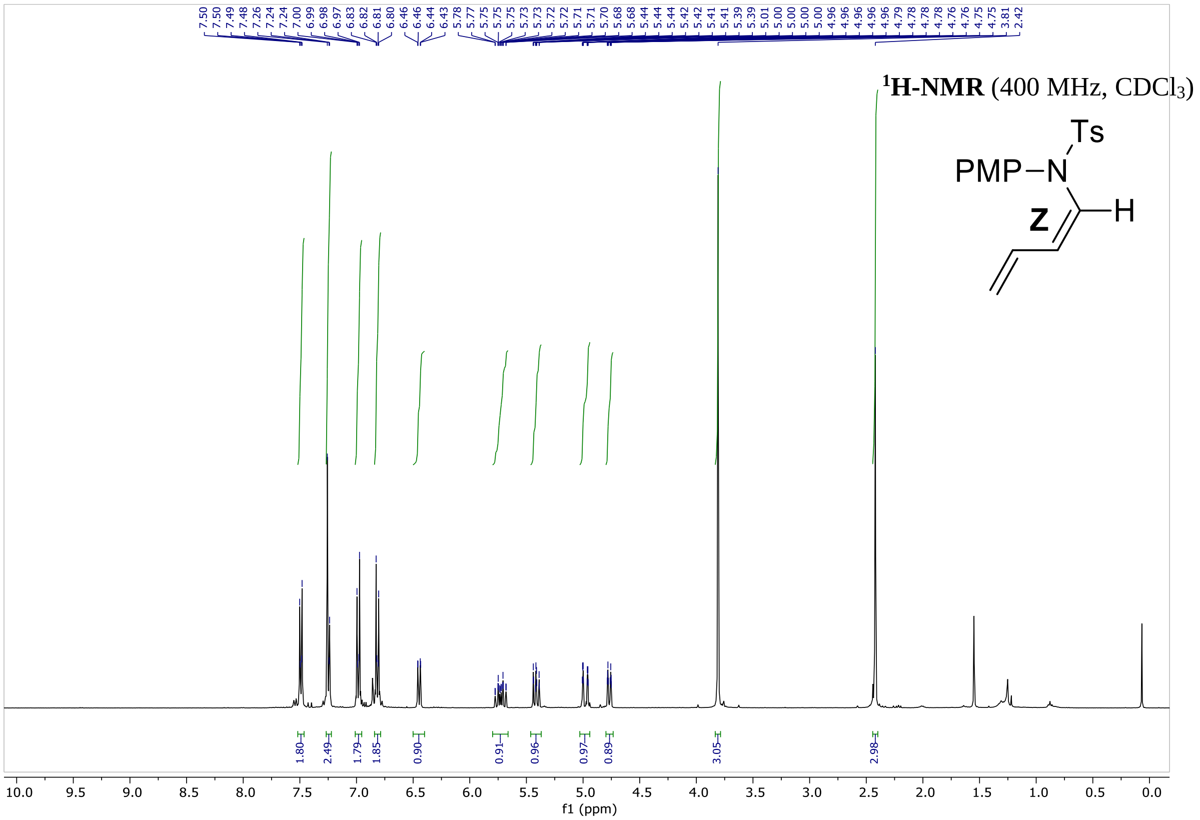

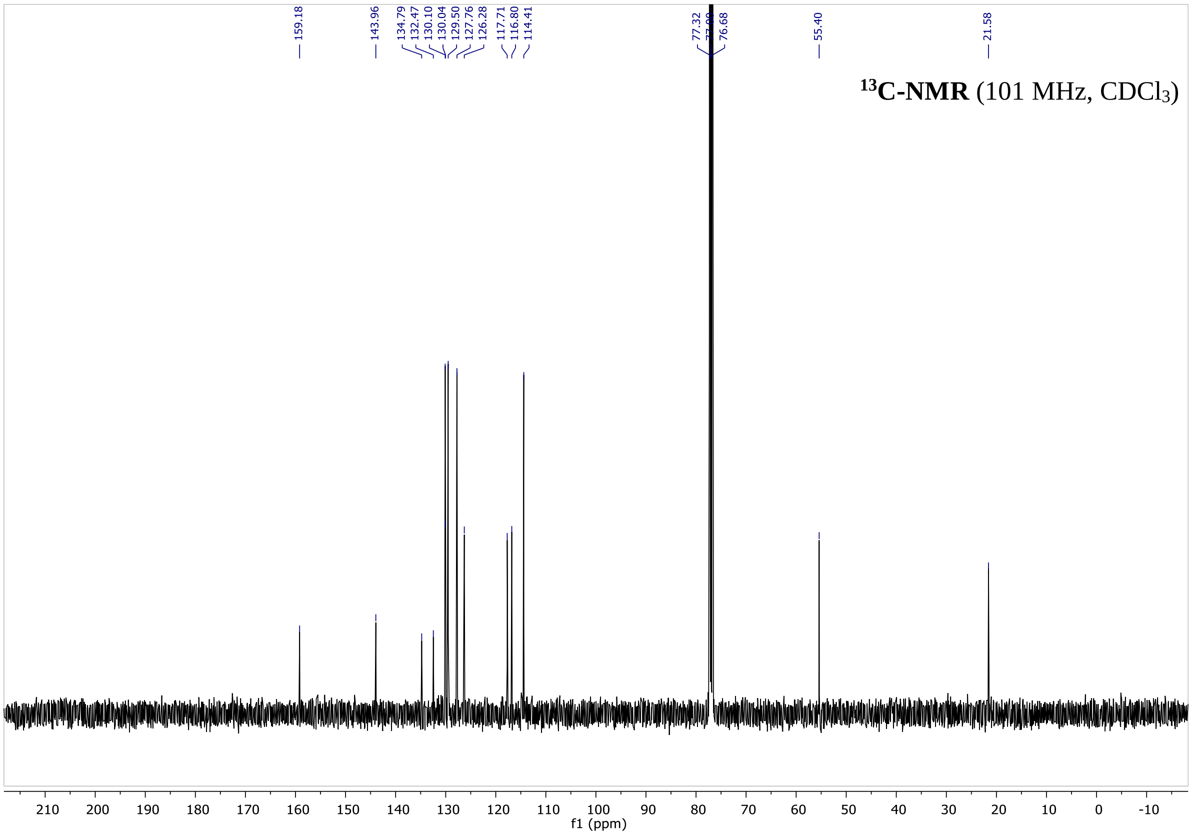

(Z)-*N*-(Cyclopentylbuta-1,3-dien-1-yl)-N-(4-methoxyphenyl)-4-methylbenzenesulfonamide (14)

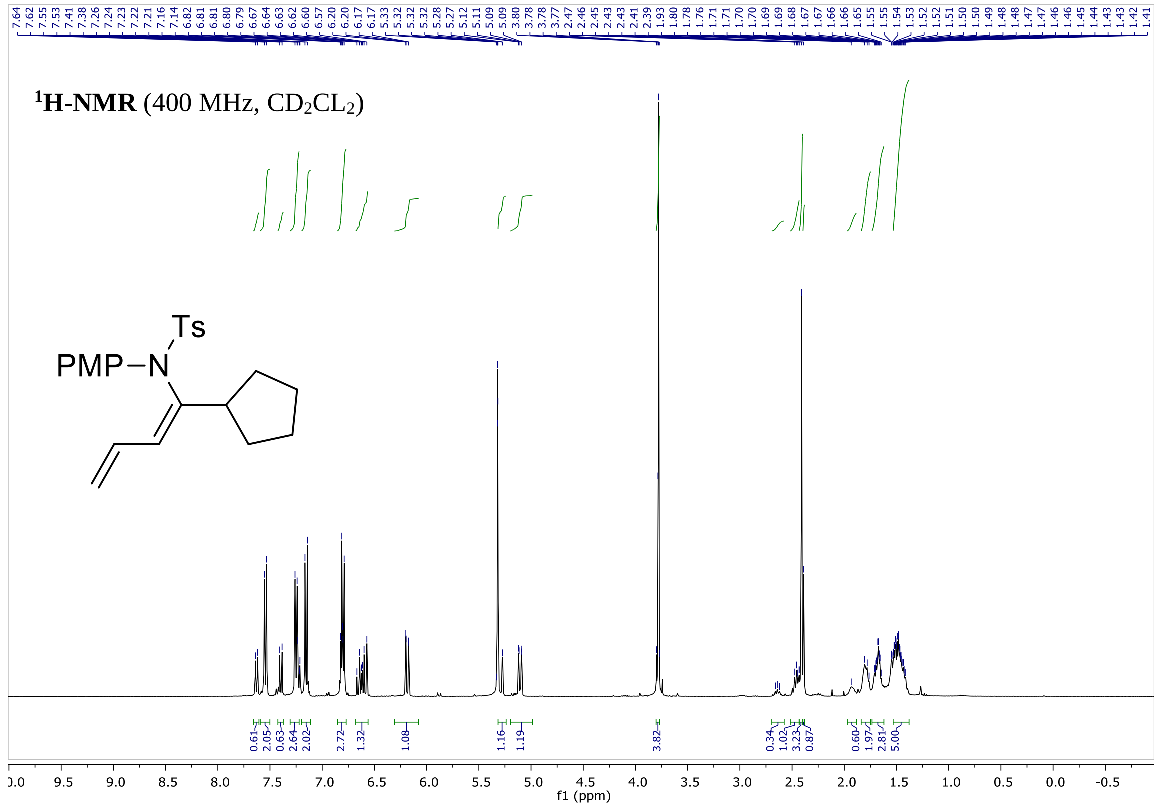

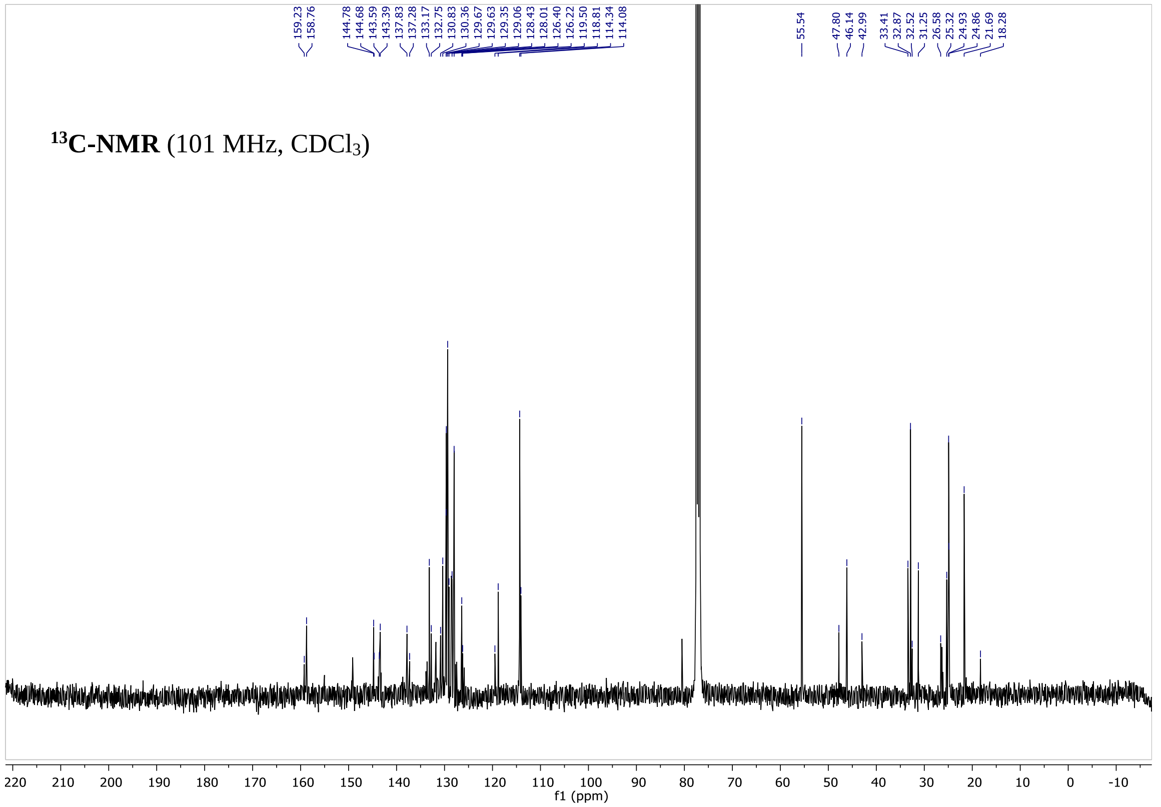

(Z)-*N*-(4-methoxyphenyl)-4-nitro-*N*-(1-phenylprop-1-en-2-yl)benzenesulfonamide (15)

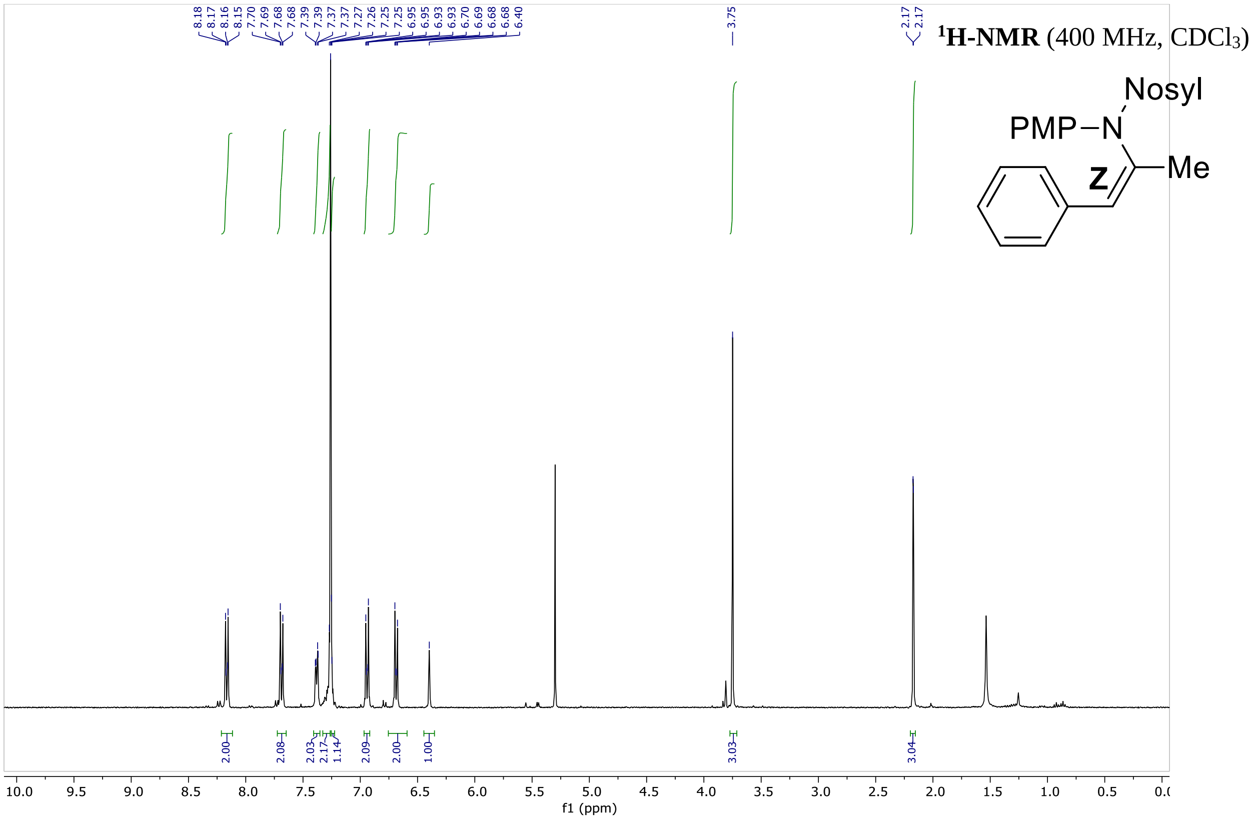

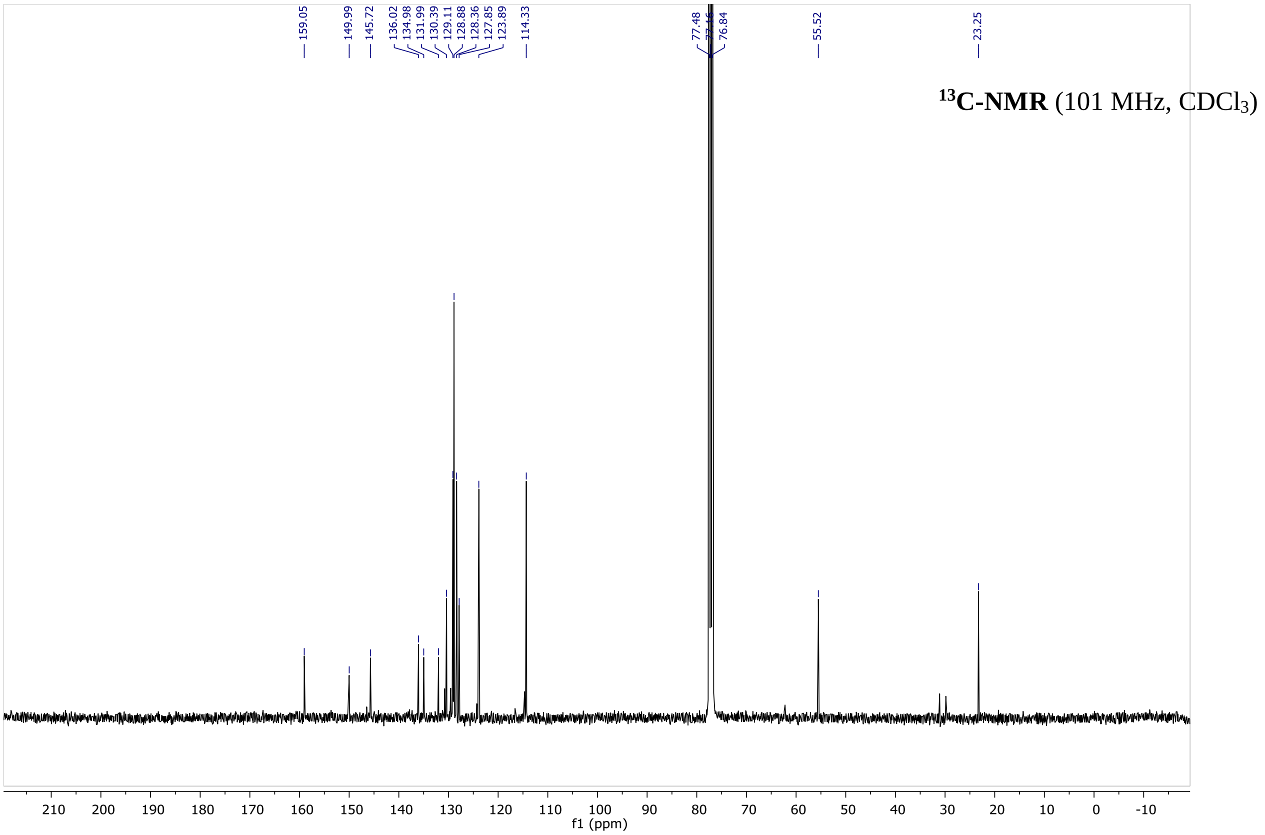

(Z)-*N*-(1-cyclopropylhex-1-en-1-yl)-*N*-(4-methoxyphenyl)-4-methylbenzenesulfonamide (16)

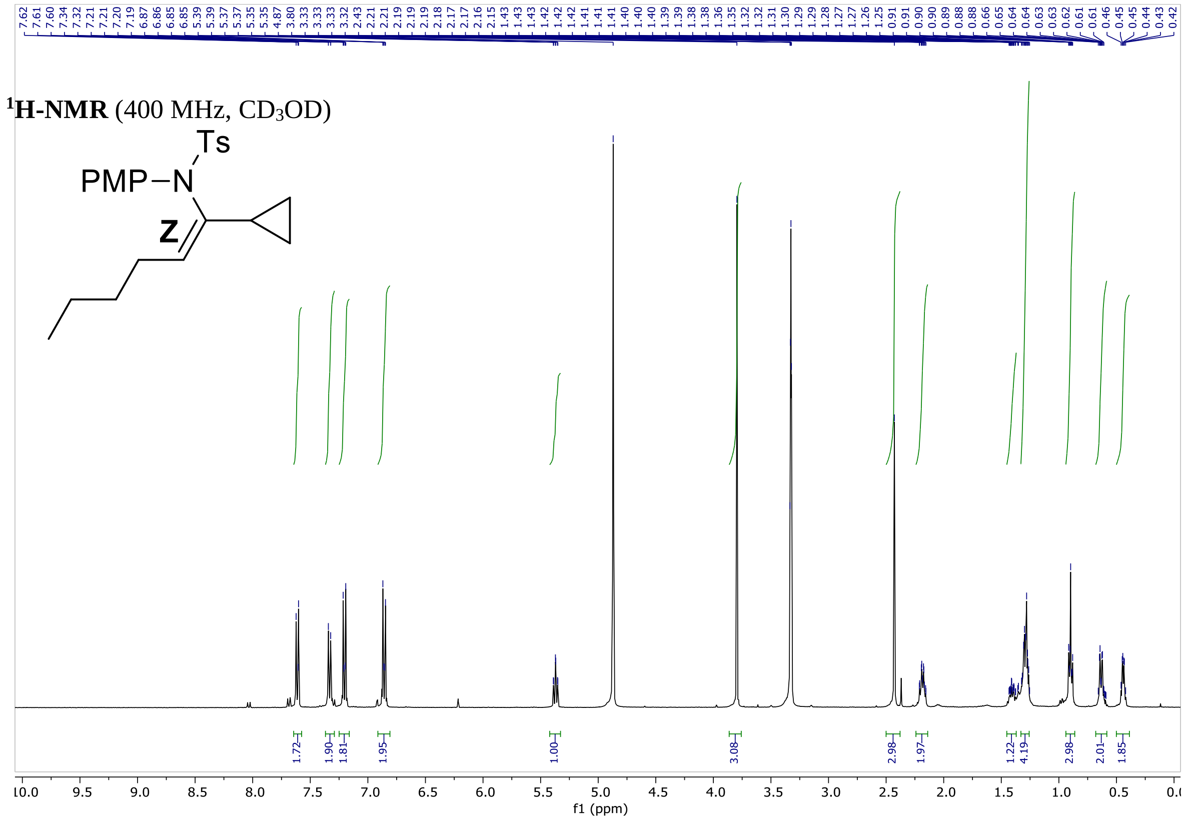

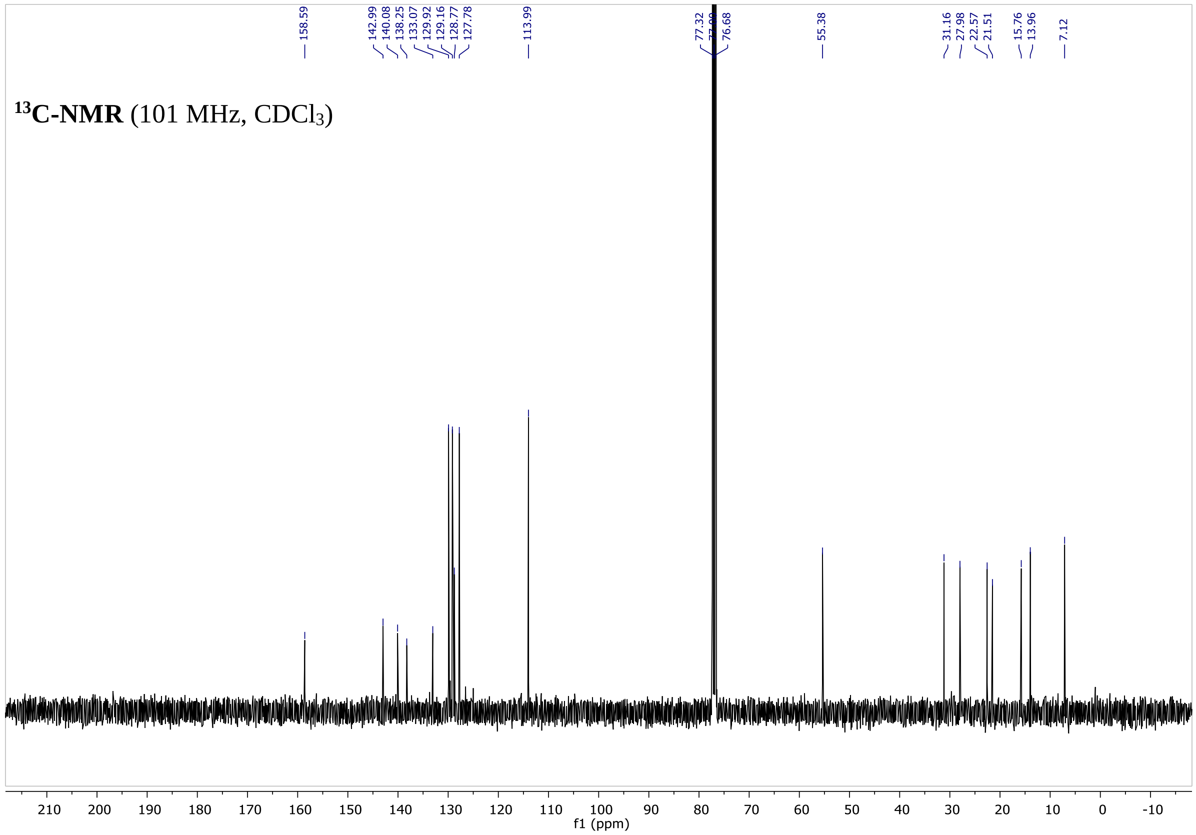

(8*R*,9*S*,13*S*,14*S*,17*S*)-3-((Z)-hepta-1,3-dien-4-yloxy)-13-methyl-7,8,9,11,12,13,14,15,16,17-decahydro-6*H*-cyclopenta[*a*]phenanthren-17-ol (17)

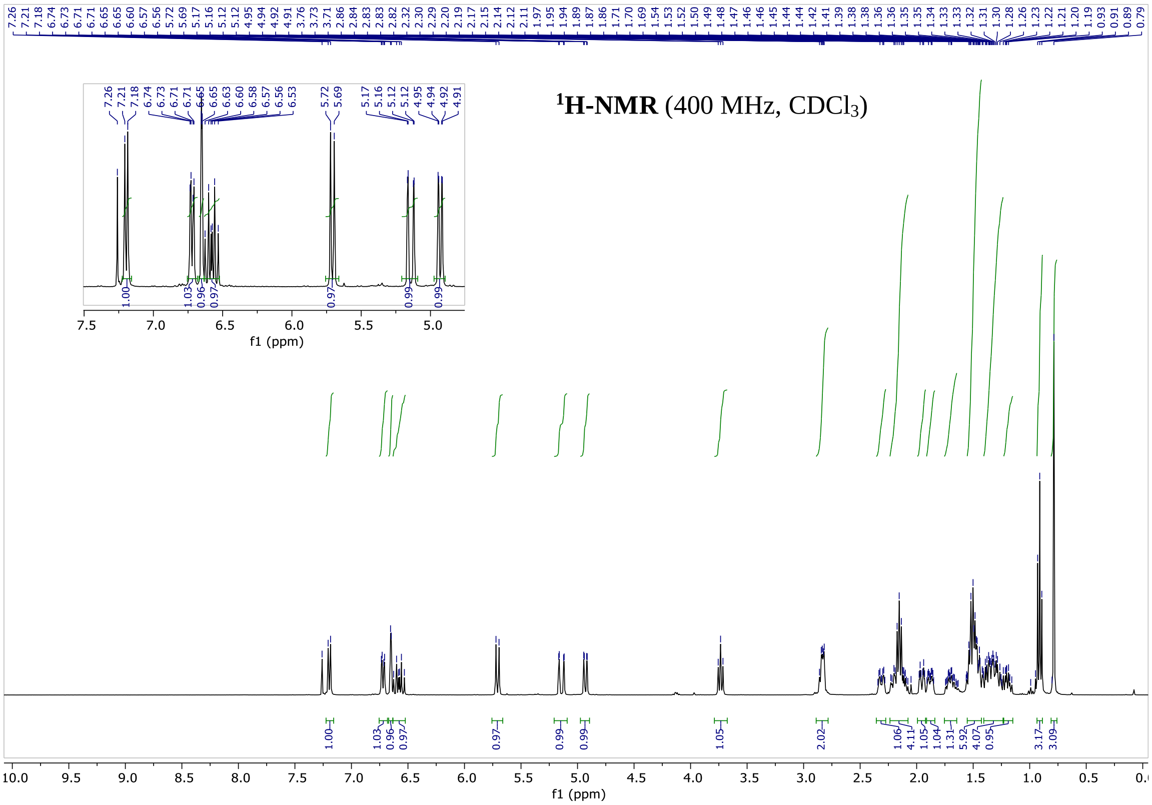

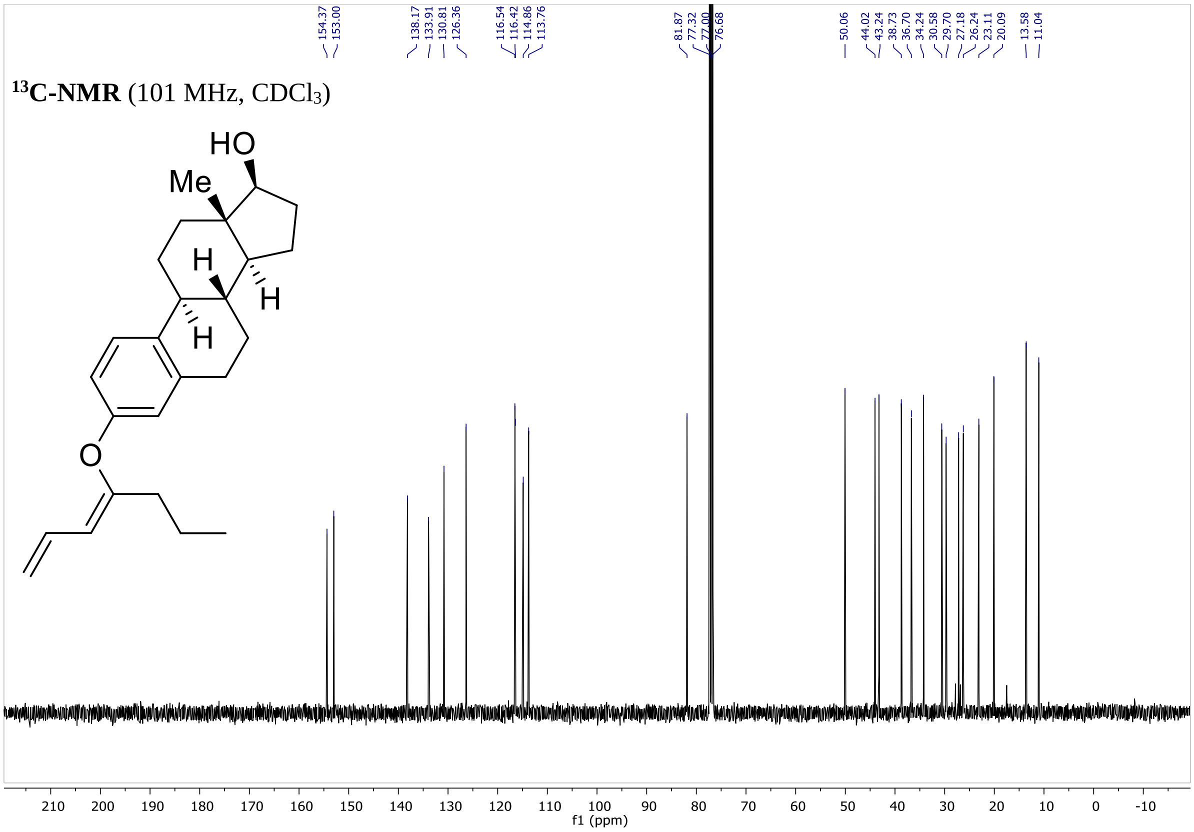

(Z)-*N*-(1-methyl-4-(trimethylsilyl)but-1-en-3-yn-1-yl)-*N*-(4-methoxyphenyl) -4-methyl benzenesulfonamide (18)

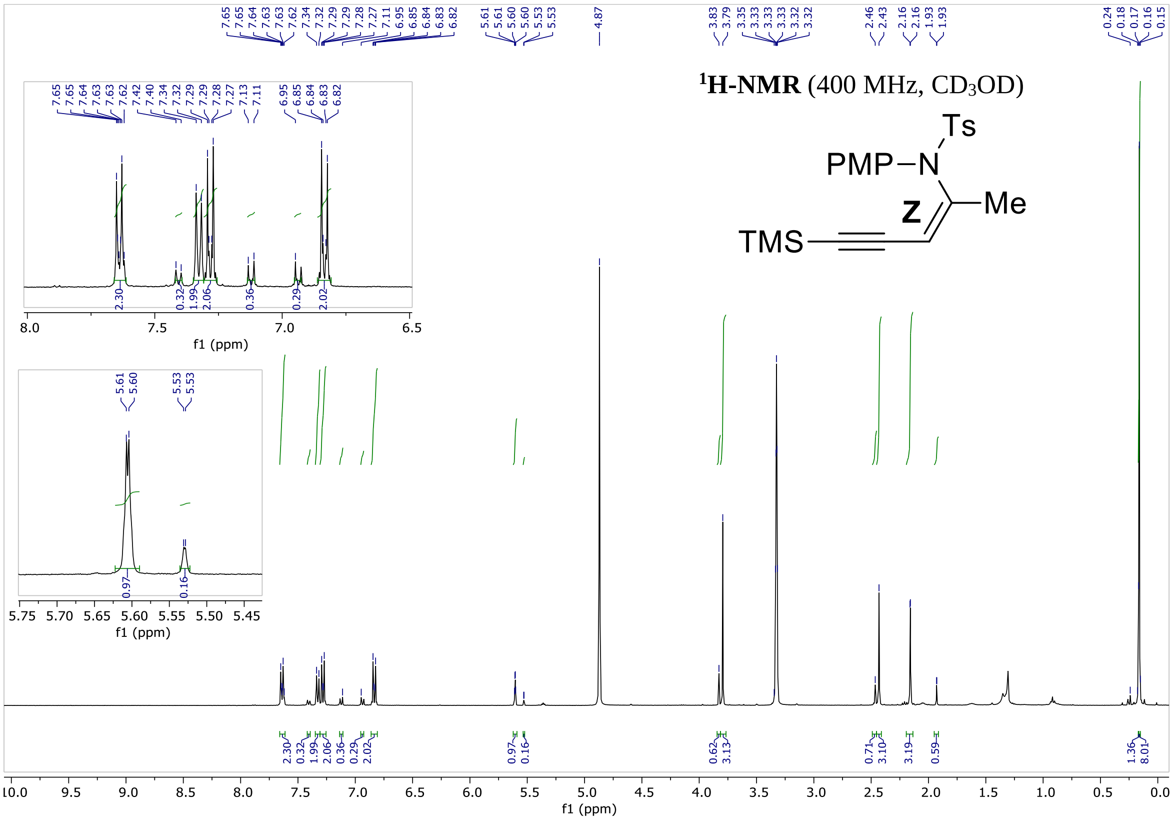

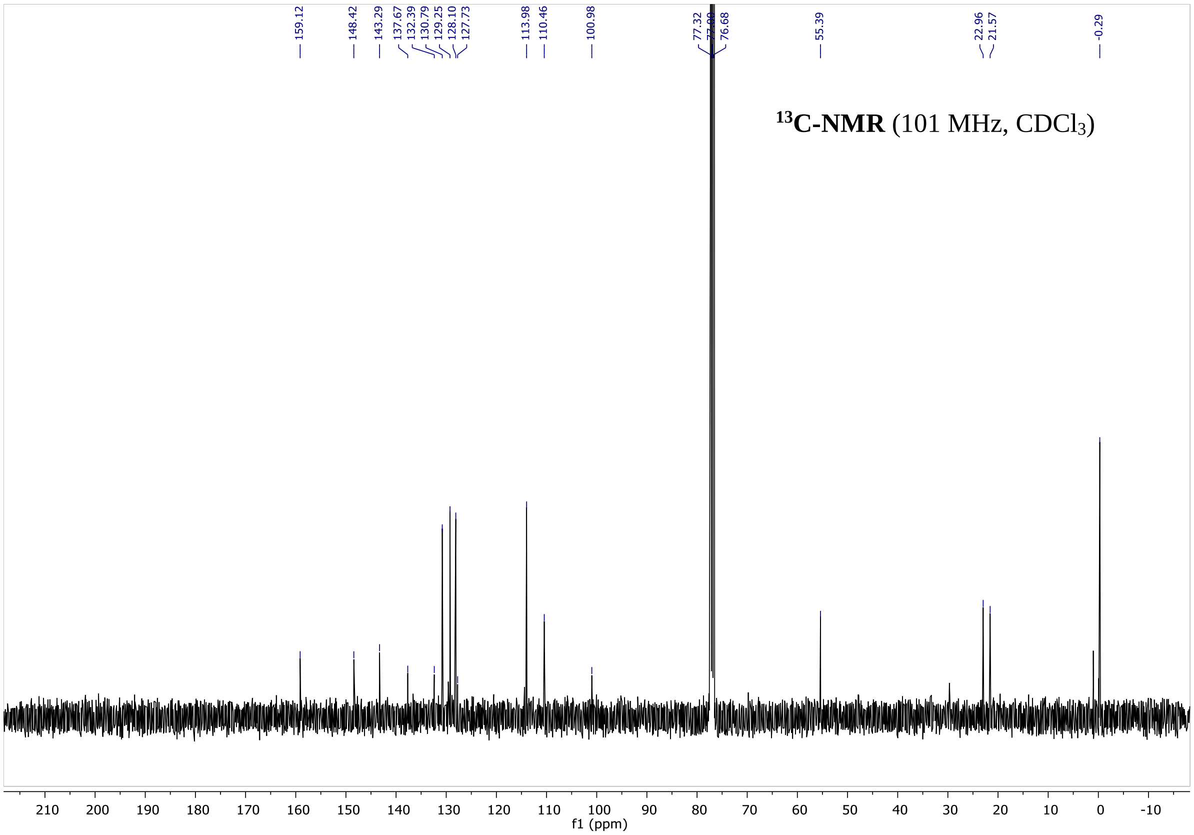

(Z)-*N*-(1-cyclohexyl-2-(phenylthio)vinyl)-*N*-(4-methoxyphenyl)-4-methylbenzene-sulfonamide (19)

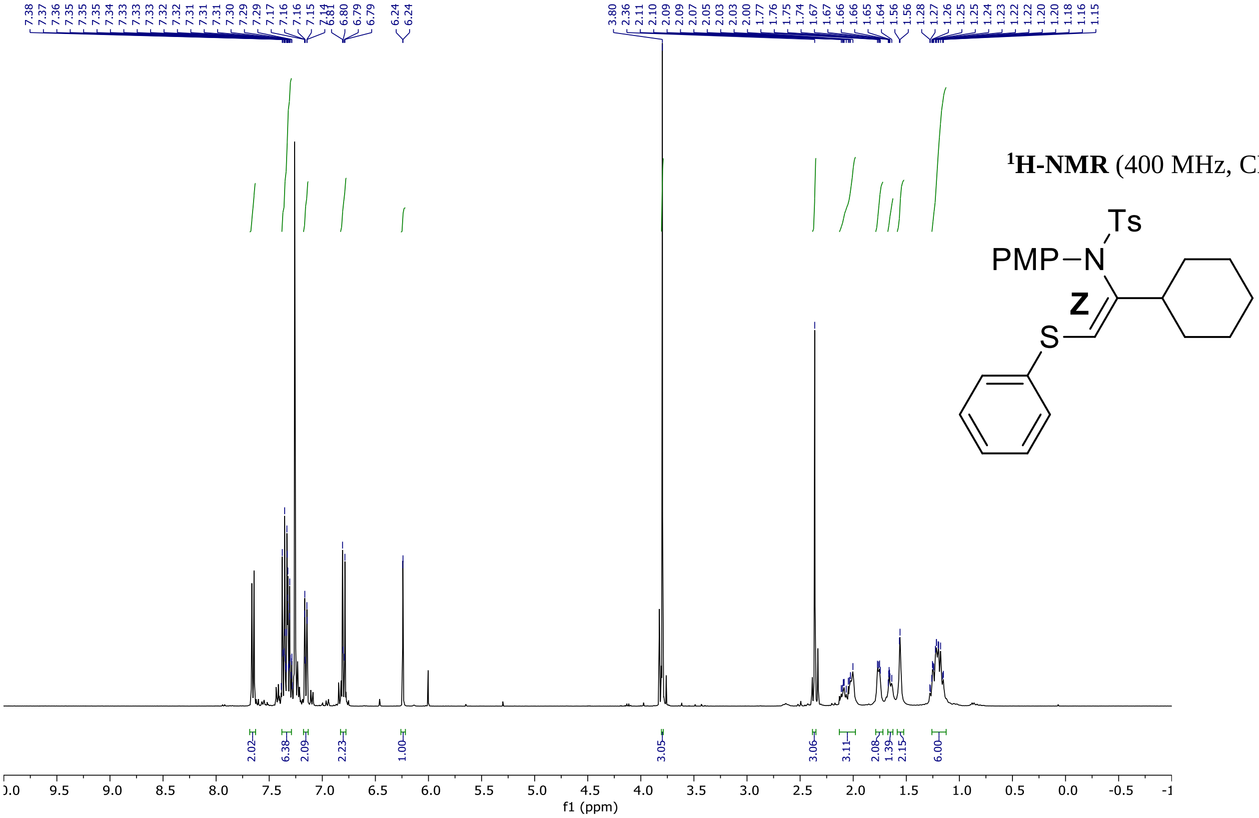

^{1}H-NMR (400 MHz, $CDCl_3$)

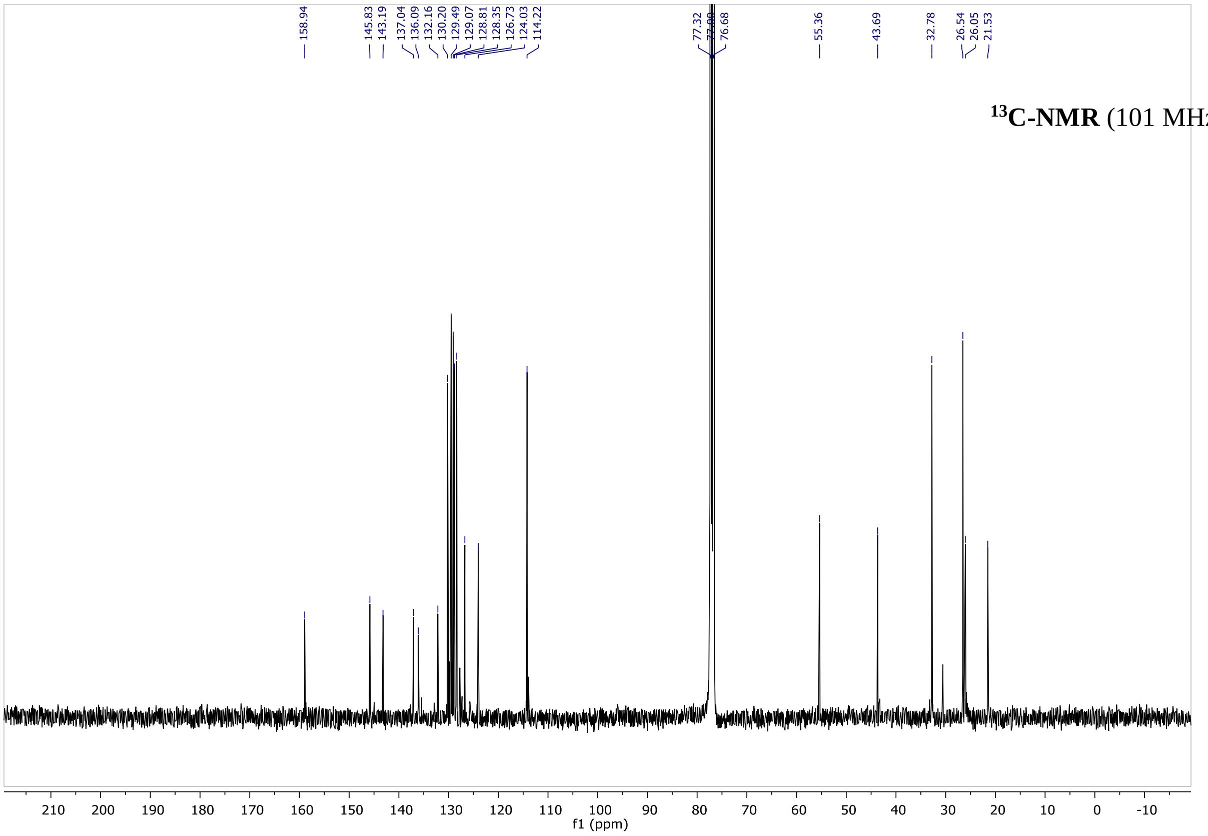

^{13}C-NMR (101 MHz, $CDCl_3$)

(Z)-*N*-(1-cyclopentyl-2-iodovinyl)-*N*-(4-methoxyphenyl)-4-methylbenzenesulfonamide (20)

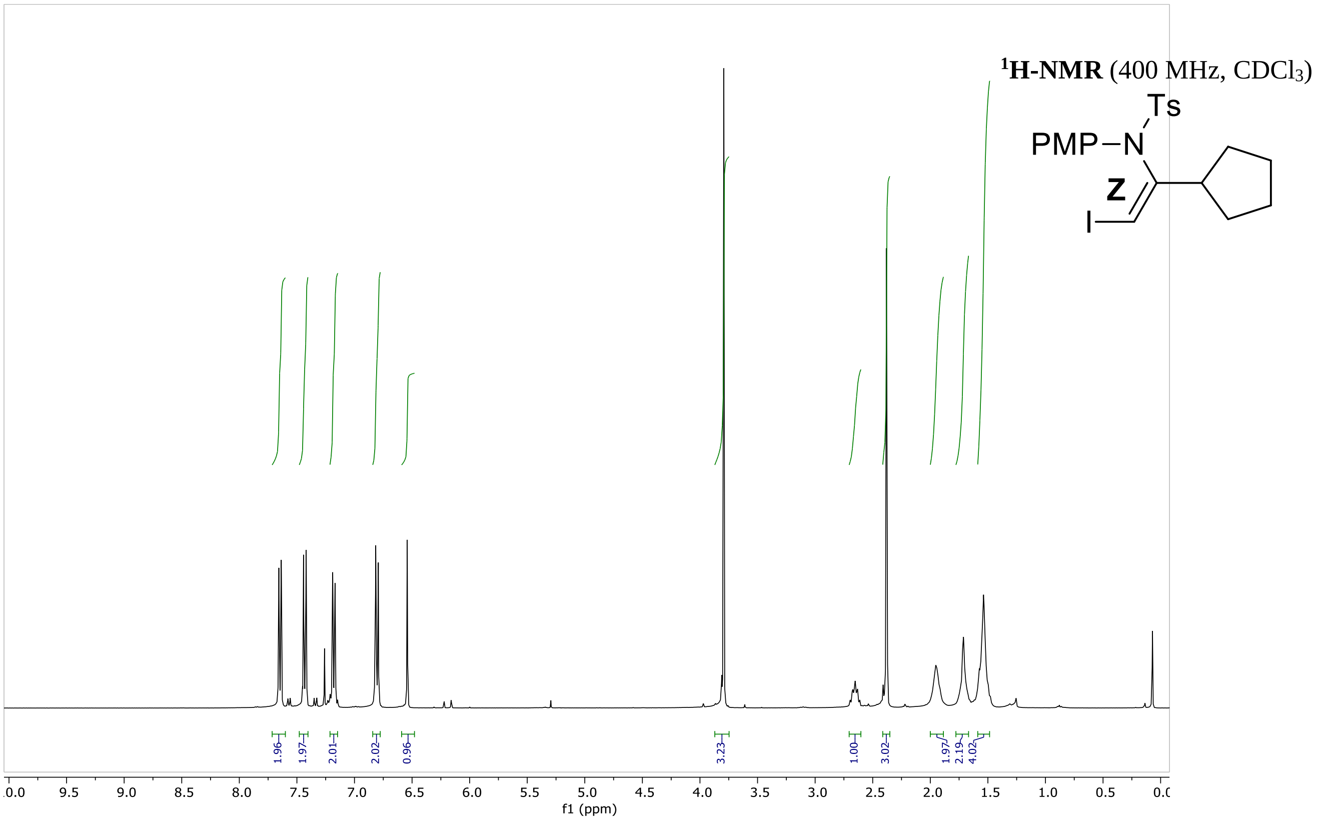

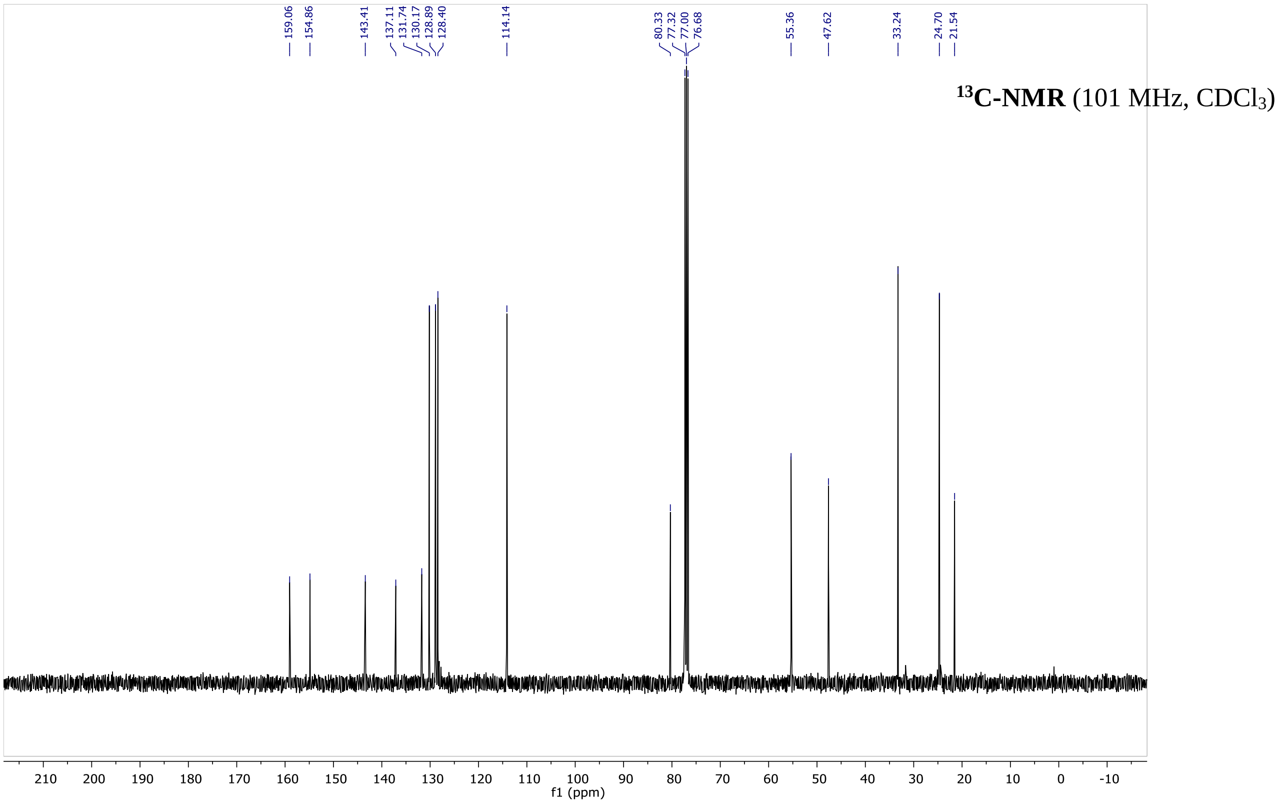

Supplement: Supplementary file 1 [file SC-010-C8SC05573D-s001.pdf]
